# Inequalities in zoster disease burden: a population‐based cohort study to identify social determinants using linked data from the U.K. Clinical Practice Research Datalink†

**DOI:** 10.1111/bjd.16399

**Published:** 2018-04-19

**Authors:** A. Jain, A.J. van Hoek, J.L. Walker, H.J. Forbes, S.M. Langan, A. Root, L. Smeeth, S.L. Thomas

**Affiliations:** ^1^ Faculty of Epidemiology and Population Health London School of Hygiene and Tropical Medicine Keppel Street London WC1E 7HT U.K.; ^2^ Statistics, Modelling and Economics Department Public Health England 61 Colindale Avenue London NW9 5EQ U.K.

## Abstract

**Background:**

Zoster vaccination was introduced in England in 2013, where tackling health inequalities is a statutory requirement. However, specific population groups with higher zoster burden remain largely unidentified.

**Objectives:**

To evaluate health inequalities in zoster disease burden prior to zoster vaccine introduction in England.

**Methods:**

This population‐based cohort study used anonymized U.K. primary care data linked to hospitalization and deprivation data. Individuals aged ≥ 65 years without prior zoster history (*N* = 862 470) were followed from 1 September 2003 to 31 August 2013. Poisson regression was used to obtain adjusted rate ratios (ARRs) for the association of sociodemographic factors (ethnicity, immigration status, individuals' area‐level deprivation, care home residence, living arrangements) with first zoster episode. Possible mediation by comorbidities and immunosuppressive medications was also assessed.

**Results:**

There were 37 014 first zoster episodes, with an incidence of 8·79 [95% confidence interval (CI) 8·70–8·88] per 1000 person‐years at risk. In multivariable analyses, factors associated with higher zoster rates included care home residence (10% higher vs. those not in care homes), being a woman (16% higher vs. men), nonimmigrants (~30% higher than immigrants) and white ethnicity (for example, twice the rate compared with those of black ethnicity). Zoster incidence decreased slightly with increasing deprivation (ARR most vs. least deprived 0·96 (95% CI 0·92–0·99) and among those living alone (ARR 0·96, 95% CI 0·94–0·98). Mediating variables made little difference to the ARR of social factors but were themselves associated with increased zoster burden (ARR varied from 1·11 to 3·84).

**Conclusions:**

The burden of zoster was higher in specific sociodemographic groups. Further study is needed to ascertain whether these individuals are attending for zoster vaccination.

## Abstract 2

**What's already known about this topic?**

- Monitoring and reducing health inequalities is a statutory requirement in the U.K.
- Incidence of herpes zoster is known to be higher among individuals with certain comorbidities and those taking immunosuppressive treatment.
- In contrast, little is known about the social determinants of zoster, which may contribute to the inequality of zoster disease burden in England.

**What does this study add?**

- Older individuals at higher risk of zoster included women, those in care homes, those of white ethnicity and nonimmigrants.
- Individuals with certain comorbidities and taking immunosuppressive therapies were at appreciable increased risk of zoster, but this explained little of the increased risk in the specific demographic and social groups.
- The findings should inform possible future use of the inactivated zoster vaccine among those at higher risk, including care home residents.

https://doi.org/10.1111/bjd.16751 available online

https://goo.gl/Uqv3dl

Herpes zoster is associated with appreciable morbidity, and postherpetic neuralgia (PHN, its commonest complication) can be an incapacitating condition.^1^, ^2^ Zoster can be precipitated by immunosenescence or suppression of cell‐mediated immunity, and incidence increases with age, with rates varying from 6–8 per 1000 person‐years to 8–12 per 1000 person‐years among those aged 60 and 80 years, respectively.^1^, ^2^, ^3^, ^4^, ^5^ Antiviral medications may limit zoster symptoms but their effect on PHN remains uncertain.^6^ A single‐dose vaccine that is effective in preventing zoster and PHN was introduced in the U.K. in 2013, targeting individuals aged 70–79 years.^7^, ^8^, ^9^, ^10^

The effect of sociodemographic factors on zoster incidence has not been extensively investigated. Older individuals and those of white ethnicity have been shown to be at higher risk of zoster.^4^, ^11^, ^12^ The association with other sociodemographic factors such as sex, country of birth, marital status and socioeconomic status has been inconclusive.^3^, ^4^, ^5^, ^12^, ^13^, ^14^ The unequal distribution of zoster between social groups could potentially be exacerbated by differential uptake of zoster vaccination, resulting in even higher disease burden among some older individuals. Identifying social groups at higher zoster risk is therefore essential for planning preventative interventions to mitigate inequalities of zoster burden and promote healthy ageing across the population.

In England, zoster incidence trends are monitored using general practice data to assess the impact of the vaccination programme, but apart from sex no other social factors are being evaluated.^15^ We have shown that some of these factors can be ascertained using routinely collected electronic health records such as the Clinical Practice Research Datalink (CPRD),^16^ one of the world's largest anonymized primary care data sources.^17^

The aim of this cohort study was to determine the association of sociodemographic factors with zoster incidence in England using linked CPRD data in the period immediately before zoster vaccine introduction, to inform suitable targeted vaccination strategies.

## Patients and methods

### Data sources

This study used anonymized CPRD data linked to hospitalization and deprivation data. CPRD provides quality‐assured clinical, lifestyle, demographic and administrative data, for a representative sample of ~7% of the U.K. population.^17^, ^18^, ^19^ The validity of recorded diagnoses within the database is generally high.^18^ In England, CPRD provides linkages at an individual level with other data, including hospitalization [Hospital Episode Statistics (HES)] and deprivation data, for ~75% of practices.^17^, ^18^ The deprivation data were based on a patient's area of residence and/or practice location [Index of Multiple Deprivation (IMD): a composite small area‐level score].^17^, ^18^

### Study population and follow‐up

This cohort study spanned the 10‐year period (1 September 2003 to 31 August 2013) prior to zoster vaccine introduction. The study population comprised patients aged ≥ 65 years who were registered with a CPRD practice in England during the study period and eligible for linkage. Patients with zoster or PHN codes prior to start of follow‐up, or whose first zoster code during follow‐up was PHN, were identified in CPRD and HES [using Read codes for CPRD and International Classification of Disease (ICD) 10th revision codes for HES, Appendix S1] and excluded.

Follow‐up started on the latest of the following: study start date (1 September 2003); 1 year after current registration date (to avoid retrospective recording of past zoster in first few months of new registration);^20^ the date the practice met CPRD's quality criteria;^18^ or individuals' 65th birthday. Follow‐up ended on the earliest of the following: study end date (31 August 2013); zoster date; transfer‐out date; last collection date from the practice; or date of death.

### Outcome

Incident zoster cases were defined as the first diagnostic code for zoster during follow‐up, using both CPRD and HES data (Appendix S1; see Supporting Information). In the HES data, patients with zoster codes in either the primary or the secondary diagnosis fields were included, and the hospital episode start date was used as the date of zoster.

### Exposures

A conceptual framework (Appendix S2), based on the World Health Organization's Commission on Social Determinants of Health framework, was hypothesized for the association of individual‐level sociodemographic factors with zoster incidence.^21^ These factors included immigration status, religion, ethnicity, deprivation, care home residence, marital status, cohabitation (individuals living as a couple) and living alone. The latter three factors provided overlapping information about an individual's living arrangements. The code lists and details of how these factors were identified in linked CPRD data are provided in Appendices S3 and S4. The following factors were categorized as binary variables: being an immigrant to the U.K., care home residence, cohabitation and living alone. Marital status had six categories: single, married, widowed, divorced, separated, and uncategorized/other partner. Religion and ethnicity had eight (Christian, Buddhist, Hindu, Jewish, Muslim, Sikh, other and no religion) and five (white, south Asian, black, other and mixed) categories, respectively. Social factors that changed with time such as marital status, cohabitation, living alone and care home residence were time‐updated over the study period.

IMD data were available at both individual‐ and practice‐small area‐level as quintiles [1 (least deprived) to 5 (most deprived)]; individuals with missing IMD data were assigned their practice's IMD quintile.

### Other variables

Based on previous data^22^ and the U.K. Green Book^10^ guidance for immunosuppressive conditions considered to be a contraindication to live zoster vaccination, certain predisposing conditions and immunosuppressive medications were postulated as mediators between sociodemographic factors and zoster incidence (Appendix S2). Conditions identified in either CPRD or HES (Appendix S3) that were considered immunosuppressive from the time of recorded diagnosis included rheumatoid arthritis, systemic lupus erythematosus, inflammatory bowel disease, diabetes mellitus, chronic kidney disease, chronic obstructive pulmonary disease, asthma, human immunodeficiency virus infection, other cellular immune deficiency and solid organ transplant. Individuals with haematopoietic stem cell transplant, leukaemia, lymphoma, myeloma and other plasma cell dyscrasias were considered to be immunosuppressed for 24 months following each record.^22^ Information on immunosuppressive medications was extracted using previously described methodology.^22^ Details for identifying immunosuppressive conditions and treatments in these data are provided in Appendices S4–5. All putative mediating variables were categorized as binary variables. Age was categorized in 5‐yearly bands from 65 to 84 years, with a final category of ≥ 85 years. The study period was divided into five approximately equal categories to observe any temporal changes in zoster incidence.

### Analyses

For all sociodemographic factors, the person‐time at risk, number of first zoster cases and missing values were tabulated. Zoster incidence rates for each factor were obtained by dividing the number of zoster cases by the person‐time at risk. The demographic characteristics of individuals with a past history of zoster excluded from the study were compared with individuals included in the study.

Poisson regression was used to estimate incidence of the first zoster episode and 95% confidence intervals (CI). Hypothesis testing was conducted using likelihood ratio tests unless otherwise stated. Age, sex and calendar period were considered as a priori confounders; age and sex were also considered as risk factors in their own right. The effect estimates for all explanatory and hypothesized mediating variables were first examined after adjusting for the three a priori confounders in a minimally adjusted model. A hierarchical approach to causal modelling, based on the hypothesized causal framework between the factors of interest, was then adopted for multivariable analyses (Appendix S6).^23^ The first multivariable model included the a priori confounders, ethnicity and immigration status (model 1); patient‐level IMD was then added (model 2), followed by care home residence and living alone status (model 3). The potential mediating effects of comorbidities and therapeutic agents were examined in models 4 and 5, respectively. Collinearity between closely related factors (for example living arrangements) was assessed by comparing measures of effect and log standard errors of the coefficient in minimally adjusted and multivariable models. For multivariable models, individuals with complete covariate data were analysed. Data were analysed using the Stata 14 software package (StataCorp, College Station, TX, U.S.A.).

### Ethics

The protocol for this research was approved by the Independent Scientific Advisory Committee (ISAC) for Medicines and Healthcare products Regulatory Agency Database Research (protocol number 16_168) and made available to the journal and reviewers during peer review. The study was also approved by the Observational/Interventions Research Ethics Committee of the London School of Hygiene and Tropical Medicine (Reference: 11910).

## Results

**Figure 1:**
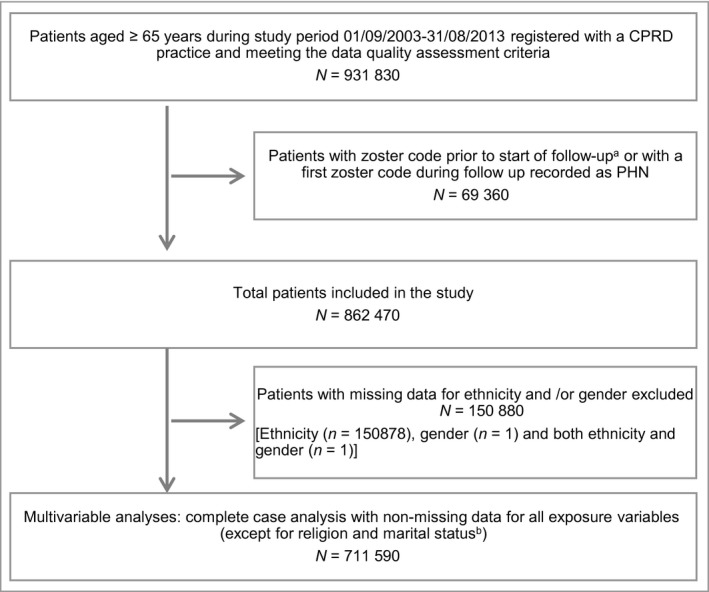
Study participants flow chart. ^a^Follow‐up started on the latest of the following: study start date (1 September 2003); 1 year after current registration date; the date the practice met CPRD's quality criteria; or individuals' 65th birthday. Follow‐up ended on the earliest of the following: study end date (31 August 2013); zoster date; transfer‐out date; last collection date from the practice; or date of death. ^b^Both these variables were excluded from the analyses. CPRD, Clinical Practice Research Datalink; PHN, post‐herpetic neuralgia.

The cohort initially included 931 830 individuals (Fig. 1) from 385 practices across England, of which 69 360 were excluded because of a prior history of zoster. The demographic characteristics of patients with and without a prior zoster history (excluded and included, respectively, in the study) are presented in Appendix S7. Those with a previous history of zoster were likely to be older at the start of the study (1 September 2003), women, nonimmigrants and individuals of white ethnicity, but were similar to included individuals with respect to IMD quintiles.

The median follow‐up time for the eligible study cohort (*N* = 862 470) was 4·33 [interquartile range (IQR)1·82–8·07] years. More than half of the eligible study population were women and were aged 65–69 years at the start of follow‐up (Table S1; see Supporting Information). Data were missing for four variables: religion (98% missing), marital status (47% missing), ethnicity (17·5% missing) and sex (< 0·01% missing) (Table S1). For < 0·1% of the study population (*n* = 849), the missing values of patient‐level IMD were replaced by practice‐level IMD (Table S1).

In total, 37 014 individuals experienced a first zoster episode during follow‐up, the median age at zoster was 75·7 years (IQR 70·2–81·8 years) and the incidence was 8·79 (95% CI 8·70–8·88) per 1000 person‐years at risk (Table S1). The unadjusted zoster rates for all sociodemographic factors and mediating variables are presented in Table S1.

A decision was made to drop the two variables with appreciable missing data (religion and marital status) from further analyses. Ethnicity and sex were retained in the analysis but individuals with missing data were dropped. The complete case analysis included 711 590 patients, after dropping participants with missing data on ethnicity (*n* = 150 878) and/or sex (*n* = 2) (Fig. 1). Demographic characteristics of individuals with and without missing ethnicity data are provided in Appendix S8. Those without ethnicity data were similar to those included in the analysis with only slightly lower zoster incidence and marginally fewer comorbidities (Appendix S8).

In the minimally adjusted model (adjusted for age, sex and calendar period, Table S2), zoster rates increased linearly with age, the adjusted rate ratios (ARRs) in the oldest age groups (aged ≥ 85 years) being 40% higher than for those aged 65–69 years. There was little evidence of changing incidence over the study period. For the sociodemographic factors of interest, men, those who were of non‐white ethnicity, immigrants, those living in more deprived areas (at the practice or patient‐level) and those living alone had lower rates of zoster, with reduced rates of between 5% (for moderate deprivation at the patient‐level) to 53% (for black ethnicity) (all *P*‐values < 0·001). Care home residents had higher zoster incidence (ARR 1·12, 95% CI 1·06–1·18) than non‐care home residents. As expected, most of the comorbidities and all immunosuppressive treatments were associated with higher zoster incidence (Table S2).

After adjusting for immigration status in addition to the a priori confounders (model 1), the effect of ethnicity was almost unchanged, with each non‐white ethnic group remaining at lower risk of zoster [ranging from a 15% reduced rate (other ethnicity) to a 51% reduced rate (black ethnicity), Table S2]. In contrast, the effect estimate for immigration status was slightly attenuated after additionally adjusting for ethnicity, although immigrants remained at 23% lower risk of zoster.

Practice‐ and patient‐level deprivation were closely correlated, as were living alone and cohabitation status; thus, only patient‐level IMD was added to model 2 and living alone to model 3. There were no appreciable changes in the effect estimates for each factor with successive adjustment (Table S2). Those living in a care home had about a 10% higher rate of zoster, and those living alone a reduced rate of 4% (model 3, Table S2). None of the factors appeared to be mediated by comorbidities or immunosuppressive therapies (models 4 and 5, Table S2).

When analyses were repeated for those with missing ethnicity data, effect estimates in the minimally adjusted analysis were all in the same direction of the main analysis (Appendix S9). In further sensitivity analyses, restriction to those of white ethnicity also showed no differences to the main analysis (Appendix S10). Substitution of practice‐ for patient‐level IMD (Appendix S11) and cohabitation instead of living alone (Appendix S12) also did not change the findings.

## Discussion

In this large population‐based cohort study, sociodemographic factors independently associated with higher zoster risk included age, female sex, white ethnicity and care home residence. There was little evidence of increased incidence among minority ethnic groups, those living alone or living in deprived areas. As in previous studies, increasing age, specific comorbidities and immunosuppressive treatments were associated with higher zoster incidence.

This study used one of the world's largest quality‐assured primary care databases, linked to hospitalization and social deprivation data, and provided data for a wide range of social factors, including potential mediating variables for zoster risk. These results could be potentially generalizable to older individuals without prior history of zoster from countries with universal access to health care as in the U.K. Ascertainment of social factors using routinely collected electronic health records was achieved by using detailed coding algorithms and rigorous methodology. Causal modelling based on a predefined conceptual framework ensured quantification of robust effect estimates.

The limitations associated with use of routinely collected data included potential misclassification of both exposure and outcome. Time‐varying exposures such as living alone may be misclassified in these data if not updated in a timely manner in general practitioner records. Similarly, for binary variables, the assumption that absence of a code implies absence of the characteristic may not be true. However, the methods used in this study are based on our previously developed methodology for ascertainment of social factors among older individuals using linked CPRD data, which found prevalence of factors such as ethnicity, living alone, cohabitation and care home residence comparable with the 2011 English Census, whereas being an immigrant was under‐represented in these data.^16^ In the U.K., zoster is mainly diagnosed clinically;^22^ however, a clinical diagnosis of zoster in primary care is reported to have a high (91%) positive predictive value among older individuals.^24^ Any misclassification of exposure or outcome is likely to be nondifferential, tending to bias effect estimates towards the null. Thus, the actual effect estimates may be even larger than observed here.

Differential zoster ascertainment in different social groups is a consideration. For example, it is possible that those living alone or in more deprived areas may not seek care for zoster. Those seeking ongoing care for their comorbidities may have a higher opportunity of zoster diagnosis, although we found no mediating effect of comorbidities for any of the social factors examined. A previous U.S. study reported that 95% of older individuals were likely to consult for zoster, irrespective of sociodemographic characteristics such as income or marital status.^25^ Unavailability of factors such as education, income and social class, and poor recording of religion (2%) and marital status (~50%) in these data precluded the assessment of their association with zoster in this study. Finally, excluding individuals with missing ethnicity data could have biased our results, but further multivariable and sensitivity analyses showed no evidence for this.

The rates in this study are comparable with previously reported zoster rates of 6–8 per 1000 person‐years and 8–12 per 1000 person‐years among individuals aged 60 and 80 years, respectively.^2^ However, the overall incidence of first zoster episodes in this study of individuals aged ≥ 65 years was slightly higher than a U.K.‐based study of only immunocompetent individuals that reported zoster rates of 5·96 (aged 65–69 years) to 6·22 (aged ≥ 85 years) per 1000 person‐years.^26^

Increasing age and female sex are both known to be associated with higher zoster incidence owing to age‐ and sex‐related immune differences and possible differences in health‐seeking behaviour.^2^, ^3^, ^4^ The finding of lower zoster disease burden among individuals of non‐white ethnicity seen in this study has been previously reported.^4^, ^11^, ^12^, ^13^, ^14^ This effect of lower zoster incidence among individuals of non‐white ethnicity could be explained by persisting immunity to zoster in later years because of late‐onset varicella in individuals born in the Caribbean or tropical countries,^3^, ^14^, ^27^, ^28^ although immigrants represent a heterogeneous group in terms of their country of origin and age of arrival in the U.K. There was little evidence of collinearity between ethnicity and immigration status, and the finding of lower zoster incidence among those of non‐white ethnicity, independent of immigration status, could perhaps be explained by social mixing patterns in extended families leading to varicella contacts and boosting of zoster immunity among older individuals.^29^ It is also possible that zoster might be underdiagnosed if individuals of non‐white ethnicity who developed zoster were less likely to consult or less likely to be diagnosed, but it seems implausible in view of the large effect seen. The association of care home residence with higher zoster incidence was noted independent of age and comorbidities. The effect of malnutrition on immune function could also play an important role; previous research has suggested that nearly one in three care home residents could be at risk of malnutrition.^30^, ^31^, ^32^ The association of higher zoster incidence with certain comorbidities and taking immunosuppressive treatment has been previously reported.^2^, ^3^, ^5^, ^22^, ^33^ Similarly, as reported previously, we also did not find any evidence of an association of diabetes with zoster disease burden.^22^ The association of social support, cohabitation or being married with zoster incidence is conflicting: some studies have reported no association with being married,^5^, ^12^, ^14^ individuals with a confidant having lower^12^ or no effect^14^ on zoster incidence, whereas another study reported higher zoster risk among individuals not cohabiting.^4^ The unexpected finding of an independent association of living alone and lower patient‐level IMD with lower incidence of first zoster episode could perhaps be because of a higher occurrence of zoster among these social groups before the age of 65 years and thus their exclusion from the current study. However, there was no evidence for any appreciable differences in patient‐level IMD scores when the characteristics of individuals with/without prior zoster history were compared.

In conclusion, this large population‐based cohort study has identified social factors associated with zoster incidence among an older population in the U.K. Typically, older patients with higher level of deprivation, those of non‐white ethnicity, immigrants and those living alone are at greater risk of infectious diseases; interestingly, our study found these groups were at lower risk of zoster. However, care home residence was associated with higher zoster burden and it may be worth considering targeted vaccination of this group. Further research on the risk of PHN in these social groups would also help inform vaccination policy. It will be interesting to determine in future studies whether these social groups with higher zoster incidence come forward for zoster vaccination.

## Supporting information


**Table S1** Baseline characteristics of the study cohort (*N* = 862 470, outcome *n* = 37 014).

**Table S2** Multivariable analysis: social factors associated with zoster disease incidence (complete case analysis; individuals with missing data for ethnicity and sex excluded) (*N* = 711 590, outcome *n* = 32 459).

**Appendix S1** Code list: zoster.

**Appendix S2** Conceptual hierarchical framework for the association of social factors with zoster disease burden.

**Appendix S3** Code lists: social factors, comorbidities and medications.

**Appendix S4** Identification of exposures, comorbidities and medications in Clinical Practice Research Datalink and Hospital Episode Statistics.

**Appendix S5** Immunosuppressive medications and conditions: defining periods of immunosuppression.

**Appendix S6** Inclusion of explanatory variables in causal modelling based on a hierarchical framework.

**Appendix S7** Comparison of patients excluded due to prior history of zoster and patients included in the study.

**Appendix S8** Baseline characteristics of patients excluded from analysis due to missing data for ethnicity and included in complete case analysis.

**Appendix S9** Multivariable analysis: social factors associated with zoster disease incidence among patients excluded from analysis due to missing data for ethnicity.

**Appendix S10** Multivariable analysis: social factors associated with zoster disease incidence restricted to patients of white ethnicity (complete case analysis).

**Appendix S11** Sensitivity analysis: multivariable analysis including practice‐level Index of Multiple Deprivation.

**Appendix S12.** Sensitivity analysis: multivariable analysis including cohabitation.
