# Supplementary material for: Inequalities in zoster disease burden: a population‐based cohort study to identify social determinants using linked data from the U.K. Clinical Practice Research Datalink
Source: Br J Dermatol. 2018 Apr 19;178(6):1324–30. doi: 10.1111/bjd.16399 (PMC6033149; doi:10.1111/bjd.16399)
Supplement: Supplementary file 1 — Table S1 Baseline characteristics of the study cohort (N = 862 470, outcome n = 37 014). [file BJD-178-1324-s001.docx]

Appendix S1 Code list: zoster

Zoster Clinical Practice Research Datalink (CPRD)

| Medical code | Read term |
| --- | --- |
| 390 | Herpes zoster |
| 516 | Shingles |
| 7331 | Ramsey Hunt Syndrome |
| 8936 | Ophthalmic herpes zoster infection |
| 14718 | Herpes zoster with ophthalmic complication |
| 14793 | Herpes zoster otitis externa |
| 18918 | Herpes zoster ophthalmicus |
| 21069 | Herpes zoster with unspecified complication |
| 21471 | Herpes zoster NOS |
| 25320 | Herpes zoster with dermatitis of eyelid |
| 27403 | Geniculate herpes zoster |
| 27546 | Herpes zoster with keratoconjunctivitis |
| 31681 | Herpes zoster - otitis externa |
| 33810 | Herpes zoster with other ophthalmic complication |
| 38531 | Herpes zoster with other specified complication NOS |
| 39692 | Polyneuropathy in herpes zoster |
| 43235 | Herpes zoster with other specified complication |
| 44944 | Herpes zoster with meningitis |
| 47375 | Zoster encephalitis |
| 50537 | Herpes zoster with other CNS complications |
| 51692 | Encephalitis due to herpes zoster |
| 52126 | Herpes zoster with other central nervous system complication |
| 52319 | Disseminated zoster |
| 55940 | Herpes zoster iridocyclitis |
| 57895 | Herpes zoster meningitis |
| 62558 | Infective otitis externa due to herpes zoster |
| 63739 | Herpes zoster with other CNS complication NOS |
| 69405 | Herpes zoster encephalitis |
| 70197 | [X]Zoster without complications |
| 71464 | Meningitis due to herpes zoster virus |
| 105157 | Hutchinson's sign - herpes zoster involving nose tip |

History of zoster codes CPRD

| Medical code | Read term |
| --- | --- |
| 1598 | Post-herpetic neuralgia |
| 7584 | Post-herpetic trigeminal neuralgia |
| 10223 | Postherpetic neuralgia |
| 17180 | Postzoster neuralgia |
| 31709 | Postherpetic polyneuropathy |
| 11498 | Postherpetic trigeminal neuralgia |

Zoster Hospital Episode Statistics (HES)

| ICD code | ICD description |
| --- | --- |
| B02 | Herpes zoster |
| B02.0 | Zoster encephalitis |
| B02.1 | Zoster meningitus |
| B02.3 | Zoster ocular disease |
| B02.7 | Disseminated zoster |
| B02.8 | Zoster with other complications |
| B02.9 | Zoster without complications |

History of zoster codes HES

| ICD code | ICD description |
| --- | --- |
| G53.0 | Postherpectic neuralgia |
| B02.2 | Zoster with other nervous system involvement |
