# Supplementary material for: Inequalities in zoster disease burden: a population‐based cohort study to identify social determinants using linked data from the U.K. Clinical Practice Research Datalink
Source: Br J Dermatol. 2018 Apr 19;178(6):1324–30. doi: 10.1111/bjd.16399 (PMC6033149; doi:10.1111/bjd.16399)
Supplement: Supplementary file 2 — Table S2 Multivariable analysis: social factors associated with zoster disease incidence (complete case analysis; individuals with missing data for ethnicity and sex excluded) (N = 711 590, outcome n = 32 459). [file BJD-178-1324-s002.docx]

Appendix S2 Conceptual hierarchical framework for the association of social factors with zoster disease burden

**Level 4**: Co-morbidities^

**Level 2**: Deprivation

**Level 3**: Marital status, cohabitation, living alone , care home residence

**Level 1**: Ethnicity, immigration status, religion

**Level 5**: Immunosuppressive medications~

**Outcome: Zoster 1st episode**

**A priori confounders**

Age

Sex

Calendar period

^included rheumatoid arthritis, systemic lupus erythematosus, inflammatory bowel disease, diabetes mellitus, chronic kidney disease, chronic obstructive pulmonary disease or asthma, HIV infection, other cellular immune deficiency, leukemia, lymphoma, myeloma, other plasma cell dyscrasias, haematopoietic stem cell transplant & solid organ transplant ~included immune-suppressive doses of oral/injectable corticosteroids, other immune-suppressants drugs (e.g. azathioprine, biological therapy, methotrexate) and cancer chemo/radiotherapy
