# Supplementary material for: Inequalities in zoster disease burden: a population‐based cohort study to identify social determinants using linked data from the U.K. Clinical Practice Research Datalink
Source: Br J Dermatol. 2018 Apr 19;178(6):1324–30. doi: 10.1111/bjd.16399 (PMC6033149; doi:10.1111/bjd.16399)
Supplement: Supplementary file 3 — Appendix S1 Code list: zoster. [file BJD-178-1324-s003.docx]

Appendix S3 Code lists: social factors, co-morbidities and medications

**Social factors**

1. Codelist for Clinical Practice Research Datalink

ia) Marital status: Read codes

| **Medcode** | **Readcode** | **Readterm** |
| --- | --- | --- |
| 207 | 13M1.00 | Death of spouse |
| 333 | 13H4200 | Marital conflict |
| 723 | 13H4.12 | Marital stress |
| 838 | 13H3000 | Divorce proceedings |
| 954 | 13H4100 | Marital breakdown |
| 1328 | 6124.00 | Partner had vasectomy |
| 1349 | 13H4.00 | Marital problems |
| 1522 | 1334.00 | Divorced |
| 1540 | 13H4212 | Marital disharmony |
| 1580 | 1332.11 | Remarried |
| 2093 | 13H2.00 | Separation |
| 2159 | 13H3100 | Divorce proceedings pending |
| 3111 | 13HP100 | Girlfriend relationship problem |
| 3321 | SN56300 | Battered wife |
| 3394 | 13W9.00 | Single parent family |
| 3483 | 13HD.00 | Violent spouse |
| 3551 | 13H4211 | Marital discord |
| 3719 | 13L6.00 | Spouse unwell |
| 3988 | 1332.00 | Married |
| 4204 | 1333.00 | Separated |
| 4312 | 1335.00 | Widowed |
| 4531 | 13HP000 | Boyfriend relationship problem |
| 4565 | 6741.00 | Marital counselling |
| 4925 | 1333.13 | Wife left home |
| 5055 | 1333.12 | Husband left home |
| 6056 | 13H3.00 | Divorce |
| 6104 | 13HP.00 | Relationship problems |
| 7419 | 1332.12 | Newly wed |
| 7869 | 13H1.00 | Marriage |
| 8470 | 13IL100 | Wife pregnant |
| 9112 | 13HX.00 | New relationship |
| 9551 | ZV61100 | [V]Marital problems |
| 9612 | 1311.00 | Housewife |
| 9910 | 13H3.11 | Divorce problems |
| 10330 | 13L3.11 | Alcoholic spouse |
| 11103 | 1331.11 | Single - unmarried |
| 11251 | 13MG.00 | Death of wife |
| 12076 | ZU14111 | Husband died |
| 12325 | ZU14100 | Death of husband |
| 13001 | 6123.00 | No partner at present |
| 15020 | 13HG.11 | Spouse left home |
| 15115 | 13HH.15 | Looks after chronically sick spouse |
| 15313 | 13H4300 | Maladjustment to married life |
| 15404 | 13L3.13 | Husband alcoholic |
| 15527 | 133C.00 | Widower |
| 15777 | 13HV311 | Spouse committed infidelity |
| 15824 | 13L1.11 | Disabled spouse |
| 15950 | 13H5.12 | Spouse returned home |
| 16262 | 13H6.11 | Unmarried parent |
| 16315 | 133G.00 | Common-law husband |
| 16344 | 8C81.12 | Artificial insemin by husband |
| 16552 | 13HH.13 | Looks after chronically sick husband |
| 17538 | 13IL300 | Wife alive |
| 17802 | 13MH.00 | Husband died |
| 20079 | 13H6.00 | Single parent |
| 20149 | 13HH.16 | Looks after chronically sick wife |
| 20217 | 13HM.11 | Legal problem with separation |
| 20313 | 13HM.12 | Legal problem with divorce |
| 20536 | 13HV100 | Affair ended |
| 21346 | 8C92.00 | Spouse reassured |
| 21433 | 13HV313 | Husband committed adultery |
| 21860 | 13IL.00 | Health of spouse |
| 21925 | 13HV400 | Seven year itch - marital |
| 22336 | 13L6.11 | Has infirm partner |
| 22909 | 13JK.13 | Partnership problems |
| 22934 | 6124.11 | Partner sterilised |
| 23385 | 13FD100 | Spouse cannot care for patient |
| 23409 | 13HV000 | Affair started |
| 23445 | 13HV200 | Affair unsatisfactory |
| 23508 | SN56400 | Battered husband |
| 23514 | 13HT114 | Wife unable to cope |
| 23858 | ZV61011 | [V]Divorce |
| 23974 | 13IL200 | Wife well |
| 24055 | 13HG.00 | Broken with partner |
| 24769 | 13I7100 | Husband in prison |
| 25097 | 13MI.00 | Death of husband |
| 25149 | 13W9011 | Single parent family - mother |
| 25452 | 13D2.00 | Homeless single person |
| 25503 | 1336.00 | Cohabiting |
| 27385 | 1333.11 | Separated from cohabitee |
| 27434 | 13HV011 | Lover taken |
| 27572 | 13HE.00 | Engaged |
| 28440 | 13MF.00 | Death of partner |
| 28484 | U3N0.00 | [X]Other maltreatment syndromes, by spouse or partner |
| 29543 | 13H5.00 | Marital reconciliation |
| 29544 | 13H4.11 | Marital trouble |
| 30597 | ZV26500 | [V]Artificial insemination from husband |
| 30950 | 13H4213 | Row with wife |
| 31495 | 8H7I.00 | Refer to partner |
| 31678 | 13EF.00 | Divorced couple sharing house |
| 32451 | 67M..00 | Informing partner |
| 32984 | 1F81.00 | Spouse cooks food |
| 33000 | 13Q..12 | Widows pensions |
| 33001 | 131..11 | Occupation of husband |
| 33153 | 1312.00 | House husband |
| 33188 | 13ID.00 | Partner unemployed |
| 34771 | 13HV.00 | Extra-marital problems |
| 36077 | 131..00 | Occupation of spouse |
| 36333 | ZLB4.00 | Seen by marriage guidance counsellor |
| 36947 | 13L7.00 | Partner dying |
| 37113 | U3M0.00 | [X]Neglect and abandonment, by spouse or partner |
| 37265 | 1331.00 | Single |
| 37551 | 13HVZ00 | Extra-marital problems NOS |
| 38325 | 13H5.11 | Cohabitee returned |
| 39292 | 131..12 | Occupation of wife |
| 39474 | 9NA8.00 | Cohabitee made appointment |
| 39651 | 13HV300 | Spouse committed adultery |
| 39879 | 13HV314 | Oil rig wives syndrome |
| 40493 | 13HF.00 | Broken engagement |
| 40866 | 13Q..00 | Widows benefits |
| 41203 | 13L1200 | Spouse is handicapped |
| 42321 | 13H4311 | Spouse unsympathetic |
| 42386 | 133H.00 | Common-law wife |
| 42390 | 13I7300 | Boyfriend arrested |
| 42398 | 13I7200 | Spouse arrested |
| 42400 | 13HV312 | Wife committed adultery |
| 42402 | 13WE.00 | Spouse works away from home |
| 42428 | 1333.14 | Cohabitee left home |
| 45005 | 1AZ5.00 | Fertility problems in partner |
| 45010 | 13Q3.00 | Widows pension |
| 47411 | 13I2.00 | Partner stops work |
| 49666 | 1AZ4.00 | Low sperm count in partner |
| 50149 | 13W7000 | Crime against spouse |
| 50485 | 13HY.00 | First relationship |
| 54096 | 131Z.00 | Occupation of spouse NOS |
| 54816 | 13H4312 | Spouse inattentive |
| 56178 | 13HV012 | Mistress taken |
| 59817 | 9d31.00 | Husband |
| 59829 | 9d32.00 | Wife |
| 60723 | 918j.00 | Partner is informal carer |
| 60821 | 13QZ.00 | Widows benefits NOS |
| 61291 | 1A85.00 | Breast lump detected by partner |
| 61509 | 13Q..11 | Widows allowances |
| 63118 | 1276.11 | Spouse haemophiliac |
| 68095 | 13I7400 | Girlfriend arrested |
| 88373 | 133S.00 | Married/civil partner |
| 91652 | 7E0A300 | Intrauterine insemination superovulation partner sperm |
| 94044 | 133V.00 | Widowed/surviving civil partner |
| 94917 | 13Ir.00 | Partner pregnant |
| 95101 | 9d30.00 | Spouse |
| 96856 | 133T.00 | Divorced/person whose civil partnership has been dissolved |
| 97076 | 6127.00 | Partner had tubal ligation |
| 98130 | 9d33.00 | Cohabitee |
| 98610 | 13Q5.00 | War widows pension |
| 98818 | 13Q1.00 | Widows allowance |
| 99328 | 13I1.00 | Partner begins work |
| 100785 | 68b9.00 | Anten screen, partner tested and no genetic risk identified |
| 101900 | 13I4.00 | Partner works after retirement |
| 102413 | 133b.00 | Partner in relationship |
| 104879 | 133e.00 | Common law partnership |
| 104936 | U3P0.00 | [X]Maltreatment, by spouse or partner |
| 109323 | 13I3.00 | Partner retires |

ib) Entity type for marital status: Entity Type: 98

iia) Cohabitation Read codes

| **Medcode** | **Readcode** | **Readterm** |
| --- | --- | --- |
| 333 | 13H4200 | Marital conflict |
| 723 | 13H4.12 | Marital stress |
| 954 | 13H4100 | Marital breakdown |
| 1349 | 13H4.00 | Marital problems |
| 1540 | 13H4212 | Marital disharmony |
| 1580 | 1332.11 | Remarried |
| 3321 | SN56300 | Battered wife |
| 3394 | 13W9.00 | Single parent family |
| 3483 | 13HD.00 | Violent spouse |
| 3551 | 13H4211 | Marital discord |
| 3719 | 13L6.00 | Spouse unwell |
| 3988 | 1332.00 | Married |
| 4565 | 6741.00 | Marital counselling |
| 4925 | 1333.13 | Wife left home |
| 5055 | 1333.12 | Husband left home |
| 7419 | 1332.12 | Newly wed |
| 7869 | 13H1.00 | Marriage |
| 8470 | 13IL100 | Wife pregnant |
| 9551 | ZV61100 | [V]Marital problems |
| 9612 | 1311.00 | Housewife |
| 10330 | 13L3.11 | Alcoholic spouse |
| 11103 | 1331.11 | Single - unmarried |
| 13001 | 6123.00 | No partner at present |
| 15115 | 13HH.15 | Looks after chronically sick spouse |
| 15313 | 13H4300 | Maladjustment to married life |
| 15404 | 13L3.13 | Husband alcoholic |
| 15777 | 13HV311 | Spouse committed infidelity |
| 15824 | 13L1.11 | Disabled spouse |
| 15950 | 13H5.12 | Spouse returned home |
| 16262 | 13H6.11 | Unmarried parent |
| 16315 | 133G.00 | Common-law husband |
| 16344 | 8C81.12 | Artificial insemin by husband |
| 16552 | 13HH.13 | Looks after chronically sick husband |
| 17538 | 13IL300 | Wife alive |
| 20079 | 13H6.00 | Single parent |
| 20149 | 13HH.16 | Looks after chronically sick wife |
| 21346 | 8C92.00 | Spouse reassured |
| 21433 | 13HV313 | Husband committed adultery |
| 21860 | 13IL.00 | Health of spouse |
| 21925 | 13HV400 | Seven year itch - marital |
| 23385 | 13FD100 | Spouse cannot care for patient |
| 23508 | SN56400 | Battered husband |
| 23514 | 13HT114 | Wife unable to cope |
| 23974 | 13IL200 | Wife well |
| 24769 | 13I7100 | Husband in prison |
| 25149 | 13W9011 | Single parent family - mother |
| 25452 | 13D2.00 | Homeless single person |
| 25503 | 1336.00 | Cohabiting |
| 27385 | 1333.11 | Separated from cohabitee |
| 29543 | 13H5.00 | Marital reconciliation |
| 29544 | 13H4.11 | Marital trouble |
| 30597 | ZV26500 | [V]Artificial insemination from husband |
| 30950 | 13H4213 | Row with wife |
| 31678 | 13EF.00 | Divorced couple sharing house |
| 32984 | 1F81.00 | Spouse cooks food |
| 33001 | 131..11 | Occupation of husband |
| 33153 | 1312.00 | House husband |
| 34771 | 13HV.00 | Extra-marital problems |
| 36077 | 131..00 | Occupation of spouse |
| 36333 | ZLB4.00 | Seen by marriage guidance counsellor |
| 37113 | U3M0.00 | [X]Neglect and abandonment, by spouse or partner |
| 37265 | 1331.00 | Single |
| 37551 | 13HVZ00 | Extra-marital problems NOS |
| 38325 | 13H5.11 | Cohabitee returned |
| 39292 | 131..12 | Occupation of wife |
| 39474 | 9NA8.00 | Cohabitee made appointment |
| 39651 | 13HV300 | Spouse committed adultery |
| 39879 | 13HV314 | Oil rig wives syndrome |
| 41203 | 13L1200 | Spouse is handicapped |
| 42321 | 13H4311 | Spouse unsympathetic |
| 42386 | 133H.00 | Common-law wife |
| 42398 | 13I7200 | Spouse arrested |
| 42400 | 13HV312 | Wife committed adultery |
| 42402 | 13WE.00 | Spouse works away from home |
| 42428 | 1333.14 | Cohabitee left home |
| 50149 | 13W7000 | Crime against spouse |
| 54096 | 131Z.00 | Occupation of spouse NOS |
| 54816 | 13H4312 | Spouse inattentive |
| 59817 | 9d31.00 | Husband |
| 59829 | 9d32.00 | Wife |
| 60723 | 918j.00 | Partner is informal carer |
| 63118 | 1276.11 | Spouse haemophiliac |
| 88373 | 133S.00 | Married/civil partner |
| 95101 | 9d30.00 | Spouse |
| 98130 | 9d33.00 | Cohabitee |
| 104879 | 133e.00 | Common law partnership |

iib) Entity type for cohabitation: Entity Type: 98

iiia) Living arrangement: living alone Read codes

| **Medcode** | **Readcode** | **Readterm** |
| --- | --- | --- |
| 464 | 13HT115 | Domestic problems |
| 1123 | 13HQ.00 | In prison |
| 1650 | 13HT100 | Stress at home |
| 2562 | 13D..11 | Homeless |
| 2955 | 1B1K.12 | Lives alone |
| 11504 | ZU33200 | Lives with daughter |
| 12798 | ZU33600 | Lives with father |
| 12807 | ZU33100 | Lives with children |
| 13355 | 13F1.00 | Independant housing, not alone |
| 13356 | 13F2.00 | Lives alone - help available |
| 13357 | 13F3.00 | Lives alone -no help available |
| 13358 | 13FH.00 | Lives with relatives |
| 15416 | 13F7300 | Lives in a childrens home |
| 15691 | 13F3100 | Lives alone needs housekeeper |
| 15700 | 13JS.00 | Works away from home |
| 15840 | 13F7100 | Lives in a welfare home |
| 17279 | 13FH000 | Elderly relative lives with family |
| 19610 | 13FJ.00 | Independent housing, lives alone |
| 20155 | 13HT113 | Home unsettled |
| 21405 | 13F7400 | Admitted to a children's home |
| 22249 | ZU3..11 | Lives with |
| 22336 | 13L6.11 | Has infirm partner |
| 22503 | ZU33300 | Lives with son |
| 23575 | 13FD.00 | No carers, though not alone |
| 25167 | ZU31.00 | Lives alone |
| 25452 | 13D2.00 | Homeless single person |
| 25715 | 8He1.00 | Referral to intermediate care - community rehabilitation |
| 26177 | 8He0.00 | Referral to intermediate care - hospital at home |
| 28448 | ZU33500 | Lives with mother |
| 30200 | ZV60611 | [V]Boarding school resident |
| 30965 | 13E6.00 | Overcrowded in house |
| 31385 | 13F8100 | Long stay hospital inpatient |
| 31678 | 13EF.00 | Divorced couple sharing house |
| 32753 | 13D3.11 | Tramp |
| 32774 | 13D1.00 | Homeless family |
| 32882 | 8He..00 | Referral to intermediate care |
| 33006 | 1311.11 | Homemaker |
| 33994 | ZW63200 | Staying with carer |
| 34506 | 13FL.00 | Living rough |
| 36418 | ZV60300 | [V]Person living alone |
| 36730 | Z37C.00 | Provision of special residential school |
| 36809 | ZU33.00 | Lives with family |
| 36947 | 13L7.00 | Partner dying |
| 36968 | ZU33400 | Lives with parents |
| 40822 | ZU33700 | Lives with grandparents |
| 41388 | 13D5.00 | Vagrant |
| 42533 | ZU26100 | Number of dependants in household |
| 42654 | ZU35.00 | Lives with companion |
| 43393 | ZU33800 | Lives with grandfather |
| 43911 | ZU33900 | Lives with grandmother |
| 47577 | ZW63100 | Living with carer |
| 47591 | 13FS.00 | Long stay hospital inpatient |
| 49138 | ZV63212 | [V]Delayed discharge - nursing home vacancy awaited |
| 50111 | 13HH.11 | Cares for mentally handicapped dependent |
| 50206 | 8O0A.00 | Provision of special residential school |
| 50994 | 13HH.18 | Looks after physically handicapped dependent |
| 51193 | ZU32.00 | Lives with friends |
| 52682 | 6992.00 | Prison medical examination |
| 53343 | ZU3..12 | LW - Lives with |
| 55276 | ZU37.00 | Lives in a community |
| 57438 | ZU32100 | Lives with friend |
| 59330 | T776.00 | Place of occurrence of accident or poisoning, prison |
| 59523 | 13D3.12 | Vagabond |
| 61385 | 9b1C.00 | Hospice - independent |
| 66549 | 13EA.00 | Multiple occupancy |
| 67112 | 9k6..00 | Homeless - enhanced services administration |
| 67187 | 13FM.00 | Sleeping in night shelter |
| 68005 | 13FV.00 | Lives in a welfare home |
| 70021 | ZU36.00 | Lives as companion |
| 71339 | 0A82.00 | Companion |
| 71663 | ZU37200 | Lives in boarding school |
| 73177 | ZLG6100 | Discharge to long stay hospital |
| 86390 | 13FY.00 | Lives in a children's unit |
| 90547 | ZU34.00 | Lives with lodger |
| 94886 | 13Il.00 | Subject to interim supervision order under Children Act 1989 |
| 95555 | ZU37300 | Lives in a commune |
| 95880 | 13It.00 | Lives with grandmother |
| 96605 | 9k60.00 | Homeless - enhanced service completed |
| 97138 | 13Is.00 | Lives with grandfather |
| 99907 | ZV60011 | [V]Hobo |
| 101400 | 13Zr.00 | Lives with immunocompromised person |
| 101582 | 9b0t.00 | Children's home visit note |
| 103510 | ZV60014 | [V]Tramp |
| 103553 | ZU37100 | Lives in a school community |
| 104962 | 13D8.00 | Length of time homeless |
| 106972 | 13IZ000 | Lives with adoptive parents |
| 107393 | 9Ngr.00 | Under care of homeless advocacy service |
| 107733 | 13IZ200 | Lives with biological parents |
| 107809 | 918F200 | Lives with carer |
| 109673 | 13IZ100 | Lives with biological parent and step parent |

iiib) Entity type for living alone Entity Type: 132

iva) Residence Read codes

| **Medcode** | **Readcode** | **Readterm** |
| --- | --- | --- |
| 1123 | 13HQ.00 | In prison |
| 2562 | 13D..11 | Homeless |
| 6855 | 9491.00 | Patient died at home |
| 6859 | 9N1F.00 | Seen in warden sup home |
| 6991 | 9493.00 | Patient died in nursing home |
| 7101 | 9N1F.12 | Seen in old people's home |
| 7653 | 9N1G.00 | Seen in nursing home |
| 10120 | 9N1C.00 | Seen in own home |
| 10993 | ZLG4.00 | Discharge to nursing home |
| 11419 | 13F7200 | Lives in an old peoples home |
| 11504 | ZU33200 | Lives with daughter |
| 11949 | 13F4.00 | Warden attended |
| 12798 | ZU33600 | Lives with father |
| 12807 | ZU33100 | Lives with children |
| 13355 | 13F1.00 | Independant housing, not alone |
| 13357 | 13F3.00 | Lives alone -no help available |
| 13358 | 13FH.00 | Lives with relatives |
| 13359 | 13F6100 | Lives in a nursing home |
| 13360 | 13F6.00 | Nursing/other home |
| 13361 | 13F4.11 | Lives in warden controlled accommodation |
| 13562 | ZV70317 | [V]Old age home admission medical |
| 15691 | 13F3100 | Lives alone needs housekeeper |
| 15700 | 13JS.00 | Works away from home |
| 15840 | 13F7100 | Lives in a welfare home |
| 17279 | 13FH000 | Elderly relative lives with family |
| 18291 | 13EC.00 | House in poor repair |
| 19610 | 13FJ.00 | Independent housing, lives alone |
| 21280 | 13F5200 | Resident in part III accomodation |
| 22503 | ZU33300 | Lives with son |
| 24494 | ZV60600 | [V]Institution resident |
| 24756 | 13KD.00 | Owner-occupier |
| 24815 | 13K8.00 | House rented from council |
| 24816 | Z177C00 | Residential care |
| 24828 | Z177F00 | Nursing home care |
| 24910 | 13KA.00 | House rented-private landlord |
| 24956 | 13FK.00 | Lives in a residential home |
| 25143 | 13K6.00 | Houseowner - no mortgage |
| 25452 | 13D2.00 | Homeless single person |
| 26177 | 8He0.00 | Referral to intermediate care - hospital at home |
| 26720 | 13FB.00 | Living in lodgings |
| 26812 | 9494.00 | Patient died in resid.inst.NOS |
| 27360 | 13F5100 | Part III accomodation arranged |
| 27425 | 13F5.00 | Part III accommodation |
| 27936 | 8HE6.00 | Delayed discharge to nursing home |
| 27968 | 13F7.00 | Residential institution |
| 28448 | ZU33500 | Lives with mother |
| 28773 | ZV60700 | [V]Sheltered housing |
| 30200 | ZV60611 | [V]Boarding school resident |
| 30807 | 13F4000 | Resident in sheltered accommodation |
| 31385 | 13F8100 | Long stay hospital inpatient |
| 31678 | 13EF.00 | Divorced couple sharing house |
| 31951 | 13F9.00 | Living in hostel |
| 32448 | 13EH100 | Harrassment by landlord |
| 32753 | 13D3.11 | Tramp |
| 32774 | 13D1.00 | Homeless family |
| 33006 | 1311.11 | Homemaker |
| 33153 | 1312.00 | House husband |
| 33994 | ZW63200 | Staying with carer |
| 34506 | 13FL.00 | Living rough |
| 34794 | 13F9.11 | Living in sheltered accomodatn |
| 35040 | ZLG5.00 | Discharge to sheltered housing |
| 35172 | 9N1E.00 | Seen in warden sup flat |
| 35187 | 9N1D.00 | Seen in warden sup house |
| 35279 | 9N1H.00 | Seen in Elderly Mentaly Infirm home |
| 35716 | 13FA.00 | Living in B&B accommodation |
| 36096 | 13F5.11 | Part 3 accomodation |
| 36730 | Z37C.00 | Provision of special residential school |
| 36809 | ZU33.00 | Lives with family |
| 36905 | ZLG5100 | Discharge to warden controlled accommodation |
| 36968 | ZU33400 | Lives with parents |
| 37829 | U195100 | [X]Victim of volcanic eruption occurrn in resident instit'n |
| 39311 | 9492.00 | Patient died in part 3 accom. |
| 39685 | 13K7.00 | Houseowner with mortgage |
| 40822 | ZU33700 | Lives with grandparents |
| 41188 | 13FC.11 | Lives in a bedsit |
| 41388 | 13D5.00 | Vagrant |
| 41986 | 699Z.00 | Exam. for institution NOS |
| 42191 | ZLG3.00 | Discharge to residential home |
| 42533 | ZU26100 | Number of dependants in household |
| 42654 | ZU35.00 | Lives with companion |
| 43057 | 13FG.00 | Squatter |
| 43393 | ZU33800 | Lives with grandfather |
| 43709 | ZV70H00 | [V]Examination for admission to residential institutions |
| 43911 | ZU33900 | Lives with grandmother |
| 43915 | ZLG4100 | Discharge to private nursing home |
| 44053 | 699..00 | Examination for institution |
| 45650 | T704.00 | Place of occurrence of accident/poisoning, residential house |
| 46222 | T774.00 | Place of occurrence of accident/poisoning, old people's home |
| 46303 | U10z100 | [X]Unspecified fall, occurrence in residential institution |
| 46588 | 13K9.00 | House rented from housing ass. |
| 46642 | 9b79.00 | Other residential care homes managed by local authority |
| 47577 | ZW63100 | Living with carer |
| 47591 | 13FS.00 | Long stay hospital inpatient |
| 47609 | T77..00 | Place of accident or poisoning, residential institution |
| 47685 | ZV6y200 | [V]Other boarder in health-care facility |
| 48549 | ZLG3100 | Discharge to private residential home |
| 48733 | U198100 | [X]Victim of flood, occurrence in residential institution |
| 48805 | U120100 | [X]Hit struck kick twist bit/scratch anoth pers resid instit |
| 48932 | U125100 | [X]Bitten/struck by oth mammal occurrn in resident instit'n |
| 49138 | ZV63212 | [V]Delayed discharge - nursing home vacancy awaited |
| 49210 | U101100 | [X]Fall same level from slip trip + stumb occ resid instit |
| 49681 | 13FX.00 | Lives in care home |
| 50206 | 8O0A.00 | Provision of special residential school |
| 50792 | 9N1F.11 | Seen in Part 3 accomodation |
| 51193 | ZU32.00 | Lives with friends |
| 51495 | 13FC.00 | Living in bedsitter |
| 51851 | U104100 | [X]Fall whle carried/supported oth persons occ resid instit |
| 52249 | 13FQ.00 | Lives on council site |
| 52466 | U10A100 | [X]Fall on + from stair + step occurrnce resident instit'n |
| 52682 | 6992.00 | Prison medical examination |
| 52881 | U291.00 | [X]Intent self harm by sharp object occ resident instit'n |
| 53140 | Z177D00 | Local authority residential care |
| 53600 | U12A100 | [X]Contct wth plant thorn+spine+sharp leave occ resid instit |
| 54260 | U3F1.00 | [X]Assault by blunt object occurrn in resident institution |
| 54735 | 13EC.11 | Slum housing |
| 54948 | ZLG5200 | Discharge to part III accommodation |
| 55276 | ZU37.00 | Lives in a community |
| 56326 | U3K1.00 | [X]Assault by bodily force occurrn in residential institut'n |
| 56969 | T77z.00 | Accident/poisoning occurred in residential institution NOS |
| 57438 | ZU32100 | Lives with friend |
| 59330 | T776.00 | Place of occurrence of accident or poisoning, prison |
| 59523 | 13D3.12 | Vagabond |
| 59548 | 13FT.00 | Lives in an old peoples home |
| 59653 | 6991.00 | Geriatric home admission exam. |
| 60404 | U221.00 | [X]Intent self harm by drowning/submersn occ resid instit'n |
| 60684 | U2A1.00 | [X]Intent self harm by blunt object occ resident instit'n |
| 61385 | 9b1C.00 | Hospice - independent |
| 62522 | U3z1.00 | [X]Assault by unspecified means occurrn resident institut'n |
| 64410 | U211.00 | [X]Intent self harm by hangng strangult/suffoct resid instit |
| 65445 | 13FP.00 | Lives on private site |
| 66122 | 13F5111 | Part 3 accomodation arranged |
| 66599 | U152100 | [X]Exposure to unspecif electric current occ resid instit'n |
| 66656 | U128100 | [X]Bitten/struck by crocodil/alligatr occ in resid instit'n |
| 66922 | U108100 | [X]Fall involv other furniture occurrn resident institut'n |
| 67112 | 9k6..00 | Homeless - enhanced services administration |
| 67187 | 13FM.00 | Sleeping in night shelter |
| 67586 | U241.00 | [X]Int self harm rifl s'gun/lrg frarm disch occ resid instit |
| 67903 | U105100 | [X]Fall involvng wheelchair occurrence residential instit'n |
| 67930 | 13FG.11 | Illegal tennant |
| 68005 | 13FV.00 | Lives in a welfare home |
| 69028 | ZLG3200 | Discharge to part III residential home |
| 69762 | U106100 | [X]Fall involving bed occurrence in residential institution |
| 70021 | ZU36.00 | Lives as companion |
| 70848 | 13FW.00 | Living in temporary housing |
| 71663 | ZU37200 | Lives in boarding school |
| 72474 | U10J100 | [X]Other fall on same level, occurrnce in resident instit'n |
| 72716 | U143100 | [X]Inhalation of gastric contents occurrn resident instit'n |
| 72838 | 13FR.00 | Lives on unofficial site |
| 73083 | 9b0Y.00 | Nursing home visit note |
| 73101 | U3L1.00 | [X]Sexual assault by bodily force occurrn resident instit'n |
| 73177 | ZLG6100 | Discharge to long stay hospital |
| 73321 | 9b1P.00 | Nursing home |
| 87882 | U2y1.00 | [X]Intent self harm by oth specif means occ resid instit'n |
| 90547 | ZU34.00 | Lives with lodger |
| 91941 | U11Q100 | [X]Foreign body enter into/thr eye/natrl orif, resid instit |
| 92265 | U197100 | [X]Victim of cataclysmic storm occurrn in resident instit'n |
| 92315 | U3y1.00 | [X]Assault by oth specif means occurrn resident institution |
| 93837 | U2C1.00 | [X]Int self harm jump/lying befr mov obje occ resid instit'n |
| 93865 | U11H100 | [X]Explosn+ruptur of pressr tyre pipe/hose occ resid instit |
| 93998 | 9b0i.00 | Residential home visit note |
| 94070 | 8O24.00 | Provision of continuing care in nursing home |
| 95555 | ZU37300 | Lives in a commune |
| 95661 | U116100 | [X]Contact wth knife sword/dagger occurrn in resid instit'n |
| 95880 | 13It.00 | Lives with grandmother |
| 96605 | 9k60.00 | Homeless - enhanced service completed |
| 96663 | U112100 | [X]Strikng against/struck by other object occ in resid inst |
| 97138 | 13Is.00 | Lives with grandfather |
| 97757 | 13D7.00 | Sofa surfer - person of no fixed abode |
| 99091 | U122100 | [X]Crush push/step on by crowd/humn stampede occ resid inst |
| 99110 | U10F100 | [X]Fall from cliff, occurrence in residential institution |
| 99120 | U193100 | [X]Victim of lightning, occurrn in residential institution |
| 99148 | 9b7A.00 | Other residential care home man voluntary/private agents |
| 99453 | U156100 | [X]Expos unspecif type of radiatn occurrn resident instit'n |
| 99598 | U114100 | [X]Contact with lifting+transmissn dev NEC occ resid instit |
| 99907 | ZV60011 | [V]Hobo |
| 100246 | ZVu5700 | [X]Other boarder in health care facility |
| 100389 | U321.00 | [X]Assault by pesticides occurrn in residential institution |
| 100710 | U10D100 | [X]Fall from out of/thro buildng/struct occ resid instit'n |
| 101003 | 9NFR.00 | Home visit request by residential institution |
| 101078 | 949D.00 | Patient died in care home |
| 101400 | 13Zr.00 | Lives with immunocompromised person |
| 102230 | M270100 | Nursing home acquired pressure ulcer |
| 102493 | 8Ht..00 | Admission to nursing home |
| 103138 | U3E1.00 | [X]Assault by sharp object occurrn in resident institution |
| 103285 | 9b70.00 | Client's or patient's home |
| 103461 | 133c.00 | Hospital at home patient |
| 103510 | ZV60014 | [V]Tramp |
| 103553 | ZU37100 | Lives in a school community |
| 104962 | 13D8.00 | Length of time homeless |
| 105063 | U127100 | [X]Bittn/stung by nven insct+oth nven arthrop occ resid inst |
| 106027 | 13KD.11 | Lives in own home |
| 106285 | U126100 | [X]Contact wth marine animal occurrn in resident institut'n |
| 106972 | 13IZ000 | Lives with adoptive parents |
| 107072 | U144100 | [X]Inhal+ingest food caus obst resp tract occ resid instit'n |
| 107393 | 9Ngr.00 | Under care of homeless advocacy service |
| 107733 | 13IZ200 | Lives with biological parents |
| 107757 | 9NFW.00 | Care home visit |
| 107809 | 918F200 | Lives with carer |
| 107927 | U1B4100 | [X]Lack of water, occurrence in residential institution |
| 108702 | 13D6.00 | Lives in squat |
| 109437 | TD17200 | Accident due to fall from burning convalescent home |
| 109673 | 13IZ100 | Lives with biological parent and step parent |

ivb) Entity type for residence Entity Type: 132

v) Religion

| **Medcode** | **Readcode** | **Readterm** |
| --- | --- | --- |
| 2053 | 1357 | Jehovah's Witness |
| 12477 | 1355 | Jewish |
| 12622 | 135Y.00 | Spiritualist |
| 12685 | 1358 | Hindu |
| 19559 | 135D.00 | Religion, none |
| 24263 | 1351 | Church of England |
| 24268 | 1352 | Roman Catholic |
| 24273 | 135S.00 | Buddhist |
| 24297 | 135B.00 | Sikh |
| 24341 | 1359 | Islam |
| 24669 | 135A.00 | Christian |
| 25594 | 135F.00 | Baptist |
| 25996 | 1359.11 | Muslim |
| 29954 | 1356 | Christian Scientist |
| 31044 | 135J.00 | Church of Scotland |
| 31585 | 135N.00 | Plymouth Brethren |
| 39701 | 135M.00 | Society of Friends |
| 39751 | 135G.00 | Methodist |
| 39839 | 1354 | Atheist |
| 46063 | 9iF6.00 | Jewish - ethnic category 2001 Census |
| 47091 | 9iF7.00 | Muslim - ethnic category 2001 Census |
| 47329 | 135W.00 | Salvation Army |
| 47959 | 135K.00 | Pentecostal |
| 47961 | 135L.00 | Evangelical |
| 47962 | 135C.00 | Mixed religion |
| 47968 | 135P.00 | Agnostic |
| 47971 | 1351.11 | Anglican |
| 47972 | 135I.00 | Presbyterian |
| 47998 | 135d.00 | Orthodox Christian |
| 47999 | 135V.00 | Jainism |
| 48000 | 135H.00 | United Reform Church |
| 49658 | 9iF8.00 | Sikh - ethnic category 2001 Census |
| 50229 | 135T.00 | Rastafarian |
| 52201 | 135O.00 | Christadelphian |
| 56127 | 9iF5.00 | Hindu - ethnic category 2001 Census |
| 57757 | 1353 | Nonconformist |
| 58665 | 1359100 | Sunni muslim |
| 63872 | 9iF4.00 | Buddhist - ethnic category 2001 Census |
| 64041 | 1359000 | Shiite muslim |
| 64056 | 135b.00 | Pagan |
| 64057 | 135c.00 | Mormon |
| 64058 | 135a.00 | Moravian religion |
| 92227 | 135X.00 | Eastern Catholic |
| 99738 | 13zA.00 | Protestant |
| 100125 | 13zp.00 | Church of England, follower of religion |
| 100433 | 13z2.00 | Armenian Orthodox |
| 100435 | 13yH.00 | Follower of Goddess tradition |
| 100497 | 13yX.00 | Mennonite |
| 100522 | 13zJ.00 | Church of Scotland, follower of religion |
| 100526 | 13yc.00 | Seventh Day Adventist |
| 100536 | 13z6.00 | Scottish Episcopalian |
| 100713 | 13yQ.00 | Pure Land Buddhist |
| 100794 | 13zC.00 | French Protestant |
| 100867 | 135l.00 | African religion, follower of religion |
| 100951 | 135m.00 | Yoruba, follower of religion |
| 101115 | 13zG.00 | Lutheran |
| 101118 | 13yB.00 | Ancestral worship |
| 101144 | 135z.00 | New age practitioner |
| 101384 | 13zi.00 | Orthodox Jew |
| 101650 | 135i.00 | Baha'i |
| 101828 | 13z5.00 | Ukrainian Catholic |
| 101837 | 13yP.00 | Zen Buddhist |
| 101856 | 13z3.00 | Greek Orthodox |
| 101905 | 13y8.00 | Black magic |
| 102123 | 135x.00 | Native American religion, follower of religion |
| 102124 | 13z9.00 | Catholic: non Roman Catholic |
| 102238 | 13yi.00 | Judaic Christian |
| 102253 | 135v.00 | Radha Soami |
| 102459 | 13zE.00 | Follower of United Reformed Church |
| 102498 | 13yD.00 | Wiccan |
| 102646 | 135e.00 | Shinto |
| 102697 | 13yy.00 | Romanian Orthodox |
| 102902 | 13zF.00 | Quaker |
| 102956 | 13za.00 | Sanatana Dharma |
| 102991 | 13zd.00 | Shakti Hindu |
| 103060 | 135j.00 | Druze |
| 103225 | 135t.00 | Satanist |
| 103376 | 13y5.00 | Chondogyo |
| 103692 | 13ys.00 | Russian Orthodox |
| 103742 | 13yH.11 | Goddess |
| 103845 | 13y3.00 | Humanist |
| 103929 | 13zD.00 | Free Church of Scotland |
| 104111 | 13z1.00 | Bulgarian Orthodox |
| 104329 | 135v.11 | Sant Mat |
| 104723 | 13zH.00 | Congregationalist |
| 104737 | 13zl.00 | Ashkenazi Jew |
| 104893 | 13yl.00 | Christian Existentialist |
| 104965 | 13ze.00 | Smarta Hindu |
| 105120 | 135w.00 | Pantheist |
| 105407 | 13y4.00 | Deist |
| 105517 | 13yF.00 | Occultist |
| 105548 | 13y1.00 | Kabbalist |
| 105874 | 135q.00 | Taoist |
| 106119 | 135p.00 | Unitarian Universalist |
| 106797 | 13yt.00 | Ethiopian Orthodox Tewahedo |
| 107174 | 135Z.11 | Rastafarian |
| 107288 | 13yh.00 | Messianic Jew |
| 107636 | 13zP.00 | Reformed Christian |
| 107810 | 13yv.00 | Ukrainian Orthodox |
| 107871 | 13yG.00 | Heathen |
| 107962 | 13zh.00 | Reform Jew |
| 108011 | 135k.00 | Ahmadi |
| 108014 | 13ym.00 | Celtic Christian |
| 108015 | 13yj.00 | Christian Spiritualist |
| 108066 | 13yZ.00 | Free Church |
| 108225 | 13yC.00 | Zoroastrian |
| 108497 | 13zg.00 | Arya Samaj Hindu |
| 108571 | 13yn.00 | Celtic Orthodox Christian |
| 109080 | 13yN.00 | Mahayana Buddhist |
| 109590 | 13z8.00 | Church in Wales |

vi) Country of birth

| **Medcode** | **Readcode** | **Readterm** |
| --- | --- | --- |
| 4114 | 13ZC.00 | Immigrant |
| 8929 | ZV70314 | [V]Immigration medical |
| 9144 | 13Z6800 | Speaks English poorly |
| 9292 | 133L.00 | Immigrant |
| 9627 | 13ZN.00 | Asylum seeker |
| 11552 | 13e..00 | Country of birth (Asian) |
| 12458 | 13gf.00 | Born in South Africa |
| 12713 | 13eG.00 | Born in Iraq |
| 22294 | 13lZ.00 | Main spoken language Turkish |
| 23523 | 13Z6000 | English as a second language |
| 24295 | 13Z6500 | Language Punjabi |
| 24296 | 13Z6300 | Language Hindi |
| 24403 | 13ZB.00 | Refugee |
| 24691 | 13Z6600 | Language Urdu |
| 24712 | 13Z6200 | Language Gujurati |
| 24741 | 13Z6100 | Language Bengali |
| 24881 | 13lC.00 | Main spoken language Polish |
| 25007 | 13eH.00 | Born in Israel |
| 25008 | 13go.00 | Born in Zimbabwe |
| 25092 | 13eY.00 | Born in Philippines |
| 25133 | 13gi.00 | Born in Tanzania |
| 25256 | 13dl.00 | Born in Yugoslavia |
| 25410 | 13b0.00 | Vietnamese language |
| 25423 | 13lS.00 | Main spoken language Albanian |
| 25472 | 13l2.00 | Main spoken language Cantonese |
| 25609 | 13lx.00 | Main spoken language Thai |
| 25616 | 13lp.00 | Main spoken language Malayalam |
| 25632 | ZV70516 | [V]Refugee health examination |
| 25664 | 13gC.00 | Born in Congo |
| 25665 | 13l5.00 | Main spoken language French |
| 25730 | 13dM.00 | Born in Kosovo |
| 25752 | 13dC.00 | Born in England |
| 25802 | 13l1.00 | Main spoken language Bengali |
| 25829 | 13lE.00 | Main spoken language Punjabi |
| 25995 | 13eW.00 | Born in Pakistan |
| 26078 | 13lP.00 | Main spoken language Shona |
| 26196 | 13Z6400 | Language Pashtu |
| 26247 | 13lH.00 | Main spoken language Spanish |
| 26334 | 13eo.00 | Born in Vietnam |
| 26335 | 13lb.00 | Main spoken language Vietnamese |
| 26337 | 13l0.00 | Main spoken language Arabic |
| 26361 | 13lL.00 | Main spoken language Urdu |
| 26426 | 13eF.00 | Born in Iran |
| 26463 | 13dA.00 | Born in Czech Republic |
| 26464 | 13l3.00 | Main spoken language Czech |
| 28301 | 13dP.00 | Born in Lithuania |
| 28529 | 13eg.00 | Born in Syria |
| 30224 | 13gY.00 | Born in Niger |
| 30606 | 13f5.00 | Born in Canada |
| 30800 | 13d..00 | Country of birth (European) |
| 32053 | 13f..00 | Country of birth (American) |
| 32055 | 13d0.00 | Born in Albania |
| 32058 | 13e8.00 | Born in China |
| 32060 | 13e0.00 | Born in Afghanistan |
| 32061 | 13h0.00 | Born in Australia |
| 32062 | 13jC.00 | Born in Trinidad and Tobago |
| 32065 | 13db.00 | Born in Scotland |
| 32067 | 13gJ.00 | Born in Ghana |
| 32068 | 13gl.00 | Born in Uganda |
| 32070 | 13eI.00 | Born in Japan |
| 32072 | 13fL.00 | Born in USA |
| 32074 | 13h1.00 | Born in New Zealand |
| 32075 | 13f3.00 | Born in Brazil |
| 32076 | 13j6.00 | Born in Jamaica |
| 32079 | 13g0.00 | Born in Algeria |
| 32080 | 13dH.00 | Born in Greece |
| 32081 | 13dF.00 | Born in France |
| 32082 | 13eD.00 | Born in India |
| 32085 | 13dG.00 | Born in Germany |
| 32089 | 13j2.00 | Born in Barbados |
| 32090 | 13fN.00 | Born in Venezuela |
| 32094 | 13dW.00 | Born in Poland |
| 32097 | 13de.00 | Born in Spain |
| 32098 | 13df.00 | Born in Sweden |
| 32099 | 13gM.00 | Born in Ivory Coast |
| 32102 | 13g..00 | Country of birth (African) |
| 32103 | 13di.00 | Born in Ukraine |
| 32105 | 13dk.00 | Born in Wales |
| 32108 | 13gV.00 | Born in Morocco |
| 32111 | 13gZ.00 | Born in Nigeria |
| 32112 | 13gN.00 | Born in Kenya |
| 32113 | 13dK.00 | Born in Ireland |
| 32114 | 13ek.00 | Born in Turkey |
| 32115 | 13gS.00 | Born in Malawi |
| 32116 | 13dL.00 | Born in Italy |
| 32117 | 13dU.00 | Born in Northern Ireland |
| 32119 | 13dc.00 | Born in Slovakia |
| 32120 | 13fB.00 | Born in Grenada |
| 32125 | 13e3.00 | Born in Bangladesh |
| 32127 | 13eb.00 | Born in Russia |
| 32128 | 13eM.00 | Born in Kyrgyzstan |
| 32131 | 13j9.00 | Born in St. Lucia |
| 32135 | 13ej.00 | Born in Thailand |
| 32139 | 13dD.00 | Born in Estonia |
| 32140 | 13gU.00 | Born in Mauritius |
| 32144 | 13ec.00 | Born in Saudi Arabia |
| 32150 | 13dh.00 | Born in The Netherlands |
| 32157 | 13ed.00 | Born in Singapore |
| 32158 | 13gG.00 | Born in Ethiopia |
| 32160 | 13dX.00 | Born in Portugal |
| 32162 | 13gW.00 | Born in Mozambique |
| 32166 | 13fJ.00 | Born in Peru |
| 32167 | 13g5.00 | Born in Burundi |
| 32168 | 13gn.00 | Born in Zambia |
| 32169 | 13d7.00 | Born in Bulgaria |
| 32171 | 13eP.00 | Born in Malaysia |
| 32173 | 13dE.00 | Born in Finland |
| 32186 | 13dB.00 | Born in Denmark |
| 32189 | 13ge.00 | Born in Somalia |
| 32190 | 13d9.00 | Born in Cyprus |
| 32197 | 13gd.00 | Born in Sierra Leone |
| 32201 | 13fF.00 | Born in Mexico |
| 32202 | 13e7.00 | Born in Chechnya |
| 32207 | 13gI.00 | Born in Gambia |
| 32217 | 13eT.00 | Born in Nepal |
| 32220 | 13eC.00 | Born in Hong Kong |
| 32233 | 13gX.00 | Born in Namibia |
| 32237 | 13gP.00 | Born in Liberia |
| 32242 | 13dN.00 | Born in Latvia |
| 32245 | 13ef.00 | Born in Sri Lanka |
| 32254 | 13e6.00 | Born in Burma |
| 32255 | 13g7.00 | Born in Cameroon |
| 32260 | 13g1.00 | Born in Angola |
| 32273 | 13e2.00 | Born in Bahrain |
| 32293 | 13k4.00 | Born in Seychelles |
| 32301 | 13j0.00 | Born in Antigua and Barbuda |
| 32303 | 13f7.00 | Born in Columbia |
| 32304 | 13jB.00 | Born in Togo |
| 32309 | 13gc.00 | Born in Senegal |
| 32311 | 13f9.00 | Born in Ecuador |
| 32313 | 13d2.00 | Born in Austria |
| 32325 | 13f0.00 | Born in Argentina |
| 32331 | 13ga.00 | Born in Rwanda |
| 32333 | 13gE.00 | Born in Egypt |
| 32342 | 13f4.00 | Born in British Guyana |
| 32345 | 13dZ.00 | Born in Romania |
| 32347 | 13gL.00 | Born in Guinea Republic |
| 32352 | 13d4.00 | Born in Belgium |
| 32361 | 13e1.00 | Born in Armenia |
| 32369 | 13j4.00 | Born in Dominican Republic |
| 32390 | 13eh.00 | Born in Taiwan |
| 32397 | 13d6.00 | Born in Bosnia - Herzegovnia |
| 32417 | 13k..00 | Country of birth (Pacific) |
| 32427 | 13lF.00 | Main spoken language Russian |
| 32456 | 13lG.00 | Main spoken language Somali |
| 32688 | 13dI.00 | Born in Hungary |
| 32728 | 13lD.00 | Main spoken language Portuguese |
| 32741 | 13gk.00 | Born in Tunisia |
| 32776 | 13lB.00 | Main spoken language Mandarin |
| 32807 | 13dg.00 | Born in Switzerland |
| 36794 | 13gg.00 | Born in Sudan |
| 36852 | 13lW.00 | Main spoken language Japanese |
| 36862 | 13lt.00 | Main spoken language Serbian |
| 36980 | 13lQ.00 | Main spoken language Italian |
| 37197 | 13gR.00 | Born in Madagascar |
| 38075 | 13fD.00 | Born in Guyana |
| 38117 | 13gA.00 | Born in Chad |
| 39974 | 13dS.00 | Born in Moldavia |
| 41209 | 13gm.00 | Born in Zaire |
| 41210 | 13d8.00 | Born in Croatia |
| 41211 | 13eO.00 | Born in Lebanon |
| 41213 | 13dV.00 | Born in Norway |
| 41217 | 13eL.00 | Born in Kuwait |
| 41228 | 13ee.00 | Born in South Korea |
| 41230 | 13f2.00 | Born in Bolivia |
| 41233 | 13d5.00 | Born in Belorussia |
| 41280 | 13fE.00 | Born in Honduras |
| 41289 | 13eE.00 | Born in Indonesia |
| 41290 | 13j3.00 | Born in Cuba |
| 41291 | 13f6.00 | Born in Chile |
| 41292 | 13eK.00 | Born in Kazakhstan |
| 41297 | 13eX.00 | Born in Palestine |
| 41302 | 13el.00 | Born in Turkmenistan |
| 41304 | 13dd.00 | Born in Slovenia |
| 41311 | 13eJ.00 | Born in Jordan |
| 41312 | 13dJ.00 | Born in Iceland |
| 41316 | 13g3.00 | Born in Botswana |
| 41318 | 13g2.00 | Born in Benin |
| 41327 | 13h..00 | Country of birth (Australasian) |
| 41337 | 13d3.00 | Born in Azerbaijan |
| 41341 | 13gQ.00 | Born in Libya |
| 41344 | 13ep.00 | Born in Yemen |
| 41350 | 13gh.00 | Born in Swaziland |
| 41351 | 13eS.00 | Born in Mongolia |
| 41354 | 13e9.00 | Born in Democratic People's Republic of Korea |
| 41356 | 13ea.00 | Born in Republic of Korea |
| 41357 | 13dY.00 | Born in Republic of Ireland |
| 41364 | 13dR.00 | Born in Malta |
| 41365 | 13gK.00 | Born in Guinea Bissau |
| 41367 | 13fM.00 | Born in Uruguay |
| 41372 | 13em.00 | Born in United Arab Emirates |
| 41399 | 13fI.00 | Born in Paraguay |
| 41402 | 13en.00 | Born in Uzbekistan |
| 42635 | 13k0.00 | Born in Fiji |
| 42639 | 13eZ.00 | Born in Qatar |
| 46014 | 13lN.00 | Main spoken language Kurdish |
| 46029 | 13l8.00 | Main spoken language Hindi |
| 46325 | 13lY.00 | Main spoken language Lithuanian |
| 46861 | 13lK.00 | Main spoken language Tamil |
| 46973 | 13ln.00 | Main spoken language Lingala |
| 46974 | 13lV.00 | Main spoken language Greek |
| 47007 | 13li.00 | Main spoken language French Creole |
| 47029 | 13lO.00 | Main spoken language Farsi |
| 47073 | 133Q.00 | Family reunion immigrant |
| 47399 | 13n2.00 | Reads Punjabi |
| 47400 | 13n9.00 | Reads Cantonese |
| 47402 | 13n0.00 | Reads Arabic |
| 47404 | 13n7.00 | Reads Urdu |
| 47559 | 13k6.00 | Born in Tonga |
| 47627 | 13lJ.00 | Main spoken language Sylheti |
| 47628 | 13ld.00 | Main spoken language Amharic |
| 47630 | 13lR.00 | Main spoken language German |
| 47631 | 13lw.00 | Main spoken language Tagalog |
| 47641 | 13lI.00 | Main spoken language Swahili |
| 47643 | 13lu.00 | Main spoken language Sinhala |
| 47644 | 13ly.00 | Main spoken language Tigrinya |
| 47646 | 13lm.00 | Main spoken language Igbo |
| 48002 | 13l6.00 | Main spoken language Gujerati |
| 48029 | 6951.00 | Immigration examination |
| 48297 | 13k5.00 | Born in Solomon Islands |
| 49402 | 13eB.00 | Born in Georgia |
| 49907 | 13j..00 | Country of birth (Atlantic) |
| 51778 | 13eV.00 | Born in Oman |
| 52200 | 13b4.00 | Mirpuri language |
| 52204 | 13nD.00 | Reads Hindi |
| 52209 | 13n8.00 | Reads Bengali |
| 54409 | 13lM.00 | Main spoken language Yoruba |
| 54410 | 13lT.00 | Main spoken language Croatian |
| 54413 | 13lc.00 | Main spoken language Akan |
| 54414 | 13lf.00 | Main spoken language Dutch |
| 54415 | 13lX.00 | Main spoken language Korean |
| 54416 | 13l9.00 | Main spoken language Iba |
| 54417 | 13lv.00 | Main spoken language Swedish |
| 56879 | 13lh.00 | Main spoken language Flemish |
| 57186 | 13eA.00 | Born in East Timor |
| 57189 | 13k7.00 | Born in Tuvalu |
| 57341 | 13nE.00 | Reads Chinese |
| 57343 | 13n5.00 | Reads Spanish |
| 57345 | 13n1.00 | Reads Portuguese |
| 57462 | 13b3.00 | Creole language |
| 57755 | 13ll.00 | Main spoken language Hebrew |
| 57758 | 13lq.00 | Main spoken language Norwegian |
| 58192 | 13j5.00 | Born in Haiti |
| 58193 | 13l7.00 | Main spoken language Hausa |
| 58525 | 13nC.00 | Reads French |
| 58527 | 13g4.00 | Born in Burkina Faso |
| 58528 | 13n3.00 | Reads Russian |
| 58531 | 13n4.00 | Reads Somali |
| 58533 | 13j1.00 | Born in Bahamas |
| 58537 | 13nF.00 | Reads Polish |
| 58552 | 13nA.00 | Reads Czech |
| 58643 | 13lr.00 | Main spoken language Pashto |
| 59657 | 13gH.00 | Born in Gabon |
| 62298 | 13e5.00 | Born in Brunei |
| 63923 | 13fH.00 | Born in Panama |
| 63927 | 13gj.00 | Born in The Gambia |
| 63932 | 13sA.00 | English as a second language |
| 63943 | 13fA.00 | Born in El Salvador |
| 64120 | 13k3.00 | Born in Papua New Guinea |
| 64391 | 13lj.00 | Main spoken language Gaelic |
| 64948 | 13ls.00 | Main spoken language Patois |
| 64949 | 13gp.00 | Born in Eritrea |
| 64984 | 13f8.00 | Born in Costa Rica |
| 65310 | 13eR.00 | Born in Mali |
| 65503 | 69D8.00 | Exam. of refugee |
| 66551 | 13jA.00 | Born in St. Vincent |
| 66553 | 13j8.00 | Born in St. Kitts and Nevis |
| 66560 | 13eU.00 | Born in North Korea |
| 66564 | 13nW.00 | Reads Greek |
| 66565 | 13nR.00 | Reads Italian |
| 66685 | 13la.00 | Main spoken language Ukrainian |
| 66826 | 13n6.00 | Reads Tamil |
| 68778 | 13nS.00 | Reads German |
| 68866 | 13gD.00 | Born in Djibouti |
| 69131 | 13dQ.00 | Born in Luxembourg |
| 69135 | 13j7.00 | Born in Puerto Rico |
| 69139 | 13lg.00 | Main spoken language Ethiopian |
| 69143 | 13gT.00 | Born in Mauritania |
| 69153 | 13lo.00 | Main spoken language Luganda |
| 69426 | 13fK.00 | Born in Suriname |
| 69431 | 13eN.00 | Born in Laos |
| 69560 | 13g8.00 | Born in Cape Verde Islands |
| 69806 | 13fC.00 | Born in Guatemala |
| 71190 | 13dT.00 | Born in Monaco |
| 72379 | 13lA.00 | Main spoken language Kutchi |
| 74892 | 13fG.00 | Born in Nicaragua |
| 90860 | 13nH.00 | Reads Farsi |
| 90868 | 13nG.00 | Reads Lithuanian |
| 91328 | 13eQ.00 | Born in Maldives |
| 91419 | 13nY.00 | Reads Turkish |
| 91420 | 13nQ.00 | Reads Kurdish |
| 91422 | 13lk.00 | Main spoken language Hakka |
| 91423 | 13nJ.00 | Reads Chinese - Traditional |
| 93443 | 13nc.00 | Reads Pashto |
| 93444 | 13nf.00 | Reads Tigrinya |
| 93462 | 13nK.00 | Reads Gujarati |
| 93569 | 13nX.00 | Reads Japanese |
| 93697 | 13gO.00 | Born in Lesotho |
| 93893 | 13nh.00 | Reads Burmese |
| 93923 | 13gF.00 | Born in Equatorial Guinea |
| 93935 | 13d1.00 | Born in Andorra |
| 94050 | 13f1.00 | Born in Belize |
| 94072 | 13ng.00 | Reads Bulgarian |
| 94906 | 13Zd.00 | Failed asylum seeker |
| 95590 | 13nM.00 | Reads Chinese - Simplified |
| 95593 | 13nV.00 | Reads Croatian |
| 95708 | 13dO.00 | Born in Liechtenstein |
| 95775 | 13nZ.00 | Reads Vietnamese |
| 95897 | 13u0.00 | Main spoken language Bulgarian |
| 95940 | 13ur.00 | Main spoken language Latvian |
| 95968 | 13lT.11 | Main spoken language Serbo-Croatian |
| 95969 | 13lt.11 | Main spoken language Serbo-Croatian |
| 95970 | 13lO.11 | Main spoken language Persian |
| 95974 | 13lu.11 | Main spoken language Sinhalese |
| 95978 | 13w1.00 | Main spoken language Nepali |
| 95985 | 13nm.00 | Reads Malay |
| 96041 | 13ua.00 | Main spoken language Hungarian |
| 96146 | 13uh.00 | Main spoken language Irish |
| 96147 | 13lE.11 | Main spoken language Panjabi |
| 96148 | 13u5.00 | Main spoken language Afrikaans |
| 96152 | 13u1.00 | Main spoken language Romanian |
| 96163 | 13wL.00 | Main spoken language Telugu |
| 96223 | 13wR.00 | Main spoken language Twi |
| 96230 | 13wG.00 | Main spoken language Slovenian |
| 96240 | 13wD.00 | Main spoken language Sindhi |
| 96267 | 13ux.00 | Main spoken language Marathi |
| 96268 | 13uj.00 | Main spoken language Kannada |
| 96289 | 13uN.00 | Main spoken language Danish |
| 96290 | 13u6.00 | Main spoken language Armenian |
| 96295 | 13t..00 | Born in British overseas territory |
| 96296 | 13na.00 | Reads Amharic |
| 96317 | 13w5.00 | Main spoken language Quechua |
| 96370 | 13uv.00 | Main spoken language Maltese |
| 96376 | 13uu.00 | Main spoken language Malay |
| 96485 | 13le.00 | Main spoken language Brawa |
| 96558 | 13wM.00 | Main spoken language Tibetan |
| 96559 | 13uG.00 | Main spoken language Burmese |
| 96560 | 13uT.00 | Main spoken language Finnish |
| 96611 | 13us.00 | Main spoken language Macedonian |
| 96634 | 13wN.00 | Main spoken language Tongan |
| 96636 | 13g6.00 | Born in Cambodia |
| 96784 | 13wT.00 | Main spoken language Uzbek |
| 96805 | 13nj.00 | Reads Indonesian |
| 96824 | 13dm.00 | Born in former Yugoslav Republic of Macedonia |
| 96857 | 13u2.00 | Main spoken language Oromo |
| 96858 | 13w2.00 | Main spoken language Occitan |
| 96868 | 13u4.00 | Main spoken language Afar |
| 96873 | 13wa.00 | Main spoken language Zulu |
| 96877 | 13w..00 | Supplemental main language spoken |
| 96928 | 13uQ.00 | Main spoken language Estonian |
| 97015 | 13uk.00 | Main spoken language Kashmiri |
| 97038 | 13uz.00 | Main spoken language Mongolian |
| 97039 | 13wW.00 | Main spoken language Wolof |
| 97041 | 13wQ.00 | Main spoken language Turkmen |
| 97083 | 13u9.00 | Main spoken language Azerbaijani |
| 97131 | 13wH.00 | Main spoken language Sundanese |
| 97136 | 13no.00 | Reads Ndebele |
| 97212 | 13uX.00 | Main spoken language Georgian |
| 97273 | 13wX.00 | Main spoken language Xhosa |
| 97274 | 13uc.00 | Main spoken language Indonesian |
| 97297 | 13w3.00 | Main spoken language Oriya |
| 97298 | 9NUC.11 | Persian language interpreter needed |
| 97390 | 13e4.00 | Born in Bhutan |
| 97439 | 13wB.00 | Main spoken language Southern Sotho |
| 97440 | 13w6.00 | Main spoken language Romansh |
| 97574 | 13uB.00 | Main spoken language Basque |
| 97595 | 13ul.00 | Main spoken language Kazakh |
| 97612 | 13v..00 | Born French overseas region department collectivity territor |
| 97644 | 9NUz.00 | Bulgarian language interpreter needed |
| 97685 | 13wP.00 | Main spoken language Tsonga |
| 97789 | 13uP.00 | Main spoken language Esperanto |
| 97997 | 13uK.00 | Main spoken language Catalan |
| 98038 | 13t1.00 | Born in Bermuda |
| 98062 | 9Nmm.00 | Burmese language interpreter needed |
| 98070 | 13uZ.00 | Main spoken language Guarani |
| 98132 | 13up.00 | Main spoken language Lao |
| 98194 | 13um.00 | Main spoken language Kinyarwanda |
| 98215 | 13uy.00 | Main spoken language Moldavian |
| 98228 | 13nN.00 | Reads Swahili |
| 98229 | 13nn.00 | Reads Mongolian |
| 98255 | 13uw.00 | Main spoken language Maori |
| 98285 | 13w4.00 | Main spoken language Filipino |
| 98369 | 13nP.00 | Reads Yoruba |
| 98510 | 13uY.00 | Main spoken language Kalaallisut |
| 98530 | 13da.00 | Born in San Marino |
| 98604 | 13ub.00 | Main spoken language Icelandic |
| 98762 | 13u8.00 | Main spoken language Aymara |
| 98809 | 9NmQ.00 | Hungarian language interpreter needed |
| 98841 | 9NUy.00 | Romanian language interpreter needed |
| 98942 | 13nb.00 | Reads Lingala |
| 99119 | 13dj.00 | Born in Vatican City |
| 99258 | 13g9.00 | Born in Central African Republic |
| 99431 | 13jD.00 | Born in Dominica |
| 99712 | 13wS.00 | Main spoken language Uighur |
| 99740 | 13nd.00 | Reads Serbian |
| 99794 | 9Nn1.00 | Tsonga language interpreter needed |
| 100007 | 13ei.00 | Born in Tajikistan |
| 100010 | 13nT.00 | Reads Albanian |
| 100011 | 13ui.00 | Main spoken language Javanese |
| 100013 | 13uL.00 | Main spoken language Slovak |
| 100438 | 9NmA.00 | Macedonian language interpreter needed |
| 100517 | 13dn.00 | Born in Serbia |
| 100707 | 13uR.00 | Main spoken language Faeroese |
| 100714 | 13uS.00 | Main spoken language Fijian |
| 100716 | 13ug.00 | Main spoken language Inuktitut |
| 100743 | 13uW.00 | Main spoken language Galician |
| 100759 | 9Nn7.00 | Slovenian language interpreter needed |
| 100813 | 9NmM.00 | Interlingue language interpreter needed |
| 100828 | 13uH.00 | Main spoken language Belarusian |
| 100949 | 13uD.00 | Main spoken language Bihari |
| 101038 | 13wb.00 | Main spoken language Konkani |
| 101158 | 13gB.00 | Born in Comoros Islands |
| 101189 | 13uJ.00 | Main spoken language Central Khmer |
| 101220 | 13wA.00 | Main spoken language Dari |
| 101284 | 9NnK.00 | Nepali language interpreter needed |
| 101591 | 13gb.00 | Born in Sao Tome and Principe |
| 101614 | 9Nn4.00 | Telugu language interpreter needed |
| 101620 | 13ut.00 | Main spoken language Malagasy |
| 101659 | 13ud.00 | Main spoken language Interlingua |
| 101761 | 13l9.11 | Main spoken language Iban |
| 101788 | 13uM.00 | Main spoken language Corsican |
| 101814 | 9NUc.11 | Punjabi language interpreter needed |
| 102007 | 13uY.11 | Main spoken language Greenlandic |
| 102127 | 13uC.00 | Main spoken language Dzongkha |
| 102128 | 13uF.00 | Main spoken language Breton |
| 102129 | 13ue.00 | Main spoken language Interlingue |
| 102184 | 13u3.00 | Main spoken language Abkhazian |
| 102218 | 13u7.00 | Main spoken language Assamese |
| 102259 | 13uA.00 | Main spoken language Bashkir |
| 102877 | 13wC.00 | Main spoken language Tswana |
| 103200 | 13uV.00 | Main spoken language Frisian |
| 103219 | 13w7.00 | Main spoken language Samoan |
| 103364 | 13v0.00 | Born in Martinique |
| 103965 | 13k9.00 | Born in Western Samoa |
| 104071 | 13uq.00 | Main spoken language Bamun |
| 104123 | 13Zw.00 | Has United Kingdom student visa |
| 104284 | 13ni.00 | Reads Chechen |
| 104635 | 9NmC.00 | Latvian language interpreter needed |
| 104678 | 9Nmx.00 | Oromo language interpreter needed |
| 104886 | 13wE.00 | Main spoken language Ndebele |
| 104901 | 9Nm6.00 | Brawa language interpreter needed |
| 104983 | 13t2.00 | Born in Anguilla |
| 105079 | 9Nmd.00 | Catalan language interpreter needed |
| 105153 | 133A000 | International student |
| 105523 | 13wV.00 | Main spoken language Tetum |
| 105529 | 13wc.00 | Main spoken language Aragonese |
| 105608 | 13nk.00 | Reads Kinyarwanda |
| 105923 | 13t0.00 | Born in Montserrat |
| 105960 | 13wJ.00 | Main spoken language Tajik |
| 107687 | 133A011 | Overseas student |
| 108184 | 9Nn6.00 | Turkmen language interpreter needed |
| 108271 | 13jE.00 | Born in Aruba |
| 108936 | 13v7.00 | Born in Guadeloupe |
| 109092 | 13ds.00 | Born in Jersey |
| 109093 | 13dr.00 | Born in Guernsey |
| 109226 | 13eq.00 | Born in Christmas Island |
| 109260 | 13dq.00 | Born in Republic of Moldova |
| 109276 | 13t5.00 | Born in St Helena, Ascension and Tristan da Cunha |
| 109457 | 13do.00 | Born in Montenegro |
| 109458 | 13jG.00 | Born in Saint Vincent and the Grenadines |
| 109489 | 9NmE.00 | Kinyarwanda language interpreter needed |
| 109727 | 13t3.00 | Born in British Virgin Islands |
| 109791 | 13kB.00 | Born in American Samoa |
| 109898 | 13dp.00 | Born in Belarus |
| 109992 | 13gq.00 | Born in Democratic Republic of Congo |

vii) Ethnicity

| Medcode | Readcode | Readterm |
| --- | --- | --- |
| 10196 | 9S...00 | Ethnic groups (1991 census) |
| 22467 | 9S1..00 | White |
| 12446 | 9S10.00 | White British |
| 24837 | 9S11.00 | White Irish |
| 12444 | 9S12.00 | Other white ethnic group |
| 26467 | 9S13.00 | White Scottish |
| 26310 | 9S14.00 | Other white British ethnic group |
| 12632 | 9S2..00 | Black Caribbean |
| 12778 | 9S3..00 | Black African |
| 24339 | 9S4..00 | Black, other, non-mixed origin |
| 12452 | 9S41.00 | Black British |
| 57435 | 9S42.00 | Black Caribbean/W.I./Guyana |
| 47950 | 9S42.11 | Black Caribbean |
| 47997 | 9S42.12 | Black West Indian |
| 32100 | 9S42.13 | Black Guyana |
| 41329 | 9S43.00 | Black N African/Arab/Iranian |
| 46812 | 9S43.11 | Black North African |
| 57752 | 9S43.12 | Black Arab |
| 50286 | 9S43.13 | Black Iranian |
| 35412 | 9S44.00 | Black - other African country |
| 47965 | 9S45.00 | Black E Afric Asia/Indo-Caribb |
| 57753 | 9S45.11 | Black East African Asian |
| 57763 | 9S45.12 | Black Indo-Caribbean |
| 48005 | 9S46.00 | Black Indian sub-continent |
| 35350 | 9S47.00 | Black - other Asian |
| 26312 | 9S48.00 | Black Black - other |
| 25676 | 9S5..00 | Black - other, mixed |
| 25623 | 9S51.00 | Other Black - Black/White orig |
| 32165 | 9S52.00 | Other Black - Black/Asian orig |
| 12482 | 9S6..00 | Indian |
| 24690 | 9S7..00 | Pakistani |
| 24740 | 9S8..00 | Bangladeshi |
| 24272 | 9S9..00 | Chinese |
| 30280 | 9SA..00 | Other ethnic non-mixed (NMO) |
| 32110 | 9SA1.00 | Brit. ethnic minor. spec.(NMO) |
| 57764 | 9SA2.00 | Brit. ethnic minor. unsp (NMO) |
| 54593 | 9SA3.00 | Caribbean I./W.I./Guyana (NMO) |
| 57094 | 9SA3.11 | Caribbean Island (NMO) |
| 57075 | 9SA3.12 | West Indian (NMO) |
| 93144 | 9SA3.13 | Guyana (NMO) |
| 24962 | 9SA4.00 | N African Arab/Iranian (NMO) |
| 47285 | 9SA4.11 | North African Arab (NMO) |
| 25082 | 9SA4.12 | Iranian (NMO) |
| 47969 | 9SA5.00 | Other African countries (NMO) |
| 38097 | 9SA6.00 | E Afric Asian/Indo-Carib (NMO) |
| 46818 | 9SA6.11 | East African Asian (NMO) |
| 99316 | 9SA6.12 | Indo-Caribbean (NMO) |
| 39696 | 9SA7.00 | Indian sub-continent (NMO) |
| 26379 | 9SA8.00 | Other Asian (NMO) |
| 24270 | 9SA9.00 | Irish (NMO) |
| 45947 | 9SAA.00 | Greek/Greek Cypriot (NMO) |
| 45955 | 9SAA.11 | Greek (NMO) |
| 47949 | 9SAA.12 | Greek Cypriot (NMO) |
| 32066 | 9SAB.00 | Turkish/Turkish Cypriot (NMO) |
| 32126 | 9SAB.11 | Turkish (NMO) |
| 32069 | 9SAB.12 | Turkish Cypriot (NMO) |
| 12633 | 9SAC.00 | Other European (NMO) |
| 41214 | 9SAD.00 | Other ethnic NEC (NMO) |
| 12696 | 9SB..00 | Other ethnic, mixed origin |
| 47401 | 9SB1.00 | Other ethnic, Black/White orig |
| 32401 | 9SB2.00 | Other ethnic, Asian/White orig |
| 35459 | 9SB3.00 | Other ethnic, mixed white orig |
| 32420 | 9SB4.00 | Other ethnic, other mixed orig |
| 32425 | 9SB5.00 | Black Caribbean and White |
| 32443 | 9SB6.00 | Black African and White |
| 25411 | 9SC..00 | Vietnamese |
| 12429 | 9SD..00 | Ethnic group not given - patient refused |
| 24340 | 9SE..00 | Ethnic group not recorded |
| 32136 | 9SG..00 | Other black ethnic group |
| 12668 | 9SH..00 | Other Asian ethnic group |
| 47601 | 9SI..00 | Irish traveller |
| 12757 | 9SJ..00 | Other ethnic group |
| 45199 | 9SZ..00 | Ethnic groups (census) NOS |
| 23955 | 9T...00 | Ethnicity and other related nationality data |
| 45008 | 9T1..00 | New Zealand ethnic groups |
| 57286 | 9T11.00 | New Zealand European |
| 85509 | 9T11.11 | Pakeha |
| 85505 | 9T12.00 | Other European in New Zealand |
| 32479 | 9T13.00 | New Zealand Maori |
| 64610 | 9T14.00 | Samoan |
| 89910 | 9T15.00 | Cook Island Maori |
| 60837 | 9T16.00 | Tongan |
| 55584 | 9T17.00 | Niuean |
| 25434 | 9T18.00 | Tokelauan |
| 64609 | 9T19.00 | Fijian |
| 46752 | 9T1A.00 | Other Pacific ethnic group |
| 46649 | 9T1B.00 | South East Asian |
| 12718 | 9T1C.00 | Chinese |
| 25920 | 9T1D.00 | Indian |
| 32396 | 9T1E.00 | Other Asian |
| 96789 | 9T1Y.00 | Other New Zealand ethnic group |
| 71425 | 9T1Z.00 | New Zealand ethnic group NOS |
| 32781 | 9T2..00 | Traveller - gypsy |
| 94487 | 9T3..00 | Yemeni |
| 12435 | 9i...00 | Ethnic category - 2001 census |
| 12351 | 9i0..00 | British or mixed British - ethnic category 2001 census |
| 98111 | 9i00.00 | White British - ethnic category 2001 census |
| 12532 | 9i1..00 | Irish - ethnic category 2001 census |
| 98213 | 9i10.00 | White Irish - ethnic category 2001 census |
| 12421 | 9i2..00 | Other White background - ethnic category 2001 census |
| 12352 | 9i20.00 | English - ethnic category 2001 census |
| 12436 | 9i21.00 | Scottish - ethnic category 2001 census |
| 12681 | 9i22.00 | Welsh - ethnic category 2001 census |
| 28887 | 9i23.00 | Cornish - ethnic category 2001 census |
| 42294 | 9i24.00 | Northern Irish - ethnic category 2001 census |
| 40102 | 9i25.00 | Ulster Scots - ethnic category 2001 census |
| 32778 | 9i26.00 | Cypriot (part not stated) - ethnic category 2001 census |
| 12355 | 9i27.00 | Greek - ethnic category 2001 census |
| 12769 | 9i28.00 | Greek Cypriot - ethnic category 2001 census |
| 12746 | 9i29.00 | Turkish - ethnic category 2001 census |
| 32413 | 9i2A.00 | Turkish Cypriot - ethnic category 2001 census |
| 12412 | 9i2B.00 | Italian - ethnic category 2001 census |
| 55223 | 9i2C.00 | Irish Traveller - ethnic category 2001 census |
| 55113 | 9i2D.00 | Traveller - ethnic category 2001 census |
| 42290 | 9i2E.00 | Gypsy/Romany - ethnic category 2001 census |
| 12467 | 9i2F.00 | Polish - ethnic category 2001 census |
| 12433 | 9i2G.00 | Baltic Estonian/Latvian/Lithuanian - ethn categ 2001 census |
| 28973 | 9i2H.00 | Commonwealth (Russian) Indep States - ethn categ 2001 census |
| 26341 | 9i2J.00 | Kosovan - ethnic category 2001 census |
| 25422 | 9i2K.00 | Albanian - ethnic category 2001 census |
| 46956 | 9i2L.00 | Bosnian - ethnic category 2001 census |
| 28866 | 9i2M.00 | Croatian - ethnic category 2001 census |
| 47074 | 9i2N.00 | Serbian - ethnic category 2001 census |
| 28936 | 9i2P.00 | Other republics former Yugoslavia - ethnic categ 2001 census |
| 26391 | 9i2Q.00 | Mixed Irish and other White - ethnic category 2001 census |
| 12402 | 9i2R.00 | Oth White European/European unsp/Mixed European 2001 census |
| 28900 | 9i2S.00 | Other mixed White - ethnic category 2001 census |
| 12591 | 9i2T.00 | Other White or White unspecified ethnic category 2001 census |
| 12742 | 9i3..00 | White and Black Caribbean - ethnic category 2001 census |
| 12437 | 9i4..00 | White and Black African - ethnic category 2001 census |
| 12638 | 9i5..00 | White and Asian - ethnic category 2001 census |
| 12873 | 9i6..00 | Other Mixed background - ethnic category 2001 census |
| 12795 | 9i60.00 | Black and Asian - ethnic category 2001 census |
| 49940 | 9i61.00 | Black and Chinese - ethnic category 2001 census |
| 40110 | 9i62.00 | Black and White - ethnic category 2001 census |
| 12706 | 9i63.00 | Chinese and White - ethnic category 2001 census |
| 47005 | 9i64.00 | Asian and Chinese - ethnic category 2001 census |
| 32408 | 9i65.00 | Other Mixed or Mixed unspecified ethnic category 2001 census |
| 12414 | 9i7..00 | Indian or British Indian - ethnic category 2001 census |
| 12460 | 9i8..00 | Pakistani or British Pakistani - ethnic category 2001 census |
| 28888 | 9i9..00 | Bangladeshi or British Bangladeshi - ethn categ 2001 census |
| 12513 | 9iA..00 | Other Asian background - ethnic category 2001 census |
| 26392 | 9iA1.00 | Punjabi - ethnic category 2001 census |
| 64133 | 9iA2.00 | Kashmiri - ethnic category 2001 census |
| 47077 | 9iA3.00 | East African Asian - ethnic category 2001 census |
| 12608 | 9iA4.00 | Sri Lankan - ethnic category 2001 census |
| 12760 | 9iA5.00 | Tamil - ethnic category 2001 census |
| 12887 | 9iA6.00 | Sinhalese - ethnic category 2001 census |
| 32399 | 9iA7.00 | Caribbean Asian - ethnic category 2001 census |
| 12653 | 9iA8.00 | British Asian - ethnic category 2001 census |
| 46056 | 9iA9.00 | Mixed Asian - ethnic category 2001 census |
| 28935 | 9iAA.00 | Other Asian or Asian unspecified ethnic category 2001 census |
| 12432 | 9iB..00 | Caribbean - ethnic category 2001 census |
| 12350 | 9iC..00 | African - ethnic category 2001 census |
| 32389 | 9iD..00 | Other Black background - ethnic category 2001 census |
| 12443 | 9iD0.00 | Somali - ethnic category 2001 census |
| 32886 | 9iD1.00 | Nigerian - ethnic category 2001 census |
| 40097 | 9iD2.00 | Black British - ethnic category 2001 census |
| 40096 | 9iD3.00 | Mixed Black - ethnic category 2001 census |
| 46047 | 9iD4.00 | Other Black or Black unspecified ethnic category 2001 census |
| 12468 | 9iE..00 | Chinese - ethnic category 2001 census |
| 12434 | 9iF..00 | Other - ethnic category 2001 census |
| 12719 | 9iF0.00 | Vietnamese - ethnic category 2001 census |
| 12473 | 9iF1.00 | Japanese - ethnic category 2001 census |
| 12420 | 9iF2.00 | Filipino - ethnic category 2001 census |
| 12730 | 9iF3.00 | Malaysian - ethnic category 2001 census |
| 63872 | 9iF4.00 | Buddhist - ethnic category 2001 census |
| 56127 | 9iF5.00 | Hindu - ethnic category 2001 census |
| 46063 | 9iF6.00 | Jewish - ethnic category 2001 census |
| 47091 | 9iF7.00 | Muslim - ethnic category 2001 census |
| 49658 | 9iF8.00 | Sikh - ethnic category 2001 census |
| 46059 | 9iF9.00 | Arab - ethnic category 2001 census |
| 47028 | 9iFA.00 | North African - ethnic category 2001 census |
| 28909 | 9iFB.00 | Mid East (excl Israeli, Iranian & Arab) - eth cat 2001 cens |
| 46964 | 9iFC.00 | Israeli - ethnic category 2001 census |
| 25937 | 9iFD.00 | Iranian - ethnic category 2001 census |
| 45964 | 9iFE.00 | Kurdish - ethnic category 2001 census |
| 25451 | 9iFF.00 | Moroccan - ethnic category 2001 census |
| 26246 | 9iFG.00 | Latin American - ethnic category 2001 census |
| 12756 | 9iFH.00 | South and Central American - ethnic category 2001 census |
| 32382 | 9iFJ.00 | Mauritian/Seychellois/Maldivian/St Helena eth cat 2001census |
| 26455 | 9iFK.00 | Any other group - ethnic category 2001 census |
| 12459 | 9iG..00 | Ethnic category not stated - 2001 census |

B) Deriving residence information from patient file in Hospital Episodes Statistics

| **Code** | **Description** | **Information extracted** |
| --- | --- | --- |
| 19 | The usual place of residence, including no fixed abode | Not used |
| 29 | Temporary place of residence when usually resident elsewhere, for example, hotels and residential educational establishments | Not used |
| 30 | Repatriation from high security psychiatric hospital (1999-00 to 2006-07) | residence: other |
| 37 | Penal establishment: court (1999-00 to 2006-07) | Not used |
| 38 | Penal establishment: police station (1999-00 to 2006-07) | Not used |
| 39 | Penal establishment (court and police station excluded from 1999-2000) | residence: other |
| 48 | High security psychiatric hospital, Scotland (1999-00 to 2006-07) | residence: other |
| 49 | NHS other hospital provider: high security psychiatric accommodation in an NHS hospital provider (NHS trust) | residence: other |
| 50 | NHS other hospital provider: medium secure unit (1999-00 to 2006-07) | residence: other |
| 51 | NHS other hospital provider: ward for general patients or the younger physically disabled or A&E department | Not used |
| 52 | NHS other hospital provider: ward for maternity patients or neonates | Not used |
| 53 | NHS other hospital provider: ward for patients who are mentally ill or have learning disabilities | Not used |
| 54 | NHS run nursing home, residential care home or group home | care home residence |
| 65 | Local authority Part 3 residential accommodation: where care is provided (from 1996-97) | sheltered accommodation |
| 66 | Local authority foster care, but not in Part 3 residential accommodation: where care is provided (from 1996-97) | residence: other |
| 69 | Local authority home or care (1989-90 to 1995-96) | care home residence |
| 79 | Babies born in or on the way to hospital | Not used |
| 85 | Non-NHS (other than Local Authority) run residential care home (from 1996-97) | care home residence |
| 86 | Non-NHS (other than Local Authority) run nursing home (from 1996-97 to 2006-07) | care home residence |
| 87 | Non-NHS run hospital | Not used |
| 88 | non-NHS (other than Local Authority) run hospice | residence: other |
| 89 | Non-NHS institution (1989-90 to 1995-96) | Not used |
| 98 | Not applicable | Not used |
| 99 | Not known | Not used |

**Codelist: co-morbidities and medications**

1. Systemic lupus erythematosus
2. CPRD

| **medcode** | **readcode** | **readterm** |
| --- | --- | --- |
| 4125 | M154.00 | Lupus erythematosus |
| 7522 | M154z00 | Lupus erythematosus NOS |
| 7871 | N000.00 | Systemic lupus erythematosus |
| 11920 | N000400 | Systemic lupus erythematosus with pericarditis |
| 20007 | N000000 | Disseminated lupus erythematosus |
| 22205 | K01x411 | Lupus nephritis |
| 29519 | N000300 | Systemic lupus erythematosus with organ or sys involv |
| 31564 | H57y400 | Lung disease with systemic lupus erythematosus |
| 33449 | M154000 | Lupus erythematosus chronicus |
| 36942 | N000200 | Drug-induced systemic lupus erythematosus |
| 40797 | M154200 | Lupus erythematosus migrans |
| 42719 | N000z00 | Systemic lupus erythematosus NOS |
| 44095 | F371000 | Polyneuropathy in disseminated lupus erythematosus |
| 45726 | ZRq9.00 | Systemic lupus erythematosus disease activity index |
| 47047 | ZR2l.11 | BILAG - British isles lupus assessment group score |
| 47672 | K01x400 | Nephrotic syndrome in systemic lupus erythematosus |
| 51798 | ZRq8.00 | Systemic lupus activity measure |
| 57675 | N000100 | Libman-Sacks disease |
| 58706 | Nyu4300 | [X]Other forms of systemic lupus erythematosus |
| 63283 | ZRq9.11 | SLEDAI-Sys lup ery dis act ind |
| 63955 | M154600 | Lupus erythematosus unguium mutilans |
| 65391 | M154300 | Lupus erythematosus nodularis |
| 101433 | N000600 | Cerebral lupus |
| 106086 | ZRq8.11 | SLAM - Systemic lupus activity measure |
| 108072 | F396100 | Myopathy due to disseminated lupus erythematosus |

1. Hospital Episodes Statistics

| **ICD 10** | **DESCRIPTION** |
| --- | --- |
| M32 | Systemic lupus erythematosus |
| M32.0 | Drug-induced systemic lupus erythematosus |
| M32.1 | Systemic lupus erythematosus with organ or system involvement |
| M32.8 | Other forms of systemic lupus erythematosus |
| M32.9 | Systemic lupus erythematosus, unspecified |

1. Rheumatoid arthritis
2. CPRD

| **medcode** | **readcode** | **readterm** |
| --- | --- | --- |
| 844 | N040.00 | Rheumatoid arthritis |
| 4186 | N043.00 | Juvenile rheumatoid arthritis - Still's disease |
| 5723 | N042200 | Rheumatoid nodule |
| 6916 | N040P00 | Seronegative rheumatoid arthritis |
| 8350 | N040T00 | Flare of rheumatoid arthritis |
| 9707 | N047.00 | Seropositive errosive rheumatoid arthritis |
| 9954 | H570.00 | Rheumatoid lung |
| 12019 | N04X.00 | Seropositive rheumatoid arthritis, unspecified |
| 17412 | 66H..13 | Rheumatoid arthrit. monitoring |
| 18155 | N040Q00 | Rheumatoid bursitis |
| 21358 | N040200 | Rheumatoid arthritis of shoulder |
| 21533 | N043200 | Pauciarticular juvenile rheumatoid arthritis |
| 23552 | N041.00 | Felty's syndrome |
| 23834 | N005.00 | Adult Still's Disease |
| 27557 | N043z00 | Juvenile rheumatoid arthritis NOS |
| 28853 | N04y012 | Fibrosing alveolitis associated with rheumatoid arthritis |
| 30548 | N040N00 | Rheumatoid vasculitis |
| 31054 | N040S00 | Rheumatoid arthritis - multiple joint |
| 31209 | F396400 | Myopathy due to rheumatoid arthritis |
| 31360 | N045500 | Juvenile rheumatoid arthritis |
| 31724 | N04y000 | Rheumatoid lung |
| 32001 | N04y200 | Adult-onset Still's disease |
| 33264 | 2G27.00 | O/E-hands-rheumatoid spindling |
| 36276 | N043300 | Monarticular juvenile rheumatoid arthritis |
| 37431 | N042z00 | Rheumatoid arthropathy + visceral/systemic involvement NOS |
| 41941 | N040900 | Rheumatoid arthritis of PIP joint of finger |
| 42299 | N040800 | Rheumatoid arthritis of MCP joint |
| 42719 | N000z00 | Systemic lupus erythematosus NOS |
| 43816 | G5yA.00 | Rheumatoid carditis |
| 44203 | N040100 | Other rheumatoid arthritis of spine |
| 44743 | N040000 | Rheumatoid arthritis of cervical spine |
| 46436 | N042100 | Rheumatoid lung disease |
| 47831 | N043100 | Acute polyarticular juvenile rheumatoid arthritis |
| 48832 | N040700 | Rheumatoid arthritis of wrist |
| 49067 | N040B00 | Rheumatoid arthritis of hip |
| 49227 | N042.00 | Other rheumatoid arthropathy + visceral/systemic involvement |
| 49787 | G5y8.00 | Rheumatoid myocarditis |
| 50644 | N043000 | Juvenile rheumatoid arthropathy unspecified |
| 50863 | N040D00 | Rheumatoid arthritis of knee |
| 51238 | N040K00 | Rheumatoid arthritis of 1st MTP joint |
| 51239 | N040F00 | Rheumatoid arthritis of ankle |
| 53621 | N040R00 | Rheumatoid nodule |
| 56202 | Nyu1G00 | [X]Seropositive rheumatoid arthritis, unspecified |
| 56838 | N04y011 | Caplan's syndrome |
| 59738 | N040500 | Rheumatoid arthritis of elbow |
| 62401 | F371200 | Polyneuropathy in rheumatoid arthritis |
| 63198 | N040A00 | Rheumatoid arthritis of DIP joint of finger |
| 63365 | N040600 | Rheumatoid arthritis of distal radio-ulnar joint |
| 70221 | Nyu1200 | [X]Other specified rheumatoid arthritis |
| 70658 | N040H00 | Rheumatoid arthritis of talonavicular joint |
| 71784 | N040J00 | Rheumatoid arthritis of other tarsal joint |
| 73619 | N040G00 | Rheumatoid arthritis of subtalar joint |
| 93715 | Nyu1100 | [X]Other seropositive rheumatoid arthritis |
| 99414 | N040L00 | Rheumatoid arthritis of lesser MTP joint |
| 100776 | N040C00 | Rheumatoid arthritis of sacro-iliac joint |
| 100914 | N040400 | Rheumatoid arthritis of acromioclavicular joint |
| 102088 | 7P20300 | Delivery of rehabilitation for rheumatoid arthritis |
| 103829 | 38DZ000 | Disease activity score 28 joint in rheumatoid arthritis |
| 105507 | 66HB000 | Rheumatoid arthritis annual review |
| 106092 | 9hR1.00 | Except rheumatoid arthritis qual indicator: informed dissent |
| 106093 | 9hR..00 | Exception reporting: rheumatoid arthritis quality indicators |
| 106118 | 9hR0.00 | Except rheumatoid arthritis quality indicator: pt unsuitable |
| 106440 | Nyu1000 | [X]Rheumatoid arthritis+involvement/other organs or systems |
| 107112 | N040M00 | Rheumatoid arthritis of IP joint of toe |
| 107340 | 9mM..00 | Rheumatoid arthritis monitoring invitation |
| 107435 | 9mM0.00 | Rheumatoid arthritis monitoring invitation first letter |
| 107575 | 9mM1.00 | Rheumatoid arthritis monitoring invitation second letter |
| 107606 | 9mM3.00 | Rheumatoid arthritis monitoring verbal invitation |
| 107676 | 9mM2.00 | Rheumatoid arthritis monitoring invitation third letter |
| 107791 | N040E00 | Rheumatoid arthritis of tibio-fibular joint |
| 107797 | 9mM4.00 | Rheumatoid arthritis monitoring telephone invitation |
| 107963 | N040300 | Rheumatoid arthritis of sternoclavicular joint |

1. Hospital Episodes Statistics

| **ICD 10** | **DESCRIPTION** |
| --- | --- |
| J99.0 | Rheumatoid lung disease |
| M05 | Seropositive rheumatoid arthritis |
| M05.0 | Felty's syndrome |
| M05.00 | Felty's syndrome |
| M05.01 | Felty's syndrome |
| M05.02 | Felty's syndrome |
| M05.03 | Felty's syndrome |
| M05.04 | Felty's syndrome |
| M05.05 | Felty's syndrome |
| M05.06 | Felty's syndrome |
| M05.07 | Felty's syndrome |
| M05.08 | Felty's syndrome |
| M05.09 | Felty's syndrome |
| M05.1 | Rheumatoid lung disease |
| M05.10 | Rheumatoid lung disease |
| M05.11 | Rheumatoid lung disease |
| M05.12 | Rheumatoid lung disease |
| M05.13 | Rheumatoid lung disease |
| M05.14 | Rheumatoid lung disease |
| M05.15 | Rheumatoid lung disease |
| M05.16 | Rheumatoid lung disease |
| M05.17 | Rheumatoid lung disease |
| M05.18 | Rheumatoid lung disease |
| M05.19 | Rheumatoid lung disease |
| M05.2 | Rheumatoid vasculitis |
| M05.20 | Rheumatoid vasculitis |
| M05.21 | Rheumatoid vasculitis |
| M05.22 | Rheumatoid vasculitis |
| M05.23 | Rheumatoid vasculitis |
| M05.24 | Rheumatoid vasculitis |
| M05.25 | Rheumatoid vasculitis |
| M05.26 | Rheumatoid vasculitis |
| M05.27 | Rheumatoid vasculitis |
| M05.28 | Rheumatoid vasculitis |
| M05.29 | Rheumatoid vasculitis |
| M05.3 | Rheumatoid arthritis with involvement of other organs and systems |
| M05.30 | Rheumatoid arthritis with involvement of other organs and systems |
| M05.31 | Rheumatoid arthritis with involvement of other organs and systems |
| M05.32 | Rheumatoid arthritis with involvement of other organs and systems |
| M05.33 | Rheumatoid arthritis with involvement of other organs and systems |
| M05.34 | Rheumatoid arthritis with involvement of other organs and systems |
| M05.35 | Rheumatoid arthritis with involvement of other organs and systems |
| M05.36 | Rheumatoid arthritis with involvement of other organs and systems |
| M05.37 | Rheumatoid arthritis with involvement of other organs and systems |
| M05.38 | Rheumatoid arthritis with involvement of other organs and systems |
| M05.39 | Rheumatoid arthritis with involvement of other organs and systems |
| M05.8 | Other seropositive rheumatoid arthritis |
| M05.80 | Other seropositive rheumatoid arthritis |
| M05.81 | Other seropositive rheumatoid arthritis |
| M05.82 | Other seropositive rheumatoid arthritis |
| M05.83 | Other seropositive rheumatoid arthritis |
| M05.84 | Other seropositive rheumatoid arthritis |
| M05.85 | Other seropositive rheumatoid arthritis |
| M05.86 | Other seropositive rheumatoid arthritis |
| M05.87 | Other seropositive rheumatoid arthritis |
| M05.88 | Other seropositive rheumatoid arthritis |
| M05.89 | Other seropositive rheumatoid arthritis |
| M05.9 | Seropositive rheumatoid arthritis, unspecified |
| M05.90 | Seropositive rheumatoid arthritis, unspecified |
| M05.91 | Seropositive rheumatoid arthritis, unspecified |
| M05.92 | Seropositive rheumatoid arthritis, unspecified |
| M05.93 | Seropositive rheumatoid arthritis, unspecified |
| M05.94 | Seropositive rheumatoid arthritis, unspecified |
| M05.95 | Seropositive rheumatoid arthritis, unspecified |
| M05.96 | Seropositive rheumatoid arthritis, unspecified |
| M05.97 | Seropositive rheumatoid arthritis, unspecified |
| M05.98 | Seropositive rheumatoid arthritis, unspecified |
| M05.99 | Seropositive rheumatoid arthritis, unspecified |
| M06 | Other rheumatoid arthritis |
| M06.0 | Seronegative rheumatoid arthritis |
| M06.00 | Seronegative rheumatoid arthritis |
| M06.01 | Seronegative rheumatoid arthritis |
| M06.02 | Seronegative rheumatoid arthritis |
| M06.03 | Seronegative rheumatoid arthritis |
| M06.04 | Seronegative rheumatoid arthritis |
| M06.05 | Seronegative rheumatoid arthritis |
| M06.06 | Seronegative rheumatoid arthritis |
| M06.07 | Seronegative rheumatoid arthritis |
| M06.08 | Seronegative rheumatoid arthritis |
| M06.09 | Seronegative rheumatoid arthritis |
| M06.1 | Adult-onset Still's disease |
| M06.10 | Adult-onset Still's disease |
| M06.11 | Adult-onset Still's disease |
| M06.12 | Adult-onset Still's disease |
| M06.13 | Adult-onset Still's disease |
| M06.14 | Adult-onset Still's disease |
| M06.15 | Adult-onset Still's disease |
| M06.16 | Adult-onset Still's disease |
| M06.17 | Adult-onset Still's disease |
| M06.18 | Adult-onset Still's disease |
| M06.19 | Adult-onset Still's disease |
| M06.2 | Rheumatoid bursitis |
| M06.20 | Rheumatoid bursitis |
| M06.21 | Rheumatoid bursitis |
| M06.22 | Rheumatoid bursitis |
| M06.23 | Rheumatoid bursitis |
| M06.24 | Rheumatoid bursitis |
| M06.25 | Rheumatoid bursitis |
| M06.26 | Rheumatoid bursitis |
| M06.27 | Rheumatoid bursitis |
| M06.28 | Rheumatoid bursitis |
| M06.29 | Rheumatoid bursitis |
| M06.3 | Rheumatoid nodule |
| M06.30 | Rheumatoid nodule |
| M06.31 | Rheumatoid nodule |
| M06.32 | Rheumatoid nodule |
| M06.33 | Rheumatoid nodule |
| M06.34 | Rheumatoid nodule |
| M06.35 | Rheumatoid nodule |
| M06.36 | Rheumatoid nodule |
| M06.37 | Rheumatoid nodule |
| M06.38 | Rheumatoid nodule |
| M06.39 | Rheumatoid nodule |
| M06.8 | Other specified rheumatoid arthritis |
| M06.80 | Other specified rheumatoid arthritis |
| M06.81 | Other specified rheumatoid arthritis |
| M06.82 | Other specified rheumatoid arthritis |
| M06.83 | Other specified rheumatoid arthritis |
| M06.84 | Other specified rheumatoid arthritis |
| M06.85 | Other specified rheumatoid arthritis |
| M06.86 | Other specified rheumatoid arthritis |
| M06.87 | Other specified rheumatoid arthritis |
| M06.88 | Other specified rheumatoid arthritis |
| M06.89 | Other specified rheumatoid arthritis |
| M06.9 | Rheumatoid arthritis, unspecified |
| M06.90 | Rheumatoid arthritis, unspecified |
| M06.91 | Rheumatoid arthritis, unspecified |
| M06.92 | Rheumatoid arthritis, unspecified |
| M06.93 | Rheumatoid arthritis, unspecified |
| M06.94 | Rheumatoid arthritis, unspecified |
| M06.95 | Rheumatoid arthritis, unspecified |
| M06.96 | Rheumatoid arthritis, unspecified |
| M06.97 | Rheumatoid arthritis, unspecified |
| M06.98 | Rheumatoid arthritis, unspecified |
| M06.99 | Rheumatoid arthritis, unspecified |
| M08.0 | Juvenile rheumatoid arthritis |
| M08.00 | Juvenile rheumatoid arthritis |
| M08.01 | Juvenile rheumatoid arthritis |
| M08.02 | Juvenile rheumatoid arthritis |
| M08.03 | Juvenile rheumatoid arthritis |
| M08.04 | Juvenile rheumatoid arthritis |
| M08.05 | Juvenile rheumatoid arthritis |
| M08.06 | Juvenile rheumatoid arthritis |
| M08.07 | Juvenile rheumatoid arthritis |
| M08.08 | Juvenile rheumatoid arthritis |
| M08.09 | Juvenile rheumatoid arthritis |

1. Inflammatory bowel disease
2. CPRD

| **medcode** | **readcode** | **readterm** |
| --- | --- | --- |
| 593 | J40..11 | Crohn's disease |
| 704 | J410100 | Ulcerative colitis |
| 1784 | J41..12 | Ulcerative colitis and/or proctitis |
| 1796 | J4...12 | Inflammatory bowel disease |
| 5133 | J41..00 | Idiopathic proctocolitis |
| 5749 | 14C4.11 | H/O: ulcerative colitis |
| 6538 | J401z11 | Crohn's colitis |
| 6650 | J410.00 | Ulcerative proctocolitis |
| 8347 | J410300 | Ulcerative proctitis |
| 9359 | J400z00 | Crohn's disease of the small bowel NOS |
| 11119 | ZR3S.11 | CDAI - Crohn's disease activity index |
| 11286 | J40..00 | Regional enteritis - Crohn's disease |
| 11337 | ZR3S.00 | Crohn's disease activity index |
| 12575 | N045300 | Juvenile arthritis in Crohn's disease |
| 15207 | J41z.00 | Idiopathic proctocolitis NOS |
| 15773 | J402.00 | Regional ileocolitis |
| 17641 | N031000 | Arthropathy in ulcerative colitis |
| 20480 | N031100 | Arthropathy in Crohn's disease |
| 20688 | J401z00 | Crohn's disease of the large bowel NOS |
| 22516 | J410400 | Exacerbation of ulcerative colitis |
| 24550 | J41y.00 | Other idiopathic proctocolitis |
| 24858 | J410200 | Ulcerative rectosigmoiditis |
| 28476 | J400200 | Crohn's disease of the terminal ileum |
| 29616 | J08z900 | Orofacial Crohn's disease |
| 30433 | J411.00 | Ulcerative (chronic) enterocolitis |
| 33456 | J410z00 | Ulcerative proctocolitis NOS |
| 36913 | J400500 | Exacerbation of Crohn's disease of small intestine |
| 39037 | J401200 | Exacerbation of Crohn's disease of large intestine |
| 39278 | J400400 | Crohn's disease of the ileum NOS |
| 42822 | J412.00 | Ulcerative (chronic) ileocolitis |
| 43090 | J41yz00 | Other idiopathic proctocolitis NOS |
| 44426 | J401.00 | Regional enteritis of the large bowel |
| 48732 | J410000 | Ulcerative ileocolitis |
| 51576 | J400.00 | Regional enteritis of the small bowel |
| 51578 | J40..12 | Granulomatous enteritis |
| 52449 | J40z.00 | Regional enteritis NOS |
| 53743 | Jyu4100 | [X]Other ulcerative colitis |
| 59994 | J40z.11 | Crohn's disease NOS |
| 62628 | J401000 | Regional enteritis of the colon |
| 63036 | J400100 | Regional enteritis of the jejunum |
| 64773 | J401100 | Regional enteritis of the rectum |
| 66238 | J400300 | Crohn's disease of the ileum unspecified |
| 69959 | Jyu4000 | [X]Other Crohn's disease |
| 71083 | N045400 | Juvenile arthritis in ulcerative colitis |
| 71945 | J400000 | Regional enteritis of the duodenum |
| 104259 | J413.00 | Ulcerative pancolitis |
| 107313 | 8Cc5.00 | Management of inflammatory bowel disease |
| 110283 | 8Cc5.11 | Management of IBD (inflammatory bowel disease) |

1. Hospital Episodes Statistics

| **ICD 10** | **DESCRIPTION** |
| --- | --- |
| K50 | Crohn's disease [regional enteritis] |
| K50.0 | Crohn's disease of small intestine |
| K50.1 | Crohn's disease of large intestine |
| K50.8 | Other Crohn's disease |
| K50.9 | Crohn's disease, unspecified |
| K51 | Ulcerative colitis |
| K51.0 | Ulcerative (chronic) pancolitis |
| K51.2 | Ulcerative (chronic) proctitis |
| K51.3 | Ulcerative (chronic) rectosigmoiditis |
| K51.4 | Inflammatory polyps |
| K51.5 | Left sided colitis |
| K51.8 | Other ulcerative colitis |
| K51.9 | Ulcerative colitis, unspecified |
| M07.4 | Arthropathy in Crohn's disease [regional enteritis] |
| M07.40 | Arthropathy in Crohn's disease [regional enteritis] |
| M07.41 | Arthropathy in Crohn's disease [regional enteritis] |
| M07.42 | Arthropathy in Crohn's disease [regional enteritis] |
| M07.43 | Arthropathy in Crohn's disease [regional enteritis] |
| M07.44 | Arthropathy in Crohn's disease [regional enteritis] |
| M07.45 | Arthropathy in Crohn's disease [regional enteritis] |
| M07.46 | Arthropathy in Crohn's disease [regional enteritis] |
| M07.47 | Arthropathy in Crohn's disease [regional enteritis] |
| M07.48 | Arthropathy in Crohn's disease [regional enteritis] |
| M07.49 | Arthropathy in Crohn's disease [regional enteritis] |
| M07.5 | Arthropathy in ulcerative colitis |
| M07.50 | Arthropathy in ulcerative colitis |
| M07.51 | Arthropathy in ulcerative colitis |
| M07.52 | Arthropathy in ulcerative colitis |
| M07.53 | Arthropathy in ulcerative colitis |
| M07.54 | Arthropathy in ulcerative colitis |
| M07.55 | Arthropathy in ulcerative colitis |
| M07.56 | Arthropathy in ulcerative colitis |
| M07.57 | Arthropathy in ulcerative colitis |
| M07.58 | Arthropathy in ulcerative colitis |
| M07.59 | Arthropathy in ulcerative colitis |
| M09.1 | Juvenile arthritis in Crohn's disease [regional enteritis] |
| M09.10 | Juvenile arthritis in Crohn's disease [regional enteritis] |
| M09.11 | Juvenile arthritis in Crohn's disease [regional enteritis] |
| M09.12 | Juvenile arthritis in Crohn's disease [regional enteritis] |
| M09.13 | Juvenile arthritis in Crohn's disease [regional enteritis] |
| M09.14 | Juvenile arthritis in Crohn's disease [regional enteritis] |
| M09.15 | Juvenile arthritis in Crohn's disease [regional enteritis] |
| M09.16 | Juvenile arthritis in Crohn's disease [regional enteritis] |
| M09.17 | Juvenile arthritis in Crohn's disease [regional enteritis] |
| M09.18 | Juvenile arthritis in Crohn's disease [regional enteritis] |
| M09.19 | Juvenile arthritis in Crohn's disease [regional enteritis] |
| M09.2 | Juvenile arthritis in ulcerative colitis |
| M09.20 | Juvenile arthritis in ulcerative colitis |
| M09.21 | Juvenile arthritis in ulcerative colitis |
| M09.22 | Juvenile arthritis in ulcerative colitis |
| M09.23 | Juvenile arthritis in ulcerative colitis |
| M09.24 | Juvenile arthritis in ulcerative colitis |
| M09.25 | Juvenile arthritis in ulcerative colitis |
| M09.26 | Juvenile arthritis in ulcerative colitis |
| M09.27 | Juvenile arthritis in ulcerative colitis |
| M09.28 | Juvenile arthritis in ulcerative colitis |
| M09.29 | Juvenile arthritis in ulcerative colitis |

1. Chronic obstructive pulmonary disease and asthma
2. CPRD

| medcode | readcode | readterm |
| --- | --- | --- |
| 78 | H33..00 | Asthma |
| 81 | 663..11 | Asthma monitoring |
| 185 | H333.00 | Acute exacerbation of asthma |
| 232 | H33z100 | Asthma attack |
| 233 | H33z011 | Severe asthma attack |
| 719 | 14B4.00 | H/O: asthma |
| 794 | H32..00 | Emphysema |
| 998 | H3...11 | Chronic obstructive airways disease |
| 1001 | H3...00 | Chronic obstructive pulmonary disease |
| 1208 | H330.12 | Childhood asthma |
| 1446 | H312200 | Acute exacerbation of chronic obstructive airways disease |
| 1555 | H33..11 | Bronchial asthma |
| 2290 | H330.11 | Allergic asthma |
| 3018 | 663V100 | Mild asthma |
| 3243 | H31..00 | Chronic bronchitis |
| 3366 | 663V300 | Severe asthma |
| 3458 | 663V000 | Occasional asthma |
| 3665 | H331.11 | Late onset asthma |
| 4084 | 663K.00 | Airways obstructn irreversible |
| 4442 | H33z.00 | Asthma unspecified |
| 4606 | H33zz11 | Exercise induced asthma |
| 4892 | H33z000 | Status asthmaticus NOS |
| 5267 | H331.00 | Intrinsic asthma |
| 5627 | H330011 | Hay fever with asthma |
| 5710 | H3z..00 | Chronic obstructive airways disease NOS |
| 5798 | H312000 | Chronic asthmatic bronchitis |
| 5909 | H312011 | Chronic wheezy bronchitis |
| 6707 | H330111 | Extrinsic asthma with asthma attack |
| 7058 | 8H2P.00 | Emergency admission, asthma |
| 7146 | H330.00 | Extrinsic (atopic) asthma |
| 7191 | 663P.00 | Asthma limiting activities |
| 7229 | 663W.00 | Asthma prophylactic medication used |
| 7378 | 663U.00 | Asthma management plan given |
| 7416 | 663N.00 | Asthma disturbing sleep |
| 7731 | H330.14 | Pollen asthma |
| 7884 | H3y1.00 | Chron obstruct pulmonary dis wth acute exacerbation, unspec |
| 8335 | H33z111 | Asthma attack NOS |
| 8355 | 9OJA.11 | Asthma monitored |
| 9018 | 663y.00 | Number of asthma exacerbations in past year |
| 9520 | 66YB.00 | Chronic obstructive pulmonary disease monitoring |
| 9552 | 66Y5.00 | Change in asthma management plan |
| 9663 | 66Y9.00 | Step up change in asthma management plan |
| 9876 | H38..00 | Severe chronic obstructive pulmonary disease |
| 10043 | 66YJ.00 | Asthma annual review |
| 10274 | 8B3j.00 | Asthma medication review |
| 10487 | 663j.00 | Asthma - currently active |
| 10802 | H37..00 | Moderate chronic obstructive pulmonary disease |
| 10863 | H36..00 | Mild chronic obstructive pulmonary disease |
| 10980 | H322.00 | Centrilobular emphysema |
| 10996 | 2126200 | Asthma resolved |
| 11019 | 8H2R.00 | Admit COPD emergency |
| 11022 | 178..00 | Asthma trigger |
| 11150 | H311.00 | Mucopurulent chronic bronchitis |
| 11287 | 66YM.00 | Chronic obstructive pulmonary disease annual review |
| 11370 | 1O2..00 | Asthma confirmed |
| 11839 | 212G.00 | Asthma resolved |
| 12166 | H3y..00 | Other specified chronic obstructive airways disease |
| 12987 | H33z200 | Late-onset asthma |
| 13064 | 663V.00 | Asthma severity |
| 13065 | 663V200 | Moderate asthma |
| 13066 | 663h.00 | Asthma - currently dormant |
| 13173 | 663O.00 | Asthma not disturbing sleep |
| 13174 | 663Q.00 | Asthma not limiting activities |
| 13175 | 663N200 | Asthma disturbs sleep frequently |
| 14777 | H330000 | Extrinsic asthma without status asthmaticus |
| 14798 | H312100 | Emphysematous bronchitis |
| 15157 | H31z.00 | Chronic bronchitis NOS |
| 15248 | H330.13 | Hay fever with asthma |
| 15626 | H310000 | Chronic catarrhal bronchitis |
| 16070 | H33zz00 | Asthma NOS |
| 16410 | H32yz00 | Other emphysema NOS |
| 16667 | 8795 | Asthma control step 2 |
| 16785 | 8794 | Asthma control step 1 |
| 18141 | 66YE.00 | Asthma monitoring due |
| 18223 | 66YA.00 | Step down change in asthma management plan |
| 18224 | 8796 | Asthma control step 3 |
| 18323 | H331111 | Intrinsic asthma with asthma attack |
| 18476 | 66YL.11 | COPD follow-up |
| 18501 | 66YI.00 | COPD self-management plan given |
| 18621 | 66YL.00 | Chronic obstructive pulmonary disease follow-up |
| 18792 | 9Oi..00 | Chronic obstructive pulmonary disease monitoring admin |
| 19003 | 66Ye.00 | Emergency COPD admission since last appointment |
| 19106 | 66Yd.00 | COPD accident and emergency attendance since last visit |
| 19167 | 66YQ.00 | Asthma monitoring by nurse |
| 19519 | 663p.00 | Asthma treatment compliance unsatisfactory |
| 19520 | 663n.00 | Asthma treatment compliance satisfactory |
| 19539 | 9OJA.00 | Asthma monitoring check done |
| 20860 | 8798 | Asthma control step 5 |
| 20886 | 8797 | Asthma control step 4 |
| 21061 | H3y0.00 | Chronic obstruct pulmonary dis with acute lower resp infectn |
| 21232 | H33zz12 | Allergic asthma NEC |
| 22752 | 173c.00 | Occupational asthma |
| 22905 | H581.00 | Interstitial emphysema |
| 23492 | H320z00 | Chronic bullous emphysema NOS |
| 24248 | H313.00 | Mixed simple and mucopurulent chronic bronchitis |
| 24479 | 663d.00 | Emergency asthma admission since last appointment |
| 24506 | 8791 | Further asthma - drug prevent. |
| 24884 | 663u.00 | Asthma causes daytime symptoms 1 to 2 times per week |
| 25181 | 663e.00 | Asthma restricts exercise |
| 25603 | H310.00 | Simple chronic bronchitis |
| 25791 | 8CR0.00 | Asthma clinical management plan |
| 25796 | H332.00 | Mixed asthma |
| 26018 | 66YS.00 | Chronic obstructive pulmonary disease monitoring by nurse |
| 26306 | H320.00 | Chronic bullous emphysema |
| 26501 | 663s.00 | Asthma never causes daytime symptoms |
| 26503 | 663v.00 | Asthma causes daytime symptoms most days |
| 26504 | 663f.00 | Asthma never restricts exercise |
| 26506 | 6.63E+102 | Asthma severely restricts exercise |
| 26861 | 663 | Asthma sometimes restricts exercise |
| 27819 | H312.00 | Obstructive chronic bronchitis |
| 27926 | H330100 | Extrinsic asthma with status asthmaticus |
| 28743 | 66Yf.00 | Number of COPD exacerbations in past year |
| 28755 | 9Oi0.00 | Chronic obstructive pulmonary disease monitoring 1st letter |
| 29325 | H331000 | Intrinsic asthma without status asthmaticus |
| 29645 | 8793 | Asthma control step 0 |
| 30458 | 66YR.00 | Asthma monitoring by doctor |
| 30815 | 663N000 | Asthma causing night waking |
| 31167 | 66YP.00 | Asthma night-time symptoms |
| 31225 | 663t.00 | Asthma causes daytime symptoms 1 to 2 times per month |
| 32727 | H33z.11 | Hyperreactive airways disease |
| 33450 | H32z.00 | Emphysema NOS |
| 34202 | 9Oi1.00 | Chronic obstructive pulmonary disease monitoring 2nd letter |
| 34215 | 9Oi2.00 | Chronic obstructive pulmonary disease monitoring 3rd letter |
| 37247 | H3z..11 | Chronic obstructive pulmonary disease NOS |
| 37371 | 66YD.00 | Chronic obstructive pulmonary disease monitoring due |
| 37959 | H311100 | Fetid chronic bronchitis |
| 38074 | 9Oi4.00 | Chronic obstructive pulmonary disease monitor phone invite |
| 38143 | 663O000 | Asthma never disturbs sleep |
| 38144 | 663w.00 | Asthma limits walking up hills or stairs |
| 38145 | 663x.00 | Asthma limits walking on the flat |
| 38146 | 663N100 | Asthma disturbs sleep weekly |
| 39570 | 663r.00 | Asthma causes night symptoms 1 to 2 times per month |
| 40159 | H311000 | Purulent chronic bronchitis |
| 40788 | H32y.00 | Other emphysema |
| 40823 | H334.00 | Brittle asthma |
| 40864 | U60F615 | [X] Adverse reaction to theophylline - asthma |
| 41017 | 1780 | Aspirin induced asthma |
| 41020 | 66YC.00 | Absent from work or school due to asthma |
| 42258 | 9Oi3.00 | Chronic obstructive pulmonary disease monitoring verb invite |
| 42313 | 679V.00 | Health education - chronic obstructive pulmonary disease |
| 42824 | 663q.00 | Asthma daytime symptoms |
| 44525 | H312z00 | Obstructive chronic bronchitis NOS |
| 45073 | H331z00 | Intrinsic asthma NOS |
| 45089 | H31y100 | Chronic tracheobronchitis |
| 45770 | 66Yg.00 | Chronic obstructive pulmonary disease disturbs sleep |
| 45771 | 66Yh.00 | Chronic obstructive pulmonary disease does not disturb sleep |
| 45777 | 8CR1.00 | Chronic obstructive pulmonary disease clini management plan |
| 45782 | H330z00 | Extrinsic asthma NOS |
| 45998 | 66YT.00 | Chronic obstructive pulmonary disease monitoring by doctor |
| 46036 | 66Yi.00 | Multiple COPD emergency hospital admissions |
| 46529 | 9OJ1.00 | Attends asthma monitoring |
| 46578 | H321.00 | Panlobular emphysema |
| 47337 | 663m.00 | Asthma accident and emergency attendance since last visit |
| 47684 | H47y000 | Detergent asthma |
| 47993 | 66YZ.00 | Does not have asthma management plan |
| 48591 | TJF7300 | Adverse reaction to theophylline (asthma) |
| 54893 | H582.00 | Compensatory emphysema |
| 56860 | H320000 | Segmental bullous emphysema |
| 58196 | H331100 | Intrinsic asthma with status asthmaticus |
| 59263 | H32y111 | Acute interstitial emphysema |
| 60188 | H320200 | Giant bullous emphysema |
| 61118 | H310z00 | Simple chronic bronchitis NOS |
| 61513 | H311z00 | Mucopurulent chronic bronchitis NOS |
| 63479 | H32y200 | MacLeod's unilateral emphysema |
| 64721 | H464000 | Chronic emphysema due to chemical fumes |
| 65733 | Hyu3100 | [X]Other specified chronic obstructive pulmonary disease |
| 66043 | H31y.00 | Other chronic bronchitis |
| 66058 | Hyu3000 | [X]Other emphysema |
| 67040 | H3y..11 | Other specified chronic obstructive pulmonary disease |
| 68066 | H31yz00 | Other chronic bronchitis NOS |
| 68662 | H320100 | Zonal bullous emphysema |
| 70787 | H32y100 | Atrophic (senile) emphysema |
| 73522 | 173d.00 | Work aggravated asthma |
| 92955 | H32y000 | Acute vesicular emphysema |
| 93568 | H39..00 | Very severe chronic obstructive pulmonary disease |
| 96931 | 14OX.00 | At risk of chronic obstructive pulmonary diseas exacerbation |
| 98283 | 9kf2.00 | COPD structured smoking assessment declined - enh serv admin |
| 98284 | 9kf1.00 | Refer COPD structured smoking assessment - enhanc serv admin |
| 99536 | H320300 | Bullous emphysema with collapse |
| 99793 | 8CMA000 | Patient has a written asthma personal action plan |
| 99948 | 9kf0.00 | COPD patient unsuitable for pulmonary rehab - enh serv admin |
| 100509 | 9NNX.00 | Under care of asthma specialist nurse |
| 101042 | 8BMW.00 | Issue of chronic obstructive pulmonary disease rescue pack |
| 102170 | 66Yp.00 | Asthma review using Roy Colleg of Physicians three questions |
| 102209 | 38DV.00 | Mini asthma quality of life questionnaire |
| 102301 | 1787 | Asthma trigger - seasonal |
| 102341 | 1781 | Asthma trigger - pollen |
| 102395 | 66Yr.00 | Asthma causes symptoms most nights |
| 102400 | 66Yq.00 | Asthma causes night time symptoms 1 to 2 times per week |
| 102449 | 1789 | Asthma trigger - respiratory infection |
| 102685 | 66YB000 | Chronic obstructive pulmonary disease 3 monthly review |
| 102713 | 663P000 | Asthma limits activities 1 to 2 times per month |
| 102888 | 663P100 | Asthma limits activities 1 to 2 times per week |
| 102952 | 1783 | Asthma trigger - warm air |
| 103007 | 66YB100 | Chronic obstructive pulmonary disease 6 monthly review |
| 103321 | 1786 | Asthma trigger - animals |
| 103400 | 9kf1.11 | Referred for COPD structured smoking assessment |
| 103494 | 14B3.12 | History of chronic obstructive pulmonary disease |
| 103558 | 8CeD.00 | Preferred place of care for next exacerbation of COPD |
| 103612 | 66Ys.00 | Asthma never causes night symptoms |
| 103678 | 8BMa000 | Chronic obstructiv pulmonary disease medication optimisation |
| 103758 | 8Hkw.00 | Referral to COPD community nursing team |
| 103760 | 9kf2.11 | COPD structured smoking assessment declined |
| 103813 | 1788.00 | Asthma trigger - cold air |
| 103864 | 9kf0.11 | COPD patient unsuitable for pulmonary rehabilitation |
| 103944 | 178A.00 | Asthma trigger - airborne dust |
| 103945 | 1785.00 | Asthma trigger - damp |
| 103952 | 1784.00 | Asthma trigger - emotion |
| 103955 | 1782.00 | Asthma trigger - tobacco smoke |
| 103998 | 663P200 | Asthma limits activities most days |
| 104117 | 661M300 | COPD self-management plan agreed |
| 104169 | 661N300 | COPD self-management plan review |
| 104265 | 9e03.00 | GP OOH service notified of COPD care plan |
| 104481 | 8CMV.00 | Has chronic obstructive pulmonary disease care plan |
| 104608 | H3A..00 | End stage chronic obstructive airways disease |
| 104710 | 9NgP.11 | On COPD (chr obstruc pulmonary disease) supportv cre pathway |
| 104985 | 9NgP.00 | On chronic obstructive pulmonary disease supprtv cre pathway |
| 104998 | 8I61000 | Chronic obstructve pulmonry disease rescue pack not indicatd |
| 105420 | 661N100 | Asthma self-management plan review |
| 105457 | 8CMW500 | Chronic obstructive pulmonary disease care pathway |
| 105674 | 661M100 | Asthma self-management plan agreed |
| 106637 | 9Nk7000 | Seen in chronic obstructive pulmonary disease clinic |
| 106805 | H335.00 | Chronic asthma with fixed airflow obstruction |
| 106945 | 8IEZ.00 | Chronic obstructive pulmonary disease rescue pack declined |
| 107167 | 66Yu.00 | Number days absent from school due to asthma in past 6 month |
| 107877 | 8IEy.00 | Chronic obstructive pulmon dis wr self managem plan declined |
| 108475 | 66Yz000 | Asthma management plan declined |
| 108586 | 66Yz100 | Chronic obstruct pulmonary disease management plan declined |
| 108599 | 38QM.00 | Childhood Asthma Control Test |
| 108912 | 9OJB.00 | Asthma monitorng invit SMS (short message servce) txt messge |
| 109468 | 9OJC.00 | Asthma monitoring invitation email |
| 109683 | 2126F00 | Chronic obstructive pulmonary disease resolved |
| 109774 | 66YB200 | Telehealth chronic obstructive pulmonary disease monitoring |
| 109958 | H3B..00 | Asthma-chronic obstructive pulmonary disease overlap syndrom |
| 109996 | 66Yz500 | Telehealth asthma monitoring |
| 110339 | 9OJB000 | Asthma monitoring SMS text message 1st invitation |
| 110345 | 38B8.00 | Severe asthma exacerbation risk assessment |
| 110406 | 14Ok000 | At risk of severe asthma exacerbation |
| 110533 | 9OJB100 | Asthma monitoring SMS text message 2nd invitation |

1. Hospital Episodes Statistics

| **ICD10** | **DESCRIPTION** |
| --- | --- |
| J41 | Simple and mucopurulent chronic bronchitis |
| J41.0 | Simple chronic bronchitis |
| J41.1 | Mucopurulent chronic bronchitis |
| J41.8 | Mixed simple and mucopurulent chronic bronchitis |
| J42 | Unspecified chronic bronchitis |
| J43 | Emphysema |
| J43.0 | MacLeod syndrome |
| J43.1 | Panlobular emphysema |
| J43.2 | Centrilobular emphysema |
| J43.8 | Other emphysema |
| J43.9 | Emphysema, unspecified |
| J44 | Other chronic obstructive pulmonary disease |
| J44.0 | Chronic obstructive pulmonary disease with acute lower respiratory infection |
| J44.1 | Chronic obstructive pulmonary disease with acute exacerbation, unspecified |
| J44.8 | Other specified chronic obstructive pulmonary disease |
| J44.9 | Chronic obstructive pulmonary disease, unspecified |
| J45 | Asthma |
| J45.0 | Predominantly allergic asthma |
| J45.1 | Nonallergic asthma |
| J45.8 | Mixed asthma |
| J45.9 | Asthma, unspecified |
| J46 | Status asthmaticus |

1. Diabetes
2. CPRD

| medcode | readcode | readterm |
| --- | --- | --- |
| 506 | C100112 | Non-insulin dependent diabetes mellitus |
| 711 | C10..00 | Diabetes mellitus |
| 758 | C10F.00 | Type 2 diabetes mellitus |
| 1038 | C100011 | Insulin dependent diabetes mellitus |
| 1323 | F420.00 | Diabetic retinopathy |
| 1407 | C10FJ00 | Insulin treated Type 2 diabetes mellitus |
| 1549 | C10E.00 | Type 1 diabetes mellitus |
| 1647 | C108.00 | Insulin dependent diabetes mellitus |
| 1682 | C101.00 | Diabetes mellitus with ketoacidosis |
| 1684 | 66A4.00 | Diabetic on oral treatment |
| 2340 | F381311 | Diabetic amyotrophy |
| 2342 | F372.12 | Diabetic neuropathy |
| 2378 | 66AJ.00 | Diabetic - poor control |
| 2471 | K01x100 | Nephrotic syndrome in diabetes mellitus |
| 2475 | C104.11 | Diabetic nephropathy |
| 2478 | 66AJ100 | Brittle diabetes |
| 2986 | F420200 | Preproliferative diabetic retinopathy |
| 3286 | F420100 | Proliferative diabetic retinopathy |
| 3837 | F420400 | Diabetic maculopathy |
| 4513 | C109.00 | Non-insulin dependent diabetes mellitus |
| 5002 | F372.11 | Diabetic polyneuropathy |
| 5884 | C109.11 | NIDDM - Non-insulin dependent diabetes mellitus |
| 6125 | 66AS.00 | Diabetic annual review |
| 6430 | 9NM0.00 | Attending diabetes clinic |
| 6509 | C108700 | Insulin dependent diabetes mellitus with retinopathy |
| 6791 | C108800 | Insulin dependant diabetes mellitus - poor control |
| 7045 | 14F4.00 | H/O: Admission in last year for diabetes foot problem |
| 7059 | 8H2J.00 | Admit diabetic emergency |
| 7069 | F420000 | Background diabetic retinopathy |
| 7328 | M037200 | Cellulitis in diabetic foot |
| 7563 | 66A3.00 | Diabetic on diet only |
| 7795 | C106.12 | Diabetes mellitus with neuropathy |
| 8403 | C109700 | Non-insulin dependant diabetes mellitus - poor control |
| 8836 | 66AR.00 | Diabetes management plan given |
| 8842 | 66A5.00 | Diabetic on insulin |
| 9013 | 66AJ.11 | Unstable diabetes |
| 9835 | 2BBL.00 | O/E - diabetic maculopathy present both eyes |
| 9881 | M271200 | Mixed diabetic ulcer - foot |
| 9958 | 42W..00 | Hb. A1C - diabetic control |
| 9974 | 9N1v.00 | Seen in diabetic eye clinic |
| 10098 | C10yy00 | Other specified diabetes mellitus with other spec comps |
| 10099 | F420300 | Advanced diabetic maculopathy |
| 10418 | C10ED00 | Type 1 diabetes mellitus with nephropathy |
| 10642 | ZC2C800 | Dietary advice for diabetes mellitus |
| 10659 | F464000 | Diabetic cataract |
| 10692 | C10EM00 | Type 1 diabetes mellitus with ketoacidosis |
| 10755 | F420600 | Non proliferative diabetic retinopathy |
| 10824 | 9N1i.00 | Seen in diabetic foot clinic |
| 10977 | 66Ac.00 | Diabetic peripheral neuropathy screening |
| 11018 | 8HBG.00 | Diabetic retinopathy 12 month review |
| 11047 | 66AH000 | Conversion to insulin |
| 11094 | 9NND.00 | Under care of diabetic foot screener |
| 11129 | 2BBQ.00 | O/E - left eye background diabetic retinopathy |
| 11433 | 2BBP.00 | O/E - right eye background diabetic retinopathy |
| 11471 | 8B3l.00 | Diabetes medication review |
| 11599 | 7276 | Pan retinal photocoagulation for diabetes |
| 11626 | F420z00 | Diabetic retinopathy NOS |
| 11663 | M271100 | Neuropathic diabetic ulcer - foot |
| 11677 | 8H7r.00 | Refer to diabetic foot screener |
| 12213 | 8BL2.00 | Patient on maximal tolerated therapy for diabetes |
| 12247 | 8I6G.00 | Diabetic foot examination not indicated |
| 12262 | 8I3X.00 | Diabetic retinopathy screening refused |
| 12307 | 66AU.00 | Diabetes care by hospital only |
| 12455 | C10E.11 | Type I diabetes mellitus |
| 12506 | 66AP.00 | Diabetes: practice programme |
| 12640 | C10FC00 | Type 2 diabetes mellitus with nephropathy |
| 12675 | 66AQ.00 | Diabetes: shared care programme |
| 12736 | C10F500 | Type 2 diabetes mellitus with gangrene |
| 13071 | 66AI.00 | Diabetic - good control |
| 13078 | 13AC.00 | Diabetic weight reducing diet |
| 13097 | 2BBT.00 | O/E - right eye proliferative diabetic retinopathy |
| 13099 | 2BBR.00 | O/E - right eye preproliferative diabetic retinopathy |
| 13101 | 2BBV.00 | O/E - left eye proliferative diabetic retinopathy |
| 13102 | 2BBW.00 | O/E - right eye diabetic maculopathy |
| 13103 | 2BBS.00 | O/E - left eye preproliferative diabetic retinopathy |
| 13108 | 2BBX.00 | O/E - left eye diabetic maculopathy |
| 13279 | C104y00 | Other specified diabetes mellitus with renal complications |
| 14049 | 42WZ.00 | Hb. A1C - diabetic control NOS |
| 14050 | 42c..00 | HbA1 - diabetic control |
| 14803 | C100100 | Diabetes mellitus, adult onset, no mention of complication |
| 14889 | C100111 | Maturity onset diabetes |
| 15690 | C103.00 | Diabetes mellitus with ketoacidotic coma |
| 16230 | C106.00 | Diabetes mellitus with neurological manifestation |
| 16490 | 66AH.00 | Diabetic treatment changed |
| 16491 | C106.13 | Diabetes mellitus with polyneuropathy |
| 16502 | C104.00 | Diabetes mellitus with renal manifestation |
| 16881 | ZV65312 | [V]Dietary counselling in diabetes mellitus |
| 17067 | F171100 | Autonomic neuropathy due to diabetes |
| 17095 | 2G5A.00 | O/E - Right diabetic foot at risk |
| 17236 | 14P3.00 | H/O: insulin therapy |
| 17247 | F35z000 | Diabetic mononeuritis NOS |
| 17262 | C109600 | Non-insulin-dependent diabetes mellitus with retinopathy |
| 17313 | F440700 | Diabetic iritis |
| 17545 | C108F11 | Type I diabetes mellitus with diabetic cataract |
| 17817 | 7L19800 | Subcutaneous injection of insulin |
| 17858 | C108.12 | Type 1 diabetes mellitus |
| 17859 | C109.12 | Type 2 diabetes mellitus |
| 17869 | 66AL.00 | Diabetic-uncooperative patient |
| 17886 | 66AM.00 | Diabetic - follow-up default |
| 18056 | 2G5C.00 | Foot abnormality - diabetes related |
| 18142 | N030000 | Diabetic cheiroarthropathy |
| 18143 | C109G11 | Type II diabetes mellitus with arthropathy |
| 18167 | 66AT.00 | Annual diabetic blood test |
| 18209 | C109012 | Type 2 diabetes mellitus with renal complications |
| 18219 | C109.13 | Type II diabetes mellitus |
| 18230 | C108J12 | Type 1 diabetes mellitus with neuropathic arthropathy |
| 18264 | C109J12 | Insulin treated Type II diabetes mellitus |
| 18278 | C109J00 | Insulin treated Type 2 diabetes mellitus |
| 18311 | 68A7.00 | Diabetic retinopathy screening |
| 18387 | C10E700 | Type 1 diabetes mellitus with retinopathy |
| 18390 | C10FM00 | Type 2 diabetes mellitus with persistent microalbuminuria |
| 18425 | C10FB00 | Type 2 diabetes mellitus with polyneuropathy |
| 18496 | C10F600 | Type 2 diabetes mellitus with retinopathy |
| 18505 | C108.11 | IDDM-Insulin dependent diabetes mellitus |
| 18642 | C10EH00 | Type 1 diabetes mellitus with arthropathy |
| 18662 | 8HBH.00 | Diabetic retinopathy 6 month review |
| 18683 | C10E500 | Type 1 diabetes mellitus with ulcer |
| 18747 | 8I6F.00 | Diabetic retinopathy screening not indicated |
| 18777 | C10F000 | Type 2 diabetes mellitus with renal complications |
| 18824 | 8I3W.00 | Diabetic foot examination declined |
| 19381 | 8HTk.00 | Referral to diabetic eye clinic |
| 19739 | 68A9.00 | Diabetic retinopathy screening offered |
| 20696 | 66AA.11 | Injection sites - diabetic |
| 21482 | C102.00 | Diabetes mellitus with hyperosmolar coma |
| 21983 | C108012 | Type 1 diabetes mellitus with renal complications |
| 22023 | 66AJz00 | Diabetic - poor control NOS |
| 22573 | C106z00 | Diabetes mellitus NOS with neurological manifestation |
| 22823 | 66Ab.00 | Diabetic foot examination |
| 22871 | C10EP00 | Type 1 diabetes mellitus with exudative maculopathy |
| 22884 | C10F.11 | Type II diabetes mellitus |
| 22967 | 2BBF.00 | Retinal abnormality - diabetes related |
| 23479 | C350011 | Bronzed diabetes |
| 24327 | M271000 | Ischaemic ulcer diabetic foot |
| 24363 | 8A13.00 | Diabetic stabilisation |
| 24423 | C108.13 | Type I diabetes mellitus |
| 24458 | C109711 | Type II diabetes mellitus - poor control |
| 24490 | C100000 | Diabetes mellitus, juvenile type, no mention of complication |
| 24571 | F372200 | Asymptomatic diabetic neuropathy |
| 24693 | C109G00 | Non-insulin dependent diabetes mellitus with arthropathy |
| 24694 | C108B00 | Insulin dependent diabetes mellitus with mononeuropathy |
| 24836 | C109C12 | Type 2 diabetes mellitus with nephropathy |
| 25041 | ZC2CA00 | Dietary advice for type II diabetes |
| 25591 | C10FQ00 | Type 2 diabetes mellitus with exudative maculopathy |
| 25627 | C10F700 | Type 2 diabetes mellitus - poor control |
| 26054 | C10FL00 | Type 2 diabetes mellitus with persistent proteinuria |
| 26664 | 2G5B.00 | O/E - Left diabetic foot at risk |
| 26666 | 2G5E.00 | O/E - Right diabetic foot at low risk |
| 26667 | 2G5I.00 | O/E - Left diabetic foot at low risk |
| 26855 | C108400 | Unstable insulin dependant diabetes mellitus |
| 27891 | N030100 | Diabetic Charcot arthropathy |
| 27921 | 2G51000 | Foot abnormality - diabetes related |
| 28769 | 66AV.00 | Diabetic on insulin and oral treatment |
| 28856 | 8CP2.00 | Transition of diabetes care options discussed |
| 28873 | 66Ai.00 | Diabetic 6 month review |
| 29041 | 66AN.00 | Date diabetic treatment start |
| 29979 | C109900 | Non-insulin-dependent diabetes mellitus without complication |
| 30247 | TJ23000 | Adverse reaction to insulins |
| 30294 | C10EL00 | Type 1 diabetes mellitus with persistent microalbuminuria |
| 30323 | C10EK00 | Type 1 diabetes mellitus with persistent proteinuria |
| 30477 | F420700 | High risk proliferative diabetic retinopathy |
| 30648 | 9N4p.00 | Did not attend diabetic retinopathy clinic |
| 31053 | R054300 | [D]Widespread diabetic foot gangrene |
| 31156 | 2G5J.00 | O/E - Left diabetic foot at moderate risk |
| 31157 | 2G5F.00 | O/E - Right diabetic foot at moderate risk |
| 31171 | 2G5G.00 | O/E - Right diabetic foot at high risk |
| 31172 | 2G5K.00 | O/E - Left diabetic foot at high risk |
| 31310 | C108900 | Insulin dependant diabetes maturity onset |
| 31790 | F372.00 | Polyneuropathy in diabetes |
| 32359 | ZRbH.00 | Perceived control of insulin-dependent diabetes |
| 32403 | C107.11 | Diabetes mellitus with gangrene |
| 32556 | C107.12 | Diabetes with gangrene |
| 32627 | C10FN00 | Type 2 diabetes mellitus with ketoacidosis |
| 33254 | C105.00 | Diabetes mellitus with ophthalmic manifestation |
| 33343 | C10y.00 | Diabetes mellitus with other specified manifestation |
| 33807 | C107200 | Diabetes mellitus, adult with gangrene |
| 34152 | G73y000 | Diabetic peripheral angiopathy |
| 34268 | C10F200 | Type 2 diabetes mellitus with neurological complications |
| 34283 | C105z00 | Diabetes mellitus NOS with ophthalmic manifestation |
| 34450 | C10FK00 | Hyperosmolar non-ketotic state in type 2 diabetes mellitus |
| 34528 | 3882 | Diabetes well being questionnaire |
| 34912 | C109400 | Non-insulin dependent diabetes mellitus with ulcer |
| 35105 | C104100 | Diabetes mellitus, adult onset, with renal manifestation |
| 35107 | C104z00 | Diabetes mellitis with nephropathy NOS |
| 35116 | 2G5L.00 | O/E - Left diabetic foot - ulcerated |
| 35288 | C10E800 | Type 1 diabetes mellitus - poor control |
| 35316 | 2G5H.00 | O/E - Right diabetic foot - ulcerated |
| 35321 | 8H3O.00 | Non-urgent diabetic admission |
| 35383 | 9OLD.00 | Diabetic patient unsuitable for digital retinal photography |
| 35385 | C10FH00 | Type 2 diabetes mellitus with neuropathic arthropathy |
| 35399 | C107.00 | Diabetes mellitus with peripheral circulatory disorder |
| 35785 | F372100 | Chronic painful diabetic neuropathy |
| 36633 | C109K00 | Hyperosmolar non-ketotic state in type 2 diabetes mellitus |
| 36695 | C10D.00 | Diabetes mellitus autosomal dominant type 2 |
| 36798 | 7L10000 | Continuous subcutaneous infusion of insulin |
| 37315 | F3y0.00 | Diabetic mononeuropathy |
| 37648 | C109J11 | Insulin treated non-insulin dependent diabetes mellitus |
| 37806 | C10FF00 | Type 2 diabetes mellitus with peripheral angiopathy |
| 38076 | M21yC00 | Insulin lipohypertrophy |
| 38130 | ZRB6.00 | Diabetes wellbeing questionnaire |
| 38161 | C108711 | Type I diabetes mellitus with retinopathy |
| 38617 | C101y00 | Other specified diabetes mellitus with ketoacidosis |
| 38986 | C100.00 | Diabetes mellitus with no mention of complication |
| 39070 | C10EE00 | Type 1 diabetes mellitus with hypoglycaemic coma |
| 39317 | C106100 | Diabetes mellitus, adult onset, + neurological manifestation |
| 39420 | F381300 | Myasthenic syndrome due to diabetic amyotrophy |
| 39809 | C108J00 | Insulin dependent diab mell with neuropathic arthropathy |
| 40023 | C102000 | Diabetes mellitus, juvenile type, with hyperosmolar coma |
| 40401 | C109500 | Non-insulin dependent diabetes mellitus with gangrene |
| 40682 | C10E900 | Type 1 diabetes mellitus maturity onset |
| 40837 | C10EN00 | Type 1 diabetes mellitus with ketoacidotic coma |
| 40962 | C109H00 | Non-insulin dependent d m with neuropathic arthropathy |
| 41049 | C108712 | Type 1 diabetes mellitus with retinopathy |
| 41389 | C105100 | Diabetes mellitus, adult onset, + ophthalmic manifestation |
| 41686 | Cyu2000 | [X]Other specified diabetes mellitus |
| 41716 | C108C00 | Insulin dependent diabetes mellitus with polyneuropathy |
| 42505 | C101z00 | Diabetes mellitus NOS with ketoacidosis |
| 42567 | C103000 | Diabetes mellitus, juvenile type, with ketoacidotic coma |
| 42729 | C108E11 | Type I diabetes mellitus with hypoglycaemic coma |
| 42762 | C109612 | Type 2 diabetes mellitus with retinopathy |
| 42831 | C10E200 | Type 1 diabetes mellitus with neurological complications |
| 43139 | C102100 | Diabetes mellitus, adult onset, with hyperosmolar coma |
| 43227 | C10F311 | Type II diabetes mellitus with multiple complications |
| 43453 | C10C.00 | Diabetes mellitus autosomal dominant |
| 43493 | M21yC11 | Insulin site lipohypertrophy |
| 43785 | C109D00 | Non-insulin dependent diabetes mellitus with hypoglyca coma |
| 43857 | C10M.00 | Lipoatrophic diabetes mellitus |
| 43921 | C10E400 | Unstable type 1 diabetes mellitus |
| 43951 | 66AK.00 | Diabetic - cooperative patient |
| 44033 | F345000 | Diabetic mononeuritis multiplex |
| 44260 | C108F00 | Insulin dependent diabetes mellitus with diabetic cataract |
| 44312 | 9M10.00 | Informed dissent for diabetes national audit |
| 44440 | C108E00 | Insulin dependent diabetes mellitus with hypoglycaemic coma |
| 44443 | C108500 | Insulin dependent diabetes mellitus with ulcer |
| 44779 | C109E12 | Type 2 diabetes mellitus with diabetic cataract |
| 44982 | C10FE00 | Type 2 diabetes mellitus with diabetic cataract |
| 45276 | C10E312 | Insulin dependent diabetes mellitus with multiple complicat |
| 45467 | C109B00 | Non-insulin dependent diabetes mellitus with polyneuropathy |
| 45491 | C10z.00 | Diabetes mellitus with unspecified complication |
| 45499 | K01x111 | Kimmelstiel - Wilson disease |
| 45913 | C109712 | Type 2 diabetes mellitus - poor control |
| 45914 | C108812 | Type 1 diabetes mellitus - poor control |
| 45919 | C109212 | Type 2 diabetes mellitus with neurological complications |
| 46150 | C109512 | Type 2 diabetes mellitus with gangrene |
| 46290 | C108y00 | Other specified diabetes mellitus with multiple comps |
| 46301 | C10EC00 | Type 1 diabetes mellitus with polyneuropathy |
| 46624 | C10C.11 | Maturity onset diabetes in youth |
| 46850 | C108811 | Type I diabetes mellitus - poor control |
| 46917 | C10FD00 | Type 2 diabetes mellitus with hypoglycaemic coma |
| 46963 | C108000 | Insulin-dependent diabetes mellitus with renal complications |
| 47032 | 8CS0.00 | Diabetes care plan agreed |
| 47058 | 8Hg4.00 | Discharged from care of diabetes specialist nurse |
| 47315 | C10F711 | Type II diabetes mellitus - poor control |
| 47321 | C10F100 | Type 2 diabetes mellitus with ophthalmic complications |
| 47328 | 2BBk.00 | O/E - right eye stable treated prolif diabetic retinopathy |
| 47341 | 8A12.00 | Diabetic crisis monitoring |
| 47370 | 8HLE.00 | Diabetology D.V. done |
| 47377 | C105y00 | Other specified diabetes mellitus with ophthalmic complicatn |
| 47409 | C109B11 | Type II diabetes mellitus with polyneuropathy |
| 47582 | C10E000 | Type 1 diabetes mellitus with renal complications |
| 47584 | F420500 | Advanced diabetic retinal disease |
| 47649 | C10E100 | Type 1 diabetes mellitus with ophthalmic complications |
| 47650 | C10E300 | Type 1 diabetes mellitus with multiple complications |
| 47816 | C109H11 | Type II diabetes mellitus with neuropathic arthropathy |
| 47954 | C10F900 | Type 2 diabetes mellitus without complication |
| 48078 | F372000 | Acute painful diabetic neuropathy |
| 48192 | C109E11 | Type II diabetes mellitus with diabetic cataract |
| 48310 | ZV6DA00 | [V]Admitted for commencement of insulin |
| 49074 | C10F400 | Type 2 diabetes mellitus with ulcer |
| 49146 | C108211 | Type I diabetes mellitus with neurological complications |
| 49276 | C108100 | Insulin-dependent diabetes mellitus with ophthalmic comps |
| 49554 | C10EF00 | Type 1 diabetes mellitus with diabetic cataract |
| 49640 | 2G5W.00 | O/E - left chronic diabetic foot ulcer |
| 49655 | C10F611 | Type II diabetes mellitus with retinopathy |
| 49869 | C109G12 | Type 2 diabetes mellitus with arthropathy |
| 49884 | 6761 | Diabetic pre-pregnancy counselling |
| 49949 | C10E411 | Unstable type I diabetes mellitus |
| 50175 | 66AW.00 | Diabetic foot risk assessment |
| 50225 | C109011 | Type II diabetes mellitus with renal complications |
| 50429 | C109100 | Non-insulin-dependent diabetes mellitus with ophthalm comps |
| 50527 | C10FB11 | Type II diabetes mellitus with polyneuropathy |
| 50609 | L180600 | Pre-existing diabetes mellitus, non-insulin-dependent |
| 50813 | C109A11 | Type II diabetes mellitus with mononeuropathy |
| 50960 | L180500 | Pre-existing diabetes mellitus, insulin-dependent |
| 50972 | C100z00 | Diabetes mellitus NOS with no mention of complication |
| 51261 | C10E.12 | Insulin dependent diabetes mellitus |
| 51697 | C10G.00 | Secondary pancreatic diabetes mellitus |
| 51756 | C10FP00 | Type 2 diabetes mellitus with ketoacidotic coma |
| 51939 | ZV6DB00 | [V]Admitted for conversion to insulin |
| 51957 | C108511 | Type I diabetes mellitus with ulcer |
| 52041 | 2BBl.00 | O/E - left eye stable treated prolif diabetic retinopathy |
| 52104 | C108300 | Insulin dependent diabetes mellitus with multiple complicatn |
| 52212 | Cyu2.00 | [X]Diabetes mellitus |
| 52237 | 9360 | Patient held diabetic record issued |
| 52283 | C108200 | Insulin-dependent diabetes mellitus with neurological comps |
| 52303 | C109000 | Non-insulin-dependent diabetes mellitus with renal comps |
| 52630 | 2BBo.00 | O/E - sight threatening diabetic retinopathy |
| 53200 | C101000 | Diabetes mellitus, juvenile type, with ketoacidosis |
| 53238 | 66AG.00 | Diabetic drug side effects |
| 53392 | C10F911 | Type II diabetes mellitus without complication |
| 53630 | C110.11 | Insulin coma |
| 53634 | R054200 | [D]Gangrene of toe in diabetic |
| 54008 | C10EJ00 | Type 1 diabetes mellitus with neuropathic arthropathy |
| 54212 | C109F00 | Non-insulin-dependent d m with peripheral angiopath |
| 54600 | C10E412 | Unstable insulin dependent diabetes mellitus |
| 54601 | 9NN8.00 | Under care of diabetologist |
| 54856 | C101100 | Diabetes mellitus, adult onset, with ketoacidosis |
| 54899 | C109F11 | Type II diabetes mellitus with peripheral angiopathy |
| 55075 | C109411 | Type II diabetes mellitus with ulcer |
| 55239 | C10EQ00 | Type 1 diabetes mellitus with gastroparesis |
| 55431 | L180X00 | Pre-existing diabetes mellitus, unspecified |
| 55842 | C109200 | Non-insulin-dependent diabetes mellitus with neuro comps |
| 56268 | C109D11 | Type II diabetes mellitus with hypoglycaemic coma |
| 56448 | C108A00 | Insulin-dependent diabetes without complication |
| 57278 | C10F011 | Type II diabetes mellitus with renal complications |
| 57333 | N030011 | Diabetic cheiropathy |
| 57389 | 93C4.00 | Patient consent given for addition to diabetic register |
| 57621 | C108D00 | Insulin dependent diabetes mellitus with nephropathy |
| 57723 | 8HHy.00 | Referral to diabetic register |
| 58133 | ZLD7500 | Discharge by diabetic liaison nurse |
| 58159 | 8I3k.00 | Insulin therapy declined |
| 58604 | C109611 | Type II diabetes mellitus with retinopathy |
| 59253 | C10FG00 | Type 2 diabetes mellitus with arthropathy |
| 59288 | C103y00 | Other specified diabetes mellitus with coma |
| 59365 | C109C00 | Non-insulin dependent diabetes mellitus with nephropathy |
| 59725 | C109111 | Type II diabetes mellitus with ophthalmic complications |
| 59903 | C106.11 | Diabetic amyotrophy |
| 59991 | C10D.11 | Maturity onset diabetes in youth type 2 |
| 60107 | C108411 | Unstable type I diabetes mellitus |
| 60208 | C108J11 | Type I diabetes mellitus with neuropathic arthropathy |
| 60499 | C108600 | Insulin dependent diabetes mellitus with gangrene |
| 60699 | C109F12 | Type 2 diabetes mellitus with peripheral angiopathy |
| 60796 | C10FL11 | Type II diabetes mellitus with persistent proteinuria |
| 61021 | 68AB.00 | Diabetic digital retinopathy screening offered |
| 61071 | C109D12 | Type 2 diabetes mellitus with hypoglycaemic coma |
| 61210 | TJ23z00 | Adverse reaction to insulins and antidiabetic agents NOS |
| 61344 | C108011 | Type I diabetes mellitus with renal complications |
| 61461 | 9M00.00 | Informed consent for diabetes national audit |
| 61520 | C110000 | Iatrogenic hyperinsulinism |
| 61523 | C106y00 | Other specified diabetes mellitus with neurological comps |
| 61557 | 8HKE.00 | Diabetology D.V. requested |
| 61670 | 889A.00 | Diab mellit insulin-glucose infus acute myocardial infarct |
| 61829 | C108212 | Type 1 diabetes mellitus with neurological complications |
| 62107 | C109511 | Type II diabetes mellitus with gangrene |
| 62146 | C109300 | Non-insulin-dependent diabetes mellitus with multiple comps |
| 62209 | C10EM11 | Type I diabetes mellitus with ketoacidosis |
| 62352 | C108H11 | Type I diabetes mellitus with arthropathy |
| 62384 | 2G5V.00 | O/E - right chronic diabetic foot ulcer |
| 62613 | C10EA11 | Type I diabetes mellitus without complication |
| 62674 | C10FA00 | Type 2 diabetes mellitus with mononeuropathy |
| 63017 | C108911 | Type I diabetes mellitus maturity onset |
| 63357 | C107100 | Diabetes mellitus, adult, + peripheral circulatory disorder |
| 63364 | U602312 | [X] Adverse reaction to insulins |
| 63371 | C10y100 | Diabetes mellitus, adult, + other specified manifestation |
| 63412 | 8CR2.00 | Diabetes clinical management plan |
| 63690 | C10FR00 | Type 2 diabetes mellitus with gastroparesis |
| 63762 | C10z100 | Diabetes mellitus, adult onset, + unspecified complication |
| 64142 | 8Hl1.00 | Referral for diabetic retinopathy screening |
| 64283 | C10zy00 | Other specified diabetes mellitus with unspecified comps |
| 64357 | C10zz00 | Diabetes mellitus NOS with unspecified complication |
| 64446 | C108G00 | Insulin dependent diab mell with peripheral angiopathy |
| 64449 | C108z00 | Unspecified diabetes mellitus with multiple complications |
| 64571 | C109C11 | Type II diabetes mellitus with nephropathy |
| 64668 | C10FJ11 | Insulin treated Type II diabetes mellitus |
| 65025 | C107z00 | Diabetes mellitus NOS with peripheral circulatory disorder |
| 65062 | C103z00 | Diabetes mellitus NOS with ketoacidotic coma |
| 65267 | C10F300 | Type 2 diabetes mellitus with multiple complications |
| 65463 | F420800 | High risk non proliferative diabetic retinopathy |
| 65616 | C108H00 | Insulin dependent diabetes mellitus with arthropathy |
| 65684 | U602311 | [X] Adverse reaction to insulins and antidiabetic agents |
| 65704 | C109412 | Type 2 diabetes mellitus with ulcer |
| 66145 | C10EN11 | Type I diabetes mellitus with ketoacidotic coma |
| 66274 | 66Ah.00 | Insulin needles changed for each injection |
| 66872 | C108D11 | Type I diabetes mellitus with nephropathy |
| 66965 | C109H12 | Type 2 diabetes mellitus with neuropathic arthropathy |
| 67853 | C106000 | Diabetes mellitus, juvenile, + neurological manifestation |
| 67905 | C109211 | Type II diabetes mellitus with neurological complications |
| 68105 | C10EB00 | Type 1 diabetes mellitus with mononeuropathy |
| 68390 | C108512 | Type 1 diabetes mellitus with ulcer |
| 68546 | ZRB4.00 | Diabetes clinic satisfaction questionnaire |
| 68792 | C10z000 | Diabetes mellitus, juvenile type, + unspecified complication |
| 68818 | ZRB5.11 | DTSQ - Diabetes treatment satisfaction questionnaire |
| 68843 | C103100 | Diabetes mellitus, adult onset, with ketoacidotic coma |
| 68928 | TJ23.00 | Adverse reaction to insulins and antidiabetic agents |
| 69043 | ZC2C900 | Dietary advice for type I diabetes |
| 69152 | 66Aj.00 | Insulin needles changed less than once a day |
| 69278 | C109E00 | Non-insulin depend diabetes mellitus with diabetic cataract |
| 69676 | C10EA00 | Type 1 diabetes mellitus without complication |
| 69748 | C105000 | Diabetes mellitus, juvenile type, + ophthalmic manifestation |
| 69993 | C10E600 | Type 1 diabetes mellitus with gangrene |
| 70316 | C109112 | Type 2 diabetes mellitus with ophthalmic complications |
| 70448 | C107000 | Diabetes mellitus, juvenile +peripheral circulatory disorder |
| 70766 | C108E12 | Type 1 diabetes mellitus with hypoglycaemic coma |
| 70821 | C10yz00 | Diabetes mellitus NOS with other specified manifestation |
| 72320 | C109A00 | Non-insulin dependent diabetes mellitus with mononeuropathy |
| 72333 | 8HME.00 | Listed for Diabetology admissn |
| 72345 | C102z00 | Diabetes mellitus NOS with hyperosmolar coma |
| 72702 | C10E812 | Insulin dependent diabetes mellitus - poor control |
| 83485 | 66Am.00 | Insulin dose changed |
| 83532 | 66Ao.00 | Diabetes type 2 review |
| 85660 | 66An.00 | Diabetes type 1 review |
| 85991 | C10FM11 | Type II diabetes mellitus with persistent microalbuminuria |
| 90301 | 66Ag.00 | Insulin needles changed daily |
| 91164 | ZRB4.11 | CSQ - Diabetes clinic satisfaction questionnaire |
| 91646 | C10F411 | Type II diabetes mellitus with ulcer |
| 91942 | C10E311 | Type I diabetes mellitus with multiple complications |
| 91943 | C10EC11 | Type I diabetes mellitus with polyneuropathy |
| 93380 | C10N100 | Cystic fibrosis related diabetes mellitus |
| 93390 | 9OLH.00 | Attended DAFNE diabetes structured education programme |
| 93468 | C10EG00 | Type 1 diabetes mellitus with peripheral angiopathy |
| 93491 | 9OLJ.00 | DAFNE diabetes structured education programme completed |
| 93631 | 9OLL.00 | XPERT diabetes structured education programme completed |
| 93704 | 8Hj3.00 | Referral to DAFNE diabetes structured education programme |
| 93727 | C10FE11 | Type II diabetes mellitus with diabetic cataract |
| 93870 | 8Hj5.00 | Referral to XPERT diabetes structured education programme |
| 93875 | C10E712 | Insulin dependent diabetes mellitus with retinopathy |
| 93878 | C10E511 | Type I diabetes mellitus with ulcer |
| 93922 | C104000 | Diabetes mellitus, juvenile type, with renal manifestation |
| 94011 | 9OLG.00 | Attended XPERT diabetes structured education programme |
| 94699 | ZRB5.00 | Diabetes treatment satisfaction questionnaire |
| 94955 | 9NiE.00 | Did not attend XPERT diabetes structured education programme |
| 94956 | 8I84.00 | Did not complete XPERT diabetes structured education program |
| 95343 | C10E711 | Type I diabetes mellitus with retinopathy |
| 95351 | C10FA11 | Type II diabetes mellitus with mononeuropathy |
| 95636 | C10ER00 | Latent autoimmune diabetes mellitus in adult |
| 95992 | C108A11 | Type I diabetes mellitus without complication |
| 95994 | 66Aq.00 | Diabetic foot screen |
| 96010 | 66Ap.00 | Insulin treatment initiated |
| 96143 | 9kL..00 | Insulin initiation - enhanced services administration |
| 96235 | C10E911 | Type I diabetes mellitus maturity onset |
| 96506 | C10G000 | Secondary pancreatic diabetes mellitus without complication |
| 97446 | C108912 | Type 1 diabetes mellitus maturity onset |
| 97474 | C108412 | Unstable type 1 diabetes mellitus |
| 97809 | 8I82.00 | Did not complete DAFNE diabetes structured education program |
| 97824 | ZRB6.11 | DWBQ - Diabetes wellbeing questionnaire |
| 97849 | C10E912 | Insulin dependent diabetes maturity onset |
| 97894 | C10EP11 | Type I diabetes mellitus with exudative maculopathy |
| 98071 | C10E112 | Insulin-dependent diabetes mellitus with ophthalmic comps |
| 98392 | C10C.12 | Maturity onset diabetes in youth type 1 |
| 98616 | C10F211 | Type II diabetes mellitus with neurological complications |
| 98704 | C10E512 | Insulin dependent diabetes mellitus with ulcer |
| 98723 | C10FD11 | Type II diabetes mellitus with hypoglycaemic coma |
| 98954 | 3883 | Diabetes treatment satisfaction questionnaire |
| 99231 | C108B11 | Type I diabetes mellitus with mononeuropathy |
| 99277 | 9NiC.00 | Did not attend DAFNE diabetes structured education programme |
| 99311 | C10E111 | Type I diabetes mellitus with ophthalmic complications |
| 99628 | Kyu0300 | [X]Glomerular disorders in diabetes mellitus |
| 99716 | C10EE12 | Insulin dependent diabetes mellitus with hypoglycaemic coma |
| 99719 | C10EA12 | Insulin-dependent diabetes without complication |
| 100033 | U60231E | [X] Adverse reaction to insulins and antidiabetic agents NOS |
| 100292 | Cyu2300 | [X]Unspecified diabetes mellitus with renal complications |
| 100770 | C10EF12 | Insulin dependent diabetes mellitus with diabetic cataract |
| 100964 | C10F111 | Type II diabetes mellitus with ophthalmic complications |
| 101311 | C10EC12 | Insulin dependent diabetes mellitus with polyneuropathy |
| 101735 | C10E212 | Insulin-dependent diabetes mellitus with neurological comps |
| 101881 | 2BBr.00 | Impaired vision due to diabetic retinopathy |
| 102112 | C10E611 | Type I diabetes mellitus with gangrene |
| 102163 | C10ED12 | Insulin dependent diabetes mellitus with nephropathy |
| 102201 | C10FC11 | Type II diabetes mellitus with nephropathy |
| 102434 | 66Au.00 | Diabetic erectile dysfunction review |
| 102490 | 66Av.00 | Diabetic assessment of erectile dysfunction |
| 102611 | 66At111 | Type 2 diabetic dietary review |
| 102620 | C10EL11 | Type I diabetes mellitus with persistent microalbuminuria |
| 102704 | 66At000 | Type I diabetic dietary review |
| 102740 | C108112 | Type 1 diabetes mellitus with ophthalmic complications |
| 102768 | 9NiZ.00 | Did not attend diabetes foot screening |
| 102946 | C10E012 | Insulin-dependent diabetes mellitus with renal complications |
| 103743 | 8IE2.00 | Diabetes care plan declined |
| 103902 | C10FG11 | Type II diabetes mellitus with arthropathy |
| 103935 | 1IA..00 | No evidence of diabetic nephropathy |
| 104254 | 7L10011 | Subcutaneous infusion with insulin pump |
| 104287 | 8Hlc.00 | Referral to community diabetes service |
| 104323 | C10F511 | Type II diabetes mellitus with gangrene |
| 104374 | 67D8.00 | Provision of diabetes clinical summary |
| 104453 | 66At011 | Type 1 diabetic dietary review |
| 104639 | C10FF11 | Type II diabetes mellitus with peripheral angiopathy |
| 105207 | 8HTE100 | Referral to community diabetes clinic |
| 105302 | K08yA00 | Proteinuric diabetic nephropathy |
| 105337 | C10E811 | Type I diabetes mellitus - poor control |
| 105446 | 679c.00 | Insulin administration education |
| 105585 | 8CMW700 | Diabetes clinical pathway |
| 105740 | 2G5d.00 | O/E - Left diabetic foot at increased risk |
| 105741 | 2G5e.00 | O/E - Right diabetic foot at increased risk |
| 105784 | C109912 | Type 2 diabetes mellitus without complication |
| 105937 | 8IEQ.00 | Referral to community diabetes specialist nurse declined |
| 106061 | C10FP11 | Type II diabetes mellitus with ketoacidotic coma |
| 106218 | 9m0A.00 | Declined diabetic retinopathy screening |
| 106269 | 9m0..00 | Diabetic retinopathy screening administrative status |
| 106327 | 9m04.00 | Excluded from diabetic retinopathy screening |
| 106328 | 9m07.00 | Excluded diabetc retinop screen as under care ophthalmolgist |
| 106329 | 9m08.00 | Excluded from diabetic retinopathy screening as blind |
| 106332 | 9m00.00 | Eligible for diabetic retinopathy screening |
| 106350 | 9m05.00 | Excluded from diabetic retinopathy screening as moved away |
| 106351 | 9m09.00 | Excluded from diabetic retinop screen as no longer diabetic |
| 106352 | 9m06.00 | Excluded from diabetic retinopathy screening as deceased |
| 106360 | K27y700 | Erectile dysfunction due to diabetes mellitus |
| 106441 | 9m01.00 | Ineligible for diabetic retinopathy screening |
| 106445 | 9m0E.00 | Excluded from diabetic retinopathy screen physical disorder |
| 106528 | C10FN11 | Type II diabetes mellitus with ketoacidosis |
| 106679 | 8OA3.00 | Provision of written information about diabetes and driving |
| 106722 | 9Oy0300 | Diabetic foot screening invitation second letter |
| 106723 | 9Oy0200 | Diabetic foot screening invitation first letter |
| 106738 | 9Oy0000 | Diabetic foot screening invitation |
| 106778 | 9m0C.00 | Excluded frm diabetic retinopathy screen as terminal illness |
| 106953 | 8IEa.00 | Referral to DAFNE diabetes structured educn prog declined |
| 107331 | 66AH100 | Conversion to insulin in secondary care |
| 107361 | 679L200 | Education about diabetes and driving |
| 107414 | 8I94.00 | Diabetes structured education programme not available |
| 107423 | 661N400 | Diabetes self-management plan review |
| 107464 | 66AS000 | Diabetes Year of Care annual review |
| 107508 | 66AH200 | Conversion to insulin by diabetes specialist nurse |
| 107597 | 9m0D.00 | Excluded from diabetic retinopthy screen as learn disability |
| 107603 | C10P.00 | Diabetes mellitus in remission |
| 107701 | C10FK11 | Hyperosmolar non-ketotic state in type II diabetes mellitus |
| 107739 | 679L211 | Advice about diabetes and driving |
| 107793 | 9Oy0400 | Diabetic foot screening invitation third letter |
| 107824 | C10P100 | Type II diabetes mellitus in remission |
| 107881 | K08yA11 | Clinical diabetic nephropathy |
| 108005 | C109312 | Type 2 diabetes mellitus with multiple complications |
| 108007 | C108311 | Type I diabetes mellitus with multiple complications |
| 108360 | C10P000 | Type I diabetes mellitus in remission |
| 108634 | 9NJy.00 | In-house diabetic foot screening |
| 108655 | 8BAp.00 | Insulin passport not checked |
| 108724 | C10EQ11 | Type I diabetes mellitus with gastroparesis |
| 108890 | 679L300 | Diabetic foot care education |
| 108993 | 661M400 | Diabetes self-management plan agreed |
| 109051 | C10E612 | Insulin dependent diabetes mellitus with gangrene |
| 109103 | C109911 | Type II diabetes mellitus without complication |
| 109197 | C10FH11 | Type II diabetes mellitus with neuropathic arthropathy |
| 109520 | 9m03.00 | Eligibility permanently inactive for diabetic retinop screen |
| 109521 | 9m02.00 | Eligibility temporarily inactive for diabetic retinop screen |
| 109628 | C10P011 | Type 1 diabetes mellitus in remission |
| 109643 | 66o1.00 | Enquiry about diabetic erectile dysfunction declined |
| 109700 | 66AH300 | Conversion to non-insulin injectable medication |
| 109760 | 1M8..00 | Diabetic peripheral neuropathic pain |
| 109806 | 8Hgd.00 | Discharge from secondary care diabetes service |
| 109837 | C10E011 | Type I diabetes mellitus with renal complications |
| 109865 | C109B12 | Type 2 diabetes mellitus with polyneuropathy |
| 109878 | ZC2C911 | Diet advice for insulin-dependent diabetes |
| 110344 | 66o2.00 | Diabetic on non-insulin injectable medication |
| 110379 | 66o5.00 | Diabetic on oral treatment and glucagon-like peptide 1 |
| 110393 | 13B1000 | Diabetic carbohydrate counting diet |
| 110400 | C108F12 | Type 1 diabetes mellitus with diabetic cataract |
| 110409 | 679l.00 | Diabetic injection administration education |
| 110611 | C10P111 | Type 2 diabetes mellitus in remission |
| 110997 | C10y000 | Diabetes mellitus, juvenile, + other specified manifestation |
| 111106 | C108A12 | Type 1 diabetes mellitus without complication |

1. Hospital Episodes Statistics

| **ICD 10** | **DESCRIPTION** |
| --- | --- |
| E10 | Type 1 diabetes mellitus |
| E10.0 | Type 1 diabetes mellitus |
| E10.1 | Type 1 diabetes mellitus |
| E10.2 | Type 1 diabetes mellitus |
| E10.3 | Type 1 diabetes mellitus |
| E10.4 | Type 1 diabetes mellitus |
| E10.5 | Type 1 diabetes mellitus |
| E10.6 | Type 1 diabetes mellitus |
| E10.7 | Type 1 diabetes mellitus |
| E10.8 | Type 1 diabetes mellitus |
| E10.9 | Type 1 diabetes mellitus |
| E11 | Type 2 diabetes mellitus |
| E11.0 | Type 2 diabetes mellitus |
| E11.1 | Type 2 diabetes mellitus |
| E11.2 | Type 2 diabetes mellitus |
| E11.3 | Type 2 diabetes mellitus |
| E11.4 | Type 2 diabetes mellitus |
| E11.5 | Type 2 diabetes mellitus |
| E11.6 | Type 2 diabetes mellitus |
| E11.7 | Type 2 diabetes mellitus |
| E11.8 | Type 2 diabetes mellitus |
| E11.9 | Type 2 diabetes mellitus |
| E13 | Other specified diabetes mellitus |
| E13.0 | Other specified diabetes mellitus |
| E13.1 | Other specified diabetes mellitus |
| E13.2 | Other specified diabetes mellitus |
| E13.3 | Other specified diabetes mellitus |
| E13.4 | Other specified diabetes mellitus |
| E13.5 | Other specified diabetes mellitus |
| E13.6 | Other specified diabetes mellitus |
| E13.7 | Other specified diabetes mellitus |
| E13.8 | Other specified diabetes mellitus |
| E13.9 | Other specified diabetes mellitus |
| E14 | Unspecified diabetes mellitus |
| E14.0 | Unspecified diabetes mellitus |
| E14.1 | Unspecified diabetes mellitus |
| E14.2 | Unspecified diabetes mellitus |
| E14.3 | Unspecified diabetes mellitus |
| E14.4 | Unspecified diabetes mellitus |
| E14.5 | Unspecified diabetes mellitus |
| E14.6 | Unspecified diabetes mellitus |
| E14.7 | Unspecified diabetes mellitus |
| E14.8 | Unspecified diabetes mellitus |
| E14.9 | Unspecified diabetes mellitus |
| G59.0 | Diabetic mononeuropathy |
| G63.2 | Diabetic polyneuropathy |
| H28.0 | Diabetic cataract |
| H36.0 | Diabetic retinopathy |
| M14.2 | Diabetic arthropathy |
| N08.3 | Glomerular disorders in diabetes mellitus |
| O24.0 | Diabetes mellitus in pregnancy: Pre-existing type 1 diabetes mellitus |
| O24.1 | Diabetes mellitus in pregnancy: Pre-existing type 2 diabetes mellitus |
| O24.3 | Diabetes mellitus in pregnancy: Pre-existing diabetes mellitus, unspecified |

1. Chronic kidney disease
2. CPRD

| **medcode** | **readcode** | **readterm** |
| --- | --- | --- |
| 512 | K05..00 | Chronic renal failure |
| 1803 | K011.00 | Nephrotic syndrome with membranous glomerulonephritis |
| 2088 | K00..00 | Acute glomerulonephritis |
| 2471 | K01x100 | Nephrotic syndrome in diabetes mellitus |
| 2475 | C104.11 | Diabetic nephropathy |
| 2773 | K0...00 | Nephritis nephrosis and nephrotic syndrome |
| 2994 | 7L1A100 | Peritoneal dialysis |
| 2995 | G760.00 | Acquired arteriovenous fistula |
| 2996 | 7L1A200 | Haemodialysis NEC |
| 2999 | K01..00 | Nephrotic syndrome |
| 3205 | 7A60100 | Creation of arteriovenous fistula NEC |
| 4480 | K07z.00 | Renal sclerosis NOS |
| 4668 | G22..00 | Hypertensive renal disease |
| 4669 | K02y200 | Chronic focal glomerulonephritis |
| 4850 | K03..11 | Nephritis and nephropathy unspecified |
| 5182 | K03z.00 | Unspecified glomerulonephritis NOS |
| 5291 | K031.00 | Membranous nephritis unspecified |
| 5417 | K00..11 | Acute nephritis |
| 6712 | K050.00 | End stage renal failure |
| 7190 | K072.00 | Glomerulosclerosis |
| 7804 | K02..00 | Chronic glomerulonephritis |
| 8037 | 7L1B000 | Insertion of ambulatory peritoneal dialysis catheter |
| 8330 | K0D..00 | End-stage renal disease |
| 8607 | K0C0.00 | Analgesic nephropathy |
| 8668 | K0A..00 | Glomerular disease |
| 8828 | 14D1.00 | H/O: nephritis |
| 8919 | K08..00 | Impaired renal function disorder |
| 9379 | K08y500 | Acute interstitial nephritis |
| 9765 | 7A61400 | Ligation of acquired arteriovenous fistula |
| 9840 | K010.00 | Nephrotic syndrome with proliferative glomerulonephritis |
| 10081 | K05..11 | Chronic uraemia |
| 10418 | C10ED00 | Type 1 diabetes mellitus with nephropathy |
| 10636 | J624.00 | Hepatorenal syndrome |
| 10647 | K02..11 | Nephritis - chronic |
| 10809 | K021.00 | Chronic membranous glomerulonephritis |
| 11553 | SP08300 | Kidney transplant failure and rejection |
| 11773 | 7L1A.11 | Dialysis for renal failure |
| 11873 | K03..12 | Nephropathy unspecified |
| 11875 | K02..12 | Nephropathy - chronic |
| 12465 | K032.00 | Membranoproliferative nephritis unspecified |
| 12479 | 1Z13.00 | Chronic kidney disease stage 4 |
| 12566 | 1Z12.00 | Chronic kidney disease stage 3 |
| 12585 | 1Z14.00 | Chronic kidney disease stage 5 |
| 12586 | 1Z11.00 | Chronic kidney disease stage 2 |
| 12640 | C10FC00 | Type 2 diabetes mellitus with nephropathy |
| 12720 | 1Z1..00 | Chronic renal impairment |
| 13279 | C104y00 | Other specified diabetes mellitus with renal complications |
| 15097 | K02z.00 | Chronic glomerulonephritis NOS |
| 15106 | G22z.00 | Hypertensive renal disease NOS |
| 15780 | K0z..00 | Nephritis nephrosis and nephrotic syndrome NOS |
| 16008 | K030.00 | Proliferative nephritis unspecified |
| 16465 | K190X00 | Persistent proteinuria |
| 16502 | C104.00 | Diabetes mellitus with renal manifestation |
| 16929 | D215.00 | Anaemia secondary to renal failure |
| 17253 | 8L50.00 | Renal transplant planned |
| 17365 | K01B.00 | Nephrotic syndrome diffuse crescentic glomerulonephritis |
| 17434 | G22..11 | Nephrosclerosis |
| 18209 | C109012 | Type 2 diabetes mellitus with renal complications |
| 18390 | C10FM00 | Type 2 diabetes mellitus with persistent microalbuminuria |
| 18777 | C10F000 | Type 2 diabetes mellitus with renal complications |
| 18779 | 7A61100 | Repair of acquired arteriovenous fistula |
| 19316 | K016.00 | Nephrotic syndrome diffuse membranous glomerulonephritis |
| 20027 | K00y000 | Acute glomerulonephritis in diseases EC |
| 20073 | 7L1A000 | Renal dialysis |
| 20074 | K00..12 | Bright's disease |
| 20129 | K00z.00 | Acute glomerulonephritis NOS |
| 20196 | 14V2.00 | H/O: renal dialysis |
| 20516 | K13yz11 | Salt-losing nephritis |
| 21297 | K0A3.00 | Chronic nephritic syndrome |
| 21423 | K032600 | Berger's IgA or IgG nephropathy |
| 21687 | C345.00 | Gout due to impairment of renal function |
| 21837 | G232.00 | Hypertensive heart&renal dis wth (congestive) heart failure |
| 21947 | K017.00 | Nephrotic syn difus mesangial prolifertiv glomerulonephritis |
| 21983 | C108012 | Type 1 diabetes mellitus with renal complications |
| 21989 | K019.00 | Nephrotic syn diffuse mesangiocapillary glomerulonephritis |
| 22205 | K01x411 | Lupus nephritis |
| 22252 | ZV45100 | [V]Renal dialysis status |
| 22852 | K015.00 | Nephrotic syndrome focal and segmental glomerular lesions |
| 22897 | D310100 | Henoch-Schonlein nephritis |
| 23773 | 7L1B100 | Removal of ambulatory peritoneal dialysis catheter |
| 23913 | K014.00 | Nephrotic syndrome minor glomerular abnormality |
| 23990 | K03T.00 | Tubulo-interstit nephritis not specif as acute or chron |
| 24151 | 7A60.00 | Arteriovenous shunt |
| 24384 | K032400 | Familial glomerulonephritis in Alport's syndrome |
| 24736 | K0C..00 | Drug/heavy-metal-induced tubulo-interstitial and tub conditn |
| 24836 | C109C12 | Type 2 diabetes mellitus with nephropathy |
| 25394 | D215000 | Anaemia secondary to chronic renal failure |
| 25521 | 7A60112 | Creation of brachial-cephalic fistula |
| 25980 | K08z.00 | Impaired renal function disorder NOS |
| 26054 | C10FL00 | Type 2 diabetes mellitus with persistent proteinuria |
| 26220 | K07..00 | Renal sclerosis unspecified |
| 26862 | 7B06300 | Exploration of renal transplant |
| 27335 | K03y000 | Other nephritis and nephrosis in diseases EC |
| 27427 | K01z.00 | Nephrotic syndrome NOS |
| 28158 | TB11.00 | Kidney dialysis with complication without blame |
| 28269 | 7A60111 | Creation of radial-cephalic fistula |
| 28684 | G233.00 | Hypertensive heart and renal disease with renal failure |
| 29013 | 1Z10.00 | Chronic kidney disease stage 1 |
| 29384 | K000.00 | Acute proliferative glomerulonephritis |
| 29634 | K013.00 | Nephrotic syndrome with minimal change glomerulonephritis |
| 29638 | K080.00 | Renal osteodystrophy |
| 30294 | C10EL00 | Type 1 diabetes mellitus with persistent microalbuminuria |
| 30301 | K03X.00 | Unsp nephrit synd diff mesang prolif glomerulonephritis |
| 30323 | C10EK00 | Type 1 diabetes mellitus with persistent proteinuria |
| 30709 | 7L1C000 | Insertion of temporary peritoneal dialysis catheter |
| 30756 | 7L1A500 | Continuous ambulatory peritoneal dialysis |
| 31478 | 7A60300 | Removal of infected arteriovenous shunt |
| 31549 | 7L1A.00 | Compensation for renal failure |
| 31581 | K0A0.00 | Acute nephritic syndrome |
| 32423 | G222.00 | Hypertensive renal disease with renal failure |
| 33580 | K03..00 | Nephritis and nephropathy unspecified |
| 34637 | K080z00 | Renal osteodystrophy NOS |
| 34648 | K080100 | Renal dwarfism |
| 34669 | K03y200 | Other interstitial nephritis |
| 34998 | K020.00 | Chronic proliferative glomerulonephritis |
| 35065 | K03y.00 | Other nephritis and nephrosis unspecified |
| 35105 | C104100 | Diabetes mellitus adult onset with renal manifestation |
| 35107 | C104z00 | Diabetes mellitus with nephropathy NOS |
| 35921 | TA22.00 | Failure of sterile precautions during perfusion |
| 36125 | K03U.00 | Unspecif nephr synd diff concentric glomerulonephritis |
| 36205 | K0A5.00 | Hereditary nephropathy not elsewhere classified |
| 36342 | K032y13 | Mesangioproliferative glomerulonephritis NEC |
| 36442 | 7L1B.11 | Placement ambulatory dialysis apparatus - compens renal fail |
| 38572 | K104.00 | Xanthogranulomatous pyelonephritis |
| 39649 | G220.00 | Malignant hypertensive renal disease |
| 39840 | K08y.00 | Other impaired renal function disorder |
| 40349 | K013.11 | Lipoid nephrosis |
| 40413 | K0A3100 | Chronic nephritic syndrm focal+segmental glomerular lesions |
| 41013 | K08y300 | Renal function impairment with growth failure |
| 41148 | K0B4000 | Renal tubulo-interstitial disorder in SLE |
| 41159 | K0C1.00 | Nephropathy induced by other drugs meds and biologl substncs |
| 41239 | K0A5100 | Hereditary nephropathy NEC focal+segmnt glomerular lesion |
| 41285 | K0A1200 | Rapid progres neph syn diffuse membranous glomerulonephritis |
| 41676 | K034.00 | Renal cortical necrosis unspecified |
| 41881 | K032y14 | Mesangiocapillary glomerulonephritis NEC |
| 42632 | PD12.00 | Medullary cystic disease |
| 43611 | K0A4.00 | Isolated proteinuria with specified morphological lesion |
| 43935 | G221.00 | Benign hypertensive renal disease |
| 44055 | K03yz00 | Other nephritis and nephrosis NOS |
| 44270 | K0A5200 | Hereditry nephropathy NEC difus membran glomerulnephritis |
| 44422 | 14V2.11 | H/O: kidney dialysis |
| 44541 | K0A2600 | Recurrent and persistent haematuria dense deposit disease |
| 44804 | K0A4100 | Isolatd proteinur/specifd morphlgcl les foc+seg glom lesn |
| 45499 | K01x111 | Kimmelstiel - Wilson disease |
| 45523 | K0B..00 | Renal tubulo-interstitial disorders in diseases EC |
| 45867 | K035.00 | Renal medullary necrosis unspecified |
| 45904 | K0B2.00 | Ren tub-interst disordr/blood dis+disordr inv immune mech |
| 46145 | ZV56011 | [V]Aftercare involving renal dialysis NOS |
| 46963 | C108000 | Insulin-dependent diabetes mellitus with renal complications |
| 47135 | PD12100 | Medullary cystic disease adult type |
| 47582 | C10E000 | Type 1 diabetes mellitus with renal complications |
| 47672 | K01x400 | Nephrotic syndrome in systemic lupus erythematosus |
| 47838 | K00yz00 | Other acute glomerulonephritis NOS |
| 47922 | K01x000 | Nephrotic syndrome in amyloidosis |
| 48022 | 7L1Ay00 | Other specified compensation for renal failure |
| 48057 | K0B5.00 | Renal tubulo-interstitial disordrs in transplant rejectn |
| 48261 | K00y200 | Acute focal nephritis |
| 48475 | K08yz11 | Renal acidaemia |
| 49150 | K0y..00 | Other specified nephritis nephrosis or nephrotic syndrome |
| 49642 | K0A2300 | Recur+persist haemuria df mesangial prolif glomerulnephritis |
| 50200 | K0A1600 | Rapid progressive nephritic syndrome dense deposit disease |
| 50225 | C109011 | Type II diabetes mellitus with renal complications |
| 50305 | K032y11 | Hypocomplementaemic persistent glomerulonephritis NEC |
| 50472 | K018.00 | Nephrotic syn difus endocapilary proliftv glomerulonephritis |
| 50728 | K080200 | Renal infantilism |
| 50804 | K08yz00 | Other impaired renal function disorder NOS |
| 50893 | K0C4.00 | Toxic nephropathy not elsewhere classified |
| 51039 | 7B01200 | Bilateral nephrectomy |
| 51113 | K0A5000 | Hereditary nephropathy NEC minor glomerular abnormality |
| 52303 | C109000 | Non-insulin-dependent diabetes mellitus with renal comps |
| 52969 | C341.00 | Gouty nephropathy |
| 53852 | K05..12 | End stage renal failure |
| 53940 | Kyu2100 | [X]Other chronic renal failure |
| 54312 | K0A0500 | Acute neph syn diffuse mesangiocapillary glomerulonephritis |
| 54844 | U612200 | [X]Failure sterile precautions dur kidney dialys/other perf |
| 55100 | K00y300 | Acute diffuse nephritis |
| 55389 | K0A0300 | Acut neph syn diffuse mesangial prolifrative glomnephritis |
| 55548 | K0A7.00 | Glom disordr in blood diseas+disordr invlvg imun mechansm |
| 56760 | 7L1B.00 | Placement ambulatory apparatus compensation renal failure |
| 56893 | K0A3300 | Chron neph syn difus mesangial prolifrtiv glomerulonephritis |
| 56939 | K08y000 | Hypokalaemic nephropathy |
| 56987 | K01A.00 | Nephrotic syndrome dense deposit disease |
| 57072 | K032500 | Other familial glomerulonephritis |
| 57168 | K0A3200 | Chron nephritic syndrom difuse membranous glomerulonephritis |
| 57278 | C10F011 | Type II diabetes mellitus with renal complications |
| 57621 | C108D00 | Insulin dependent diabetes mellitus with nephropathy |
| 57784 | K0C2.00 | Nephropathy induced by unspec drug medicament or biol subs |
| 57926 | K013.12 | Steroid sensitive nephrotic syndrome |
| 57987 | G234.00 | Hyperten heart&renal dis+both(congestv)heart and renal fail |
| 58060 | K0A1300 | Rpd prog neph syn df mesangial prolifratv glomerulonephritis |
| 58164 | K033.00 | Rapidly progressive nephritis unspecified |
| 58618 | 7A60z00 | Arteriovenous shunt NOS |
| 58671 | K0A5300 | Heredtry nephrpthy NEC difus mesangial prolif glomnephrit |
| 58750 | K01x300 | Nephrotic syndrome in polyarteritis nodosa |
| 59194 | 7L1By00 | Placement ambulatory apparatus- compensate renal failure OS |
| 59365 | C109C00 | Non-insulin dependent diabetes mellitus with nephropathy |
| 59992 | K0A4W00 | Isolated proteinuria with unspecified morpholog changes |
| 60128 | K03V.00 | Unspecified nephritic syndrome dense deposit disease |
| 60198 | K0A3600 | Chronic nephritic syndrome dense deposit disease |
| 60302 | 7A60600 | Creation of graft fistula for dialysis |
| 60484 | K0A2500 | Recur+persist hmuria df mesangiocapilary glomerulonephritis |
| 60743 | ZV56.00 | [V]Aftercare involving intermittent dialysis |
| 60796 | C10FL11 | Type II diabetes mellitus with persistent proteinuria |
| 60856 | K0A2700 | Recur+persist haematuria difus crescentic glomerulonephritis |
| 60857 | K0A3700 | Chronic nephritic syn diffuse crescentic glomerulonephritis |
| 60960 | K02y.00 | Other chronic glomerulonephritis |
| 61145 | C341z00 | Gouty nephropathy NOS |
| 61317 | K0A2200 | Recur+persist haematuria difus membranous glomerulonephritis |
| 61344 | C108011 | Type I diabetes mellitus with renal complications |
| 61494 | K022.00 | Chronic membranoproliferative glomerulonephritis |
| 61811 | K0A4500 | Isoltd prteinur+specfd morph les df mesangiocap glomnephr |
| 61814 | K0A0700 | Acute nephrotic syndrm diffuse crescentic glomerulonephritis |
| 62320 | K0A1700 | Rapid progres nephritic syn df crescentic glomerulonephritis |
| 62520 | K03W.00 | Unsp nephrit synd diff endocap prolif glomerulonephritis |
| 62868 | K032300 | Anaphylactoid glomerulonephritis |
| 62980 | K0A5X00 | Hereditary nephropathy unspecif morphological changes |
| 63000 | G231.00 | Benign hypertensive heart and renal disease |
| 63063 | 7A60000 | Insertion of arteriovenous prosthesis |
| 63190 | 7A60200 | Attention to arteriovenous shunt |
| 63305 | 7A60500 | Thrombectomy of arteriovenous fistula |
| 63466 | G23..00 | Hypertensive heart and renal disease |
| 63599 | K00y.00 | Other acute glomerulonephritis |
| 63615 | K02yz00 | Other chronic glomerulonephritis NOS |
| 63786 | K01w.00 | Congenital nephrotic syndrome |
| 64030 | Kyu5G00 | [X]Persistent proteinuria unspecified |
| 64571 | C109C11 | Type II diabetes mellitus with nephropathy |
| 64636 | 7L1Az00 | Compensation for renal failure NOS |
| 64828 | 7L1A600 | Peritoneal dialysis NEC |
| 65064 | K023.00 | Chronic rapidly progressive glomerulonephritis |
| 65089 | 7L1Cz00 | Placement other apparatus- compensate for renal failure NOS |
| 65398 | 7A60y00 | Other specified arteriovenous shunt |
| 65400 | K02y300 | Chronic diffuse glomerulonephritis |
| 66062 | K080300 | Renal rickets |
| 66136 | K0A0100 | Acute nephritic syndrome focal+segmental glomerular lesions |
| 66503 | K0A0200 | Acute nephritic syn diffuse membranous glomerulonephritis |
| 66505 | K0A3000 | Chronic nephritic syndrome minor glomerular abnormality |
| 66613 | K0A4300 | Isoltd prteinur/spcfd morph lesn df mesngl prolf glomneph |
| 66714 | TB11.11 | Renal dialysis with complication without blame |
| 66872 | C108D11 | Type I diabetes mellitus with nephropathy |
| 67193 | K032y00 | Nephritis unsp+OS membranoprolif glomerulonephritis lesion |
| 67197 | A786.00 | Haemorrhagic nephrosonephritis |
| 67232 | G230.00 | Malignant hypertensive heart and renal disease |
| 67261 | K0B4.00 | Ren tub-interstitl disordr/systemc connectv tiss disorder |
| 67460 | K001.00 | Acute nephritis with lesions of necrotising glomerulitis |
| 67995 | K032000 | Focal membranoproliferative glomerulonephritis |
| 68112 | C373600 | Nephropathic amyloidosis |
| 68364 | K0A2100 | Recur+persist haematuria focal+segmental glomerular lesions |
| 68659 | G23z.00 | Hypertensive heart and renal disease NOS |
| 69266 | TA22000 | Failure of sterile precautions during kidney dialysis |
| 69427 | TA02z00 | Accid cut puncture perf h'ge - perfusion NOS |
| 69760 | ZVu3G00 | [X]Other dialysis |
| 71124 | 7L1A300 | Haemofiltration |
| 71174 | K0A1.00 | Rapidly progressive nephritic syndrome |
| 71709 | Kyu0900 | [X]Unsp nephrit synd diff mesang prolif glomerulonephritis |
| 71964 | K0A4200 | Isolatd proteinur/specfd morphlgcl les df membrn glomneph |
| 72303 | K01w000 | Finnish nephrosis syndrome |
| 72478 | Kyu1400 | [X]Nephropathy induced by other drugs+biological substances |
| 73026 | K0A3500 | Chronic neph syn difus mesangiocapillary glomerulonephritis |
| 83513 | 7L1C.00 | Placement other apparatus for compensation for renal failure |
| 85659 | K0A2800 | IgA nephropathy |
| 85991 | C10FM11 | Type II diabetes mellitus with persistent microalbuminuria |
| 88597 | 7L1A400 | Automated peritoneal dialysis |
| 89332 | 9Ot5.00 | Predicted stage chronic kidney disease |
| 90952 | 7B0F100 | Pre-transplantation of kidney work-up recipient |
| 91738 | K0A5600 | Hereditary nephropathy NEC dense deposit disease |
| 93922 | C104000 | Diabetes mellitus juvenile type with renal manifestation |
| 94261 | K00y100 | Acute exudative nephritis |
| 94350 | K032z00 | Nephritis unsp+membranoprolif glomerulonephritis lesion NOS |
| 94373 | K01y.00 | Nephrotic syndrome with other pathological kidney lesions |
| 94789 | 1Z17.00 | Chronic kidney disease stage 1 with proteinuria |
| 94793 | 1Z1B.00 | Chronic kidney disease stage 3 with proteinuria |
| 94842 | Kyu1.00 | [X]Renal tubulo-interstitial diseases |
| 94965 | 1Z15.00 | Chronic kidney disease stage 3A |
| 95121 | 1Z1A.00 | Chronic kidney disease stage 2 without proteinuria |
| 95122 | 1Z1H.00 | Chronic kidney disease stage 4 with proteinuria |
| 95123 | 1Z1C.00 | Chronic kidney disease stage 3 without proteinuria |
| 95145 | 1Z1B.11 | CKD stage 3 with proteinuria |
| 95146 | 1Z19.00 | Chronic kidney disease stage 2 with proteinuria |
| 95175 | 1Z1E.00 | Chronic kidney disease stage 3A without proteinuria |
| 95176 | 1Z1E.11 | CKD stage 3A without proteinuria |
| 95177 | 1Z1G.00 | Chronic kidney disease stage 3B without proteinuria |
| 95178 | 1Z1F.00 | Chronic kidney disease stage 3B with proteinuria |
| 95179 | 1Z16.00 | Chronic kidney disease stage 3B |
| 95180 | 1Z1F.11 | CKD stage 3B with proteinuria |
| 95188 | 1Z1C.11 | CKD stage 3 without proteinuria |
| 95405 | 1Z1L.00 | Chronic kidney disease stage 5 without proteinuria |
| 95406 | 1Z1J.00 | Chronic kidney disease stage 4 without proteinuria |
| 95408 | 1Z1D.00 | Chronic kidney disease stage 3A with proteinuria |
| 95508 | 1Z1K.00 | Chronic kidney disease stage 5 with proteinuria |
| 95546 | K0A2000 | Recurrent+persistnt haematuria minor glomerular abnormality |
| 95571 | 1Z1D.11 | CKD stage 3A with proteinuria |
| 95572 | 1Z18.00 | Chronic kidney disease stage 1 without proteinuria |
| 96131 | 7A60400 | Banding of arteriovenous fistula |
| 96184 | TA02000 | Accid cut puncture perf h'ge - kidney dialysis |
| 96347 | 7A61900 | Ligation of arteriovenous dialysis fistula |
| 96819 | Kyu4000 | [X]Other disorders resulting/impaired renal tubular function |
| 97388 | K032y15 | Mixed membranous and proliferative glomerulonephritis NEC |
| 97587 | 1Z1J.11 | CKD stage 4 without proteinuria |
| 97683 | 1Z1L.11 | CKD stage 5 without proteinuria |
| 97734 | K0A1100 | Rapid progres nephritic syn focal+segmental glomerulr lesion |
| 97758 | K02y000 | Chronic glomerulonephritis + diseases EC |
| 97978 | 1Z1A.11 | CKD stage 2 without proteinuria |
| 97979 | 1Z19.11 | CKD stage 2 with proteinuria |
| 97980 | 1Z17.11 | CKD stage 1 with proteinuria |
| 99139 | K0B6.00 | Balkan nephropathy |
| 99160 | 1Z1K.11 | CKD stage 5 with proteinuria |
| 99312 | 1Z1H.11 | CKD stage 4 with proteinuria |
| 99628 | Kyu0300 | [X]Glomerular disorders in diabetes mellitus |
| 99644 | K012.00 | Nephrotic syndrome+membranoproliferative glomerulonephritis |
| 99685 | K0A0600 | Acute nephritic syndrome dense deposit disease |
| 100205 | K0E..00 | Acute-on-chronic renal failure |
| 100235 | 7A61111 | Ligation of acquired arteriovenous fistula |
| 100292 | Cyu2300 | [X]Unspecified diabetes mellitus with renal complications |
| 100558 | K0A0000 | Acute nephritic syndrome minor glomerular abnormality |
| 100633 | 1Z1G.11 | CKD stage 3B without proteinuria |
| 100693 | Kyu1C00 | [X]Renal tubulo-interstitial disorders/transplant rejection |
| 101358 | K0A0400 | Ac neph syn difus endocaplry prolifrative glomerulonephritis |
| 101453 | Kyu1000 | [X]Other chronic tubulo-interstitial nephritis |
| 101572 | K0A4X00 | Isolated proteinuria with oth specif morpholog changes |
| 101756 | 7L1A011 | Thomas intravascular shunt for dialysis |
| 102163 | C10ED12 | Insulin dependent diabetes mellitus with nephropathy |
| 102201 | C10FC11 | Type II diabetes mellitus with nephropathy |
| 102620 | C10EL11 | Type I diabetes mellitus with persistent microalbuminuria |
| 102946 | C10E012 | Insulin-dependent diabetes mellitus with renal complications |
| 102947 | K13yB00 | Ischaemic nephropathy |
| 103176 | K13yA00 | Dent's disease |
| 103532 | K0A5500 | [X]Heredtry nephrpthy NEC difus mesangiocapilry glomneph |
| 103757 | Kyu1E00 | [X]Tubulo-interstit nephritis, not specif as acute or chron |
| 104201 | SP08H00 | Acute rejection of renal transplant |
| 104586 | 7L1B200 | Flushing of peritoneal dialysis catheter |
| 104619 | K053.00 | Chronic kidney disease stage 3 |
| 104630 | SP08G00 | Acute rejection of renal transplant - grade III |
| 104905 | SP08D00 | Acute-on-chronic rejection of renal transplant |
| 104960 | SP08E00 | Acute rejection of renal transplant - grade I |
| 104963 | K054.00 | Chronic kidney disease stage 4 |
| 104981 | K05..13 | Chronic kidney disease |
| 105151 | K055.00 | Chronic kidney disease stage 5 |
| 105302 | K08yA00 | Proteinuric diabetic nephropathy |
| 105383 | K052.00 | Chronic kidney disease stage 2 |
| 105392 | K051.00 | Chronic kidney disease stage 1 |
| 105436 | SP0G.00 | Anaphylactoid reaction due to haemodialysis |
| 105680 | K0C6.00 | Chronic lithium nephrotoxicity |
| 105723 | K000100 | Crescentic glomerulonephritis |
| 105742 | G72D200 | Aneurysm of anastomotic site of dialysis AV fistula |
| 105760 | G72C.00 | Ruptured aneurysm of dialysis vascular access |
| 105794 | PD12012 | Autosomal recessive medullary cystic disease |
| 105811 | SP08R00 | Renal transplant rejection |
| 105859 | K0A8.00 | Rapidly progressive glomerulonephritis |
| 105976 | K0H..00 | Acute scleroderma renal crisis |
| 106058 | C372400 | Urate nephropathy |
| 106213 | C372411 | Uric acid nephropathy |
| 106620 | SP08J00 | Chronic rejection of renal transplant |
| 106720 | Gy21.00 | Thrombosis of dialysis arteriovenous fistula |
| 106975 | Gy51.00 | Haemorrhage of dialysis arteriovenous fistula |
| 107000 | SP08F00 | Acute rejection of renal transplant - grade II |
| 107027 | K0G..00 | Sickle cell nephropathy |
| 107082 | Gy31.00 | Occlusion of dialysis arteriovenous fistula |
| 107188 | G72D.00 | Aneurysm of dialysis arteriovenous fistula |
| 107216 | Kyu0F00 | [X]Hereditary nephropathy, unspecif morphological changes |
| 107220 | G72D100 | Aneurysm of needle site of dialysis arteriovenous fistula |
| 107260 | Gy41.00 | Infection of dialysis arteriovenous fistula |
| 107382 | K0J0.00 | Renal involvement in scleroderma |
| 107719 | 7A61A00 | Ligation of arteriovenous dialysis graft |
| 107746 | Gy1..00 | Stenosis of dialysis vascular access |
| 107771 | K06..12 | Kidney failure unspecified |
| 107814 | K032200 | Focal glomerulon + focal recurr macroscop glomerulonephritis |
| 107881 | K08yA11 | Clinical diabetic nephropathy |
| 107900 | SP0E.00 | Disorders associated with peritoneal dialysis |
| 108116 | Gy3..00 | Occlusion of dialysis vascular access |
| 108213 | Gy40.00 | Infection of dialysis arteriovenous graft |
| 108423 | Gy60.00 | Rupture of dialysis arteriovenous graft |
| 108591 | K01w100 | Drash syndrome |
| 108699 | Gy10.00 | Stenosis of dialysis arteriovenous graft |
| 108711 | K000111 | CGN - Crescentic glomerulonephritis |
| 108759 | Gy5..00 | Haemorrhage of dialysis vascular access |
| 108766 | 661M200 | Chronic kidney disease self-management plan agreed |
| 108785 | SP0F.00 | Haemodialysis first use syndrome |
| 108816 | K01x.00 | Nephrotic syndrome in diseases EC |
| 108922 | K01w112 | Wilms' tumour + nephrotic syndrome + pseudohermaphroditism |
| 109106 | PD12200 | Nephronophthisis - medullary cystic disease |
| 109135 | Gy30.00 | Occlusion of dialysis arteriovenous graft |
| 109657 | 1Z1Y.00 | CKD with GFR category G3b & albuminuria category A2 |
| 109750 | 2126E00 | Chronic kidney disease resolved |
| 109804 | 1Z1T.00 | CKD with GFR category G3a & albuminuria category A1 |
| 109805 | 1Z1V.00 | CKD with GFR category G3a & albuminuria category A2 |
| 109809 | Gy2..00 | Thrombosis of dialysis vascular access |
| 109837 | C10E011 | Type I diabetes mellitus with renal complications |
| 109884 | SP0H.00 | Disorder associated with dialysis |
| 109905 | 1Z1W.00 | CKD with GFR category G3a & albuminuria category A3 |
| 109945 | K0A1400 | Rapid progres neph syn df endocapilary prolifv glomnephritis |
| 109963 | 1Z1X.00 | CKD with GFR category G3b & albuminuria category A1 |
| 109980 | 1Z1a.00 | CKD with GFR category G4 & albuminuria category A1 |
| 109981 | 1Z1e.00 | CKD with GFR category G5 & albuminuria category A2 |
| 109990 | 1Z1Z.00 | CKD with GFR category G3b & albuminuria category A3 |
| 110003 | 1Z1N.00 | CKD with GFR category G1 & albuminuria category A2 |
| 110033 | 1Z1M.00 | CKD with GFR category G1 & albuminuria category A1 |
| 110051 | Gy4..00 | Infection of dialysis vascular access |
| 110072 | Z919200 | Washing back through haemodialysis lines |
| 110095 | G72D000 | Aneurysm of superficialised artery of dialysis AV fistula |
| 110108 | 1Z1R.00 | CKD with GFR category G2 & albuminuria category A2 |
| 110133 | 1Z1d.00 | CKD with GFR category G5 & albuminuria category A1 |
| 110208 | PD12211 | Autosomal dominant medullary cystic disease |
| 110251 | 1Z1S.00 | CKD with GFR category G2 & albuminuria category A3 |
| 110269 | 1Z1Q.00 | CKD with GFR category G2 & albuminuria category A1 |
| 110467 | 1Z1f.00 | CKD with GFR category G5 & albuminuria category A3 |
| 110484 | 1Z1P.00 | CKD with GFR category G1 & albuminuria category A3 |
| 110626 | 1Z1c.00 | CKD with GFR category G4 & albuminuria category A3 |
| 110749 | K01w200 | Congenital nephrotic syndrome with focal glomerulosclerosis |
| 111022 | 1Z18.11 | CKD stage 1 without proteinuria |
| 111029 | K032y12 | Lobular glomerulonephritis NEC |
| 111103 | SP0E100 | Thrombus in peritoneal dialysis catheter |

1. Hospital Episodes Statistics

| **ICD 10** | **DESCRIPTION** | **MODIFIER_4** |
| --- | --- | --- |
| E10.2 | Type 1 diabetes mellitus | With renal complications |
| E11.2 | Type 2 diabetes mellitus | With renal complications |
| E12.2 | Malnutrition-related diabetes mellitus | With renal complications |
| E13.2 | Other specified diabetes mellitus | With renal complications |
| E14.2 | Unspecified diabetes mellitus | With renal complications |
| I12 | Hypertensive renal disease |  |
| I12.0 | Hypertensive renal disease with renal failure |  |
| I12.9 | Hypertensive renal disease without renal failure |  |
| I13 | Hypertensive heart and renal disease |  |
| I13.0 | Hypertensive heart and renal disease with (congestive) heart failure |  |
| I13.1 | Hypertensive heart and renal disease with renal failure |  |
| I13.2 | Hypertensive heart and renal disease with both (congestive) heart failure and renal failure |  |
| I13.9 | Hypertensive heart and renal disease, unspecified |  |
| N00 | Acute nephritic syndrome |  |
| N00.0 | Acute nephritic syndrome | Minor glomerular abnormality |
| N00.1 | Acute nephritic syndrome | Focal and segmental glomerular lesions |
| N00.2 | Acute nephritic syndrome | Diffuse membranous glomerulonephritis |
| N00.3 | Acute nephritic syndrome | Diffuse mesangial proliferative glomerulonephritis |
| N00.4 | Acute nephritic syndrome | Diffuse endocapillary proliferative glomerulonephritis |
| N00.5 | Acute nephritic syndrome | Diffuse mesangiocapillary glomerulonephritis |
| N00.6 | Acute nephritic syndrome | Dense deposit disease |
| N00.7 | Acute nephritic syndrome | Diffuse crescentic glomerulonephritis |
| N00.8 | Acute nephritic syndrome | Other |
| N00.9 | Acute nephritic syndrome | Unspecified |
| N01 | Rapidly progressive nephritic syndrome |  |
| N01.0 | Rapidly progressive nephritic syndrome | Minor glomerular abnormality |
| N01.1 | Rapidly progressive nephritic syndrome | Focal and segmental glomerular lesions |
| N01.2 | Rapidly progressive nephritic syndrome | Diffuse membranous glomerulonephritis |
| N01.3 | Rapidly progressive nephritic syndrome | Diffuse mesangial proliferative glomerulonephritis |
| N01.4 | Rapidly progressive nephritic syndrome | Diffuse endocapillary proliferative glomerulonephritis |
| N01.5 | Rapidly progressive nephritic syndrome | Diffuse mesangiocapillary glomerulonephritis |
| N01.6 | Rapidly progressive nephritic syndrome | Dense deposit disease |
| N01.7 | Rapidly progressive nephritic syndrome | Diffuse crescentic glomerulonephritis |
| N01.8 | Rapidly progressive nephritic syndrome | Other |
| N01.9 | Rapidly progressive nephritic syndrome | Unspecified |
| N02 | Recurrent and persistent haematuria |  |
| N02.0 | Recurrent and persistent haematuria | Minor glomerular abnormality |
| N02.1 | Recurrent and persistent haematuria | Focal and segmental glomerular lesions |
| N02.2 | Recurrent and persistent haematuria | Diffuse membranous glomerulonephritis |
| N02.3 | Recurrent and persistent haematuria | Diffuse mesangial proliferative glomerulonephritis |
| N02.4 | Recurrent and persistent haematuria | Diffuse endocapillary proliferative glomerulonephritis |
| N02.5 | Recurrent and persistent haematuria | Diffuse mesangiocapillary glomerulonephritis |
| N02.6 | Recurrent and persistent haematuria | Dense deposit disease |
| N02.7 | Recurrent and persistent haematuria | Diffuse crescentic glomerulonephritis |
| N02.8 | Recurrent and persistent haematuria | Other |
| N02.9 | Recurrent and persistent haematuria | Unspecified |
| N03 | Chronic nephritic syndrome |  |
| N03.0 | Chronic nephritic syndrome | Minor glomerular abnormality |
| N03.1 | Chronic nephritic syndrome | Focal and segmental glomerular lesions |
| N03.2 | Chronic nephritic syndrome | Diffuse membranous glomerulonephritis |
| N03.3 | Chronic nephritic syndrome | Diffuse mesangial proliferative glomerulonephritis |
| N03.4 | Chronic nephritic syndrome | Diffuse endocapillary proliferative glomerulonephritis |
| N03.5 | Chronic nephritic syndrome | Diffuse mesangiocapillary glomerulonephritis |
| N03.6 | Chronic nephritic syndrome | Dense deposit disease |
| N03.7 | Chronic nephritic syndrome | Diffuse crescentic glomerulonephritis |
| N03.8 | Chronic nephritic syndrome | Other |
| N03.9 | Chronic nephritic syndrome | Unspecified |
| N04 | Nephrotic syndrome |  |
| N04.0 | Nephrotic syndrome | Minor glomerular abnormality |
| N04.1 | Nephrotic syndrome | Focal and segmental glomerular lesions |
| N04.2 | Nephrotic syndrome | Diffuse membranous glomerulonephritis |
| N04.3 | Nephrotic syndrome | Diffuse mesangial proliferative glomerulonephritis |
| N04.4 | Nephrotic syndrome | Diffuse endocapillary proliferative glomerulonephritis |
| N04.5 | Nephrotic syndrome | Diffuse mesangiocapillary glomerulonephritis |
| N04.6 | Nephrotic syndrome | Dense deposit disease |
| N04.7 | Nephrotic syndrome | Diffuse crescentic glomerulonephritis |
| N04.8 | Nephrotic syndrome | Other |
| N04.9 | Nephrotic syndrome | Unspecified |
| N05 | Unspecified nephritic syndrome |  |
| N05.0 | Unspecified nephritic syndrome | Minor glomerular abnormality |
| N05.1 | Unspecified nephritic syndrome | Focal and segmental glomerular lesions |
| N05.2 | Unspecified nephritic syndrome | Diffuse membranous glomerulonephritis |
| N05.3 | Unspecified nephritic syndrome | Diffuse mesangial proliferative glomerulonephritis |
| N05.4 | Unspecified nephritic syndrome | Diffuse endocapillary proliferative glomerulonephritis |
| N05.5 | Unspecified nephritic syndrome | Diffuse mesangiocapillary glomerulonephritis |
| N05.6 | Unspecified nephritic syndrome | Dense deposit disease |
| N05.7 | Unspecified nephritic syndrome | Diffuse crescentic glomerulonephritis |
| N05.8 | Unspecified nephritic syndrome | Other |
| N05.9 | Unspecified nephritic syndrome | Unspecified |
| N06 | Isolated proteinuria with specified morphological lesion |  |
| N06.0 | Isolated proteinuria with specified morphological lesion | Minor glomerular abnormality |
| N06.1 | Isolated proteinuria with specified morphological lesion | Focal and segmental glomerular lesions |
| N06.2 | Isolated proteinuria with specified morphological lesion | Diffuse membranous glomerulonephritis |
| N06.3 | Isolated proteinuria with specified morphological lesion | Diffuse mesangial proliferative glomerulonephritis |
| N06.4 | Isolated proteinuria with specified morphological lesion | Diffuse endocapillary proliferative glomerulonephritis |
| N06.5 | Isolated proteinuria with specified morphological lesion | Diffuse mesangiocapillary glomerulonephritis |
| N06.6 | Isolated proteinuria with specified morphological lesion | Dense deposit disease |
| N06.7 | Isolated proteinuria with specified morphological lesion | Diffuse crescentic glomerulonephritis |
| N06.8 | Isolated proteinuria with specified morphological lesion | Other |
| N06.9 | Isolated proteinuria with specified morphological lesion | Unspecified |
| N07 | Hereditary nephropathy, not elsewhere classified |  |
| N07.0 | Hereditary nephropathy, not elsewhere classified | Minor glomerular abnormality |
| N07.1 | Hereditary nephropathy, not elsewhere classified | Focal and segmental glomerular lesions |
| N07.2 | Hereditary nephropathy, not elsewhere classified | Diffuse membranous glomerulonephritis |
| N07.3 | Hereditary nephropathy, not elsewhere classified | Diffuse mesangial proliferative glomerulonephritis |
| N07.4 | Hereditary nephropathy, not elsewhere classified | Diffuse endocapillary proliferative glomerulonephritis |
| N07.5 | Hereditary nephropathy, not elsewhere classified | Diffuse mesangiocapillary glomerulonephritis |
| N07.6 | Hereditary nephropathy, not elsewhere classified | Dense deposit disease |
| N07.7 | Hereditary nephropathy, not elsewhere classified | Diffuse crescentic glomerulonephritis |
| N07.8 | Hereditary nephropathy, not elsewhere classified | Other |
| N07.9 | Hereditary nephropathy, not elsewhere classified | Unspecified |
| N08 | Glomerular disorders in diseases classified elsewhere |  |
| N08.0 | Glomerular disorders in infectious and parasitic diseases classified elsewhere |  |
| N08.1 | Glomerular disorders in neoplastic diseases |  |
| N08.2 | Glomerular disorders in blood diseases and disorders involving the immune mechanism |  |
| N08.3 | Glomerular disorders in diabetes mellitus |  |
| N08.4 | Glomerular disorders in other endocrine, nutritional and metabolic diseases |  |
| N08.5 | Glomerular disorders in systemic connective tissue disorders |  |
| N08.8 | Glomerular disorders in other diseases classified elsewhere |  |
| N11 | Chronic tubulo-interstitial nephritis |  |
| N11.0 | Nonobstructive reflux-associated chronic pyelonephritis |  |
| N11.1 | Chronic obstructive pyelonephritis |  |
| N11.8 | Other chronic tubulo-interstitial nephritis |  |
| N11.9 | Chronic tubulo-interstitial nephritis, unspecified |  |
| N12 | Tubulo-interstitial nephritis, not specified as acute or chronic |  |
| N15.0 | Balkan nephropathy |  |
| N16.2 | Renal tubulo-interstitial disorders in blood diseases and disorders involving the immune mechanism |  |
| N16.3 | Renal tubulo-interstitial disorders in metabolic diseases |  |
| N16.4 | Renal tubulo-interstitial disorders in systemic connective tissue disorders |  |
| N16.5 | Renal tubulo-interstitial disorders in transplant rejection |  |
| N16.8 | Renal tubulo-interstitial disorders in other diseases classified elsewhere |  |
| N18 | Chronic kidney disease |  |
| N18.1 | Chronic kidney disease, stage 1 |  |
| N18.2 | Chronic kidney disease, stage 2 |  |
| N18.3 | Chronic kidney disease, stage 3 |  |
| N18.4 | Chronic kidney disease, stage 4 |  |
| N18.5 | Chronic kidney disease, stage 5 |  |
| N18.9 | Chronic kidney disease, unspecified |  |
| N19 | Unspecified kidney failure |  |
| N25 | Disorders resulting from impaired renal tubular function |  |
| N25.0 | Renal osteodystrophy |  |
| N25.1 | Nephrogenic diabetes insipidus |  |
| N25.8 | Other disorders resulting from impaired renal tubular function |  |
| N25.9 | Disorder resulting from impaired renal tubular function, unspecified |  |
| T86.1 | Kidney transplant failure and rejection |  |
| Y84.1 | Kidney dialysis |  |
| Z49 | Care involving dialysis |  |
| Z49.0 | Preparatory care for dialysis |  |
| Z49.1 | Extracorporeal dialysis |  |
| Z49.2 | Other dialysis |  |
| Z99.2 | Dependence on renal dialysis |  |

1. HIV
2. CPRD

| **medcode** | **readcode** | **readterm** |
| --- | --- | --- |
| 2835 | 43C3.11 | HIV positive |
| 8281 | A789A00 | HIV disease resulting in wasting syndrome |
| 9130 | A788.11 | Human immunodeficiency virus infection |
| 23763 | 65QA.00 | AIDS carrier |
| 23770 | A788.00 | Acquired immune deficiency syndrome |
| 23951 | A789200 | HIV disease resulting in candidiasis |
| 24872 | ZV01A00 | [V]Asymptomatic human immunodeficency virus infection status |
| 27053 | 4J34.00 | HIV viral load |
| 27641 | A789300 | HIV disease resulting in Pneumocystis carinii pneumonia |
| 27853 | A789500 | HIV disease resulting in Kaposi's sarcoma |
| 33943 | 65VE.00 | Notification of AIDS |
| 36294 | A788z00 | Acquired human immunodeficiency virus infection syndrome NOS |
| 37006 | A789000 | HIV disease resulting in mycobacterial infection |
| 41185 | Eu02400 | [X]Dementia in human immunodef virus [HIV] disease |
| 43537 | 43j7.00 | HIV 1 nucleic acid detection |
| 44288 | R109.00 | [D]Laboratory evidence of human immunodeficiency virus [HIV] |
| 44303 | A789.00 | Human immunodef virus resulting in other disease |
| 44617 | A789600 | HIV disease resulting in Burkitt's lymphoma |
| 47632 | A788U00 | HIV disease result/haematological+immunologic abnorms,NEC |
| 50076 | A789400 | HIV disease resulting in multiple infections |
| 51708 | A789X00 | HIV dis reslt/oth mal neopl/lymph,h'matopoetc+reltd tissu |
| 53636 | A788400 | Human immunodeficiency virus with neurological disease |
| 58857 | A788000 | Acute human immunodeficiency virus infection |
| 58859 | A788100 | Asymptomatic human immunodeficiency virus infection |
| 62854 | AyuC.00 | [X]Human immunodeficiency virus disease |
| 62891 | A788y00 | Human immunodeficiency virus with other clinical findings |
| 65117 | A789900 | HIV disease resulting in lymphoid interstitial pneumonitis |
| 66367 | A789700 | HIV dis resulting oth types of non-Hodgkin's lymphoma |
| 66368 | A789100 | HIV disease resulting in cytomegaloviral disease |
| 67575 | A788W00 | HIV disease resulting in unspecified malignant neoplasm |
| 69766 | A788200 | HIV infection with persistent generalised lymphadenopathy |
| 69767 | AyuC600 | [X]HIV disease resulting in other non-Hodgkin's lymphoma |
| 70528 | A788500 | Human immunodeficiency virus with secondary infection |
| 70869 | A788300 | Human immunodeficiency virus with constitutional disease |
| 71450 | A788X00 | HIV disease resulting/unspcf infectious+parasitic disease |
| 93642 | 43w3.00 | Human immunodeficiency virus RNA/DNA ratio |
| 96751 | AyuCB00 | [X]HIV disease result/haematological+immunologic abnorms,NEC |
| 96902 | 4J3F.00 | Human immunodeficiency virus viral load by log rank |
| 100769 | AyuCD00 | [X]Unspecified human immunodeficiency virus [HIV] disease |
| 101191 | 66j0.00 | Human immunodeficiency virus annual review |
| 101836 | A788600 | Human immunodeficiency virus with secondary cancers |
| 102117 | AyuC300 | [X]HIV disease resulting in multiple infections |
| 102252 | AyuCC00 | [X]HIV disease resulting in other specified conditions |
| 104134 | AyuC400 | [X]HIV disease resulting/other infectious+parasitic diseases |
| 104466 | L179.00 | HIV disease complicating pregnancy childbirth puerperium |
| 104717 | A789311 | HIV disease resulting in Pneumocystis jirovecii pneumonia |
| 105040 | 9kl..00 | HIV pos gen health check serv declind - enhanc service admin |
| 105324 | A789800 | HIV disease resulting in multiple malignant neoplasms |
| 107594 | 9Nt1000 | Seen by community HIV (human immunodeficiency virus) nurse |
| 107807 | AyuC100 | [X]HIV disease resulting in other viral infections |
| 108054 | A789511 | HIV disease resulting in Kaposi sarcoma |
| 108385 | 9mN..00 | Human immunodeficiency virus infection monitoring invitation |
| 108631 | 8Hle.00 | Referral to community HIV nurse specialist |
| 109327 | 9mN0.00 | HIV infection monitoring telephone invitation |
| 109513 | 4J3N.00 | Human immunodeficiency virus drug resistance test |
| 110374 | 4J3P.00 | Human immunodeficiency virus type 1 subtype identification |

1. Hospital Episodes Statistics

| **ICD 10** | **DESCRIPTION** |
| --- | --- |
| B20 | Human immunodeficiency virus [HIV] disease resulting in infectious and parasitic diseases |
| B20.0 | HIV disease resulting in mycobacterial infection |
| B20.1 | HIV disease resulting in other bacterial infections |
| B20.2 | HIV disease resulting in cytomegaloviral disease |
| B20.3 | HIV disease resulting in other viral infections |
| B20.4 | HIV disease resulting in candidiasis |
| B20.5 | HIV disease resulting in other mycoses |
| B20.6 | HIV disease resulting in Pneumocystis jirovecii pneumonia |
| B20.7 | HIV disease resulting in multiple infections |
| B20.8 | HIV disease resulting in other infectious and parasitic diseases |
| B20.9 | HIV disease resulting in unspecified infectious or parasitic disease |
| B21 | Human immunodeficiency virus [HIV] disease resulting in malignant neoplasms |
| B21.0 | HIV disease resulting in Kaposi sarcoma |
| B21.1 | HIV disease resulting in Burkitt lymphoma |
| B21.2 | HIV disease resulting in other types of non-Hodgkin lymphoma |
| B21.3 | HIV disease resulting in other malignant neoplasms of lymphoid, haematopoietic and related tissue |
| B21.7 | HIV disease resulting in multiple malignant neoplasms |
| B21.8 | HIV disease resulting in other malignant neoplasms |
| B21.9 | HIV disease resulting in unspecified malignant neoplasm |
| B22 | Human immunodeficiency virus [HIV] disease resulting in other specified diseases |
| B22.0 | HIV disease resulting in encephalopathy |
| B22.1 | HIV disease resulting in lymphoid interstitial pneumonitis |
| B22.2 | HIV disease resulting in wasting syndrome |
| B22.7 | HIV disease resulting in multiple diseases classified elsewhere |
| B23 | Human immunodeficiency virus [HIV] disease resulting in other conditions |
| B23.0 | Acute HIV infection syndrome |
| B23.1 | HIV disease resulting in (persistent) generalized lymphadenopathy |
| B23.2 | HIV disease resulting in haematological and immunological abnormalities, not elsewhere classified |
| B23.8 | HIV disease resulting in other specified conditions |
| B24 | Unspecified human immunodeficiency virus [HIV] disease |
| F02.4 | Dementia in human immunodeficiency virus [HIV] disease |
| O98.7 | Human immunodeficiency virus [HIV] disease complicating pregnancy, childbirth and the puerperium |
| R75 | Laboratory evidence of human immunodeficiency virus [HIV] |
| Z21 | Asymptomatic human immunodeficiency virus [HIV] infection status |

1. Cellular immune deficiency
2. CPRD

| **medcode** | **readcode** | **readterm** |
| --- | --- | --- |
| 938 | D201600 | Pancytopenia NOS |
| 5823 | D201500 | Pancytopenia - acquired |
| 10955 | C391100 | Di George syndrome |
| 15422 | D20..00 | Aplastic anaemia |
| 15658 | D201.00 | Acquired aplastic anaemia |
| 16108 | D201000 | Aplastic anaemia due to chronic disease |
| 16903 | F14y011 | Louis - Bar syndrome |
| 21723 | D201z00 | Acquired aplastic anaemia NOS |
| 31275 | D201611 | Pancytopenia with malformation |
| 31322 | C391200 | Wiskott - Aldrich syndrome |
| 31491 | D200211 | Pancytopenia-dysmelia |
| 31541 | C392300 | Severe combined immunodefiency with reticular dysgenesis |
| 37539 | D2...00 | Aplastic and other anaemias |
| 41142 | D204.00 | Idiopathic aplastic anaemia |
| 42394 | D401.11 | Job's syndrome |
| 42439 | C391211 | Thrombocytopenic eczema with immunodeficiency |
| 43166 | D201100 | Aplastic anaemia due to drugs |
| 48035 | C391011 | T-lymphocyte deficiency |
| 48293 | C392100 | Severe combined immunodeficiency |
| 48307 | C391.00 | Deficiencies of cell-mediated immunity |
| 48879 | J637.00 | Hepatic veno-occlusive disease |
| 49530 | BBmC.00 | [M] T-gamma lymphoproliferative disease |
| 49542 | C392500 | Severe combined immunodef with low or normal B-cell numbers |
| 50526 | C392800 | Major histocompatibility complex class I deficiency |
| 50665 | C391000 | Predominantly T-cell immuno-deficiency NOS |
| 57552 | C30yy11 | Adenosine-deaminase deficiency |
| 57859 | D201200 | Aplastic anaemia due to infection |
| 60758 | C391012 | Cellular immunity syndrome |
| 61326 | D201612 | Pancytopenia with pancreatitis |
| 62236 | C392.00 | Combined immunity deficiency |
| 62328 | C392z00 | Combined immunity deficiency NOS |
| 66073 | C392400 | Severe combined immunodef with low T- and B-cell numbers |
| 66239 | D201400 | Aplastic anaemia due to toxic cause |
| 68087 | D20z.00 | Aplastic anaemia NOS |
| 69027 | D200.00 | Constitutional aplastic anaemia |
| 69061 | D200011 | Constitutional aplastic anaemia without malformation |
| 69379 | D200y00 | Other specified constitutional aplastic anaemia |
| 70128 | D201311 | Radiation aplastic anaemia |
| 72804 | C392600 | Adenosine deaminase deficiency |
| 73583 | F14y000 | Ataxia-telangiectasia |
| 93936 | C392700 | Purine nucleoside phosphorylase deficiency |
| 94120 | C392111 | Swiss type agammaglobulinaemia |
| 102848 | D200200 | Constitutional aplastic anaemia with malformation |
| 103977 | C392900 | Major histocompatibility complex class II deficiency |

1. Hospital Episodes Statistics

| **ICD10** | **DESCRIPTION** |
| --- | --- |
| D61 | Other aplastic anaemias |
| D61.0 | Constitutional aplastic anaemia |
| D61.1 | Drug-induced aplastic anaemia |
| D61.2 | Aplastic anaemia due to other external agents |
| D61.3 | Idiopathic aplastic anaemia |
| D61.8 | Other specified aplastic anaemias |
| D61.9 | Aplastic anaemia, unspecified |
| D81 | Combined immunodeficiencies |
| D81.0 | Severe combined immunodeficiency [SCID] with reticular dysgenesis |
| D81.1 | Severe combined immunodeficiency [SCID] with low T- and B-cell numbers |
| D81.2 | Severe combined immunodeficiency [SCID] with low or normal B-cell numbers |
| D81.3 | Adenosine deaminase [ADA] deficiency |
| D81.4 | Nezelof syndrome |
| D81.5 | Purine nucleoside phosphorylase [PNP] deficiency |
| D81.6 | Major histocompatibility complex class I deficiency |
| D81.7 | Major histocompatibility complex class II deficiency |
| D81.8 | Other combined immunodeficiencies |
| D81.9 | Combined immunodeficiency, unspecified |
| D82 | Immunodeficiency associated with other major defects |
| D82.0 | Wiskott-Aldrich syndrome |
| D82.1 | Di George syndrome |
| D82.2 | Immunodeficiency with short-limbed stature |
| D82.3 | Immunodeficiency following hereditary defective response to Epstein-Barr virus |
| D82.4 | Hyperimmunoglobulin E [IgE] syndrome |
| D82.8 | Immunodeficiency associated with other specified major defects |
| D82.9 | Immunodeficiency associated with major defect, unspecified |
| D83 | Common variable immunodeficiency |
| D83.0 | Common variable immunodeficiency with predominant abnormalities of B-cell numbers and function |
| D83.1 | Common variable immunodeficiency with predominant immunoregulatory T-cell disorders |
| D83.2 | Common variable immunodeficiency with autoantibodies to B- or T-cells |
| D83.8 | Other common variable immunodeficiencies |
| D83.9 | Common variable immunodeficiency, unspecified |
| D84.0 | Lymphocyte function antigen-1 [LFA-1] defect |
| G11.3 | Cerebellar ataxia with defective DNA repair |
| D47.1 | Chronic myeloproliferative disease |

1. Solid organ transplant
2. CPRD

| medcode | readcode | readterm |
| --- | --- | --- |
| 242 | 7901000 | Allotransplantation of heart NEC |
| 250 | 7900.00 | Transplantation of heart and lung |
| 2124 | 8HBB.00 | Transplant follow-up |
| 2997 | 7B00.00 | Transplantation of kidney |
| 4405 | 7800.00 | Transplantation of liver |
| 4438 | 7901.00 | Other transplantation of heart |
| 5504 | 7B00z00 | Transplantation of kidney NOS |
| 5911 | ZV42000 | [V]Kidney transplanted |
| 6692 | SP08600 | Liver transplant failure and rejection |
| 9026 | ZV42700 | [V]Liver transplanted |
| 9384 | ZV42100 | [V]Heart transplanted |
| 10394 | ZV42600 | [V]Lung transplanted |
| 10461 | 7450.00 | Transplantation of lung |
| 11113 | SP08100 | Transplanted organ rejection |
| 11553 | SP08300 | Kidney transplant failure and rejection |
| 11745 | 7B00100 | Transplantation of kidney from live donor |
| 18774 | TB00111 | Renal transplant with complication, without blame |
| 22653 | ZV42.00 | [V]Transplanted organ or tissue |
| 24361 | 7B00200 | Transplantation of kidney from cadaver |
| 25896 | SP08z00 | Transplanted organ complication NOS |
| 26862 | 7B06300 | Exploration of renal transplant |
| 27319 | 7800z00 | Transplantation of liver NOS |
| 27679 | SP08500 | Heart-lung transplant failure and rejection |
| 29831 | SP08000 | Transplanted organ failure |
| 30052 | SP...18 | Transplant complications |
| 31997 | TB00200 | Liver transplant with complication, without blame |
| 32025 | 7800000 | Orthotopic transplantation of liver |
| 35368 | 7830.00 | Transplantation of pancreas |
| 37198 | 14S8.00 | H/O: liver recipient |
| 38011 | 7450z00 | Transplantation of lung NOS |
| 41495 | 7901z00 | Other transplantation of heart NOS |
| 44077 | ZV42y12 | [V]Pancreas transplanted |
| 44893 | SP08.00 | Transplanted organ complication |
| 47033 | ZLEQJ00 | Discharge from transplant surgery service |
| 47484 | SP08400 | Heart transplant failure and rejection |
| 47495 | 14SZ.00 | H/O:tissue/organ recipient NOS |
| 48057 | K0B5.00 | Renal tubulo-interstitial disordrs in transplant rejectn |
| 48121 | 7B01500 | Transplant nephrectomy |
| 49028 | 14S2.00 | H/O: kidney recipient |
| 50226 | SyuKK00 | [X]Failure & rejection of other transplanted organ & tissue |
| 53626 | 7900000 | Allotransplantation of heart and lung |
| 54990 | TB00100 | Kidney transplant with complication, without blame |
| 55151 | 7B00000 | Autotransplant of kidney |
| 56993 | 7830100 | Transplantation of whole pancreas |
| 57403 | 14S..00 | H/O: tissue/organ recipient |
| 59394 | 14S3.00 | H/O: heart recipient |
| 59610 | 8C31.00 | Transplant immunosuppression |
| 60955 | 7830300 | Transplantation of islets of Langerhans |
| 61073 | 7900z00 | Transplantation of heart and lung NOS |
| 64438 | TB00000 | Heart transplant with complication, without blame |
| 65772 | 14S9.00 | H/O: lung recipient |
| 66456 | ZV42y00 | [V]Other specified transplanted organ or tissue |
| 66705 | 7B00111 | Allotransplantation of kidney from live donor |
| 67499 | 7830z00 | Transplantation of pancreas NOS |
| 69147 | ZLD4K00 | Discharge by transplant surgeon |
| 69194 | 7800200 | Replacement of previous liver transplant |
| 69734 | 7901y00 | Other specified other transplantation of heart |
| 70712 | SP08011 | Det.ren.func.after ren.transpl |
| 70874 | 7B00y00 | Other specified transplantation of kidney |
| 71422 | 7800100 | Heterotopic transplantation of liver |
| 72004 | 7B01511 | Excision of rejected transplanted kidney |
| 72092 | ZV42z00 | [V]Unspecified transplanted organ or tissue |
| 72939 | 7901100 | Xenotransplantation of heart |
| 73743 | 7450y00 | Other specified transplantation of lung |
| 89445 | 7800111 | Auxillary liver transplant |
| 89924 | 7B00300 | Allotransplantation of kidney from cadaver, heart-beating |
| 90952 | 7B0F100 | Pre-transplantation of kidney work-up, recipient |
| 93366 | 7B0F.00 | Interventions associated with transplantation of kidney |
| 93713 | 7450100 | Single lung transplant |
| 93751 | ZV42y11 | [V]Intestine transplanted |
| 93844 | 7901500 | Revision of transplantation of heart NEC |
| 94964 | 7B0F400 | Post-transplantation of kidney examination, live donor |
| 96095 | 7B0F200 | Pre-transplantation of kidney work-up, live donor |
| 96129 | 7831200 | Excision of transplanted pancreas |
| 96133 | 7B00400 | Allotransplantation kidney from cadaver, heart non-beating |
| 96423 | ZV42.11 | [V]Transplanted organ |
| 96578 | 7450000 | Double lung transplant |
| 97157 | 7800500 | Orthotopic transplantation of liver NEC |
| 98364 | 7B00211 | Allotransplantation of kidney from cadaver |
| 99250 | 7800y00 | Other specified transplantation of liver |
| 99847 | SP08A00 | Post-transplant lymphoproliferative disorder |
| 100073 | 7800112 | Piggy back liver transplant |
| 100621 | 764C.00 | Transplantation of ileum |
| 100693 | Kyu1C00 | [X]Renal tubulo-interstitial disorders/transplant rejection |
| 101231 | 7830200 | Transplantation of tail of pancreas |
| 102998 | 7125.00 | Transplantation of thymus gland |
| 103429 | 7B0F300 | Post-transplantation of kidney examination, recipient |
| 103649 | 9b8K.00 | Transplantation surgery |
| 104049 | 7B0Fz00 | Interventions associated with transplantation of kidney NOS |
| 104050 | 7B0Fy00 | OS interventions associated with transplantation of kidney |
| 104201 | SP08H00 | Acute rejection of renal transplant |
| 104630 | SP08G00 | Acute rejection of renal transplant - grade III |
| 104905 | SP08D00 | Acute-on-chronic rejection of renal transplant |
| 104960 | SP08E00 | Acute rejection of renal transplant - grade I |
| 105328 | 7B00212 | Cadaveric renal transplant |
| 105506 | 7800400 | Orthotopic transplantation of whole liver |
| 105724 | SP08N00 | Unexplained episode of renal transplant dysfunction |
| 105787 | 7B00600 | Xenograft renal transplant |
| 105811 | SP08R00 | Renal transplant rejection |
| 106015 | 7842000 | Transplantation of spleen |
| 106301 | SP08P00 | Stenosis of vein of transplanted kidney |
| 106620 | SP08J00 | Chronic rejection of renal transplant |
| 106866 | SP08W00 | Vascular complication of renal transplant |
| 107000 | SP08F00 | Acute rejection of renal transplant - grade II |
| 107416 | 7901300 | Piggyback transplantation of heart |
| 107752 | SP08T00 | Urological complication of renal transplant |
| 108330 | SP08900 | Complication of transplanted lung |
| 108437 | SP08V00 | Very mild acute rejection of renal transplant |
| 108705 | SP08V11 | Borderline changes of acute rejection |
| 109304 | 9b8B200 | Cardiothoracic transplantation |
| 109455 | 7B00500 | Allotransplantation of kidney from cadaver NEC |
| 110789 | 761N.00 | Transplantation of stomach |

1. Hospital Episodes Statistics

| **ICD10** | **DESCRIPTION** |
| --- | --- |
| T86.1 | Kidney transplant failure and rejection |
| T86.2 | Heart transplant failure and rejection |
| T86.3 | Heart-lung transplant failure and rejection |
| T86.4 | Liver transplant failure and rejection |
| Z94.0 | Kidney transplant status |
| Z94.1 | Heart transplant status |
| Z94.2 | Lung transplant status |
| Z94.3 | Heart and lungs transplant status |
| Z94.4 | Liver transplant status |

1. Office of Population Censuses and Surveys (OPCS) version 4 codes

| **Opcs** | **Description_other** | **Description** |
| --- | --- | --- |
| B171 | Transplantation of thymus gland | Allotransplantation of thymus gland |
| B178 | Transplantation of thymus gland | Other specified |
| B179 | Transplantation of thymus gland | Unspecified |
| E531 | Transplantation of lung | Double lung transplant |
| E532 | Transplantation of lung | Single lung transplant |
| E533 | Transplantation of lung | Single lobe lung transplant |
| E538 | Transplantation of lung | Other specified |
| E539 | Transplantation of lung | Unspecified |
| G681 | Transplantation of ileum | Allotransplantation of ileum |
| G688 | Transplantation of ileum | Other specified |
| G689 | Transplantation of ileum | Unspecified |
| J011 | Transplantation of liver | Orthotopic transplantation of liver nec |
| J012 | Transplantation of liver | Heterotopic transplantation of liver |
| J013 | Transplantation of liver | Replacement of previous liver transplant |
| J014 | Transplantation of liver | Transplantation of liver cells |
| J015 | Transplantation of liver | Orthotopic transplantation of whole liver |
| J018 | Transplantation of liver | Other specified |
| J019 | Transplantation of liver | Unspecified |
| J541 | Transplantation of pancreas | Transplantation of pancreas and duodenum |
| J542 | Transplantation of pancreas | Transplantation of whole pancreas |
| J543 | Transplantation of pancreas | Transplantation of tail of pancreas |
| J544 | Transplantation of pancreas | Transplantation of islet of langerhans |
| J545 | Transplantation of pancreas | Renewal of transplanted pancreatic tissue |
| J548 | Transplantation of pancreas | Other specified |
| J549 | Transplantation of pancreas | Unspecified |
| J553 | Total excision of pancreas | Excision of transplanted pancreas |
| J721 | Other operations on spleen | Transplantation of spleen |
| K011 | Transplantation of heart and lung | Allotransplantation of heart and lung |
| K012 | Transplantation of heart and lung | Revision of transplantation of heart and lung |
| K018 | Transplantation of heart and lung | Other specified |
| K019 | Transplantation of heart and lung | Unspecified |
| K021 | Other transplantation of heart | Allotransplantation of heart nec |
| K022 | Other transplantation of heart | Xenotransplantation of heart |
| K023 | Other transplantation of heart | Implantation of prosthetic heart |
| K024 | Other transplantation of heart | Piggyback transplantation of heart |
| K025 | Other transplantation of heart | Revision of implantation of prosthetic heart |
| K026 | Other transplantation of heart | Revision of transplantation of heart nec |
| K028 | Other transplantation of heart | Other specified |
| K029 | Other transplantation of heart | Unspecified |
| M011 | Transplantation of kidney | Autotransplantation of kidney |
| M012 | Transplantation of kidney | Allotransplantation of kidney from live donor |
| M013 | Transplantation of kidney | Allotransplantation of kidney from cadaver nec |
| M014 | Transplantation of kidney | Allotransplantation of kidney from cadaver heart beating |
| M015 | Transplantation of kidney | Allotransplantation of kidney from cadaver heart non-beating |
| M018 | Transplantation of kidney | Other specified |
| M019 | Transplantation of kidney | Unspecified |
| M026 | Total excision of kidney | Excision of rejected transplanted kidney |
| M084 | Other open operations on kidney | Exploration of transplanted kidney |
| M174 | Interventions associated with transplantation of kidney | Post-transplantation of kidney examination - recipient |
| M178 | Interventions associated with transplantation of kidney | Other specified |
| M179 | Interventions associated with transplantation of kidney | Unspecified |

1. Bone marrow/ stem cell transplant
2. CPRD

| medcode | readcode | readterm |
| --- | --- | --- |
| 1392 | 7K1Q.11 | Bone marrow transplant |
| 15406 | 7K1Q100 | Allograft of bone marrow NEC |
| 18628 | 7K1Q200 | Transfusion of stem cells |
| 21021 | 7K1Q.00 | Graft of bone marrow |
| 22728 | SP08700 | Acute graft-versus-host disease |
| 25695 | SP08800 | Chronic graft-versus-host disease |
| 28232 | 7L14400 | Peripheral blood stem cell graft |
| 52943 | SP08200 | Bone-marrow transplant rejection |
| 54420 | 7L14411 | Second stage peripheral stem cell infusion |
| 63236 | 7L17311 | First stage peripheral stem cell infusion |
| 70870 | 7K1Qz00 | Graft of bone marrow NOS |
| 72436 | 7K1Qy00 | Other specified graft of bone marrow |
| 85492 | 7K1Q300 | Allograft of bone marrow from sibling donor |
| 86063 | 7L14500 | Autologous peripheral blood stem cell transplant |
| 89920 | 7L14700 | Allogeneic peripheral blood stem cell transplant |
| 95840 | 7K1Q400 | Allograft of bone marrow from matched unrelated donor |
| 98608 | 7K1Q600 | Allograft of bone marrow from unmatched unrelated donor |
| 100912 | 7K1Q500 | Allograft of bone marrow from haploidentical donor |
| 110109 | 7L14600 | Syngeneic peripheral blood stem cell transplant |

1. Hospital Episodes Statistics

| **ICD10** | **DESCRIPTION** |
| --- | --- |
| T86.0 | Bone-marrow transplant rejection |

1. Office of Population Censuses and Surveys (OPCS) version 4 codes

| **Opcs** | **Description_other** | **Description** |
| --- | --- | --- |
| W341 | Graft of bone marrow | Autograft of bone marrow |
| W342 | Graft of bone marrow | Allograft of bone marrow nec |
| W343 | Graft of bone marrow | Allograft of bone marrow from sibling donor |
| W344 | Graft of bone marrow | Allograft of bone marrow from matched unrelated donor |
| W345 | Graft of bone marrow | Allograft of bone marrow from haploidentical donor |
| W346 | Graft of bone marrow | Allograft of bone marrow from unmatched unrelated donor |
| W348 | Graft of bone marrow | Other specified |
| W349 | Graft of bone marrow | Unspecified |
| W991 | Graft of cord blood stem cells to bone marrow | Allograft of cord blood stem cells to bone marrow |
| W998 | Graft of cord blood stem cells to bone marrow | Other specified |
| W999 | Graft of cord blood stem cells to bone marrow | Unspecified |
| X334 | Other blood transfusion | Autologous peripheral blood stem cell transplant |
| X335 | Other blood transfusion | Syngeneic peripheral blood stem cell transplant |
| X336 | Other blood transfusion | Allogeneic peripheral blood stem cell transplant |

1. Lymphoma, myeloma, other plasma cell dyscrasias & leukemia
2. CPRD

| medcode | readcode | readterm |
| --- | --- | --- |
| 102688 | ByuD400 | [X]Other malignant immunoproliferative diseases |
| 102688 | ByuD400 | [X]Other malignant immunoproliferative diseases |
| 1481 | B600.00 | Reticulosarcoma |
| 1483 | BBg1.11 | [M]Lymphoma NOS |
| 2462 | B61..00 | Hodgkin's disease |
| 3371 | BBg2.11 | [M]Non Hodgkins lymphoma |
| 3604 | B627.00 | Non - Hodgkin's lymphoma |
| 3672 | BBn0.12 | [M]MYELOMA NOS |
| 3710 | BBB1.00 | [M]Adenolymphoma |
| 4072 | B680.00 | Acute leukaemia NOS |
| 4222 | B64..11 | Lymphatic leukaemia |
| 4250 | B68z.00 | Leukaemia NOS |
| 4251 | B640.00 | Acute lymphoid leukaemia |
| 4413 | B650.00 | Acute myeloid leukaemia |
| 4637 | BBr..00 | [M]Leukaemias |
| 4870 | B625.11 | HISTIOCYTOSIS X (ACUTE, PROGRESSIVE) |
| 4944 | B630.00 | MULTIPLE MYELOMA |
| 5137 | B624.11 | Leukaemic reticuloendotheliosis |
| 5179 | B620.00 | Nodular lymphoma (Brill - Symmers disease) |
| 5915 | BBrA400 | [M]Hairy cell leukaemia |
| 6316 | BBr0100 | [M]Acute leukaemia NOS |
| 7176 | B65..00 | Myeloid leukaemia |
| 7940 | ByuDF11 | [X]Non-Hodgkin's lymphoma NOS |
| 8625 | B641.00 | Chronic lymphoid leukaemia |
| 8649 | ByuDF00 | [X]Non-Hodgkin's lymphoma, unspecified type |
| 9172 | BBmK.00 | [M]WALDENSTROM'S MACROGLOBULINAEMIA |
| 9673 | BBs5.00 | [M]Chronic lymphoproliferative disease |
| 10411 | C333000 | WALDENSTROM'S MACROGLOBULINAEMIA |
| 10726 | B651.00 | Chronic myeloid leukaemia |
| 12006 | B621.00 | Mycosis fungoides |
| 12146 | BBr2000 | [M]Lymphoid leukaemia NOS |
| 12323 | B6...00 | MALIGNANT NEOPLASM OF LYMPHATIC AND HAEMOPOIETIC TISSUE |
| 12335 | B62y.00 | Malignant lymphoma NOS |
| 12464 | B62x200 | Peripheral T-cell lymphoma |
| 15027 | B62yz00 | Malignant lymphoma NOS |
| 15036 | B626.00 | MALIGNANT MAST CELL TUMOURS |
| 15211 | B630.12 | MYELOMATOSIS |
| 15504 | B62y800 | Malignant lymphoma NOS of lymph nodes of multiple sites |
| 16416 | B681.00 | Chronic leukaemia NOS |
| 16460 | BBg2.00 | [M]Malignant lymphoma, non Hodgkin's type |
| 16774 | BBmD.00 | [M] Cutaneous lymphoma |
| 17177 | 1429 | H/O: * leukaemia |
| 17178 | BBg..00 | [M]Lymphomas, NOS or diffuse |
| 17182 | B627C11 | Follicular lymphoma NOS |
| 17460 | B627700 | Diffuse non-Hodgkin's lymphoblastic (diffuse) lymphoma |
| 17887 | B62x.00 | Malignant lymphoma otherwise specified |
| 18383 | BBmH.00 | [M] Large cell lymphoma |
| 18744 | BBn0.11 | [M]MULTIPLE MYELOMA |
| 19028 | B630100 | SOLITARY MYELOMA |
| 19140 | B614800 | Hodgkin's nodular sclerosis of lymph nodes of multiple sites |
| 19372 | B64..00 | Lymphoid leukaemia |
| 19974 | B660.00 | Acute monocytic leukaemia |
| 20437 | BBk..00 | [M]Lymphomas, nodular or follicular |
| 20440 | B69..00 | Myelomonocytic leukaemia |
| 20635 | BBr2011 | [M]Lymphatic leukaemia |
| 20710 | BBj..00 | [M]Hodgkin's disease |
| 21329 | B630200 | PLASMACYTOMA NOS |
| 21402 | B602.00 | Burkitt's lymphoma |
| 21463 | BBgC.11 | [M]Lymphocytic lymphoma NOS |
| 21549 | B627C00 | Follicular non-Hodgkin's lymphoma |
| 22050 | B691.00 | Chronic myelomonocytic leukaemia |
| 22071 | BBr0111 | [M]Blast cell leukaemia |
| 22158 | B630000 | Malignant plasma cell neoplasm, extramedullary plasmacytoma |
| 23711 | BBg1000 | [M]Malignant lymphoma, diffuse NOS |
| 25191 | B68..00 | Leukaemia of unspecified cell type |
| 26135 | BBm6.00 | [M] ALPHA HEAVY CHAIN DISEASE |
| 27330 | B624.00 | Leukaemic reticuloendotheliosis |
| 27340 | B670.11 | Di Guglielmo's disease |
| 27416 | B601.00 | Lymphosarcoma |
| 27458 | B661.00 | Chronic monocytic leukaemia |
| 27520 | B651z00 | Chronic myeloid leukaemia NOS |
| 27664 | B65y100 | Acute promyelocytic leukaemia |
| 27790 | B641.11 | Chronic lymphatic leukaemia |
| 27965 | BBv2.00 | [M]AngiocentricT-cell lymphoma |
| 28276 | B675.00 | ACUTE MYELOFIBROSIS |
| 28639 | B627000 | Follicular non-Hodgkin's small cleaved cell lymphoma |
| 29178 | B614.00 | Hodgkin's disease, nodular sclerosis |
| 29335 | BBr2700 | [M]Adult T-cell leukaemia/lymphoma |
| 29876 | B613z00 | Hodgkin's, lymphocytic-histiocytic predominance NOS |
| 30632 | B67z.00 | Other specified leukaemia NOS |
| 30646 | B6y..00 | MALIGNANT NEOPLASM LYMPHATIC OR HAEMATOPOIETIC TISSUE OS |
| 31324 | B626800 | MAST CELL MALIGNANCY OF LYMPH NODES OF MULTIPLE SITES |
| 31492 | BBm9.00 | [M] Monocytoid B-cell lymphoma |
| 31537 | BBj1100 | [M]Hodgkin,s disease, lymphocytic predominance, nodular |
| 31576 | B627B00 | Other types of follicular non-Hodgkin's lymphoma |
| 31586 | B64y100 | Prolymphocytic leukaemia |
| 31671 | BBn0.00 | [M]PLASMA CELL MYELOMA |
| 31701 | B651.11 | Chronic granulocytic leukaemia |
| 31726 | BBgM.00 | [M]Malignant lymphoma, small cleaved cell, diffuse |
| 31741 | BBj6200 | [M]Hodgkin,s disease, nodular sclerosis, lymphocytic deplet |
| 31749 | BBv0.00 | [M]Monocytoid B-cell lymphoma |
| 31750 | BBr0300 | [M]Chronic leukaemia NOS |
| 31794 | B627W00 | Unspecified B-cell non-Hodgkin's lymphoma |
| 32240 | 4M22.00 | Lymphoma stage III |
| 33333 | B62..00 | OTHER MALIGNANT NEOPLASM OF LYMPHOID AND HISTIOCYTIC TISSUE |
| 33344 | B65z.00 | Myeloid leukaemia NOS |
| 33869 | BBgR.00 | [M]Malignant lymphoma, large cell, diffuse NOS |
| 34089 | B62y400 | Malignant lymphoma NOS of lymph nodes of axilla and arm |
| 34352 | BBgG.12 | [M]Lymphoblastic lymphoma NOS |
| 34692 | B68y.00 | Other leukaemia of unspecified cell type |
| 34926 | B625.00 | LETTERER-SIWE DISEASE |
| 35014 | B622.00 | Sezary's disease |
| 35697 | BBr6.00 | [M]Myeloid leukaemias |
| 35875 | B66..00 | Monocytic leukaemia |
| 36114 | BBg1.00 | [M]Malignant lymphoma NOS |
| 36693 | ZV10600 | [V]Personal history of leukaemia |
| 37112 | B6...11 | MALIGNANT NEOPLASM OF HISTIOCYTIC TISSUE |
| 37182 | B63..00 | Multiple myeloma and immunoproliferative neoplasms |
| 37272 | B67..00 | Other specified leukaemia |
| 37410 | BBr2100 | [M]Acute lymphoid leukaemia |
| 37461 | B64y200 | Adult T-cell leukaemia |
| 37487 | BBrA700 | [M]Acute myelofibrosis |
| 37723 | BBr6011 | [M]Granulocytic leukaemia NOS |
| 38005 | B621z00 | Mycosis fungoides NOS |
| 38321 | B936.12 | PLASMACYTOMA NOS |
| 38331 | B64yz00 | Other lymphoid leukaemia NOS |
| 38914 | B64z.00 | Lymphoid leukaemia NOS |
| 38939 | B613.00 | Hodgkin's disease, lymphocytic-histiocytic predominance |
| 39187 | B631.00 | Plasma cell leukaemia |
| 39490 | BBn0.14 | [M]PLASMACYTIC MYELOMA |
| 39798 | B627X00 | Diffuse non-Hodgkin's lymphoma, unspecified |
| 39883 | BBk5.00 | [M]Malig lymp, follicular centre cell, cleaved, follicular |
| 39906 | BBgE.00 | [M]Malignant lymphoma, centrocytic |
| 40420 | BBr0.00 | [M]Leukaemias unspecified |
| 40508 | BBj6000 | [M]Hodgkin,s disease, nodular sclerosis, lymphocytic predom |
| 40513 | BBkz.00 | [M]Lymphoma, nodular or follicular NOS |
| 40561 | ZV10711 | [V]Personal history of Hodgkin's disease |
| 40740 | ByuD.00 | [X]Malignant neoplasms of lymphoid, haematopoietic and rela |
| 40766 | BBm5.00 | [M] Peripheral T-cell lymphoma NOS |
| 40991 | 4M2..00 | Lymphoma staging system |
| 41369 | B60..00 | Lymphosarcoma and reticulosarcoma |
| 41500 | BBr2300 | [M]Chronic lymphoid leukaemia |
| 41734 | BBr0000 | [M]Leukaemia NOS |
| 41754 | BBg7.00 | [M]Malignant lymphoma, lymphoplasmacytoid type |
| 41841 | BBgB.00 | [M]Malignant lymphoma, follicular centre cell NOS |
| 42198 | BBj6.00 | [M]Hodgkin's disease, nodular sclerosis NOS |
| 42297 | BBrz.00 | [M]Leukaemia NOS |
| 42461 | B61zz00 | Hodgkin's disease NOS |
| 42539 | B670.00 | Acute erythraemia and erythroleukaemia |
| 42579 | B62y300 | Malignant lymphoma NOS of intra-abdominal lymph nodes |
| 42769 | BBjz.00 | [M]Hodgkin's disease NOS |
| 43312 | B936.11 | MYELOMA - SOLITARY |
| 43415 | ByuD000 | [X]Other Hodgkin's disease |
| 43450 | B63z.00 | Immunoproliferative neoplasm or myeloma NOS |
| 43459 | BBn..00 | [M]Plasma cell tumours |
| 43552 | B630.11 | KAHLER'S DISEASE |
| 44196 | B611.00 | Hodgkin's granuloma |
| 44267 | B623.00 | MALIGNANT HISTIOCYTOSIS |
| 44318 | B62xX00 | Oth and unspecif peripheral & cutaneous T-cell lymphomas |
| 44617 | A789600 | HIV disease resulting in Burkitt's lymphoma |
| 45264 | B620100 | Nodular lymphoma of lymph nodes of head, face and neck |
| 45768 | BBm3.12 | [M]ACUTE PROGRESSIVE HISTIOCYTOSIS X |
| 46042 | B630300 | LAMBDA LIGHT CHAIN MYELOMA |
| 46048 | BBr2500 | [M]Prolymphocytic leukaemia |
| 46263 | BBr6700 | [M]Acute myelomonocytic leukaemia |
| 46444 | BBr4.00 | [M]Erythroleukaemias |
| 46877 | BBgL.00 | [M]Malignant lymphoma, small lymphocytic NOS |
| 46931 | BBg4.00 | [M]Malignant lymphoma, stem cell type |
| 46967 | BBl..00 | [M]Mycosis fungoides |
| 47204 | B625z00 | LETTERER-SIWE DISEASE NOS |
| 47330 | BBm2.00 | [M]HISTIOCYTIC MEDULLARY RETICULOSIS |
| 48049 | BBr6800 | [M]Chronic myelomonocytic leukaemia |
| 48155 | BBr2.00 | [M]Lymphoid leukaemias |
| 48253 | BBg8.00 | [M]Malignant lymphoma, immunoblastic type |
| 49131 | BBg0.00 | [M]Lymphomatous tumour, benign |
| 49253 | BBk0.13 | [M]Giant follicular lymphoma |
| 49262 | B627200 | Follicular non-Hodgkin's large cell lymphoma |
| 49301 | B6z..00 | MALIGNANT NEOPLASM LYMPHATIC OR HAEMATOPOIETIC TISSUE NOS |
| 49327 | BBrA500 | [M]Acute megakaryoblastic leukaemia |
| 49530 | BBmC.00 | [M] T-gamma lymphoproliferative disease |
| 49605 | B615.00 | Hodgkin's disease, mixed cellularity |
| 49725 | B64y.00 | Other lymphoid leukaemia |
| 49825 | BBh0.11 | [M]RETICULUM CELL SARCOMA NOS |
| 50668 | B627300 | Diffuse non-Hodgkin's small cell (diffuse) lymphoma |
| 50695 | B627500 | Diffuse non-Hodgkin mixed sml & lge cell (diffuse) lymphoma |
| 50696 | B62y100 | Malignant lymphoma NOS of lymph nodes of head, face and neck |
| 50858 | B674.00 | ACUTE PANMYELOSIS |
| 50928 | BBr2600 | [M]Burkitt's cell leukaemia |
| 51285 | BBj2.00 | [M]Hodgkin's disease, mixed cellularity |
| 51680 | BBgV.00 | [M]Malignant lymphoma, small cell, noncleaved, diffuse |
| 51852 | BBgD.00 | [M]Malig lymphoma, lymphocytic, intermediate different NOS |
| 51895 | BBgz.00 | [M]Lymphoma, diffuse or NOS |
| 52327 | B653000 | Chloroma |
| 52591 | BBgG.13 | [M]Lymphoblastoma NOS |
| 52593 | BBmE.00 | [M] GAMMA HEAVY CHAIN DISEASE |
| 52942 | BBr6300 | [M]Chronic myeloid leukaemia |
| 52946 | 4C53.00 | BONE MARROW: MYELOMA CELLS |
| 53397 | B61z.00 | Hodgkin's disease NOS |
| 53477 | ZV67811 | [V]Follow-up examination after chemotherapy for leukaemia |
| 53551 | B627600 | Diffuse non-Hodgkin's immunoblastic (diffuse) lymphoma |
| 53647 | BBn0.13 | [M]MYELOMATOSIS |
| 54083 | B625800 | LETTERER-SIWE DISEASE OF LYMPH NODES OF MULTIPLE SITES |
| 54190 | BBm8.00 | [M] Angioimmunoblastic lymphadenopathy |
| 54585 | BBr6100 | [M]Acute myeloid leukaemia |
| 54793 | B682.00 | Subacute leukaemia NOS |
| 55303 | B614100 | Hodgkin's nodular sclerosis of head, face and neck |
| 56041 | BBj1.00 | [M]Hodgkin's disease, lymphocytic predominance |
| 57225 | B614000 | Hodgkin's disease, nodular sclerosis of unspecified site |
| 57316 | BBr6600 | [M]Acute promyelocytic leukaemia |
| 57427 | B62y000 | Malignant lymphoma NOS of unspecified site |
| 57544 | BBm4.00 | [M]True histiocytic lymphoma |
| 57671 | B672.00 | Megakaryocytic leukaemia |
| 57713 | BBr8.00 | [M]Eosinophilic leukaemias |
| 57737 | B62x100 | Lymphoepithelioid lymphoma |
| 58015 | BBgQ.00 | [M]Malignant lymphomatous polyposis |
| 58082 | B620800 | Nodular lymphoma of lymph nodes of multiple sites |
| 58684 | B615200 | Hodgkin's mixed cellularity of intrathoracic lymph nodes |
| 58871 | B623z00 | MALIGNANT HISTIOCYTOSIS NOS |
| 58953 | BBk8.00 | [M]Malig lymp,follicular centre cell,noncleaved,follicular |
| 58962 | B62x500 | Malignant immunoproliferative small intestinal disease |
| 59115 | B602100 | Burkitt's lymphoma of lymph nodes of head, face and neck |
| 59593 | BBm3.00 | [M]LETTERER - SIWE DISEASE |
| 59663 | B936.00 | Neoplasm of uncertain behaviour of plasma cells |
| 59755 | B61z200 | Hodgkin's disease NOS of intrathoracic lymph nodes |
| 59778 | B61z100 | Hodgkin's disease NOS of lymph nodes of head, face and neck |
| 59929 | BBr0z00 | [M]Leukaemia unspecified, NOS |
| 60092 | B62y700 | Malignant lymphoma NOS of spleen |
| 60242 | B600000 | Reticulosarcoma of unspecified site |
| 60275 | BBgJ.00 | [M]Malignant lymphoma, centroblastic type NOS |
| 60433 | N330900 | OSTEOPOROSIS IN MULTIPLE MYELOMATOSIS |
| 60504 | BBgC.12 | [M]Lymphocytic lymphosarcoma NOS |
| 60918 | 4M20.00 | Lymphoma stage I |
| 61146 | BBmF.00 | [M] Angiocentric immunoproliferative lesion |
| 61149 | B614300 | Hodgkin's nodular sclerosis of intra-abdominal lymph nodes |
| 61251 | BBgN.00 | [M]Malign lymphoma,lymphocytic,intermediate differn, diffuse |
| 61500 | B690.00 | Acute myelomonocytic leukaemia |
| 61662 | B61z000 | Hodgkin's disease NOS, unspecified site |
| 61693 | ByuD600 | [X]Other myeloid leukaemia |
| 61997 | BBj0.00 | [M]Hodgkin's disease NOS |
| 62330 | BBr6z00 | [M]Other myeloid leukaemia NOS |
| 62380 | B601200 | Lymphosarcoma of intrathoracic lymph nodes |
| 62437 | B62x400 | Malignant reticulosis |
| 63054 | B614z00 | Hodgkin's disease, nodular sclerosis NOS |
| 63105 | B62y500 | Malignant lymphoma NOS of lymph node inguinal region and leg |
| 63239 | BBm1.00 | [M]MALIGNANT HISTIOCYTOSIS |
| 63375 | ByuDE00 | [X]Unspecified B-cell non-Hodgkin's lymphoma |
| 63475 | B652.00 | Subacute myeloid leukaemia |
| 63570 | BBr0113 | [M]Stem cell leukaemia |
| 63625 | B616400 | Hodgkin's lymphocytic depletion lymph nodes axilla and arm |
| 63699 | BBk0.00 | [M]Malignant lymphoma, nodular NOS |
| 63723 | B601z00 | Lymphosarcoma NOS |
| 63864 | BBn2.00 | [M]PLASMACYTOMA NOS |
| 63973 | BBm0.00 | [M]Microglioma |
| 63994 | BBgS.00 | [M]Malignant lymphoma, large cell, cleaved, diffuse |
| 64036 | B612.00 | Hodgkin's sarcoma |
| 64068 | BBnz.00 | [M]Plasma cell tumour NOS |
| 64336 | ByuD300 | [X]Other specified types of non-Hodgkin's lymphoma |
| 64343 | BBj6100 | [M]Hodgkin,s disease, nodular sclerosis, mixed cellularity |
| 64427 | B62z100 | UNSPEC MALIG NEOP LYMPHOID/HISTIOCYTIC LYMPH NODE HEAD/NECK |
| 64515 | ByuDC00 | [X]Diffuse non-Hodgkin's lymphoma, unspecified |
| 64567 | B63y.00 | Other immunoproliferative neoplasms |
| 64618 | BBr3.00 | [M]Plasma cell leukaemias |
| 64670 | B601300 | Lymphosarcoma of intra-abdominal lymph nodes |
| 64947 | BBk0.11 | [M]Brill - Symmers' disease |
| 64963 | BBr0112 | [M]Blastic leukaemia |
| 65122 | B624000 | Leukaemic reticuloendotheliosis of unspecified sites |
| 65123 | B624300 | Leukaemic reticuloend of intra-abdominal lymph nodes |
| 65165 | ByuD900 | [X]Other leukaemia of unspecified cell type |
| 65180 | B627800 | Diffuse non-Hodgkin's lymphoma undifferentiated (diffuse) |
| 65434 | B62z.00 | MALIGNANT NEOPLASMS OF LYMPHOID AND HISTIOCYTIC TISSUE NOS |
| 65483 | B614400 | Hodgkin's nodular sclerosis of lymph nodes of axilla and arm |
| 65489 | B610.00 | Hodgkin's paragranuloma |
| 65584 | BBj1000 | [M]Hodgkin,s disease, lymphocytic predominance, diffuse |
| 65642 | B623300 | MALIGNANT HISTIOCYTOSIS OF INTRA-ABDOMINAL LYMPH NODES |
| 65701 | B620z00 | Nodular lymphoma NOS |
| 65721 | B673.00 | Mast cell leukaemia |
| 65777 | B672.11 | Thrombocytic leukaemia |
| 66089 | B65yz00 | Other myeloid leukaemia NOS |
| 66327 | B620000 | Nodular lymphoma of unspecified site |
| 66367 | A789700 | HIV dis resulting oth types of non-Hodgkin's lymphoma |
| 66603 | BBgK.00 | [M]Malig lymphoma, follicular centre cell, non-cleaved NOS |
| 66694 | BBr6311 | [M]Naegeli-type monocytic leukaemia |
| 67029 | ByuD500 | [X]Other lymphoid leukaemia |
| 67339 | BBp2.00 | [M]MALIGNANT MASTOCYTOSIS |
| 67506 | B614200 | Hodgkin's nodular sclerosis of intrathoracic lymph nodes |
| 67518 | ByuD100 | [X]Other types of follicular non-Hodgkin's lymphoma |
| 67700 | B66..12 | Monoblastic leukaemia |
| 67703 | B616.00 | Hodgkin's disease, lymphocytic depletion |
| 68039 | B612400 | Hodgkin's sarcoma of lymph nodes of axilla and upper limb |
| 68330 | B613100 | Hodgkin's, lymphocytic-histiocytic pred of head, face, neck |
| 68353 | BBmJ.00 | [M] Angioendotheliomatosis |
| 68964 | BBgA.00 | [M]Malignant lymphoma, centroblastic-centrocytic, diffuse |
| 69299 | BBrA111 | [M]Thrombocytic leukaemia |
| 69301 | BBg5.00 | [M]Malignant lymphoma, convoluted cell type NOS |
| 69497 | B623000 | MALIGNANT HISTIOCYTOSIS OF UNSPECIFIED SITE |
| 69767 | AyuC600 | [X]HIV disease resulting in other non-Hodgkin's lymphoma |
| 69980 | BBgC.00 | [M]Malignant lymphoma, lymphocytic, well differentiated NOS |
| 70374 | B600300 | Reticulosarcoma of intra-abdominal lymph nodes |
| 70509 | B627D00 | Diffuse non-Hodgkin's centroblastic lymphoma |
| 70716 | B62zz11 | Immunoproliferative neoplasm |
| 70724 | B653.00 | Myeloid sarcoma |
| 70740 | BBm1.11 | [M]Malignant reticulosis |
| 70842 | B627100 | Follicular non-Hodg mixed sml cleavd & lge cell lymphoma |
| 70935 | BBr4000 | [M]Erythroleukaemia |
| 71031 | B600100 | Reticulosarcoma of lymph nodes of head, face and neck |
| 71117 | BBg3.00 | [M]Malignant lymphoma, undifferentiated cell type NOS |
| 71142 | B613000 | Hodgkin's, lymphocytic-histiocytic predominance unspec site |
| 71238 | B601100 | Lymphosarcoma of lymph nodes of head, face and neck |
| 71262 | B62y600 | Malignant lymphoma NOS of intrapelvic lymph nodes |
| 71304 | B602z00 | Burkitt's lymphoma NOS |
| 71377 | BBr8000 | [M]Eosinophilic leukaemia |
| 71609 | B62z500 | UNSPEC MALIG NEOP LYMPHOID/HISTIOCYTIC NODES INGUINAL/LEG |
| 71619 | BBgT.00 | [M]Malignant lymphoma, large cell, noncleaved, diffuse |
| 71625 | B601000 | Lymphosarcoma of unspecified site |
| 71652 | BBgP.00 | [M]Malignant lymphoma, mixed small and large cell, diffuse |
| 71672 | 4M23.00 | Lymphoma stage IV |
| 71850 | BBr6000 | [M]Myeloid leukaemia NOS |
| 72179 | BBr0200 | [M]Subacute leukaemia NOS |
| 72196 | BBgG.00 | [M]Malignant lymphoma, lymphocytic, poorly different NOS |
| 72197 | B67y000 | Lymphosarcoma cell leukaemia |
| 72222 | BBrA100 | [M]Megakaryocytic leukaemia |
| 72310 | BBr0400 | [M]Aleukaemic leukaemia NOS |
| 72433 | BBh0.00 | [M]Reticulosarcoma NOS |
| 72500 | ByuDB00 | [X]Mal neoplasm/lymphoid,haematopoietic+related tissu,unspcf |
| 72714 | B621500 | Mycosis fungoides of lymph nodes of inguinal region and leg |
| 72725 | B62y200 | Malignant lymphoma NOS of intrathoracic lymph nodes |
| 72774 | B642.00 | Subacute lymphoid leukaemia |
| 73066 | BBrA.00 | [M]Miscellaneous leukaemias |
| 73088 | BBr9000 | [M]Monocytic leukaemia NOS |
| 73135 | BBn2.12 | [M]SOLITARY MYELOMA |
| 73532 | B613300 | Hodgkin's, lymphocytic-histiocytic pred intra-abdominal node |
| 73777 | B624z00 | Leukaemic reticuloendotheliosis NOS |
| 87335 | B624.12 | Hairy cell leukaemia |
| 89230 | BBj9.00 | [M]Hodgkin's granuloma |
| 89329 | ByuD800 | [X]Other specified leukaemias |
| 89657 | B626z00 | MALIGNANT MAST CELL TUMOUR NOS |
| 89762 | ByuD700 | [X]Other monocytic leukaemia |
| 90201 | B62x000 | T-zone lymphoma |
| 91674 | B621300 | Mycosis fungoides of intra-abdominal lymph nodes |
| 91900 | B61z400 | Hodgkin's disease NOS of lymph nodes of axilla and arm |
| 92068 | B620300 | Nodular lymphoma of intra-abdominal lymph nodes |
| 92245 | B613200 | Hodgkin's, lymphocytic-histiocytic pred intrathoracic nodes |
| 92380 | B602500 | Burkitt's lymphoma of lymph nodes of inguinal region and leg |
| 93342 | B66z.00 | Monocytic leukaemia NOS |
| 93384 | B62z200 | UNSPEC MALIG NEOP LYMPHOID/HISTIOCYTIC OF INTRATHORACIC NODE |
| 93951 | B613500 | Hodgkin's, lymphocytic-histiocytic pred inguinal and leg |
| 94005 | B615z00 | Hodgkin's disease, mixed cellularity NOS |
| 94174 | B67y.00 | Other and unspecified leukaemia |
| 94239 | BBp1.00 | [M]MAST CELL SARCOMA |
| 94279 | B61z700 | Hodgkin's disease NOS of spleen |
| 94407 | B615100 | Hodgkin's mixed cellularity of lymph nodes head, face, neck |
| 94415 | B623100 | MALIGNANT HISTIOCYTOSIS OF LYMPH NODES HEAD, FACE AND NECK |
| 94597 | ZV10611 | [V]Personal history of lymphoid leukaemia |
| 94935 | 4M21.00 | Lymphoma stage II |
| 94995 | B620500 | Nodular lymphoma of lymph nodes of inguinal region and leg |
| 95012 | B621800 | Mycosis fungoides of lymph nodes of multiple sites |
| 95049 | B616000 | Hodgkin's lymphocytic depletion of unspecified site |
| 95058 | B600700 | Reticulosarcoma of spleen |
| 95338 | B613600 | Hodgkin's, lymphocytic-histiocytic pred intrapelvic nodes |
| 95464 | BBl0.00 | [M]Mycosis fungoides |
| 95545 | B627911 | Maltoma |
| 95630 | B62x600 | True histiocytic lymphoma |
| 95715 | B627900 | Mucosa-associated lymphoma |
| 95792 | B62zz00 | LYMPHOID AND HISTIOCYTIC MALIGNANCY NOS |
| 95949 | B621000 | Mycosis fungoides of unspecified site |
| 96183 | BBj4.00 | [M]Hodgkin's disease,lymphocytic depletion,diffuse fibrosis |
| 96379 | B621400 | Mycosis fungoides of lymph nodes of axilla and upper limb |
| 96893 | BBrA300 | [M]Myeloid sarcoma |
| 97577 | B602300 | Burkitt's lymphoma of intra-abdominal lymph nodes |
| 97746 | B61z800 | Hodgkin's disease NOS of lymph nodes of multiple sites |
| 97756 | BBl1.00 | [M]Sezary's disease |
| 97852 | BBk7.00 | [M]Malignant lymphoma, centroblastic type, follicular |
| 97863 | B615000 | Hodgkin's disease, mixed cellularity of unspecified site |
| 98009 | BBrA312 | [M]Granulocytic sarcoma |
| 98596 | ByuD200 | [X]Other types of diffuse non-Hodgkin's lymphoma |
| 98840 | B610300 | Hodgkin's paragranuloma of intra-abdominal lymph nodes |
| 98909 | B611100 | Hodgkin's granuloma of lymph nodes of head, face and neck |
| 98961 | BBk2.00 | [M]Malignant lymphoma, centroblastic-centrocytic, follicular |
| 99012 | B61z500 | Hodgkin's disease NOS of lymph nodes inguinal region and leg |
| 99015 | B66y.00 | Other monocytic leukaemia |
| 99067 | C333200 | GAMMA HEAVY CHAIN DISEASE |
| 99200 | BBj7.00 | [M]Hodgkin's disease, nodular sclerosis, cellular phase |
| 99240 | B600z00 | Reticulosarcoma NOS |
| 99413 | B67yz00 | Other and unspecified leukaemia NOS |
| 99655 | BBg6.00 | [M]Lymphosarcoma NOS |
| 99695 | BBlz.00 | [M]Mycosis fungoides NOS |
| 99702 | BBn3.00 | [M]Plasma cell tumour, malignant |
| 99847 | SP08A00 | Post-transplant lymphoproliferative disorder |
| 99887 | B60y.00 | Other specified reticulosarcoma or lymphosarcoma |
| 99951 | B60z.00 | Reticulosarcoma or lymphosarcoma NOS |
| 100006 | B602200 | Burkitt's lymphoma of intrathoracic lymph nodes |
| 100352 | B601500 | Lymphosarcoma of lymph nodes of inguinal region and leg |
| 100423 | B610100 | Hodgkin's paragranuloma of lymph nodes of head, face, neck |
| 100532 | B622z00 | Sezary's disease NOS |
| 100544 | BBh2.00 | [M]Reticulosarcoma, nodular |
| 100615 | B626500 | Mast cell malignancy of lymph nodes inguinal region and leg |
| 100786 | B651000 | Chronic eosinophilic leukaemia |
| 100927 | BBr4z00 | [M]Erythroleukaemia NOS |
| 101114 | B627A00 | Diffuse non-Hodgkin's large cell lymphoma |
| 101271 | BBs1.00 | [M]Acute panmyelosis |
| 101429 | BBj0.11 | [M]Lymphogranuloma, malignant |
| 101465 | B62z800 | Unspec malig neop lymphoid/histiocytic of multiple sites |
| 101530 | B616z00 | Hodgkin's disease, lymphocytic depletion NOS |
| 101606 | B662.00 | Subacute monocytic leukaemia |
| 101715 | B616700 | Hodgkin's disease, lymphocytic depletion of spleen |
| 102158 | B625200 | Letterer-Siwe disease of intrathoracic lymph nodes |
| 102164 | BBn2.11 | [M]Monostotic myeloma |
| 102594 | B627E00 | Diffuse large B-cell lymphoma |
| 102715 | B625000 | Letterer-Siwe disease of unspecified sites |
| 102764 | BBrA600 | [M]Acute panmyelosis |
| 102783 | B651200 | Chronic neutrophilic leukaemia |
| 103245 | B601700 | Lymphosarcoma of spleen |
| 103353 | B62z300 | Unspec malig neop lymphoid/histiocytic intra-abdominal nodes |
| 103645 | B66yz00 | Other monocytic leukaemia NOS |
| 103900 | B626000 | Mast cell malignancy of unspecified site |
| 104152 | B628.00 | Follicular lymphoma |
| 104291 | B61..11 | Hodgkin lymphoma |
| 104325 | B640000 | B-cell acute lymphoblastic leukaemia |
| 104328 | B641000 | B-cell chronic lymphocytic leukaemia |
| 104386 | B62F000 | Small cell B-cell lymphoma |
| 104391 | B627.11 | Non-Hodgkin lymphoma |
| 104412 | B62F200 | Lymphoblastic (diffuse) lymphoma |
| 104418 | B630400 | Solitary plasmacytoma |
| 104475 | B692.00 | Subacute myelomonocytic leukaemia |
| 104484 | B61C.00 | Other classical Hodgkin lymphoma |
| 104620 | B62F100 | Mantle cell lymphoma |
| 104743 | B613800 | Hodgkin's, lymphocytic-histiocytic pred of multiple sites |
| 104788 | B654.00 | Acute myeloblastic leukaemia |
| 104790 | B601800 | Lymphosarcoma of lymph nodes of multiple sites |
| 104862 | B62E300 | Cutaneous T-cell lymphoma |
| 104895 | B617.00 | Nodular lymphocyte predominant Hodgkin lymphoma |
| 104934 | B62Ew00 | Other mature T/NK-cell lymphoma |
| 104939 | B64y500 | Adult T-cell lymphoma/leukaemia (HTLV-1-associated) |
| 105020 | B628300 | Follicular lymphoma grade 3a |
| 105025 | ByuDA00 | [X]Oth spcf mal neoplsm/lymphoid,haematopoietic+rltd tissue |
| 105038 | B627G00 | Mediastinal (thymic) large B-cell lymphoma |
| 105069 | B693.00 | Juvenile myelomonocytic leukaemia |
| 105083 | B62D.00 | Histiocytic sarcoma |
| 105085 | B62E.00 | T/NK-cell lymphoma |
| 105095 | B628100 | Follicular lymphoma grade 2 |
| 105203 | B620200 | Nodular lymphoma of intrathoracic lymph nodes |
| 105286 | B62EA00 | Primary cutaneous CD30-positive T-cell proliferations |
| 105335 | B62A.00 | Sarcoma of dendritic cells |
| 105375 | B62E800 | Blastic NK-cell lymphoma |
| 105472 | B614700 | Hodgkin's disease, nodular sclerosis of spleen |
| 105559 | B62E100 | Anaplastic large cell lymphoma, ALK-positive |
| 105636 | B62E900 | Angioimmunoblastic T-cell lymphoma |
| 105709 | B62E600 | Enteropathy-associated T-cell lymphoma |
| 105762 | B62C.00 | Unifocal Langerhans-cell histiocytosis |
| 105792 | B629.00 | Multifocal multisystemic dissem Langerhans-cell histiocytosi |
| 105841 | B618.00 | Nodular sclerosis classical Hodgkin lymphoma |
| 105889 | B628000 | Follicular lymphoma grade 1 |
| 105925 | B62E700 | Subcutaneous panniculitic T-cell lymphoma |
| 105955 | B62E200 | Anaplastic large cell lymphoma, ALK-negative |
| 105957 | B651100 | Chronic myeloid leukaemia, BCR/ABL positive |
| 105966 | B627F00 | Extranod marg zone B-cell lymphom mucosa-assoc lymphoid tiss |
| 106063 | B628700 | Other types of follicular lymphoma |
| 106137 | BBh..00 | [M]Reticulosarcomas |
| 106197 | BBr7000 | [M]Basophilic leukaemia |
| 106349 | B61z.11 | Hodgkin lymphoma NOS |
| 106483 | BBr6200 | [M]Subacute myeloid leukaemia |
| 106597 | B61B.00 | Lymphocyte-rich classical Hodgkin lymphoma |
| 106867 | B62F.11 | Non-follicular lymphoma |
| 106884 | B62F.00 | Nonfollicular lymphoma |
| 106911 | B613700 | Hodgkin's, lymphocytic-histiocytic predominance of spleen |
| 106924 | B641200 | Clinical stage B chronic lymphocytic leukaemia |
| 106969 | B628500 | Diffuse follicle centre lymphoma |
| 106970 | BBk3.00 | [M]Malig lymphoma, lymphocytic, well differentiated,nodular |
| 107017 | B641011 | Chronic lymphocytic leukaemia of B-cell type |
| 107032 | B616800 | Hodgkin's lymphocytic depletion lymph nodes multiple sites |
| 107052 | B641100 | Clinical stage A chronic lymphocytic leukaemia |
| 107163 | B641300 | Clinical stage C chronic lymphocytic leukaemia |
| 107166 | B628200 | Follicular lymphoma grade 3 |
| 107236 | B651300 | Atypical chronic myeloid leukaemia, BCR/ABL negative |
| 107638 | B62z400 | Unspec malig neop lymphoid/histiocytic lymph node axilla/arm |
| 107643 | B64y400 | T-cell prolymphocytic leukaemia |
| 107773 | BBr8z00 | [M]Eosinophilic leukaemia NOS |
| 107804 | B61z300 | Hodgkin's disease NOS of intra-abdominal lymph nodes |
| 107949 | B62E500 | Hepatosplenic T-cell lymphoma |
| 107973 | B628400 | Follicular lymphoma grade 3b |
| 108037 | B62z000 | Unspec malig neop lymphoid/histiocytic of unspecified site |
| 108102 | C333300 | Heavy chain disease |
| 108182 | B627400 | Diffuse non-Hodgkin's small cleaved cell (diffuse) lymphoma |
| 108235 | C333011 | Waldenstrom macroglobulinaemia |
| 108316 | BBrAz00 | [M]Miscellaneous leukaemia NOS |
| 108424 | B663.00 | Acute monoblastic leukaemia |
| 108656 | B64y300 | B-cell prolymphocytic leukaemia |
| 108715 | B66..11 | Histiocytic leukaemia |
| 108719 | B628600 | Cutaneous follicle centre lymphoma |
| 108775 | B619.00 | Mixed cellularity classical Hodgkin lymphoma |
| 108886 | B615500 | Hodgkin's mixed cellularity of lymph nodes inguinal and leg |
| 108964 | BBr6900 | [M]Juvenile myelomonocytic leukaemia |
| 109342 | B62z600 | Unspec malig neop lymphoid/histiocytic of intrapelvic nodes |
| 109714 | ByuDD00 | [X]Oth and unspecif peripheral & cutaneous T-cell lymphomas |
| 109780 | B62E400 | Extranodal NK/T-cell lymphoma, nasal type |
| 110058 | ZV10613 | [V]Personal history of myeloid leukaemia |
| 110191 | B62B.00 | Multifocal and unisystemic Langerhans-cell histiocytosis |
| 110349 | BBr3z00 | [M]Plasma cell leukaemia NOS |
| 110563 | B616500 | Hodgkin's lymphocytic depletion lymph nodes inguinal and leg |
| 110838 | B676.00 | Acute erythroid leukaemia |
| 110903 | B623800 | Malignant histiocytosis of lymph nodes of multiple sites |
| 111040 | D401.12 | Lipochrome histiocytosis - familial |
| 111113 | BBj3.00 | [M]Hodgkin's disease, lymphocytic depletion NOS |

1. Hospital Episodes Statistics

| **ICD10** | **DESCRIPTION** |
| --- | --- |
| B21.1 | HIV disease resulting in Burkitt lymphoma |
| B21.2 | HIV disease resulting in other types of non-Hodgkin lymphoma |
| B21.3 | HIV disease resulting in other malignant neoplasms of lymphoid, haematopoietic and related tissue |
| C81 | Hodgkin lymphoma |
| C81.0 | Nodular lymphocyte predominant Hodgkin lymphoma |
| C81.1 | Nodular sclerosis (classical) Hodgkin lymphoma |
| C81.2 | Mixed cellularity (classical) Hodgkin lymphoma |
| C81.3 | Lymphocyte depleted (classical) Hodgkin lymphoma |
| C81.4 | Lymphocyte-rich (classical) Hodgkin lymphoma |
| C81.7 | Other (classical) Hodgkin lymphoma |
| C81.9 | Hodgkin lymphoma, unspecified |
| C82 | Follicular lymphoma |
| C82.0 | Follicular lymphoma grade I |
| C82.1 | Follicular lymphoma grade II |
| C82.2 | Follicular lymphoma grade III, unspecified |
| C82.3 | Follicular lymphoma grade IIIa |
| C82.4 | Follicular lymphoma grade IIIb |
| C82.5 | Diffuse follicle centre lymphoma |
| C82.6 | Cutaneous follicle centre lymphoma |
| C82.7 | Other types of follicular lymphoma |
| C82.9 | Follicular lymphoma, unspecified |
| C83 | Non-follicular lymphoma |
| C83.0 | Small cell B-cell lymphoma |
| C83.1 | Mantle cell lymphoma |
| C83.2 | Diffuse non-Hodgkin mixed sml & lge cell (diffuse) lymphoma |
| C83.3 | Diffuse large B-cell lymphoma |
| C83.4 | Diffuse non-Hodgkin's immunoblastic (diffuse) lymphoma |
| C83.5 | Lymphoblastic (diffuse) lymphoma |
| C83.6 | Diffuse non-Hodgkin's lymphoma undifferentiated (diffuse) |
| C83.7 | Burkitt lymphoma |
| C83.8 | Other non-follicular lymphoma |
| C83.9 | Non-follicular (diffuse) lymphoma, unspecified |
| C84 | Mature T/NK-cell lymphomas |
| C84.0 | Mycosis fungoides |
| C84.1 | S├®zary disease |
| C84.2 | Peripheral and cutaneous T-cell lymphomas, T-zone lymphoma |
| C84.3 | Periph & cutan T-cell lymphomas, lymphoepithelioid lymphoma |
| C84.4 | Peripheral T-cell lymphoma, not elsewhere classified |
| C84.5 | Other mature T/NK-cell lymphomas |
| C84.6 | Anaplastic large cell lymphoma, ALK-positive |
| C84.7 | Anaplastic large cell lymphoma, ALK-negative |
| C84.8 | Cutaneous T-cell lymphoma, unspecified |
| C84.9 | Mature T/NK-cell lymphoma, unspecified |
| C85 | Other and unspecified types of non-Hodgkin lymphoma |
| C85.0 | Oth & unspec types of non-Hodgkin's lymphoma, lymphosarcoma |
| C85.1 | B-cell lymphoma, unspecified |
| C85.2 | Mediastinal (thymic) large B-cell lymphoma |
| C85.7 | Other specified types of non-Hodgkin lymphoma |
| C85.9 | Non-Hodgkin lymphoma, unspecified |
| C86 | Other specified types of T/NK-cell lymphoma |
| C86.0 | Extranodal NK/T-cell lymphoma, nasal type |
| C86.1 | Hepatosplenic T-cell lymphoma |
| C86.2 | Enteropathy-type (intestinal) T-cell lymphoma |
| C86.3 | Subcutaneous panniculitis-like T-cell lymphoma |
| C86.4 | Blastic NK-cell lymphoma |
| C86.5 | Angioimmunoblastic T-cell lymphoma |
| C86.6 | Primary cutaneous CD30-positive T-cell proliferations |
| C88 | Malignant immunoproliferative diseases |
| C88.0 | Waldenstr├Âm macroglobulinaemia |
| C88.1 | Alpha heavy chain disease |
| C88.2 | Other heavy chain disease |
| C88.3 | Immunoproliferative small intestinal disease |
| C88.4 | Extranodal marginal zone B-cell lymphoma of mucosa-associated lymphoid tissue [MALT-lymphoma] |
| C88.7 | Other malignant immunoproliferative diseases |
| C88.9 | Malignant immunoproliferative disease, unspecified |
| C90 | Multiple myeloma and malignant plasma cell neoplasms |
| C90.0 | Multiple myeloma |
| C90.1 | Plasma cell leukaemia |
| C90.2 | Extramedullary plasmacytoma |
| C90.3 | Solitary plasmacytoma |
| C91 | Lymphoid leukaemia |
| C91.0 | Acute lymphoblastic leukaemia [ALL] |
| C91.1 | Chronic lymphocytic leukaemia of B-cell type |
| C91.2 | Subacute lymphocytic leukaemia |
| C91.3 | Prolymphocytic leukaemia of B-cell type |
| C91.4 | Hairy-cell leukaemia |
| C91.5 | Adult T-cell lymphoma/leukaemia [HTLV-1-associated] |
| C91.6 | Prolymphocytic leukaemia of T-cell type |
| C91.7 | Other lymphoid leukaemia |
| C91.8 | Mature B-cell leukaemia Burkitt-type |
| C91.9 | Lymphoid leukaemia, unspecified |
| C92 | Myeloid leukaemia |
| C92.0 | Acute myeloblastic leukaemia [AML] |
| C92.1 | Chronic myeloid leukaemia [CML], BCR/ABL-positive |
| C92.2 | Atypical chronic myeloid leukaemia, BCR/ABL-negative |
| C92.3 | Myeloid sarcoma |
| C92.4 | Acute promyelocytic leukaemia [PML] |
| C92.5 | Acute myelomonocytic leukaemia |
| C92.6 | Acute myeloid leukaemia with 11q23-abnormality |
| C92.7 | Other myeloid leukaemia |
| C92.8 | Acute myeloid leukaemia with multilineage dysplasia |
| C92.9 | Myeloid leukaemia, unspecified |
| C93 | Monocytic leukaemia |
| C93.0 | Acute monoblastic/monocytic leukaemia |
| C93.1 | Chronic myelomonocytic leukaemia |
| C93.2 | Subacute monocytic leukaemia |
| C93.3 | Juvenile myelomonocytic leukaemia |
| C93.7 | Other monocytic leukaemia |
| C93.9 | Monocytic leukaemia, unspecified |
| C94 | Other leukaemias of specified cell type |
| C94.0 | Acute erythroid leukaemia |
| C94.1 | Chronic erythraemia |
| C94.2 | Acute megakaryoblastic leukaemia |
| C94.3 | Mast cell leukaemia |
| C94.4 | Acute panmyelosis with myelofibrosis |
| C94.5 | Acute myelofibrosis |
| C94.6 | Myelodysplastic and myeloproliferative disease, not elsewhere classified |
| C94.7 | Other specified leukaemias |
| C95 | Leukaemia of unspecified cell type |
| C95.0 | Acute leukaemia of unspecified cell type |
| C95.1 | Chronic leukaemia of unspecified cell type |
| C95.2 | Subacute leukaemia unsp cell type |
| C95.7 | Other leukaemia of unspecified cell type |
| C95.9 | Leukaemia, unspecified |
| C96 | Other and unspecified malignant neoplasms of lymphoid, haematopoietic and related tissue |
| C96.0 | Multifocal and multisystemic (disseminated) Langerhans-cell histiocytosis [Letterer-Siwe disease] |
| C96.1 | Malignant histiocytosis |
| C96.2 | Malignant mast cell tumour |
| C96.3 | True histiocyt lymphoma |
| C96.4 | Sarcoma of dendritic cells (accessory cells) |
| C96.5 | Multifocal and unisystemic Langerhans-cell histiocytosis |
| C96.6 | Unifocal Langerhans-cell histiocytosis |
| C96.7 | Other specified malignant neoplasms of lymphoid, haematopoietic and related tissue |
| C96.8 | Histiocytic sarcoma |
| C96.9 | Malignant neoplasm of lymphoid, haematopoietic and related tissue, unspecified |
| Z85.6 | Personal history of leukaemia |
| Z85.7 | Personal history of other malignant neoplasms of lymphoid, haematopoietic and related tissues |

1. Radiotherapy and chemotherapy
2. CPRD

| **medcode** | **readcode** | **readterm** |
| --- | --- | --- |
| 320 | 7M37100 | Radiotherapy NEC |
| 783 | 8BAD.00 | CHEMOTHERAPY |
| 1009 | 8HB6.00 | Radiotherapy follow-up |
| 1482 | 59...00 | External radiotherapy |
| 3622 | 5A11.00 | Thyroid gland ablat - irradiat |
| 5019 | 8BAD000 | CANCER CHEMOTHERAPY |
| 5404 | 7M0P.00 | Introduction removable radioactive material into organ NOC |
| 5527 | ZV58000 | [V]Radiotherapy session |
| 9706 | 5154.00 | Radiotherapy completed |
| 10346 | 9N0D.00 | Seen in radiotherapy clinic |
| 10542 | 5A16.00 | Radioactive drug therapy |
| 10776 | 5A...11 | Radiotherapy - internal |
| 10932 | 8H67.00 | Referred for radiotherapy |
| 10950 | ZL93100 | Seen by radiotherapist |
| 14887 | 8BA5.00 | ORAL CHEMOTHERAPY |
| 15362 | 7E0C000 | Introduction of radioactive substance into uterine cavity |
| 15386 | 7L16100 | INTRAVENOUS CHEMOTHERAPY |
| 16662 | ZV66100 | [V]Convalescence after radiotherapy |
| 16771 | 59...11 | X-ray therapy -external |
| 16935 | 515..00 | Progress of radiotherapy |
| 18079 | 8HB7.00 | CHEMOTHERAPY FOLLOW-UP |
| 18675 | 8BAK.00 | POST-OPERARTIVE CHEMOTHERAPY |
| 18715 | 5AB..00 | Stereotactic radiotherapy |
| 18832 | 8BAa.00 | DATE CHEMOTHERAPY COMPLETED |
| 18904 | ZV67100 | [V]Radiotherapy follow-up |
| 19467 | 8BAJ.00 | PRE-OPERATIVE CHEMOTHERAPY |
| 20282 | 7M0Py00 | Introduction removable radioactive material to organ NOC OS |
| 20336 | 7272200 | Radiotherapy to lesion of retina |
| 20381 | ZV58100 | [V]MAINTENANCE CHEMOTHERAPY |
| 20443 | ZV67200 | [V]CHEMOTHERAPY FOLLOW-UP |
| 21318 | 8BA5.11 | Oral cytotoxic drug therapy |
| 22472 | 59Z..00 | External radiotherapy NOS |
| 22490 | 8BAL.00 | COMBINED PRE-OPERATIVE CHEMOTHERAPY AND RADIOTHERAPY |
| 23589 | 7M0Pz00 | Introduction removable radioactive material to organ NOC NOS |
| 25479 | ZV67800 | [V]FOLLOW-UP EXAMIN AFTER CHEMOTHERAPY FOR MALIGN NEOPLASM |
| 25490 | ZLD2400 | Discharge by radiotherapist |
| 26149 | 8CV1.00 | CHEMOTHERAPY STARTED |
| 28071 | 7L10200 | CONTINUOUS INFUSION OF CHEMOTHERAPY |
| 28427 | 7D03400 | Implantation of radioactive substance into vulva |
| 28712 | 8J01.00 | Iodine seed radiotherapy |
| 28809 | 8BAM.00 | COMBINED POST-OPERATIVE CHEMOTHERAPY AND RADIOTHERAPY |
| 29285 | 5149.00 | Radiotherapy-tumour palliation |
| 29301 | 514Z.00 | Radiotherapy purpose - NOS |
| 29679 | 5146.00 | Radiotherapy - post-op.control |
| 30264 | 7L19300 | SUBCUTANEOUS CHEMOTHERAPY |
| 30547 | ZV67700 | [V]Follow-up exam after radiotherapy for malignant neoplasm |
| 30942 | 9N1yC00 | Seen in radiotherapy clinic |
| 31489 | ZV58800 | [V]CHEMOTHERAPY SESSION FOR NEOPLASM |
| 31527 | 514..00 | Purpose of radiotherapy |
| 31804 | TB12100 | Radiotherapy procedure with complication, without blame |
| 32478 | ZL13100 | Under care of radiotherapist |
| 35597 | 5147.00 | Radiotherapy for analgesia |
| 35609 | ZV66200 | [V]CONVALESCENCE AFTER CHEMOTHERAPY |
| 36225 | 5A4..11 | Radium needles |
| 36489 | 7M0B200 | Radiofrequency controlled thermal destruction of organ NOC |
| 36810 | 5AC..00 | Strontium 89 therapy |
| 36981 | 5155.00 | Awaiting radiotherapy |
| 37123 | 7L18200 | INTRAMUSCULAR CHEMOTHERAPY |
| 38466 | 5151.00 | Radiotherapy started |
| 38662 | 7M0P000 | Introduction of radioactive caesium into organ NOC |
| 38773 | 5A...00 | Other nuclear therapy |
| 39951 | 5A9..00 | Selectron therapy |
| 40070 | 7101100 | Implantation of radioactive substance into pituitary gland |
| 40310 | D400312 | Neutropenia due to irradiation |
| 40490 | 7046200 | INTRATHECAL CHEMOTHERAPY |
| 41044 | 8J...00 | Radiotherapy treatment groups |
| 42351 | 591..00 | X-ray beam therapy |
| 42671 | 5A8..00 | Other radiotherapy misc. |
| 43261 | 5144.00 | Radiotherapy - pre-op. control |
| 44148 | 5914.11 | Deep X-ray therapy |
| 44831 | 5153.00 | Radiotherapy stopped |
| 45087 | 8H3L.00 | Non-urgent radiotherapy admisn |
| 45099 | 515Z.00 | Radiotherapy progress NOS |
| 46028 | 8BAY.00 | DATE CHEMOTHERAPY STOPPED |
| 46824 | 8H2G.00 | Admit radiotherapy emergency |
| 48991 | 5136.00 | X-ray metastasis control |
| 49760 | U603318 | [X] Adverse reaction to mitomycin |
| 50731 | 7H2C000 | Introduction of radioactive substance into peritoneal cavity |
| 51781 | ZVu3L00 | [X]OTHER CHEMOTHERAPY |
| 51787 | 5975.00 | EXT.BEAM + CHEMOTHERAPY |
| 51959 | 8BAI.00 | AMBULATORY CHEMOTHERAPY |
| 52108 | 8F83.00 | Convalescence after radiother. |
| 53180 | 5A46.00 | Radioth.: temp. pelvic implant |
| 53477 | ZV67811 | [V]FOLLOW-UP EXAMINATION AFTER CHEMOTHERAPY FOR LEUKAEMIA |
| 54828 | 7D15400 | Implantation of radioactive substance into vagina |
| 54919 | 5A8Z.00 | Other radiotherapy NOS |
| 55261 | 5152.00 | Radiotherapy changed |
| 55828 | 5145.00 | Radiotherapy -intra-op.control |
| 55832 | 5143.00 | Radioth. for lymphat.irradiat. |
| 55836 | 5148.00 | Radiotherapy for inflammation |
| 57591 | 591Z.00 | X-ray beam therapy NOS |
| 58036 | 7220100 | Radiotherapy to lacrimal gland |
| 59684 | 7M0c.00 | Radiotherapy procedures |
| 59796 | 5914.00 | Deep X-ray therapy 150-400 Kv |
| 59890 | 5913.00 | Half deep therapy 60-150 Kv |
| 60076 | 5141.00 | Radioth.for immunosuppression |
| 60091 | 5912.00 | Superfic.X-ray therapy 10-60Kv |
| 60674 | 7052200 | Radiotherapy to lesion of peripheral nerve |
| 60682 | ZV6B100 | [V]FOLLOW-UP EXAM AFTER CHEMOTHERAPY FOR OTHER CONDITIONS |
| 61955 | 5AA..00 | Iridium wire therapy |
| 62202 | 8J00.00 | High dose brachytherapy |
| 62864 | 5A73.00 | Radio-chemo.: oral route |
| 62951 | 5A15.00 | Bone tumour/metast.irradiat. |
| 64143 | 597Z.00 | Combined radiotherapy NOS |
| 64801 | 5A3..00 | Intern.radioth-permanent seeds |
| 64997 | 7L1d.00 | DELIVERY OF CHEMOTHERAPY FOR NEOPLASM |
| 65739 | 5A53.00 | Preload radioth.- nose |
| 67248 | 5A12.00 | Thyroid tumour/metast irradiat |
| 68344 | 7244400 | Radiotherapy to lesion of cornea |
| 68423 | 7L1Z.00 | Radiotherapy delivery |
| 69165 | 594..00 | Heavy particle therapy |
| 69387 | 5974.00 | EXT.BEAM-SURGERY+CHEMOTHERAPY |
| 69877 | 5A3Z.00 | Radioth.: permanent seeds NOS |
| 69979 | 5AZ..00 | Other nuclear therapy NOS |
| 70128 | D201311 | Radiation aplastic anaemia |
| 70246 | 5A1..00 | Internal metabolic radiotherap |
| 70290 | 5A7..00 | RADIOMIMETIC CHEMOTHERAPY |
| 70386 | TA32.00 | Overdose of radiation in therapy |
| 70445 | 7M0cy00 | Other specified radiotherapy procedures |
| 70478 | 5A4..00 | Radioth.: temporary implant |
| 70549 | 5142.00 | Radioth. for haemopo. irradiat |
| 71008 | 7809400 | Percutaneous radiofrequency ablation of lesion of liver |
| 71098 | 592Z.00 | High-energy beam therapy NOS |
| 71598 | 5A45.00 | Radioth.: temp. abdom. implant |
| 71599 | 5A1Z.00 | Internal metabolic radioth.NOS |
| 71837 | U603300 | [X]OTHER ANTINEOPLAST DRUGS CAUS ADVERSE EFF IN THERAP USE |
| 71926 | 5134.00 | X-ray # reduction control |
| 72850 | 5971.00 | Extern.beam+intern.radiotherap |
| 72978 | 597..00 | Combined radiotherapy |
| 73171 | 5A64.00 | Afterload radioth.-fem.genital |
| 73172 | 5A7Z.00 | RADIO-CHEMOTHERAPY NOS |
| 73173 | 5A13.00 | Bone marrow suppres.-irradiat. |
| 73300 | 5922.00 | Betatron photon therapy |
| 73462 | 5917.00 | Intracavitary X-ray therapy |
| 73692 | 5A4Z.00 | Radioth.:temporary implant NOS |
| 85984 | 5941.00 | Proton therapy |
| 86329 | 7L1Z400 | Oral delivery of radiotherapy for thyroid ablation |
| 87860 | 7M0P300 | Intro radioactive substance org interstit brachytherapy NOC |
| 88367 | 7B0A800 | Percutaneous radiofrequency ablation of lesion of kidney |
| 88762 | 5135.00 | Radiological tumour control |
| 88889 | 7M0Q300 | Radioactive seed implantation NOC |
| 89452 | 7L1b.00 | PROCUREMENT DRUGS FOR CHEMOTHERAPY FOR NEOPLASM IN BANDS 1-5 |
| 90743 | 7L1e.00 | DELIVERY OF ORAL CHEMOTHERAPY FOR NEOPLASM |
| 91433 | 7B3CB00 | Radioactive seed implantation into prostate |
| 91694 | 7L1Z300 | Delivery of a fraction of external beam radiotherapy NEC |
| 91778 | 7L1d000 | Del comp chemo neo inc prolong infusion treat first attend |
| 91891 | 7L1Zz00 | Radiotherapy delivery NOS |
| 91918 | 7272700 | External beam radiotherapy to lesion of retina |
| 92174 | 7454400 | Percutaneous radiofrequency ablation of lesion of lung |
| 92956 | 7L1Z011 | Delivery of a fraction of total body irradiation |
| 92999 | 5A27.00 | Radioth.: infuse organ cavity |
| 93607 | 5A81.00 | Give radiosensitising drug |
| 93669 | 7L1Y.00 | Radiotherapy preparation |
| 94305 | 596..00 | Short dis.+contact radiotherap |
| 94306 | 593..00 | Fast-electron therapy |
| 94431 | 7L1ez00 | DELIVERY OF ORAL CHEMOTHERAPY FOR NEOPLASM NOS |
| 94478 | 5A42.00 | Radioth:temp.head/neck implant |
| 94479 | 5973.00 | Ext.beam-surg.+post-op.radioth |
| 94617 | 5A62.00 | Afterload radioth.- upper GIT |
| 94760 | U603316 | [X] Adverse reaction to chlorambucil |
| 94764 | 7L1Y000 | Preparation for total body irradiation |
| 95009 | 7L1Z100 | Delivery of a fraction of intracavitary radiotherapy |
| 95066 | 5961.00 | Radium contact therapy |
| 95098 | 5A33.00 | Radioth.: seeds into cavity |
| 95126 | 7L1Zy00 | Other specified radiotherapy delivery |
| 95424 | 7L1dz00 | DELIVERY OF CHEMOTHERAPY FOR NEOPLASM NOS |
| 95693 | 7L1Xy00 | Other specified radiotherapy volume definition |
| 95851 | U603311 | [X] ADVERSE REACTION TO ANTINEOPLASTIC ANTIBIOTICS |
| 95890 | 5962.00 | Beta source contact therapy |
| 96310 | 7L1dy00 | OTHER SPECIFIED DELIVERY OF CHEMOTHERAPY FOR NEOPLASM |
| 96446 | U613200 | [X]Overdose of radiation given during therapy |
| 96458 | 7L1Z000 | Delivery of a fraction of total body irradiation (TBI) |
| 96467 | 5942.00 | Deuteron therapy |
| 96535 | 7B3BC00 | Endoscopic radiofrequency ablation of lesion of prostate |
| 97097 | 5131.00 | Radiological pre-op. control |
| 97106 | 5A28.00 | Radioth.: infiltrate tissue |
| 97154 | 5137.00 | X-ray radiotherapy control |
| 97253 | 7K1V700 | Percutaneous radiofrequency ablation of lesion of bone |
| 97412 | 7M0P400 | Intro radioactive substance into organ for brachytherapy NOC |
| 98358 | 7L1h000 | Preparation for intensity modulated radiation therapy |
| 98625 | U603100 | [X]ANTINEOPLAST ANTIMETABS CAUS ADVERSE EFF IN THERAP USE |
| 98750 | Z9KG500 | Brachytherapy monitoring |
| 98826 | 5A58.00 | Preload radioth-female genital |
| 98828 | 592..00 | High-energy beam therapy |
| 98829 | 5921.00 | Linear-accelerator photon ther |
| 98830 | 593Z.00 | Fast-electron therapy NOS |
| 98882 | 5A74.00 | Radio-chemo.: I-V route |
| 98902 | 7M0c500 | Superficial or orthovoltage treatment for radiotherapy |
| 98940 | 7L1i.00 | Preparation for brachytherapy |
| 99327 | 5963.00 | Mould technique gamma/beta |
| 99469 | 56B5.00 | Image: field control:radiother |
| 100382 | 5A21.00 | Radioth.: infuse - skull/brain |
| 100390 | 7M0P500 | Intro non-remov radioact subst into organ for brachyther NOC |
| 100396 | 7L1Z500 | Delivery of a fraction of intraluminal brachytherapy |
| 100724 | 5A2Z.00 | Intern. unsealed radioth. NOS |
| 100725 | 5A26.00 | Radioth:infuse-urinary bladder |
| 100758 | 5953.00 | Comb.tele+int.dist.curietherap |
| 100832 | 5133.00 | Radiological post-op. control |
| 100901 | 7M0c400 | Megavoltage treatment for simple radiotherapy |
| 100942 | U60331B | [X] Adverse reaction to estramustine phosphate |
| 101203 | Z1Q1.11 | Administering radionuclide |
| 101391 | 7809300 | Selective internal radiotherapy microspheres lesion of liver |
| 101436 | U603314 | [X] Adverse reaction to bleomycin |
| 101693 | 8CRC.00 | Cancer chemotherapy management plan |
| 102671 | 5A59.00 | Preload radioth-urinary system |
| 102672 | 5A14.00 | Polycythaemia irradiation |
| 102823 | 782M600 | Percutaneous brachytherapy of lesion of bile duct |
| 103302 | U603200 | [X]Antineoplast natural prod caus adverse eff in therap use |
| 103372 | 5A2..00 | Intern.radioth-unsealed source |
| 103413 | 7L1i100 | Preparation for intracavitary brachytherapy |
| 103525 | 7L1Z200 | Delivery of a fraction of interstitial radiotherapy |
| 103600 | U603118 | [X] Adverse reaction to carboplatin |
| 103872 | 7M0l.00 | Support for preparation for radiotherapy |
| 103957 | 7B3CA00 | Transurethral radiofrequency needle ablation of prostate |
| 104000 | 7L1h400 | Prep for simple radioth with imaging and simple calculation |
| 104099 | 7M0c300 | Megavoltage treatment for complex radiotherapy |
| 104142 | 8BAl.00 | Neoadjuvant chemotherapy |
| 105128 | 596Z.00 | Short dist/contact radioth NOS |
| 105129 | 5A57.00 | Preload radioth.- resp.organs |
| 105323 | 7M0c000 | Delivery fraction complex radiotherapy megavoltage machine |
| 105336 | 7L1d400 | Electrochemotherapy |
| 105610 | 7M0ly00 | Other specified support for preparation for radiotherapy |
| 105864 | ZV1C300 | [V]Personal history of chemotherapy for neoplastic disease |
| 106149 | 5933.00 | Positron therapy |
| 106334 | 5A22.00 | Radioth.: infuse - head/neck |
| 107069 | 7L1Yz00 | Radiotherapy preparation NOS |
| 107130 | 5A77.00 | Radio-chem.: into cavity |
| 107133 | 595..00 | Gamma-ray+int.dist.curietherap |
| 107175 | 5A51.00 | Preload radioth.- orbit |
| 107418 | 5A17.00 | Combined internal radiotherapy |
| 107525 | 7L1iz00 | Preparation for brachytherapy NOS |
| 107734 | 7L1d200 | Deliver simple parenteral chemother neoplas first attendance |
| 107914 | U60331D | [X] Adverse reaction to antineoplastic antibiotics NOS |
| 108138 | 7L2..00 | Radiotherapy |
| 108140 | 7L1hz00 | Preparation for external beam radiotherapy NOS |
| 108202 | 7M0c200 | Delivery fraction radiotherapy superficial orthovoltage mach |
| 108561 | 7Q0J.00 | Other chemotherapy drugs |
| 109592 | 5932.00 | Betatron electron therapy |
| 110074 | 7M0p000 | High dose rate brachytherapy |
| 110894 | SL07.00 | Antineoplastic antibiotic poisoning |

1. Hospital Episodes Statistics

| **ICD 10** | **DESCRIPTION** |
| --- | --- |
| Z08.1 | Follow-up examination after radiotherapy for malignant neoplasm |
| Z08.2 | Follow-up examination after chemotherapy for malignant neoplasm |
| Z09.1 | Follow-up examination after radiotherapy for other conditions |
| Z09.2 | Follow-up examination after chemotherapy for other conditions |
| Z51.0 | Radiotherapy session |
| Z51.1 | Chemotherapy session for neoplasm |
| Z51.2 | Other chemotherapy |
| Z54.1 | Convalescence following radiotherapy |
| Z54.2 | Convalescence following chemotherapy |
| Z29.2 | Other prophylactic chemotherapy |
| Z92.6 | Personal history of chemotherapy for neoplastic disease |

1. Office of Population Censuses and Surveys (OPCS) version 4 codes

| **Opcs** | **Description_other** | **Description** |
| --- | --- | --- |
| B022 | Destruction of pituitary gland | Implantation of radioactive substance into pituitary gland |
| C242 | Operations on lacrimal gland | Radiotherapy to lacrimal gland |
| C823 | Destruction of lesion of retina | External beam radiotherapy to lesion of retina |
| J123 | Other therapeutic percutaneous operations on liver | Selective internal radiotherapy with microspheres to lesion |
| M706 | Other operations on outlet of male bladder | Radioactive seed implantation into prostate |
| M712 | Other operations on prostate | Implantation of radioactive substance into prostate |
| P064 | Extirpation of lesion of vulva | Implantation of radioactive substance into vulva |
| P205 | Extirpation of lesion of vagina | Implantation of radioactive substance into vagina |
| Q151 | Introduction of other substance into uterine cavity | Introduction of radioactive substance into uterine cavity |
| T133 | Introduction of substance into pleural cavity | Introduction of cytotoxic substance into pleural cavity |
| T481 | Other operations on peritoneum | Introduction of radioactive substance into peritoneal cavity |
| T482 | Other operations on peritoneum | Introduction of cytotoxic substance into peritoneal cavity |
| X352 | Other intravenous injection | Intravenous chemotherapy |
| X373 | Intramuscular injection | Intramuscular chemotherapy |
| X384 | Subcutaneous injection | Subcutaneous chemotherapy |
| X651 | Radiotherapy delivery | Delivery of a fraction of total body irradiation |
| X652 | Radiotherapy delivery | Delivery of a fraction of intracavitary radiotherapy |
| X653 | Radiotherapy delivery | Delivery of a fraction of interstitial radiotherapy |
| X654 | Radiotherapy delivery | Delivery of a fraction of external beam radiotherapy nec |
| X655 | Radiotherapy delivery | Oral delivery of radiotherapy for thyroid ablation |
| X656 | Radiotherapy delivery | Delivery of a fraction of intraluminal brachytherapy |
| X658 | Radiotherapy delivery | Other specified |
| X659 | Radiotherapy delivery | Unspecified |
| X671 | Preparation for external beam radiotherapy | Preparation for intensity modulated radiation therapy |
| X672 | Preparation for external beam radiotherapy | Preparation for total body irradiation |
| X673 | Preparation for external beam radiotherapy | Preparation for hemi body irradiation |
| X674 | Preparation for external beam radiotherapy | Preparation for simple radiotherapy imaging and dosimetry |
| X675 | Preparation for external beam radiotherapy | Preparation for simple radioth imaging simple calculation |
| X676 | Preparation for external beam radiotherapy | Preparation for superficial radiotherapy simple calculation |
| X677 | Preparation for external beam radiotherapy | Preparation for complex conformal radiotherapy |
| X678 | Preparation for external beam radiotherapy | Other specified |
| X679 | Preparation for external beam radiotherapy | Unspecified |
| X721 | Delivery of chemotherapy for neoplasm | Del. Of complex chemo./neoplasm/prolonged infusional treat. |
| X722 | Delivery of chemotherapy for neoplasm | Del. Of complex parental chemo./neoplasm first attendance |
| X723 | Delivery of chemotherapy for neoplasm | Del. Of simple parental chemo./neoplasm first attendance |
| X724 | Delivery of chemotherapy for neoplasm | Del. Of subsequent element of cycle of chemo. For neoplasm |
| X728 | Delivery of chemotherapy for neoplasm | Other specified |
| X729 | Delivery of chemotherapy for neoplasm | Unspecified |
| X731 | Delivery of oral chemotherapy for neoplasm | Delivery of exclusively oral chemotherapy for neoplasm |
| X738 | Delivery of oral chemotherapy for neoplasm | Other specified |
| X739 | Delivery of oral chemotherapy for neoplasm | Unspecified |
| Y351 | Introduction of removable radioactive material into org | Introduction of radioactive caesium into organ noc |
| Y352 | Introduction of removable radioactive material into org | Introduction of iridium wire into organ noc |
| Y353 | Introduction of removable radioactive material into org | Introduction of radium into organ noc |
| Y354 | Introduction of removable radioactive material into org | Introduction of radioactive substance organ brachytherap noc |
| Y358 | Introduction of removable radioactive material into org | Other specified |
| Y359 | Introduction of removable radioactive material into org | Unspecified |
| Y363 | Introduction of non-removable material into organ noc | Radioactive seed implantation noc |
| Y364 | Introduction of non-removable material into organ noc | Introduction non-rem radioact substance organ for brach noc |
| Y911 | External beam radiotherapy | Megavoltage treatment for complex radiotherapy |
| Y912 | External beam radiotherapy | Megavoltage treatment for simple radiotherapy |
| Y913 | External beam radiotherapy | Superficial or orthovoltage treatment for radiotherapy |
| Y914 | External beam radiotherapy | Megavoltage treatment for adaptive radiotherapy |
| Y918 | External beam radiotherapy | Other specified |
| Y919 | External beam radiotherapy | Unspecified |
| Y921 | Support for preparation for radiotherapy | Technical support for preparation for radiotherapy |
| Y928 | Support for preparation for radiotherapy | Other specified |
| Y929 | Support for preparation for radiotherapy | Unspecified |

1. Oral corticosteroids

CPRD

| prodcode | productname | drugsubstancename | substancestrength | formulation |
| --- | --- | --- | --- | --- |
| 44 | prednisolone enteric coated tablets 5mg | prednisolone | 5mg | enteric coated tablets |
| 95 | prednisolone tablets 5mg | prednisolone | 5mg | tablets |
| 186 | dexamethasone elixir 0.5mg/5ml | dexamethasone | 0.5mg/5ml | elixir |
| 229 | cortisone acetate tablets 25mg | cortisone acetate | 25mg | tablets |
| 557 | prednisolone enteric coated tablets 2.5mg | prednisolone | 2.5mg | enteric coated tablets |
| 578 | prednisolone tablets 1mg | prednisolone | 1mg | tablets |
| 955 | prednisolone sodium phosphate soluble tablet 5mg | prednisolone sodium phosphate | 5mg | soluble tablet |
| 1063 | PREDNESOL tablets 5mg [SOVEREIGN] | prednisolone sodium phosphate | 5mg | tablets |
| 1280 | dexamethasone tablets 2mg | dexamethasone | 2mg | tablets |
| 1380 | ENTOCORT CR modified release capsules 3mg [ASTRAZENEC] | budesonide | 3mg | modified release capsules |
| 1709 | hydrocortisone pellets 2.5 mg loz |  |  |  |
| 1971 | BETNESOL tablets 0.5mg [FOCUS] | betamethasone sodium phosphate | 0.5mg | tablets |
| 2044 | prednisone 2.5 mg tab |  |  |  |
| 2130 | methylprednisolone tablets 4mg | methylprednisolone | 4mg | tablets |
| 2368 | prednisolone tablets 2.5mg | prednisolone | 2.5mg | tablets |
| 2390 | prednisolone e/c 1 mg tab |  |  |  |
| 2704 | prednisolone tablets 25mg | prednisolone | 25mg | tablets |
| 2799 | prednisolone 10 mg tab |  |  |  |
| 2949 | prednisone tablets 5mg | prednisone | 5mg | tablets |
| 3059 | prednisolone 50 mg tab |  |  |  |
| 3345 | SINTISONE tablets [PHARMACIA] | prednisolone steaglate |  | tablets |
| 3418 | hydrocortisone tablets 10mg | hydrocortisone | 10mg | tablets |
| 3557 | prednisone tablets 1mg | prednisone | 1mg | tablets |
| 3898 | budesonide modified release capsules 3mg | budesonide | 3mg | modified release capsules |
| 3969 | dexamethasone 8 mg tab |  |  |  |
| 3992 | deflazacort tablets 6mg | deflazacort | 6mg | tablets |
| 4535 | hydrocortisone tablets 20mg | hydrocortisone | 20mg | tablets |
| 4779 | dexamethasone tablets 0.5mg | dexamethasone | 0.5mg | tablets |
| 4943 | dexamethasone sugar free oral solution 2mg/5ml | dexamethasone sodium phosphate | 2mg/5ml | sugar free oral solution |
| 5157 | dexamethasone oral solution 2mg/5ml | dexamethasone | 2mg/5ml | oral solution |
| 5490 | DELTACORTRIL ENTERIC tablets 5mg [ALLIANCE] | prednisolone | 5mg | tablets |
| 5913 | DELTACORTRIL ENTERIC tablets 2.5mg [ALLIANCE] | prednisolone | 2.5mg | tablets |
| 6095 | budesonide capsules 3mg | budesonide | 3mg | capsules |
| 6098 | HYDROCORTONE tablets 10mg [M S D] | hydrocortisone | 10mg | tablets |
| 7286 | betamethasone sodium phosphate soluble tablet 500micrograms | betamethasone sodium phosphate | 500micrograms | soluble tablet |
| 7548 | cortisone acetate capsules 5mg | cortisone acetate | 5mg | capsules |
| 7584 | prednisolone 4 mg tab |  |  |  |
| 7710 | prednisolone 15 mg tab |  |  |  |
| 7934 | prednisone 30 mg tab |  |  |  |
| 8261 | MEDRONE tablets 16mg [PHARMACIA] | methylprednisolone | 16mg | tablets |
| 9375 | deflazacort tablets 1mg | deflazacort | 1mg | tablets |
| 9727 | prednisolone tablets 50mg | prednisolone | 50mg | tablets |
| 9994 | DECADRON tablets 0.5mg [M S D] | dexamethasone | 0.5mg | tablets |
| 10552 | methylprednisolone tablets 16mg | methylprednisolone | 16mg | tablets |
| 10574 | cortisone acetate tablets 5mg | cortisone acetate | 5mg | tablets |
| 10683 | MEDRONE tablets 2mg [PHARMACIA] | methylprednisolone | 2mg | tablets |
| 10684 | methylprednisolone tablets 2mg | methylprednisolone | 2mg | tablets |
| 10754 | HYDROCORTISTAB tablets 20mg [WAYMADE] | hydrocortisone | 20mg | tablets |
| 10864 | betamethasone tablets 500micrograms | betamethasone | 500micrograms | tablets |
| 11149 | BETNELAN tablets 0.5mg [FOCUS] | betamethasone | 0.5mg | tablets |
| 12398 | CORTELAN tablets 25mg [GLAXO] | cortisone acetate | 25mg | tablets |
| 12400 | CORTISYL tablets 25mg [AVENTIS] | cortisone acetate | 25mg | tablets |
| 13043 | HYDROCORTONE tablets 20mg [M S D] | hydrocortisone | 20mg | tablets |
| 13522 | prednisolone 2 mg tab |  |  |  |
| 13615 | prednisone 10 mg tab |  |  |  |
| 14076 | hydrocortisone sugar free oral suspension 5mg/5ml | hydrocortisone | 5mg/5ml | sugar free oral suspension |
| 14172 | methylprednisolone tablets 100mg | methylprednisolone | 100mg | tablets |
| 15471 | hydrocortisone 25 mg tab |  |  |  |
| 15555 | MEDRONE tablets 4mg [PHARMACIA] | methylprednisolone | 4mg | tablets |
| 15617 | LEDERCORT tablets 4mg [WYETH PHAR] | triamcinolone | 4mg | tablets |
| 16525 | BUDENOFALK capsules 3mg [DR FALK] | budesonide | 3mg | capsules |
| 16724 | prednisone 50 mg tab |  |  |  |
| 17101 | dexamethasone 750 mcg tab |  |  |  |
| 17410 | deflazacort tablets 30mg | deflazacort | 30mg | tablets |
| 18042 | MEDRONE tablets 100mg [PHARMACIA] | methylprednisolone | 100mg | tablets |
| 18637 | CORTISTAB tablets 25mg [WAYMADE] | cortisone acetate | 25mg | tablets |
| 18955 | hydrocortisone 4.5 mg loz |  |  |  |
| 19141 | PREDNISOLONE soluble tablet 5mg [SOVEREIGN] | prednisolone sodium phosphate | 5mg | soluble tablet |
| 19908 | triamcinolone tablets 2mg | triamcinolone | 2mg | tablets |
| 20095 | PRECORTISYL FORTE tablets 25mg [AVENTIS] | prednisolone | 25mg | tablets |
| 20577 | CALCORT tablets 6mg [SHIRE] | deflazacort | 6mg | tablets |
| 20670 | prednisolone e/c |  |  |  |
| 20731 | hydrocortisone pellets |  |  |  |
| 21218 | DEXSOL oral solution 2mg/5ml [ROSEMONT] | dexamethasone sodium phosphate | 2mg/5ml | oral solution |
| 21417 | PREDNISOLONE tablets 5mg [HILLCROSS] | prednisolone | 5mg | tablets |
| 21465 | betamethasone .1 mg tab |  |  |  |
| 21833 | DECORTISYL tablets 5mg [ROUSSEL] | prednisone | 5mg | tablets |
| 21903 | ORADEXON-ORGANON tablets 2mg [ORGANON] | dexamethasone | 2mg | tablets |
| 22555 | CALCORT tablets 1mg [SHIRE] | deflazacort | 1mg | tablets |
| 22827 | betamethasone .1 mg pel |  |  |  |
| 23111 | triamcinolone tablets 4mg | triamcinolone | 4mg | tablets |
| 23210 | CORTISTAB tablets 5mg [WAYMADE] | cortisone acetate | 5mg | tablets |
| 23512 | PRECORTISYL tablets 5mg [HOECHSTMAR] | prednisolone | 5mg | tablets |
| 23788 | cortisone acetate 2.5 mg tab |  |  |  |
| 24014 | LEDERCORT tablets 2mg [WYETH PHAR] | triamcinolone | 2mg | tablets |
| 24716 | prednisolone e/c |  |  |  |
| 25272 | PRECORTISYL tablets 1mg [HOECHSTMAR] | prednisolone | 1mg | tablets |
| 27083 | betamethasone valerate .1 mg tab |  |  |  |
| 27720 | hydrocortisone |  |  |  |
| 27889 | prednisolone |  |  |  |
| 27959 | prednisolone |  |  |  |
| 27962 | DELTASTAB tablets 1mg [WAYMADE] | prednisolone | 1mg | tablets |
| 28375 | PREDNISOLONE enteric coated tablets 2.5mg [HILLCROSS] | prednisolone | 2.5mg | enteric coated tablets |
| 28376 | PREDNISOLONE enteric coated tablets 2.5mg [BIOREX] | prednisolone | 2.5mg | enteric coated tablets |
| 28615 | methylprednisolone l/a 4 mg cap |  |  |  |
| 28859 | DELTASTAB tablets 5mg [WAYMADE] | prednisolone | 5mg | tablets |
| 29112 | CALCORT tablets 30mg [SHIRE] | deflazacort | 30mg | tablets |
| 29322 | betamethasone loz |  |  |  |
| 29333 | PREDNISOLONE tablets 5mg [ACTAVIS] | prednisolone | 5mg | tablets |
| 30390 | deltastab 2 mg tab |  |  |  |
| 30971 | decortisyl 25 mg tab |  |  |  |
| 31327 | prednisolone steaglate tablets 6.65mg | prednisolone steaglate | 6.65mg | tablets |
| 31532 | PREDNISOLONE enteric coated tablets 5mg [HILLCROSS] | prednisolone | 5mg | enteric coated tablets |
| 32803 | PREDNISOLONE enteric coated tablets 5mg [ACTAVIS] | prednisolone | 5mg | enteric coated tablets |
| 32835 | PREDNISOLONE tablets 5mg [WOCKHARDT] | prednisolone | 5mg | tablets |
| 33639 | cortisone acetate msd 25 mg tab |  |  |  |
| 33691 | PREDNISOLONE enteric coated tablets 5mg [BIOREX] | prednisolone | 5mg | enteric coated tablets |
| 33988 | PREDNISOLONE tablets 5mg [CO-PHARMA] | prednisolone | 5mg | tablets |
| 33990 | PREDNISOLONE tablets 5mg [IVAX] | prednisolone | 5mg | tablets |
| 34109 | prednisolone enteric coated tablets 5mg | prednisolone | 5mg | enteric coated tablets |
| 34393 | PREDNISOLONE enteric coated tablets 5mg [TEVA] | prednisolone | 5mg | enteric coated tablets |
| 34404 | PREDNISOLONE tablets 1mg [ACTAVIS] | prednisolone | 1mg | tablets |
| 34452 | PREDNISOLONE tablets 1mg [HILLCROSS] | prednisolone | 1mg | tablets |
| 34461 | PREDNISOLONE enteric coated tablets 2.5mg [ACTAVIS] | prednisolone | 2.5mg | enteric coated tablets |
| 34631 | PREDNISOLONE tablets 1mg [CO-PHARMA] | prednisolone | 1mg | tablets |
| 34660 | PREDNISOLONE tablets 1mg [KENT] | prednisolone | 1mg | tablets |
| 34748 | PREDNISOLONE tablets 1mg [TEVA] | prednisolone | 1mg | tablets |
| 34781 | PREDNISOLONE tablets 5mg [KENT] | prednisolone | 5mg | tablets |
| 34801 | DEXAMETHASONE elixir 0.5mg/5ml [ROSEMONT] | dexamethasone | 0.5mg/5ml | elixir |
| 34880 | DEXAMETHASONE tablets 2mg [ORGANON] | dexamethasone | 2mg | tablets |
| 34914 | PREDNISOLONE tablets 1mg [CELLTECH] | prednisolone | 1mg | tablets |
| 34915 | DEXAMETHASONE tablets 0.5mg [ORGANON] | dexamethasone | 0.5mg | tablets |
| 34978 | PREDNISOLONE tablets 1mg [WOCKHARDT] | prednisolone | 1mg | tablets |
| 36055 | DEXAMETHASONE tablets 2mg [HILLCROSS] | dexamethasone | 2mg | tablets |
| 36686 | cortisone acetate msd 5 mg tab |  |  |  |
| 37203 | beclometasone gastro-resistant modified release tablets 5mg | beclometasone dipropionate | 5mg | gastro-resistant modified release tablets |
| 38022 | hydrocortisone oral suspension 10mg/5ml | hydrocortisone | 10mg/5ml | oral suspension |
| 38054 | hydrocortisone tablets | hydrocortisone |  | tablets |
| 38407 | prednisolone (roi) tablets 20mg | prednisolone | 20mg | tablets |
| 39067 | CLIPPER gastro-resistant modified release tablets 5mg [CHIESI] | beclometasone dipropionate | 5mg | gastro-resistant modified release tablets |
| 41335 | CALCORT tablets 6mg [SANOFI/AVE] | deflazacort | 6mg | tablets |
| 41515 | PREDNISOLONE tablets 5mg [TEVA] | prednisolone | 5mg | tablets |
| 41745 | PREDNISOLONE tablets 25mg [WINTHROP] | prednisolone | 25mg | tablets |
| 43544 | PREDNISONE tablets 5mg [KNOLL] | prednisone | 5mg | tablets |
| 44380 | prednisone modified release tablet 1mg | prednisone | 1mg | modified release tablet |
| 44723 | prednisone modified release tablet 5mg | prednisone | 5mg | modified release tablet |
| 44802 | LODOTRA modified release tablet 5mg [NAPPPHARM] | prednisone | 5mg | modified release tablet |
| 44803 | LODOTRA modified release tablet 2mg [NAPPPHARM] | prednisone | 2mg | modified release tablet |
| 45234 | dexamethasone capsules | dexamethasone |  | capsules |
| 45302 | PREDNISOLONE tablets 5mg [BIOREX] | prednisolone | 5mg | tablets |
| 46711 | prednisone modified release tablet 2mg | prednisone | 2mg | modified release tablet |
| 47142 | Prednisolone 5mg Soluble tablet (Amdipharm Plc) | Prednisolone sodium phosphate | 5mg | Soluble tablet |
| 47225 | Budesonide 9mg gastro-resistant granules sachets | Budesonide | 9mg | Gastro-resistant granules |
| 48088 | Budenofalk 9mg gastro-resistant granules sachets (Dr. Falk Pharma UK Ltd) | Budesonide | 9mg | Gastro-resistant granules |
| 50225 | Betnesol 500microgram soluble tablets (Waymade Healthcare Plc) | Betamethasone sodium phosphate | 500microgram | Soluble tablet |
| 51722 | Hydrocortisone 5mg/5ml oral suspension | Hydrocortisone | 1mg/1ml | Oral suspension |
| 51753 | Prednisolone 1mg tablets (Co-Pharma Ltd) | Prednisolone | 1mg | Tablet |
| 51824 | Hydrocortisone 5mg/5ml oral suspension sugar free | Hydrocortisone | 1mg/1ml | Oral suspension |
| 51849 | Hydrocortisone 1mg/5ml oral suspension |  |  |  |
| 51871 | Hydrocortisone 2mg capsules |  |  |  |
| 51872 | Hydrocortisone 2.5mg capsules | Hydrocortisone | 2.5mg | Capsule |
| 51997 | Budesonide 9mg gastro-resistant granules sachets | Budesonide | 9mg | Gastro-resistant granules |
| 52053 | Hydrocortisone 3mg/5ml oral suspension |  |  |  |
| 52396 | Dexamethasone 1mg/5ml oral solution | Dexamethasone | 200microgram/1ml | Oral solution |
| 53143 | Cortisone 25mg tablets (A A H Pharmaceuticals Ltd) | Cortisone acetate | 25mg | Tablet |
| 53207 | Dexamethasone tablets | Dexamethasone |  |  |
| 53313 | Prednisolone 20mg/5ml oral suspension | Prednisolone | 4mg/1ml | Oral suspension |
| 53336 | Prednisolone 25mg tablets (A A H Pharmaceuticals Ltd) | Prednisolone | 25mg | Tablet |
| 53705 | Cortisone acetate 5mg Capsule (Martindale Pharmaceuticals Ltd) | Cortisone acetate | 5mg | Capsule |
| 53953 | Hydrocortisone 5mg modified-release tablets |  |  |  |
| 54118 | Prednisolone 25mg/5ml oral suspension | Prednisolone | 5mg/1ml | Oral suspension |
| 54432 | Lodotra 1mg modified-release tablets (Napp Pharmaceuticals Ltd) | Prednisone | 1mg | Modified-release tablet |
| 54434 | Prednisolone 2.5mg/5ml oral suspension | Prednisolone | 500microgram/1ml | Oral suspension |
| 54793 | Dexamethasone 2mg/5ml oral suspension | Dexamethasone | 400microgram/1ml | Oral suspension |
| 54794 | Hydrocortisone 20mg modified-release tablets |  |  |  |
| 55024 | Prednisolone 5mg/5ml oral solution | Prednisolone | 1mg/1ml | Oral solution |
| 55401 | Dexamethasone 500microgram tablets (A A H Pharmaceuticals Ltd) | Dexamethasone | 500microgram | Tablet |
| 55480 | Prednisolone 2.5mg gastro-resistant tablets (Alliance Pharmaceuticals Ltd) | Prednisolone | 2.5mg | Gastro-resistant tablet |
| 56144 | Budenofalk 9mg gastro-resistant granules sachets (Dr. Falk Pharma UK Ltd) | Budesonide | 9mg | Gastro-resistant granules |
| 56319 | Hydrocortisone 2.5mg muco-adhesive buccal tablets sugar free (A A H Pharmaceuticals Ltd) | Hydrocortisone sodium succinate | 2.5mg | Muco-adhesive buccal tablet |
| 56347 | Dexamethasone 5mg/5ml oral solution | Dexamethasone | 1mg/1ml | Oral solution |
| 56443 | Dexamethasone 10mg/5ml oral solution |  |  |  |
| 56891 | Prednisolone 1mg tablets (Waymade Healthcare Plc) | Prednisolone | 1mg | Tablet |
| 57931 | Hydrocortisone 20mg tablets (Teva UK Ltd) | Hydrocortisone | 20mg | Tablet |
| 58000 | Prednisolone 5mg tablets (Almus Pharmaceuticals Ltd) | Prednisolone | 5mg | Tablet |
| 58061 | Prednisone 50mg tablets | Prednisone | 50mg | Tablet |
| 58234 | Prednisolone 10mg/5ml oral solution | Prednisolone | 2mg/1ml | Oral solution |
| 58369 | Prednisolone 5mg tablets (Boston Healthcare Ltd) | Prednisolone | 5mg | Tablet |
| 58384 | Prednisolone 1mg tablets (Almus Pharmaceuticals Ltd) | Prednisolone | 1mg | Tablet |
| 58474 | Dexamethasone 2mg/5ml oral solution sugar free (A A H Pharmaceuticals Ltd) | Dexamethasone sodium phosphate | 400microgram/1ml | Oral solution |
| 58592 | Plenadren 20mg modified-release tablets (Shire Pharmaceuticals Ltd) |  |  |  |
| 58987 | Prednisolone 5mg gastro-resistant tablets (Phoenix Healthcare Distribution Ltd) | Prednisolone | 5mg | Gastro-resistant tablet |
| 59229 | Dilacort 5mg gastro-resistant tablets (Auden McKenzie (Pharma Division) Ltd) | Prednisolone | 5mg | Gastro-resistant tablet |
| 59283 | Dilacort 2.5mg gastro-resistant tablets (Auden McKenzie (Pharma Division) Ltd) | Prednisolone | 2.5mg | Gastro-resistant tablet |
| 59338 | Prednisolone 1mg/5ml oral solution | Prednisolone | 200microgram/1ml | Oral solution |
| 59418 | Plenadren 5mg modified-release tablets (Shire Pharmaceuticals Ltd) |  |  |  |
| 59912 | Prednisolone 5mg gastro-resistant tablets (Waymade Healthcare Plc) | Prednisolone | 5mg | Gastro-resistant tablet |
| 60064 | Dexamethasone 10mg/5ml oral solution sugar free |  |  |  |
| 60120 | Dexamethasone 2mg tablets (Alliance Healthcare (Distribution) Ltd) | Dexamethasone | 2mg | Tablet |
| 60421 | Prednisolone 5mg tablets (Co-Pharma Ltd) | Prednisolone | 5mg | Tablet |
| 60946 | Entocort CR 3mg capsules (Waymade Healthcare Plc) | Budesonide | 3mg | Modified-release capsule |
| 61132 | Prednisolone 1mg tablets (Boston Healthcare Ltd) | Prednisolone | 1mg | Tablet |
| 61162 | Prednisolone 5mg tablets (Waymade Healthcare Plc) | Prednisolone | 5mg | Tablet |
| 61689 | Prednisolone 5mg soluble tablets (A A H Pharmaceuticals Ltd) | Prednisolone sodium phosphate | 5mg | Soluble tablet |
| 61791 | Hydrocortisone 2.5mg muco-adhesive buccal tablets sugar free (Waymade Healthcare Plc) | Hydrocortisone sodium succinate | 2.5mg | Muco-adhesive buccal tablet |
| 62656 | Prednisone 5mg Tablet (Hillcross Pharmaceuticals Ltd) | Prednisone | 5mg | Tablet |
| 62909 | Dexamethasone 2mg tablets (A A H Pharmaceuticals Ltd) | Dexamethasone | 2mg | Tablet |
| 63066 | Prednisolone 2.5mg tablets | Prednisolone | 2.5mg | Tablet |
| 63082 | Prednisolone 20mg tablets |  |  |  |
| 63138 | Hydrocortisone 5mg/5ml oral solution |  |  |  |
| 63172 | Prednisolone 10mg tablets |  |  |  |
| 63214 | Prednisolone 5mg soluble tablets (Alliance Healthcare (Distribution) Ltd) | Prednisolone sodium phosphate | 5mg | Soluble tablet |
| 63549 | Prednisolone 1mg/ml oral solution (Logixx Pharma Solutions Ltd) | Prednisolone | 1mg/1ml | Oral solution |
| 63791 | Prednisolone 5mg/5ml oral solution unit dose |  |  |  |
| 63893 | Budesonide 9mg modified-release tablets | Budesonide | 9mg | Modified-release tablet |
| 64007 | Pevanti 10mg tablets (AMCo) | Prednisolone | 10mg | Tablet |
| 64008 | Pevanti 2.5mg tablets (AMCo) | Prednisolone | 2.5mg | Tablet |
| 64009 | Pevanti 20mg tablets (AMCo) |  |  |  |
| 64050 | Martapan 2mg/5ml oral solution (Martindale Pharmaceuticals Ltd) | Dexamethasone sodium phosphate | 400microgram/1ml | Oral solution |
| 64059 | Hydrocortisone 2.5mg/5ml oral suspension | Hydrocortisone | 500microgram/1ml | Oral suspension |
| 64128 | Pevanti 5mg tablets (AMCo) | Prednisolone | 5mg | Tablet |
| 64221 | Prednisolone 5mg/5ml oral suspension | Prednisolone | 1mg/1ml | Oral suspension |
| 64235 | Betamethasone 500microgram soluble tablets sugar free (Alliance Healthcare (Distribution) Ltd) | Betamethasone sodium phosphate | 500microgram | Soluble tablet |
| 64416 | Prednisolone 10mg/ml oral solution sugar free |  |  |  |
| 64557 | Cortiment 9mg modified-release tablets (Ferring Pharmaceuticals Ltd) | Budesonide | 9mg | Modified-release tablet |
| 64747 | Dexamethasone 2mg/5ml oral solution |  |  |  |
| 64766 | Dexamethasone 20mg/5ml oral solution sugar free | Dexamethasone sodium phosphate | 4mg/1ml | Oral solution |
| 64787 | Hydrocortisone 10mg tablets (Almus Pharmaceuticals Ltd) | Hydrocortisone | 10mg | Tablet |
| 65020 | Prednisolone 25mg/5ml oral solution |  |  |  |
| 65626 | Prednisolone 10mg/5ml oral suspension | Prednisolone | 2mg/1ml | Oral suspension |
| 65984 | Hydrocortisone 10mg tablets (Actavis UK Ltd) | Hydrocortisone | 10mg | Tablet |
| 66015 | Prednisolone Dompe 5mg/5ml oral solution unit dose (Logixx Pharma Solutions Ltd) |  |  |  |
| 66200 | Dexamethasone 2mg soluble tablets sugar free |  |  |  |
| 66287 | Dexamethasone 8mg soluble tablets sugar free |  |  |  |
| 66327 | Hydrocortisone 20mg tablets (Actavis UK Ltd) | Hydrocortisone | 20mg | Tablet |
| 66524 | Dexamethasone 4mg soluble tablets sugar free | Dexamethasone sodium phosphate | 4mg | Soluble tablet |
|  |  |  |  |  |
| 66550 | Prednisolone 5mg gastro-resistant tablets (Alliance Healthcare (Distribution) Ltd) | Prednisolone | 5mg | Gastro-resistant tablet |
| 66556 | Hydrocortisone 2.5mg muco-adhesive buccal tablets sugar free (Sigma Pharmaceuticals Plc) | Hydrocortisone sodium succinate | 2.5mg | Muco-adhesive buccal tablet |
| 66645 | Prednisolone 5mg/5ml oral solution unit dose (Logixx Pharma Solutions Ltd) |  |  |  |
| 66666 | Hydrocortisone 10mg tablets (Teva UK Ltd) | Hydrocortisone | 10mg | Tablet |
| 66724 | Dexamethasone 10mg capsules |  |  |  |
| 66914 | Prednisolone 1mg gastro-resistant tablets |  |  |  |

1. Other immunosuppressants drugs* excluding oral corticosteroids

CPRD

| prodcode | productname | drugsubstancename | substancestrength | formulation |
| --- | --- | --- | --- | --- |
| 12339 | AZAMUNE tablets 50mg [PENN] | azathioprine | 50mg | tablets |
| 6882 | adalimumab injection 40mg | adalimumab | 40mg | injection |
| 65339 | Azapress 50mg tablets (Ennogen Pharma Ltd) | Azathioprine | 50mg | Tablet |
| 55773 | Azathioprine 10mg/5ml oral suspension | Azathioprine | 2mg/1ml | Oral suspension |
| 53869 | Azathioprine 20mg/5ml oral solution | Azathioprine | 4mg/1ml | Oral solution |
| 54982 | Azathioprine 20mg/5ml oral suspension | Azathioprine | 4mg/1ml | Oral suspension |
| 63121 | Azathioprine 25mg tablets (Alliance Healthcare (Distribution) Ltd) | Azathioprine | 25mg | Tablet |
| 59006 | Azathioprine 25mg tablets (Kent Pharmaceuticals Ltd) | Azathioprine | 25mg | Tablet |
| 66003 | Azathioprine 25mg tablets (Mawdsley-Brooks & Company Ltd) | Azathioprine | 25mg | Tablet |
| 53956 | Azathioprine 50mg tablets (Almus Pharmaceuticals Ltd) | Azathioprine | 50mg | Tablet |
| 53797 | Azathioprine 50mg tablets (Arrow Generics Ltd) | Azathioprine | 50mg | Tablet |
| 58654 | Azathioprine 50mg tablets (Sandoz Ltd) | Azathioprine | 50mg | Tablet |
| 61160 | Azathioprine 50mg tablets (Tillomed Laboratories Ltd) | Azathioprine | 50mg | Tablet |
| 23850 | HUMIRA injection 40mg [ABBOTT] | adalimumab | 40mg | injection |
| 65735 | Aflibercept 2mg/50microlitres solution for injection vials | Aflibercept | 40mg/1ml | Solution for injection |
| 60484 | Eylea 2mg/50microlitres solution for injection vials (Bayer Plc) | Aflibercept | 40mg/1ml | Solution for injection |
| 51181 | Azathioprine 60mg/5ml oral solution | Azathioprine | 12mg/1ml | Oral solution |
| 770 | azathioprine capsules | azathioprine |  | capsules |
| 42273 | alemtuzumab concentrate for solution for infusion 30mg/1ml | alemtuzumab | 30mg/1ml | concentrate for solution for infusion |
| 55815 | MabCampath 30mg/1ml concentrate for solution for infusion vials (Genzyme Therapeutics Ltd) | Alemtuzumab | 30mg/1ml | Solution for infusion |
| 39115 | azathioprine capsules 10mg | azathioprine | 10mg | capsules |
| 270 | azathioprine injection 50mg/vial | azathioprine | 50mg/vial | injection |
| 55858 | Azathioprine oral solution | Azathioprine |  |  |
| 22982 | azathioprine oral solution 50mg/5ml | azathioprine | 50mg/5ml | oral solution |
| 31598 | MABCAMPATH concentrate for solution for infusion 10mg/ml [SCHERING] | alemtuzumab | 10mg/ml | concentrate for solution for infusion |
| 36792 | azathioprine oral solution 50mg/ml | azathioprine | 50mg/ml | oral solution |
| 35518 | azathioprine oral suspension 50mg/5ml | azathioprine | 50mg/5ml | oral suspension |
| 13320 | azathioprine tablets 10mg | azathioprine | 10mg | tablets |
| 451 | azathioprine tablets 25mg | azathioprine | 25mg | tablets |
| 34816 | AZATHIOPRINE tablets 25mg [GEN (UK)] | azathioprine | 25mg | tablets |
| 32101 | AZATHIOPRINE tablets 25mg [HILLCROSS] | azathioprine | 25mg | tablets |
| 571 | azathioprine tablets 50mg | azathioprine | 50mg | tablets |
| 43562 | AZATHIOPRINE tablets 50mg [ACTAVIS] | azathioprine | 50mg | tablets |
| 41670 | AZATHIOPRINE tablets 50mg [CP PHARM] | azathioprine | 50mg | tablets |
| 34451 | AZATHIOPRINE tablets 50mg [GEN (UK)] | azathioprine | 50mg | tablets |
| 34687 | AZATHIOPRINE tablets 50mg [HILLCROSS] | azathioprine | 50mg | tablets |
| 29340 | AZATHIOPRINE tablets 50mg [IVAX] | azathioprine | 50mg | tablets |
| 31215 | AZATHIOPRINE tablets 50mg [KENT] | azathioprine | 50mg | tablets |
| 41620 | AZATHIOPRINE tablets 50mg [TEVA] | azathioprine | 50mg | tablets |
| 26261 | BERKAPRINE tablets 50mg [RORER] | azathioprine | 50mg | tablets |
| 21899 | IMMUNOPRIN tablets 50mg [ASHBOURNE] | azathioprine | 50mg | tablets |
| 14395 | IMURAN injection 50mg/vial [ASPEN EURO] | azathioprine | 50mg/vial | injection |
| 30495 | IMURAN tablets 10mg [WELLCOME] | azathioprine | 10mg | tablets |
| 43077 | IMURAN tablets 25mg [ASPEN EURO] | azathioprine | 25mg | tablets |
| 671 | IMURAN tablets 25mg [WELLCOME] | azathioprine | 25mg | tablets |
| 42988 | IMURAN tablets 50mg [ASPEN EURO] | azathioprine | 50mg | tablets |
| 1899 | IMURAN tablets 50mg [WELLCOME] | azathioprine | 50mg | tablets |
| 19072 | OPRISINE tablets 50mg [OPUS] | azathioprine | 50mg | tablets |
| 3874 | busulfan tablets 2mg | busulfan | 2mg | tablets |
| 22204 | busulfan tablets 500micrograms | busulfan | 500micrograms | tablets |
| 26301 | MYLERAN tablets 2mg [WELLCOME] | busulfan | 2mg | tablets |
| 32412 | MYLERAN tablets 500micrograms [WELLCOME] | busulfan | 500micrograms | tablets |
| 65607 | Capecitabine 150mg tablets (A A H Pharmaceuticals Ltd) | Capecitabine | 150mg | Tablet |
| 7340 | capecitabine tablets 150mg | capecitabine | 150mg | tablets |
| 7341 | capecitabine tablets 500mg | capecitabine | 500mg | tablets |
| 33127 | XELODA tablets 150mg [ROCHE] | capecitabine | 150mg | tablets |
| 18063 | XELODA tablets 500mg [ROCHE] | capecitabine | 500mg | tablets |
| 61065 | Carboplatin 450mg/45ml solution for infusion vials | Carboplatin | 10mg/1ml | Solution for infusion |
| 10328 | carboplatin concentrate for solution for infusion 10mg/ml | carboplatin | 10mg/ml | concentrate for solution for infusion |
| 40781 | carboplatin concentrate for solution for infusion 150mg/15ml | carboplatin | 150mg/15ml | concentrate for solution for infusion |
| 35855 | carboplatin concentrate for solution for infusion 50mg/5ml | carboplatin | 50mg/5ml | concentrate for solution for infusion |
| 39450 | carboplatin concentrate for solution for infusion 600mg/60ml | carboplatin | 600mg/60ml | concentrate for solution for infusion |
| 24096 | carboplatin injection 150mg | carboplatin | 150mg | injection |
| 36726 | anakinra injection 100mg/0.67ml | anakinra | 100mg/0.67ml | injection |
| 32418 | KINERET injection 100mg/0.67ml [SWED ORPH] | anakinra | 100mg/0.67ml | injection |
| 42696 | THYMOGLOBULINE powder for solution for infusion 25mg [GENZYME] | antithymocyte immunoglobulin (rabbit) | 25mg | powder for solution for infusion |
| 18236 | carboplatin injection 50mg/vial | carboplatin | 50mg/vial | injection |
| 64626 | Otezla 30mg tablets (Celgene Ltd) | Apremilast | 30mg | Tablet |
| 37915 | basiliximab powder for solution for infusion 10mg | basiliximab | 10mg | powder for solution for infusion |
| 40632 | basiliximab powder for solution for infusion 20mg | basiliximab | 20mg | powder for solution for infusion |
| 41963 | PARAPLATIN concentrate for solution for infusion 10mg/ml [BRISTOL] | carboplatin | 10mg/ml | concentrate for solution for infusion |
| 37542 | PARAPLATIN injection 150mg [BRISTOL] | carboplatin | 150mg | injection |
| 41281 | carmustine implant 7.7mg | carmustine | 7.7mg | implant |
| 5600 | chlorambucil tablets 2mg | chlorambucil | 2mg | tablets |
| 8665 | chlorambucil tablets 5mg | chlorambucil | 5mg | tablets |
| 16838 | LEUKERAN tablets 2mg [WELLCOME] | chlorambucil | 2mg | tablets |
| 61351 | Simulect 10mg powder and solvent for solution for injection vials (Novartis Pharmaceuticals UK Ltd) | Basiliximab | 10mg | Powder and solvent for solution for injection |
| 26315 | LEUKERAN tablets 5mg [WELLCOME] | chlorambucil | 5mg | tablets |
| 28709 | chlormethine injection 10mg | chlormethine hydrochloride | 10mg | injection |
| 26119 | CHLORMETHINE injection 10mg/ml [SOVEREIGN] | chlormethine hydrochloride | 10mg/ml | injection |
| 47102 | Capimune 50mg capsules (Generics (UK) Ltd) | Ciclosporin | 50mg | Capsule |
| 46395 | CAPIMUNE capsules 100mg [GEN (UK)] | ciclosporin | 100mg | capsules |
| 46637 | CAPIMUNE capsules 25mg [GEN (UK)] | ciclosporin | 25mg | capsules |
| 63798 | Ciclosporin 100mg capsules (A A H Pharmaceuticals Ltd) | Ciclosporin | 100mg | Capsule |
| 59250 | Ciclosporin 100mg capsules (Colorama Pharmaceuticals Ltd) | Ciclosporin | 100mg | Capsule |
| 54975 | Ciclosporin 100mg capsules (Cubic Pharmaceuticals Ltd) | Ciclosporin | 100mg | Capsule |
| 65639 | Ciclosporin 100mg capsules (J M McGill Ltd) | Ciclosporin | 100mg | Capsule |
| 48556 | Ciclosporin 100mg capsules (Phoenix Healthcare Distribution Ltd) | Ciclosporin | 100mg | Capsule |
| 54134 | Ciclosporin 100mg capsules (Sigma Pharmaceuticals Plc) | Ciclosporin | 100mg | Capsule |
| 55116 | Ciclosporin 25mg capsules (Cubic Pharmaceuticals Ltd) | Ciclosporin | 25mg | Capsule |
| 62051 | Ciclosporin 25mg capsules (Niche Pharma Ltd) | Ciclosporin | 25mg | Capsule |
| 48763 | Ciclosporin 25mg capsules (Phoenix Healthcare Distribution Ltd) | Ciclosporin | 25mg | Capsule |
| 52743 | Ciclosporin 25mg capsules (Sigma Pharmaceuticals Plc) | Ciclosporin | 25mg | Capsule |
| 59249 | Ciclosporin 50mg capsules (Colorama Pharmaceuticals Ltd) | Ciclosporin | 50mg | Capsule |
| 54974 | Ciclosporin 50mg capsules (Cubic Pharmaceuticals Ltd) | Ciclosporin | 50mg | Capsule |
| 48798 | Ciclosporin 50mg capsules (Phoenix Healthcare Distribution Ltd) | Ciclosporin | 50mg | Capsule |
| 54867 | Ciclosporin 50mg capsules (Sigma Pharmaceuticals Plc) | Ciclosporin | 50mg | Capsule |
| 3896 | ciclosporin capsules 100mg | ciclosporin | 100mg | capsules |
| 16035 | ciclosporin capsules 10mg | ciclosporin | 10mg | capsules |
| 32614 | SIMULECT powder for solution for infusion 20mg [NOVARTIS] | basiliximab | 20mg | powder for solution for infusion |
| 2838 | ciclosporin capsules 25mg | ciclosporin | 25mg | capsules |
| 25740 | AVASTIN concentrate for solution for infusion 100mg/4ml [ROCHE] | bevacizumab | 100mg/4ml | concentrate for solution for infusion |
| 2837 | ciclosporin capsules 50mg | ciclosporin | 50mg | capsules |
| 42924 | ciclosporin concentrate for solution for infusion 250mg/5ml | ciclosporin | 250mg/5ml | concentrate for solution for infusion |
| 38056 | ciclosporin concentrate for solution for infusion 50mg/1ml | ciclosporin | 50mg/1ml | concentrate for solution for infusion |
| 19370 | ciclosporin concentrate for solution for infusion 50mg/ml | ciclosporin | 50mg/ml | concentrate for solution for infusion |
| 1626 | ciclosporin oral solution 100mg/ml | ciclosporin | 100mg/ml | oral solution |
| 42449 | DEXIMUNE capsules 100mg [DEXCEL] | ciclosporin | 100mg | capsules |
| 42637 | DEXIMUNE capsules 25mg [DEXCEL] | ciclosporin | 25mg | capsules |
| 42448 | DEXIMUNE capsules 50mg [DEXCEL] | ciclosporin | 50mg | capsules |
| 52615 | Neoral 100mg capsules (Sigma Pharmaceuticals Plc) | Ciclosporin | 100mg | Capsule |
| 49958 | Neoral 25mg capsules (Doncaster Pharmaceuticals Ltd) | Ciclosporin | 25mg | Capsule |
| 53175 | Neoral 25mg capsules (Mawdsley-Brooks & Company Ltd) | Ciclosporin | 25mg | Capsule |
| 53176 | Neoral 50mg capsules (Doncaster Pharmaceuticals Ltd) | Ciclosporin | 50mg | Capsule |
| 973 | NEORAL capsules 100mg [NOVARTIS] | ciclosporin | 100mg | capsules |
| 16137 | NEORAL capsules 10mg [NOVARTIS] | ciclosporin | 10mg | capsules |
| 972 | NEORAL capsules 25mg [NOVARTIS] | ciclosporin | 25mg | capsules |
| 4231 | NEORAL capsules 50mg [NOVARTIS] | ciclosporin | 50mg | capsules |
| 1905 | NEORAL oral solution 100mg/ml [NOVARTIS] | ciclosporin | 100mg/ml | oral solution |
| 13556 | SANDIMMUN capsules 100mg [NOVARTIS] | ciclosporin | 100mg | capsules |
| 3920 | SANDIMMUN capsules 25mg [NOVARTIS] | ciclosporin | 25mg | capsules |
| 15596 | SANDIMMUN capsules 50mg [NOVARTIS] | ciclosporin | 50mg | capsules |
| 26790 | SANDIMMUN concentrate for solution for infusion 50mg/ml [NOVARTIS] | ciclosporin | 50mg/ml | concentrate for solution for infusion |
| 13494 | SANDIMMUN sugar free solution 100mg/ml [NOVARTIS] | ciclosporin | 100mg/ml | sugar free solution |
| 64857 | Vanquoral 100mg capsules (Teva UK Ltd) | Ciclosporin | 100mg | Capsule |
| 66785 | Vanquoral 10mg capsules (Teva UK Ltd) | Ciclosporin | 10mg | Capsule |
| 66473 | Vanquoral 25mg capsules (Teva UK Ltd) | Ciclosporin | 25mg | Capsule |
| 64858 | Vanquoral 50mg capsules (Teva UK Ltd) | Ciclosporin | 50mg | Capsule |
| 38145 | bevacizumab concentrate for solution for infusion 100mg/4ml | bevacizumab | 100mg/4ml | concentrate for solution for infusion |
| 48068 | Cisplatin 100mg/100ml solution for infusion vials | Cisplatin | 1mg/1ml | Solution for infusion |
| 38453 | cisplatin concentrate for solution for infusion 10mg/10ml | cisplatin | 10mg/10ml | concentrate for solution for infusion |
| 32824 | cisplatin concentrate for solution for infusion 1mg/ml | cisplatin | 1mg/ml | concentrate for solution for infusion |
| 44388 | cisplatin concentrate for solution for infusion 50mg/50ml | cisplatin | 50mg/50ml | concentrate for solution for infusion |
| 28324 | cisplatin powder 25mg/vial | cisplatin | 25mg/vial | powder |
| 38185 | cisplatin powder for concentrate for solution for infusion 50mg | cisplatin | 50mg | powder for concentrate for solution for infusion |
| 40454 | cladribine injection 10mg/5ml | cladribine | 10mg/5ml | injection |
| 38081 | ERWINASE powder for solution for injection 10000 units/vial [OPI] | crisantaspase | 10000 units/vial | powder for solution for injection |
| 47752 | Cyclophosphamide 25mg tablets | Cyclophosphamide | 25mg | Tablet |
| 26322 | cyclophosphamide injection 100mg | cyclophosphamide | 100mg | injection |
| 26066 | cyclophosphamide injection 200mg | cyclophosphamide | 200mg | injection |
| 29840 | cyclophosphamide powder for solution for injection 1000mg | cyclophosphamide | 1000mg | powder for solution for injection |
| 16105 | cyclophosphamide powder for solution for injection 500mg | cyclophosphamide | 500mg | powder for solution for injection |
| 3984 | cyclophosphamide tablets 10mg | cyclophosphamide | 10mg | tablets |
| 3985 | cyclophosphamide tablets 50mg | cyclophosphamide | 50mg | tablets |
| 34728 | CYCLOPHOSPHAMIDE tablets 50mg [PHARMACIA] | cyclophosphamide | 50mg | tablets |
| 44309 | ENDOXANA injection 1000mg [BAXTER ONC] | cyclophosphamide | 1000mg | injection |
| 44273 | ENDOXANA injection 200mg [BAXTER ONC] | cyclophosphamide | 200mg | injection |
| 31193 | ENDOXANA tablets 10mg [BAXTER ONC] | cyclophosphamide | 10mg | tablets |
| 10729 | ENDOXANA tablets 50mg [BAXTER ONC] | cyclophosphamide | 50mg | tablets |
| 44740 | bortezomib powder for solution for injection 3.5mg | bortezomib | 3.5mg | powder for solution for injection |
| 41266 | cytarabine injection solution 1g/10ml | cytarabine | 1g/10ml | injection solution |
| 40732 | VELCADE powder for solution for injection 3.5mg [ORTHO BIO] | bortezomib | 3.5mg | powder for solution for injection |
| 45647 | canakinumab powder for solution for injection 150mg | canakinumab | 150mg | powder for solution for injection |
| 44100 | certolizumab pegol injection solution 200mg/1ml | certolizumab pegol | 200mg/1ml | injection solution |
| 43703 | CIMZIA injection solution 200mg/1ml [UCB] | certolizumab pegol | 200mg/1ml | injection solution |
| 19335 | CYTOSAR injection 500mg [PHARMACIA] | cytarabine | 500mg | injection |
| 66380 | DepoCyte 50mg/5ml suspension for injection vials (Napp Pharmaceuticals Ltd) | Cytarabine | 10mg/1ml | Suspension for injection |
| 18238 | dacarbazine powder for solution for injection 100mg | dacarbazine citrate | 100mg | powder for solution for injection |
| 28682 | dacarbazine powder for solution for injection 200mg | dacarbazine citrate | 200mg | powder for solution for injection |
| 32204 | DTIC-DOME injection 100mg/vial [BAYER] | dacarbazine citrate | 100mg/vial | injection |
| 58496 | Cosmegen Lyovac 500microgram powder for solution for injection vials (Orphan Europe (UK) Ltd) | Dactinomycin | 500microgram | Powder for solution for injection |
| 27071 | dactinomycin powder for solution for injection 500micrograms | dactinomycin | 500micrograms | powder for solution for injection |
| 11003 | CERUBIDIN powder for concentrate for solution for injection 20mg/vial [RHONE] | daunorubicin | 20mg/vial | powder for concentrate for solution for injection |
| 43805 | daunorubicin powder for concentrate for solution for injection 20mg/vial | daunorubicin | 20mg/vial | powder for concentrate for solution for injection |
| 52396 | Dexamethasone 1mg/5ml oral solution | Dexamethasone | 200microgram/1ml | Oral solution |
| 13952 | DECADRON injection 4mg/ml [MSD MORSON] | dexamethasone sodium phosphate | 4mg/ml | injection |
| 26454 | DECADRON injection 4mg/ml [MSD MORSON] | dexamethasone sodium phosphate | 4mg/ml | injection |
| 21668 | DECADRON SHOCK PAK 20mg/ml [M S D] | dexamethasone sodium phosphate | 20mg/ml | SHOCK PAK |
| 31948 | DEXAMETHASONE injection 4mg/ml [MAYNE] | dexamethasone sodium phosphate | 4mg/ml | injection |
| 34083 | DEXAMETHASONE injection 5mg/ml [ORGANON] | dexamethasone sodium phosphate | 5mg/ml | injection |
| 19259 | dexamethasone injection 8mg/2ml | dexamethasone sodium phosphate | 8mg/2ml | injection |
| 26300 | dexamethasone shock treatment pack 20mg/ml | dexamethasone sodium phosphate | 20mg/ml | shock treatment pack |
| 28215 | dexamethasone sodium phosphate injection 120mg/5ml | dexamethasone sodium phosphate | 120mg/5ml | injection |
| 4233 | dexamethasone sodium phosphate injection 4mg/ml | dexamethasone sodium phosphate | 4mg/ml | injection |
| 13972 | dexamethasone sodium phosphate injection 5mg/ml | dexamethasone sodium phosphate | 5mg/ml | injection |
| 11334 | dexamethasone sodium phosphate IV injection 4mg/ml | dexamethasone sodium phosphate | 4mg/ml | IV injection |
| 47755 | Docetaxel 160mg/16ml solution for infusion vials | Docetaxel | 160mg/16ml | Concentrate For Solution For Infusion |
| 36831 | docetaxel concentrate for dilution for infusion solution 20mg/0.5ml | docetaxel | 20mg/0.5ml | concentrate for dilution for infusion solution |
| 38999 | docetaxel concentrate for dilution for infusion solution 80mg/2ml | docetaxel | 80mg/2ml | concentrate for dilution for infusion solution |
| 33560 | docetaxel concentrate for intravenous infusion 40mg/ml | docetaxel | 40mg/ml | concentrate for intravenous infusion |
| 44425 | docetaxel concentrate for solution for infusion 20mg/1ml | docetaxel | 20mg/1ml | concentrate for solution for infusion |
| 23849 | TAXOTERE concentrate for intravenous infusion 40mg/ml [AVENTIS] | docetaxel | 40mg/ml | concentrate for intravenous infusion |
| 36552 | TAXOTERE concentrate for solution for infusion 20mg/0.5ml [AVENTIS] | docetaxel | 20mg/0.5ml | concentrate for solution for infusion |
| 44087 | TAXOTERE concentrate for solution for infusion 20mg/1ml [AVENTIS] | docetaxel | 20mg/1ml | concentrate for solution for infusion |
| 26680 | ADRIAMYCIN injection 10mg/vial [PHARMACIA] | doxorubicin hydrochloride | 10mg/vial | injection |
| 27469 | ADRIAMYCIN injection 50mg/vial [PHARMACIA] | doxorubicin hydrochloride | 50mg/vial | injection |
| 42817 | daclizumab concentrate for solution for infusion 25mg/5ml | daclizumab | 25mg/5ml | concentrate for solution for infusion |
| 42390 | dasatinib tablets 100mg | dasatinib | 100mg | tablets |
| 37238 | dasatinib tablets 20mg | dasatinib | 20mg | tablets |
| 36062 | dasatinib tablets 50mg | dasatinib | 50mg | tablets |
| 36957 | dasatinib tablets 70mg | dasatinib | 70mg | tablets |
| 66774 | Benepali 50mg/1ml solution for injection pre-filled syringes (Biogen Idec Ltd) | Etanercept | 50mg/1ml | Solution for injection |
| 61373 | Enbrel 50mg/1ml solution for injection pre-filled MyClic pen (Pfizer Ltd) | Etanercept | 50mg/1ml | Solution for injection |
| 37784 | CAELYX concentrate for solution for infusion 20mg/10ml [JANSSEN] | doxorubicin hydrochloride | 20mg/10ml | concentrate for solution for infusion |
| 24997 | CAELYX concentrate for solution for infusion 2mg/ml [SCHERING-P] | doxorubicin hydrochloride | 2mg/ml | concentrate for solution for infusion |
| 40250 | CAELYX concentrate for solution for infusion 50mg/25ml [JANSSEN] | doxorubicin hydrochloride | 50mg/25ml | concentrate for solution for infusion |
| 37942 | doxorubicin citrate liposomal complex powder for concentrate for solution for infusion 50mg | doxorubicin hydrochloride | 50mg | powder for concentrate for solution for infusion |
| 55271 | Doxorubicin encapsulated in liposomes 2mg/ml concentrate solution for infusion | Doxorubicin Hydrochloride |  |  |
| 39307 | doxorubicin injection 10mg/5ml | doxorubicin hydrochloride | 10mg/5ml | injection |
| 45026 | doxorubicin injection 200mg/100ml | doxorubicin hydrochloride | 200mg/100ml | injection |
| 26947 | doxorubicin injection 2mg/ml | doxorubicin hydrochloride | 2mg/ml | injection |
| 30836 | doxorubicin powder for solution for injection 10mg | doxorubicin hydrochloride | 10mg | powder for solution for injection |
| 33878 | MYOCET powder for concentrate for solution for infusion 50mg [CEPHALON] | doxorubicin hydrochloride | 50mg | powder for concentrate for solution for infusion |
| 56410 | Caelyx 50mg/25ml concentrate for solution for infusion vials (Janssen-Cilag Ltd) | Doxorubicin hydrochloride liposomal pegylated | 2mg/1ml | Solution for infusion |
| 31539 | epirubicin hydrochloride injection (powder) 20mg | epirubicin hydrochloride | 20mg | injection (powder) |
| 39387 | epirubicin hydrochloride injection 100mg/50ml | epirubicin hydrochloride | 100mg/50ml | injection |
| 40251 | epirubicin hydrochloride injection 200mg/100ml | epirubicin hydrochloride | 200mg/100ml | injection |
| 29652 | epirubicin hydrochloride injection 2mg/ml | epirubicin hydrochloride | 2mg/ml | injection |
| 40816 | epirubicin hydrochloride injection 50mg/25ml | epirubicin hydrochloride | 50mg/25ml | injection |
| 49856 | Enbrel 50mg/1ml solution for injection pre-filled syringes (Pfizer Ltd) | Etanercept | 50mg/1ml | Solution for injection |
| 41058 | ENBREL FOR PAEDIATRIC USE powder for solution for injection 25mg [PFIZER] | etanercept | 25mg | powder for solution for injection |
| 35419 | ENBREL injection solution 25mg [PFIZER] | etanercept | 25mg | injection solution |
| 36556 | ENBREL injection solution 50mg [PFIZER] | etanercept | 50mg | injection solution |
| 14886 | ENBREL powder for solution for injection 25mg [PFIZER] | etanercept | 25mg | powder for solution for injection |
| 19257 | ENBREL powder for solution for injection 50mg [WYETH PHAR] | etanercept | 50mg | powder for solution for injection |
| 47843 | Etanercept 10mg powder and solvent for solution for injection vials | Etanercept | 10mg | Powder For Solution For Injection |
| 28325 | epirubicin hydrochloride powder for solution for injection 50mg | epirubicin hydrochloride | 50mg | powder for solution for injection |
| 28889 | PHARMORUBICIN injection solution 2mg/ml [PHARMACIA] | epirubicin hydrochloride | 2mg/ml | injection solution |
| 43639 | PHARMORUBICIN powder for solution for injection 50mg [PHARMACIA] | epirubicin hydrochloride | 50mg | powder for solution for injection |
| 62759 | Eribulin 880micrograms/2ml solution for injection vials | Eribulin | .44mg/1ml | Solution for injection |
| 13604 | ESTRACYT capsules 140mg [PHARMACIA] | estramustine sodium phosphate | 140mg | capsules |
| 13735 | estramustine phosphate capsules 140mg | estramustine sodium phosphate | 140mg | capsules |
| 50998 | Etanercept 50mg/1ml solution for injection pre-filled syringes | Etanercept | 50mg/1ml | Solution for injection |
| 36008 | etanercept injection solution 25mg | etanercept | 25mg | injection solution |
| 35126 | etanercept injection solution 50mg | etanercept | 50mg | injection solution |
| 36263 | EPOSIN concentrate for solution for infusion 20mg/ml [MEDAC UK] | etoposide | 20mg/ml | concentrate for solution for infusion |
| 48177 | Etoposide 100mg/5ml solution for infusion vials | Etoposide | 20mg/1ml | Solution for infusion |
| 63011 | Etoposide 500mg/25ml solution for infusion vials | Etoposide | 20mg/1ml | Solution for infusion |
| 18751 | etoposide capsules 100mg | etoposide | 100mg | capsules |
| 8756 | etoposide capsules 50mg | etoposide | 50mg | capsules |
| 31115 | etoposide concentrate for solution for infusion 20mg/ml | etoposide | 20mg/ml | concentrate for solution for infusion |
| 37375 | VEPESID capsules 100mg [BRISTOL] | etoposide | 100mg | capsules |
| 29761 | VEPESID capsules 50mg [BRISTOL] | etoposide | 50mg | capsules |
| 44387 | etoposide phosphate lyophilised powder for injection 100mg | etoposide phosphate | 100mg | lyophilised powder for injection |
| 29743 | FLUDARA ORAL tablets 10mg [GENZYME] | fludarabine phosphate | 10mg | tablets |
| 24681 | fludarabine powder for solution for injection 50mg | fludarabine phosphate | 50mg | powder for solution for injection |
| 18476 | fludarabine tablets 10mg | fludarabine phosphate | 10mg | tablets |
| 18070 | Fluorouracil 250mg capsules | Fluorouracil | 250mg | Capsule |
| 15921 | etanercept powder for solution for injection 25mg | etanercept | 25mg | powder for solution for injection |
| 26387 | etanercept powder for solution for injection 50mg | etanercept | 50mg | powder for solution for injection |
| 43781 | AFINITOR tablets 10mg [NOVARTIS] | everolimus | 10mg | tablets |
| 65378 | Everolimus 500microgram tablets | Everolimus | 500microgram | Tablet |
| 46070 | everolimus tablets 5mg | everolimus | 5mg | tablets |
| 47398 | Golimumab 50mg/0.5ml solution for injection pre-filled syringes | Golimumab | 100mg/1ml | Solution for injection |
| 46370 | golimumab pre-filled pen injection solution 50mg | golimumab | 50mg | injection solution |
| 18070 | fluorouracil capsules 250mg | fluorouracil | 250mg | capsules |
| 19556 | FLUORO-URACIL capsules 250mg [CAMBRIDGE] | fluorouracil | 250mg | capsules |
| 39388 | fluorouracil injection 1g/20ml | fluorouracil | 1g/20ml | injection |
| 20229 | FLUORO-URACIL injection 25mg/ml [CAMBRIDGE] | fluorouracil | 25mg/ml | injection |
| 36575 | fluorouracil injection 50mg/ml | fluorouracil | 50mg/ml | injection |
| 58240 | Fluorouracil 2.5g/100ml solution for infusion vials | Fluorouracil sodium | 25mg/1ml | Solution for infusion |
| 55091 | Fluorouracil 500mg/10ml solution for injection vials | Fluorouracil sodium | 50mg/1ml | Solution for injection |
| 40780 | gemcitabine powder for solution for infusion 1g/vial | gemcitabine hydrochloride | 1g/vial | powder for solution for infusion |
| 33418 | gemcitabine powder for solution for infusion 200mg/vial | gemcitabine hydrochloride | 200mg/vial | powder for solution for infusion |
| 6884 | HYDREA capsules 500mg [SQUIBB] | hydroxycarbamide | 500mg | capsules |
| 47127 | Hydroxycarbamide 100mg tablets | Hydroxycarbamide | 100mg | Film Coated Tablets |
| 50565 | Hydroxycarbamide 300mg capsules | Hydroxycarbamide | 300mg | Capsule |
| 59007 | Hydroxycarbamide 500mg capsules (A A H Pharmaceuticals Ltd) | Hydroxycarbamide | 500mg | Capsule |
| 66728 | Idelalisib 100mg tablets | Idelalisib | 100mg | Tablet |
| 65085 | Zydelig 100mg tablets (Gilead Sciences International Ltd) | Idelalisib | 100mg | Tablet |
| 6333 | hydroxycarbamide capsules 500mg | hydroxycarbamide | 500mg | capsules |
| 21295 | GLIVEC capsules 100mg [NOVARTIS] | imatinib mesilate | 100mg | capsules |
| 33823 | GLIVEC tablets 100mg [NOVARTIS] | imatinib mesilate | 100mg | tablets |
| 28800 | GLIVEC tablets 400mg [NOVARTIS] | imatinib mesilate | 400mg | tablets |
| 33330 | HYDROXYCARBAMIDE capsules 500mg [MEDAC UK] | hydroxycarbamide | 500mg | capsules |
| 39548 | hydroxycarbamide film coated tablets 1000mg | hydroxycarbamide | 1000mg | film coated tablets |
| 38319 | hydroxycarbamide oral solution 500mg/5ml | hydroxycarbamide | 500mg/5ml | oral solution |
| 3873 | hydroxyurea capsules 500mg | hydroxycarbamide | 500mg | capsules |
| 65463 | Siklos 1000mg tablets (Nordic Pharma Ltd) | Hydroxycarbamide | 1gram | Tablet |
| 31339 | idarubicin hydrochloride capsules 10mg | idarubicin hydrochloride | 10mg | capsules |
| 31984 | idarubicin hydrochloride capsules 5mg | idarubicin hydrochloride | 5mg | capsules |
| 43168 | ifosfamide injection 2g/vial | ifosfamide | 2g/vial | injection |
| 21286 | imatinib capsules 100mg | imatinib mesilate | 100mg | capsules |
| 29229 | imatinib tablets 100mg | imatinib mesilate | 100mg | tablets |
| 21318 | imatinib tablets 400mg | imatinib mesilate | 400mg | tablets |
| 64636 | Inflectra 100mg powder for concentrate for solution for infusion vials (Hospira UK Ltd) | Infliximab | 100mg | Powder for solution for infusion |
| 40983 | irinotecan hydrochloride concentrate for solution for infusion 40mg/2ml | irinotecan hydrochloride | 40mg/2ml | concentrate for solution for infusion |
| 59339 | Irinotecan 40mg/2ml concentrate for solution for infusion vials (Hospira UK Ltd) | Irinotecan hydrochloride trihydrate | 20mg/1ml | Solution for infusion |
| 16822 | infliximab powder for concentrate for solution for infusion 100mg | infliximab | 100mg | powder for concentrate for solution for infusion |
| 18460 | ARAVA tablets 100mg [AVENTIS] | leflunomide | 100mg | tablets |
| 16522 | ARAVA tablets 10mg [AVENTIS] | leflunomide | 10mg | tablets |
| 17642 | ARAVA tablets 20mg [AVENTIS] | leflunomide | 20mg | tablets |
| 48217 | Leflunomide 10mg tablets (medac UK) | Leflunomide | 10mg | Tablet |
| 62993 | Leflunomide 20mg tablets (Sandoz Ltd) | Leflunomide | 20mg | Tablet |
| 4970 | leflunomide tablets 100mg | leflunomide | 100mg | tablets |
| 4971 | leflunomide tablets 10mg | leflunomide | 10mg | tablets |
| 6934 | leflunomide tablets 20mg | leflunomide | 20mg | tablets |
| 60630 | Lenalidomide 2.5mg capsules | Lenalidomide | 2.5mg | Capsule |
| 44529 | lenalidomide capsules 10mg | lenalidomide | 10mg | capsules |
| 46205 | lenalidomide capsules 15mg | lenalidomide | 15mg | capsules |
| 40626 | lenalidomide capsules 25mg | lenalidomide | 25mg | capsules |
| 42056 | lenalidomide capsules 5mg | lenalidomide | 5mg | capsules |
| 58227 | Revlimid 10mg capsules (Celgene Ltd) | Lenalidomide | 10mg | Capsule |
| 56818 | Revlimid 15mg capsules (Celgene Ltd) | Lenalidomide | 15mg | Capsule |
| 41139 | REVLIMID capsules 25mg [CELGENE] | lenalidomide | 25mg | capsules |
| 22392 | REMICADE powder for concentrate for solution for infusion 100mg [SCHERING-P] | infliximab | 100mg | powder for concentrate for solution for infusion |
| 58862 | Ipilimumab 200mg/40ml solution for infusion vials | Ipilimumab | 5mg/1ml | Solution for infusion |
| 25848 | CCNU capsules 10mg [LUNDBECK] | lomustine | 10mg | capsules |
| 8404 | CCNU capsules 40mg [LUNDBECK] | lomustine | 40mg | capsules |
| 12067 | lomustine capsules 40mg | lomustine | 40mg | capsules |
| 30014 | MITHRACIN injection 2.5mg/vial [PFIZER] | mannitol/sodium phosphate/plicamycin | 2.5mg/vial | injection |
| 31494 | mithramycin injection 2.5mg/vial | mannitol/sodium phosphate/plicamycin | 2.5mg/vial | injection |
| 26343 | ALKERAN tablets 2mg [WELLCOME] | melphalan | 2mg | tablets |
| 23270 | ALKERAN tablets 5mg [WELLCOME] | melphalan | 5mg | tablets |
| 16929 | melphalan tablets 2mg | melphalan | 2mg | tablets |
| 12150 | melphalan tablets 5mg | melphalan | 5mg | tablets |
| 26580 | melphalan injection 100mg/vial | melphalan hydrochloride | 100mg/vial | injection |
| 37099 | melphalan powder for solution for injection 50mg | melphalan hydrochloride | 50mg | powder for solution for injection |
| 57239 | Mercaptopurine 20mg/ml oral suspension | Mercaptopurine | 20mg/1ml | Oral suspension |
| 56753 | Mercaptopurine 25mg tablets | Mercaptopurine | 25mg | Tablet |
| 55772 | Mercaptopurine 25mg/5ml oral suspension | Mercaptopurine | 5mg/1ml | Oral suspension |
| 61545 | Mercaptopurine 50mg tablets (Aspen Pharma Trading Ltd) | Mercaptopurine | 50mg | Tablet |
| 52333 | Mercaptopurine 75mg/5ml oral suspension | Mercaptopurine | 15mg/1ml | Oral suspension |
| 19982 | mercaptopurine capsules 10mg | mercaptopurine | 10mg | capsules |
| 47369 | Mercaptopurine Oral solution | Mercaptopurine |  | Oral Liquid |
| 32972 | mercaptopurine tablets 10mg | mercaptopurine | 10mg | tablets |
| 3450 | mercaptopurine tablets 50mg | mercaptopurine | 50mg | tablets |
| 29675 | PURI-NETHOL tablets 50mg [ALKOPHARMA] | mercaptopurine | 50mg | tablets |
| 21753 | MAXTREX tablets 10mg [PHARMACIA] | methotrexate | 10mg | tablets |
| 13428 | MAXTREX tablets 2.5mg [PHARMACIA] | methotrexate | 2.5mg | tablets |
| 57441 | Methotrexate 10mg tablets (A A H Pharmaceuticals Ltd) | Methotrexate | 10mg | Tablet |
| 58885 | Methotrexate 10mg tablets (Sigma Pharmaceuticals Plc) | Methotrexate | 10mg | Tablet |
| 59538 | Methotrexate 10mg tablets (Teva UK Ltd) | Methotrexate | 10mg | Tablet |
| 57174 | Methotrexate 10mg tablets (Waymade Healthcare Plc) | Methotrexate | 10mg | Tablet |
| 61151 | Methotrexate 10mg/0.2ml solution for injection pre-filled disposable devices | Methotrexate | 50mg/1ml | Solution for injection |
| 56037 | Methotrexate 2.5mg tablets (A A H Pharmaceuticals Ltd) | Methotrexate | 2.5mg | Tablet |
| 51120 | Methotrexate 2.5mg tablets (Alliance Healthcare (Distribution) Ltd) | Methotrexate | 2.5mg | Tablet |
| 62833 | Methotrexate 2.5mg tablets (DE Pharmaceuticals) | Methotrexate | 2.5mg | Tablet |
| 60979 | Methotrexate 2.5mg tablets (Morningside Healthcare Ltd) | Methotrexate | 2.5mg | Tablet |
| 58303 | Methotrexate 2.5mg tablets (Orion Pharma (UK) Ltd) | Methotrexate | 2.5mg | Tablet |
| 49951 | Methotrexate 2.5mg tablets (Sandoz Ltd) | Methotrexate | 2.5mg | Tablet |
| 52606 | Methotrexate 2.5mg tablets (Sigma Pharmaceuticals Plc) | Methotrexate | 2.5mg | Tablet |
| 59685 | Methotrexate 2.5mg tablets (Teva UK Ltd) | Methotrexate | 2.5mg | Tablet |
| 53385 | Methotrexate 2.5mg tablets (Waymade Healthcare Plc) | Methotrexate | 2.5mg | Tablet |
| 61085 | Methotrexate 2.5mg tablets (Waymade Healthcare Plc) | Methotrexate | 2.5mg | Tablet |
| 61122 | Methotrexate 25mg/0.5ml solution for injection pre-filled disposable devices | Methotrexate | 50mg/1ml | Solution for injection |
| 59723 | Methotrexate 7.5mg/5ml oral solution | Methotrexate | 1.5mg/1ml | Oral solution |
| 40371 | methotrexate injection 10mg/0.2ml | methotrexate | 10mg/0.2ml | injection |
| 32865 | methotrexate injection 10mg/1ml | methotrexate | 10mg/1ml | injection |
| 46152 | methotrexate injection 12.5mg/0.25ml | methotrexate | 12.5mg/0.25ml | injection |
| 40281 | methotrexate injection 15mg/0.3ml | methotrexate | 15mg/0.3ml | injection |
| 27404 | methotrexate injection 15mg/1.5ml | methotrexate | 15mg/1.5ml | injection |
| 46156 | methotrexate injection 17.5mg/0.35ml | methotrexate | 17.5mg/0.35ml | injection |
| 40273 | methotrexate injection 20mg/0.4ml | methotrexate | 20mg/0.4ml | injection |
| 45165 | methotrexate injection 20mg/1ml | methotrexate | 20mg/1ml | injection |
| 26064 | methotrexate injection 20mg/2ml | methotrexate | 20mg/2ml | injection |
| 46129 | methotrexate injection 22.5mg/0.45ml | methotrexate | 22.5mg/0.45ml | injection |
| 40328 | methotrexate injection 25mg/0.5ml | methotrexate | 25mg/0.5ml | injection |
| 45558 | methotrexate injection 25mg/1.25ml | methotrexate | 25mg/1.25ml | injection |
| 24634 | methotrexate injection 25mg/2.5ml | methotrexate | 25mg/2.5ml | injection |
| 44908 | methotrexate injection 30mg/0.6ml | methotrexate | 30mg/0.6ml | injection |
| 46039 | methotrexate injection 30mg/1.5ml | methotrexate | 30mg/1.5ml | injection |
| 40301 | methotrexate injection 7.5mg/0.15ml | methotrexate | 7.5mg/0.15ml | injection |
| 35402 | methotrexate injection 7.5mg/0.75ml | methotrexate | 7.5mg/0.75ml | injection |
| 36800 | methotrexate oral solution 10mg/5ml | methotrexate | 10mg/5ml | oral solution |
| 36849 | methotrexate oral suspension 10mg/5ml | methotrexate | 10mg/5ml | oral suspension |
| 28041 | methotrexate oral suspension 12.5mg/5ml | methotrexate | 12.5mg/5ml | oral suspension |
| 35752 | methotrexate oral suspension 7.5mg/5ml | methotrexate | 7.5mg/5ml | oral suspension |
| 18424 | methotrexate sodium tablets 2.5mg | methotrexate | 2.5mg | tablets |
| 41585 | METHOTREXATE SODIUM tablets 2.5mg [WYETH PHAR] | methotrexate | 2.5mg | tablets |
| 17035 | methotrexate suspension 2.5mg/5ml | methotrexate | 2.5mg/5ml | suspension |
| 877 | methotrexate tablets 10mg | methotrexate | 10mg | tablets |
| 34929 | METHOTREXATE tablets 10mg [HOSPIRA] | methotrexate | 10mg | tablets |
| 823 | methotrexate tablets 2.5mg | methotrexate | 2.5mg | tablets |
| 41104 | METHOTREXATE tablets 2.5mg [CP PHARM] | methotrexate | 2.5mg | tablets |
| 20951 | METHOTREXATE tablets 2.5mg [GOLDSHIELD] | methotrexate | 2.5mg | tablets |
| 32111 | METHOTREXATE tablets 2.5mg [HOSPIRA] | methotrexate | 2.5mg | tablets |
| 30780 | METHOTREXATE tablets 2.5mg [PHARMACIA] | methotrexate | 2.5mg | tablets |
| 40356 | METOJECT injection 10mg/0.2ml [MEDAC UK] | methotrexate | 10mg/0.2ml | injection |
| 37117 | METOJECT injection 10mg/1ml [MEDAC UK] | methotrexate | 10mg/1ml | injection |
| 46098 | METOJECT injection 12.5mg/0.25ml [MEDAC UK] | methotrexate | 12.5mg/0.25ml | injection |
| 40284 | METOJECT injection 15mg/0.3ml [MEDAC UK] | methotrexate | 15mg/0.3ml | injection |
| 27400 | METOJECT injection 15mg/1.5ml [MEDAC UK] | methotrexate | 15mg/1.5ml | injection |
| 46265 | METOJECT injection 17.5mg/0.35ml [MEDAC UK] | methotrexate | 17.5mg/0.35ml | injection |
| 40292 | METOJECT injection 20mg/0.4ml [MEDAC UK] | methotrexate | 20mg/0.4ml | injection |
| 14348 | METOJECT injection 20mg/2ml [MEDAC UK] | methotrexate | 20mg/2ml | injection |
| 46197 | METOJECT injection 22.5mg/0.45ml [MEDAC UK] | methotrexate | 22.5mg/0.45ml | injection |
| 40293 | METOJECT injection 25mg/0.5ml [MEDAC UK] | methotrexate | 25mg/0.5ml | injection |
| 33601 | METOJECT injection 25mg/2.5ml [MEDAC UK] | methotrexate | 25mg/2.5ml | injection |
| 40280 | METOJECT injection 7.5mg/0.15ml [MEDAC UK] | methotrexate | 7.5mg/0.15ml | injection |
| 35865 | METOJECT injection 7.5mg/0.75ml [MEDAC UK] | methotrexate | 7.5mg/0.75ml | injection |
| 61050 | Metoject PEN 10mg/0.2ml solution for injection pre-filled pen (medac UK) | Methotrexate | 50mg/1ml | Solution for injection |
| 61171 | Metoject PEN 15mg/0.3ml solution for injection pre-filled pen (medac UK) | Methotrexate | 50mg/1ml | Solution for injection |
| 61181 | Metoject PEN 17.5mg/0.35ml solution for injection pre-filled pen (medac UK) | Methotrexate | 50mg/1ml | Solution for injection |
| 61180 | Metoject PEN 20mg/0.4ml solution for injection pre-filled pen (medac UK) | Methotrexate | 50mg/1ml | Solution for injection |
| 61169 | Metoject PEN 25mg/0.5ml solution for injection pre-filled pen (medac UK) | Methotrexate | 50mg/1ml | Solution for injection |
| 62421 | Metoject PEN 27.5mg/0.55ml solution for injection pre-filled pen (medac UK) | Methotrexate | 50mg/1ml | Solution for injection |
| 61488 | Metoject PEN 30mg/0.6ml solution for injection pre-filled pen (medac UK) | Methotrexate | 50mg/1ml | Solution for injection |
| 27342 | MAXTREX injection 2.5mg/ml [PHARMACIA] | methotrexate sodium | 2.5mg/ml | injection |
| 51667 | Methotrexate 200mg/8ml solution for injection vials | Methotrexate sodium | 25mg/1ml | Solution for injection |
| 65584 | Methotrexate 2mg/ml oral solution sugar free | Methotrexate sodium | 2mg/1ml | Oral solution |
| 51321 | Methotrexate 50mg/2ml solution for injection vials | Methotrexate sodium | 25mg/1ml | Solution for injection |
| 53696 | Methotrexate 50mg/2ml solution for injection vials (A A H Pharmaceuticals Ltd) | Methotrexate sodium | 25mg/1ml | Solution for injection |
| 49547 | Methotrexate 5g/200ml solution for infusion vials | Methotrexate sodium | 25mg/1ml | Solution for infusion |
| 36167 | methotrexate injection 1000mg/10ml | methotrexate sodium | 1000mg/10ml | injection |
| 46407 | methotrexate injection 1000mg/40ml | methotrexate sodium | 1000mg/40ml | injection |
| 12816 | methotrexate injection 100mg/ml | methotrexate sodium | 100mg/ml | injection |
| 7337 | methotrexate injection 10mg/0.4ml | methotrexate sodium | 10mg/0.4ml | injection |
| 7336 | methotrexate injection 12.5mg/0.5ml | methotrexate sodium | 12.5mg/0.5ml | injection |
| 16540 | methotrexate injection 15mg/0.6ml | methotrexate sodium | 15mg/0.6ml | injection |
| 18890 | methotrexate injection 17.5mg/0.7ml | methotrexate sodium | 17.5mg/0.7ml | injection |
| 14347 | methotrexate injection 20mg/0.8ml | methotrexate sodium | 20mg/0.8ml | injection |
| 34258 | METHOTREXATE injection 20mg/0.8ml [CENT HOME] | methotrexate sodium | 20mg/0.8ml | injection |
| 17672 | methotrexate injection 22.5mg/0.9ml | methotrexate sodium | 22.5mg/0.9ml | injection |
| 16519 | methotrexate injection 25mg/1ml | methotrexate sodium | 25mg/1ml | injection |
| 8583 | methotrexate injection 25mg/ml | methotrexate sodium | 25mg/ml | injection |
| 27642 | methotrexate injection 27.5mg/1.1ml | methotrexate sodium | 27.5mg/1.1ml | injection |
| 30703 | methotrexate injection 30mg/1.2ml | methotrexate sodium | 30mg/1.2ml | injection |
| 41086 | methotrexate injection 5000mg/50ml | methotrexate sodium | 5000mg/50ml | injection |
| 32229 | methotrexate injection 500mg/20ml | methotrexate sodium | 500mg/20ml | injection |
| 24783 | methotrexate injection 50mg/2ml | methotrexate sodium | 50mg/2ml | injection |
| 8327 | methotrexate injection 50mg/3ml | methotrexate sodium | 50mg/3ml | injection |
| 30932 | methotrexate injection 5mg/0.2ml | methotrexate sodium | 5mg/0.2ml | injection |
| 9528 | methotrexate injection 5mg/2ml | methotrexate sodium | 5mg/2ml | injection |
| 16570 | methotrexate injection 7.5mg/0.3ml | methotrexate sodium | 7.5mg/0.3ml | injection |
| 14748 | methotrexate sodium injection 25mg/ml | methotrexate sodium | 25mg/ml | injection |
| 29069 | methotrexate sterile powder 500mg/vial | methotrexate sodium | 500mg/vial | sterile powder |
| 57103 | Metoject 27.5mg/0.55ml solution for injection pre-filled syringes (medac UK) | Methotrexate sodium | 50mg/1ml | Solution for injection |
| 66487 | Zlatal 12.5mg/0.5ml solution for injection pre-filled syringes (Nordic Pharma Ltd) | Methotrexate sodium | 25mg/1ml | Solution for injection |
| 37396 | MYELOBROMOL tablets 125mg [DURBIN] | mitobronitol | 125mg | tablets |
| 44478 | LYSODREN tablets 500mg [LAB HRA] | mitotane | 500mg | tablets |
| 35826 | mitotane tablets 500mg | mitotane | 500mg | tablets |
| 39366 | mitoxantrone concentrate for solution for infusion 10mg/5ml | mitoxantrone hydrochloride | 10mg/5ml | concentrate for solution for infusion |
| 41267 | mitoxantrone concentrate for solution for infusion 20mg/10ml | mitoxantrone hydrochloride | 20mg/10ml | concentrate for solution for infusion |
| 33174 | mitoxantrone concentrate for solution for infusion 2mg/ml | mitoxantrone hydrochloride | 2mg/ml | concentrate for solution for infusion |
| 15405 | NOVANTRONE concentrate for solution for infusion 2mg/ml [WYETH PHAR] | mitoxantrone hydrochloride | 2mg/ml | concentrate for solution for infusion |
| 45393 | ARZIP tablets 500mg [WINTHROP] | mycophenolate mofetil | 500mg | tablets |
| 57593 | CellCept 500mg tablets (Waymade Healthcare Plc) | Mycophenolate mofetil | 500mg | Tablet |
| 16919 | CELLCEPT capsules 250mg [ROCHE] | mycophenolate mofetil | 250mg | capsules |
| 21732 | CELLCEPT oral suspension 1g/5ml [ROCHE] | mycophenolate mofetil | 1g/5ml | oral suspension |
| 30581 | CELLCEPT powder for concentrate for solution for infusion 500mg [ROCHE] | mycophenolate mofetil | 500mg | powder for concentrate for solution for infusion |
| 18804 | CELLCEPT tablets 500mg [ROCHE] | mycophenolate mofetil | 500mg | tablets |
| 57272 | Mycophenolate mofetil 125mg/5ml oral suspension | Mycophenolate mofetil | 25mg/1ml | Oral suspension |
| 60231 | Mycophenolate mofetil 250mg capsules (A A H Pharmaceuticals Ltd) | Mycophenolate mofetil | 250mg | Capsule |
| 54317 | Mycophenolate mofetil 250mg capsules (Sandoz Ltd) | Mycophenolate mofetil | 250mg | Capsule |
| 53255 | Mycophenolate mofetil 250mg capsules (Sigma Pharmaceuticals Plc) | Mycophenolate mofetil | 250mg | Capsule |
| 58530 | Mycophenolate mofetil 500mg tablets (Sigma Pharmaceuticals Plc) | Mycophenolate mofetil | 500mg | Tablet |
| 4438 | mycophenolate mofetil capsules 250mg | mycophenolate mofetil | 250mg | capsules |
| 16879 | mycophenolate mofetil oral suspension 1g/5ml | mycophenolate mofetil | 1g/5ml | oral suspension |
| 7077 | mycophenolate mofetil powder for concentrate for solution for infusion 500mg | mycophenolate mofetil | 500mg | powder for concentrate for solution for infusion |
| 4230 | mycophenolate mofetil tablets 500mg | mycophenolate mofetil | 500mg | tablets |
| 50669 | Mycophenolate motefil 500mg tablets (Sandoz Ltd) | Mycophenolate mofetil | 500mg | Tablet |
| 47746 | Mycophenolate motefil 500mg tablets (Wockhardt UK Ltd) | Mycophenolate mofetil | 500mg | Tablet |
| 45489 | MYFENAX capsules 250mg [TEVA] | mycophenolate mofetil | 250mg | capsules |
| 45043 | MYFENAX tablets 500mg [TEVA] | mycophenolate mofetil | 500mg | tablets |
| 35301 | mycophenolic acid gastro-resistant tablets 180mg | mycophenolate sodium | 180mg | gastro-resistant tablets |
| 26097 | mycophenolic acid gastro-resistant tablets 360mg | mycophenolate sodium | 360mg | gastro-resistant tablets |
| 27290 | MYFORTIC tablets 180mg [NOVARTIS] | mycophenolate sodium | 180mg | tablets |
| 27289 | MYFORTIC tablets 360mg [NOVARTIS] | mycophenolate sodium | 360mg | tablets |
| 23832 | ELOXATIN powder for concentrate for solution for infusion 100mg [SANOFI S] | oxaliplatin | 100mg | powder for concentrate for solution for infusion |
| 50277 | Oxaliplatin 100mg/20ml concentrate for solution for infusion vials (A A H Pharmaceuticals Ltd) | Oxaliplatin | 5mg/1ml | Solution for infusion |
| 39895 | oxaliplatin concentrate for solution for infusion 100mg/20ml | oxaliplatin | 100mg/20ml | concentrate for solution for infusion |
| 39553 | oxaliplatin concentrate for solution for infusion 50mg/10ml | oxaliplatin | 50mg/10ml | concentrate for solution for infusion |
| 36714 | oxaliplatin powder for concentrate for solution for infusion 100mg | oxaliplatin | 100mg | powder for concentrate for solution for infusion |
| 27293 | oxaliplatin powder for concentrate for solution for infusion 50mg | oxaliplatin | 50mg | powder for concentrate for solution for infusion |
| 41191 | natalizumab concentrate for solution for infusion 300mg/15ml | natalizumab | 300mg/15ml | concentrate for solution for infusion |
| 47754 | Paclitaxel 100mg/16.7ml solution for infusion vials | Paclitaxel | 6mg/1ml | Solution for infusion |
| 39919 | paclitaxel albumin bound powder for suspension for infusion 100mg | paclitaxel | 100mg | powder for suspension for infusion |
| 45147 | paclitaxel concentrate for solution for infusion 150mg/25ml | paclitaxel | 150mg/25ml | concentrate for solution for infusion |
| 35854 | paclitaxel concentrate for solution for infusion 30mg/5ml | paclitaxel | 30mg/5ml | concentrate for solution for infusion |
| 14381 | paclitaxel concentrate for solution for infusion 6mg/ml | paclitaxel | 6mg/ml | concentrate for solution for infusion |
| 35384 | TAXOL concentrate for solution for infusion 30mg/5ml [BMS] | paclitaxel | 30mg/5ml | concentrate for solution for infusion |
| 16173 | TAXOL concentrate for solution for infusion 6mg/ml [BMS] | paclitaxel | 6mg/ml | concentrate for solution for infusion |
| 61366 | Abraxane 100mg powder for suspension for infusion vials (Celgene Ltd) | Paclitaxel albumin | 100mg | Powder for suspension for infusion |
| 37272 | pemetrexed powder for concentrate for solution for infusion 500mg | pemetrexed disodium | 500mg | powder for concentrate for solution for infusion |
| 43888 | pentostatin powder for solution for injection 10mg | pentostatin (deoxycoformycin) | 10mg | powder for solution for injection |
| 38254 | TYSABRI concentrate for solution for infusion 300mg/15ml [BIOGEN] | natalizumab | 300mg/15ml | concentrate for solution for infusion |
| 45820 | nilotinib capsules 150mg | nilotinib hydrochloride monohydrate | 150mg | capsules |
| 28605 | NATULAN capsules 50mg [CAMBRIDGE] | procarbazine hydrochloride | 50mg | capsules |
| 17186 | procarbazine capsules 50mg | procarbazine hydrochloride | 50mg | capsules |
| 38317 | nilotinib capsules 200mg | nilotinib hydrochloride monohydrate | 200mg | capsules |
| 39111 | rituximab concentrate for intravenous infusion 10mg/ml | rituximab | 10mg/ml | concentrate for intravenous infusion |
| 44222 | raltitrexed powder for concentrate for solution for infusion 2mg | raltitrexed | 2mg | powder for concentrate for solution for infusion |
| 22640 | razoxane tablets 125mg | razoxane | 125mg | tablets |
| 12066 | RAZOXIN tablets 125mg [CAMBRIDGE] | razoxane | 125mg | tablets |
| 33728 | RAPAMUNE oral solution 1mg/ml [PFIZER] | sirolimus | 1mg/ml | oral solution |
| 23289 | RAPAMUNE tablets 1mg [PFIZER] | sirolimus | 1mg | tablets |
| 28999 | RAPAMUNE tablets 2mg [PFIZER] | sirolimus | 2mg | tablets |
| 20097 | sirolimus oral solution 1mg/ml | sirolimus | 1mg/ml | oral solution |
| 6600 | sirolimus tablets 1mg | sirolimus | 1mg | tablets |
| 6484 | sirolimus tablets 2mg | sirolimus | 2mg | tablets |
| 44783 | sirolimus tablets 500 micrograms | sirolimus | 500 micrograms | tablets |
| 65704 | Adoport 0.75mg capsules (Sandoz Ltd) | Tacrolimus | 750microgram | Capsule |
| 63866 | Adoport 2mg capsules (Sandoz Ltd) | Tacrolimus | 2mg | Capsule |
| 44804 | ADOPORT twice daily capsules 1mg [SANDOZ] | tacrolimus | 1mg | twice daily capsules |
| 44640 | ADOPORT twice daily capsules 500 micrograms [SANDOZ] | tacrolimus | 500 micrograms | twice daily capsules |
| 28490 | rituximab concentrate for solution for infusion 100mg/10ml | rituximab | 100mg/10ml | concentrate for solution for infusion |
| 44641 | ADOPORT twice daily capsules 5mg [SANDOZ] | tacrolimus | 5mg | twice daily capsules |
| 37506 | ADVAGRAF once daily modified release capsules 1mg [ASTELLAS] | tacrolimus | 1mg | once daily modified release capsules |
| 40765 | ADVAGRAF once daily modified release capsules 3mg [ASTELLAS] | tacrolimus | 3mg | once daily modified release capsules |
| 39633 | ADVAGRAF once daily modified release capsules 500 micrograms [ASTELLAS] | tacrolimus | 500 micrograms | once daily modified release capsules |
| 38919 | ADVAGRAF once daily modified release capsules 5mg [ASTELLAS] | tacrolimus | 5mg | once daily modified release capsules |
| 46325 | MODIGRAF granules for oral suspension 1mg [ASTELLAS] | tacrolimus | 1mg | granules for oral suspension |
| 46324 | MODIGRAF granules for oral suspension 200micrograms [ASTELLAS] | tacrolimus | 200micrograms | granules for oral suspension |
| 54198 | Prograf 1mg capsules (Lexon (UK) Ltd) | Tacrolimus | 1mg | Capsule |
| 3683 | PROGRAF twice daily capsules 1mg [ASTELLAS] | tacrolimus | 1mg | twice daily capsules |
| 5870 | PROGRAF twice daily capsules 500 micrograms [ASTELLAS] | tacrolimus | 500 micrograms | twice daily capsules |
| 13271 | PROGRAF twice daily capsules 5mg [ASTELLAS] | tacrolimus | 5mg | twice daily capsules |
| 55066 | Tacrolimus 2.5mg/5ml oral solution | Tacrolimus | 500microgram/1ml | Oral solution |
| 63924 | Tacrolimus 2mg capsules | Tacrolimus | 2mg | Capsule |
| 54048 | Tacrolimus 500micrograms/5ml oral suspension | Tacrolimus | 100microgram/1ml | Oral suspension |
| 63720 | Tacrolimus 750microgram capsules | Tacrolimus | 750microgram | Capsule |
| 36294 | rituximab concentrate for solution for infusion 500mg/50ml | rituximab | 500mg/50ml | concentrate for solution for infusion |
| 33123 | tacrolimus concentrate for solution for infusion 5mg/1ml | tacrolimus | 5mg/1ml | concentrate for solution for infusion |
| 43081 | tacrolimus granules for oral suspension 1mg | tacrolimus | 1mg | granules for oral suspension |
| 43082 | tacrolimus granules for oral suspension 200micrograms | tacrolimus | 200micrograms | granules for oral suspension |
| 40453 | TORISEL concentrate for solution for infusion 30mg/1.2ml [PFIZER] | temsirolimus | 30mg/1.2ml | concentrate for solution for infusion |
| 62957 | Tocilizumab 400mg/20ml solution for infusion vials | Tocilizumab | 20mg/1ml | Solution for infusion |
| 46348 | tocilizumab concentrate for solution for infusion 200mg/10ml | tocilizumab | 200mg/10ml | concentrate for solution for infusion |
| 37985 | tacrolimus once daily modified release capsules 1mg | tacrolimus | 1mg | once daily modified release capsules |
| 40964 | tacrolimus once daily modified release capsules 3mg | tacrolimus | 3mg | once daily modified release capsules |
| 38113 | tacrolimus once daily modified release capsules 500 micrograms | tacrolimus | 500 micrograms | once daily modified release capsules |
| 38989 | tacrolimus once daily modified release capsules 5mg | tacrolimus | 5mg | once daily modified release capsules |
| 44926 | tacrolimus oral suspension 2.5mg/5ml | tacrolimus | 2.5mg/5ml | oral suspension |
| 37155 | tacrolimus suspension 1mg/ml | tacrolimus | 1mg/ml | suspension |
| 41502 | tocilizumab concentrate for solution for infusion 80mg/4ml | tocilizumab | 80mg/4ml | concentrate for solution for infusion |
| 64612 | Apremilast 30mg tablets |  |  |  |
| 2839 | tacrolimus twice daily capsules 1mg | tacrolimus | 1mg | twice daily capsules |
| 6495 | tacrolimus twice daily capsules 500 micrograms | tacrolimus | 500 micrograms | twice daily capsules |
| 5089 | tacrolimus twice daily capsules 5mg | tacrolimus | 5mg | twice daily capsules |
| 55010 | Vivadex 5mg capsules (Dexcel-Pharma Ltd) | Tacrolimus | 5mg | Capsule |
| 33519 | UFTORAL capsules 224mg + 100mg [MERCK SER] | tegafur/uracil | 224mg + 100mg | capsules |
| 33520 | uracil with tegafur capsules 224mg + 100mg | tegafur/uracil | 224mg + 100mg | capsules |
| 55077 | Temodal 100mg capsules (Merck Sharp & Dohme Ltd) | Temozolomide | 100mg | Capsule |
| 35226 | TEMODAL capsules 20mg [SCHERING-P] | temozolomide | 20mg | capsules |
| 29700 | TEMODAL capsules 250mg [SCHERING-P] | temozolomide | 250mg | capsules |
| 33803 | TEMODAL capsules 5mg [SCHERING-P] | temozolomide | 5mg | capsules |
| 21249 | temozolomide capsules 100mg | temozolomide | 100mg | capsules |
| 42372 | temozolomide capsules 140mg | temozolomide | 140mg | capsules |
| 32490 | temozolomide capsules 20mg | temozolomide | 20mg | capsules |
| 21250 | temozolomide capsules 250mg | temozolomide | 250mg | capsules |
| 27922 | temozolomide capsules 5mg | temozolomide | 5mg | capsules |
| 33385 | thiotepa powder for solution for injection 15mg | thiotepa | 15mg | powder for solution for injection |
| 26502 | LANVIS tablets 40mg [ALKOPHARMA] | tioguanine | 40mg | tablets |
| 20094 | tioguanine tablets 40mg | tioguanine | 40mg | tablets |
| 33227 | HYCAMTIN powder for concentrate for solution for infusion 4mg [GLAXSK PHA] | topotecan hydrochloride | 4mg | powder for concentrate for solution for infusion |
| 47396 | Topotecan 1mg capsules | Topotecan hydrochloride | 1mg | Capsule |
| 41960 | topotecan powder for concentrate for solution for infusion 1mg | topotecan hydrochloride | 1mg | powder for concentrate for solution for infusion |
| 59362 | Trabectedin 1mg powder for solution for infusion vials | Trabectedin | 1mg | Powder for solution for infusion |
| 24448 | treosulfan capsules 250mg | treosulfan | 250mg | capsules |
| 23871 | TREOSULFAN capsules 250mg [FARILLON] | treosulfan | 250mg | capsules |
| 32604 | vinblastine sulphate injection 10mg/10ml | vinblastine sulphate | 10mg/10ml | injection |
| 31223 | vinblastine sulphate injection 10mg/vial | vinblastine sulphate | 10mg/vial | injection |
| 42684 | vinorelbine concentrate for solution for infusion 10mg/1ml | vinorelbine | 10mg/1ml | concentrate for solution for infusion |
| 33171 | vinorelbine injection solution 10mg/ml | vinorelbine | 10mg/ml | injection solution |
| 46838 | Vinorelbine 80mg capsules | Vinorelbine Tartrate | 80mg | Capsules |
| 32774 | vinorelbine capsules 20mg | vinorelbine tartrate | 20mg | capsules |
| 60112 | Erivedge 150mg capsules (Roche Products Ltd) | Vismodegib | 150mg | Capsule |
| 65140 | Vismodegib 150mg capsules | Vismodegib | 150mg | Capsule |
| 59842 | Azacitidine 100mg powder for suspension for injection vials |  |  |  |
| 3918 | AZATHIOPRINE 10 MG TAB |  |  |  |
| 8776 | AZATHIOPRINE 100 MG TAB |  |  |  |
| 15556 | AZATHIOPRINE 125 MG TAB |  |  |  |
| 52921 | Azathioprine 125mg/5ml oral suspension |  |  |  |
| 58142 | Brentuximab vedotin 50mg powder for solution for infusion vials |  |  |  |
| 17206 | AZATHIOPRINE 50 MG SUS |  |  |  |
| 66436 | Cetuximab 500mg/100ml solution for infusion vials |  |  |  |
| 50494 | Etanercept 50mg/1ml solution for injection 1ml pre-filled disposable devices |  |  |  |
| 58819 | Herceptin 600mg/5ml solution for injection vials (Roche Products Ltd) |  |  |  |
| 20721 | CCNU |  |  |  |
| 26806 | DECADRON 2 MG TAB |  |  |  |
| 64427 | Idelalisib 150mg tablets |  |  |  |
| 62157 | Hydroxycarbamide 500mg/5ml oral suspension |  |  |  |
| 1901 | IMURAN 10 MG TAB |  |  |  |
| 62007 | Leflunomide 15mg tablets |  |  |  |
| 65629 | Mercaptopurine 30mg capsules |  |  |  |
| 66583 | Mercaptopurine 75mg tablets |  |  |  |
| 56041 | Ipilimumab 50mg/10ml solution for infusion vials |  |  |  |
| 27579 | METHOTREXATE |  |  |  |
| 61081 | Methotrexate 12.5mg/0.25ml solution for injection pre-filled disposable devices |  |  |  |
| 61273 | Methotrexate 15mg/0.3ml solution for injection pre-filled disposable devices |  |  |  |
| 61137 | Methotrexate 17.5mg/0.35ml solution for injection pre-filled disposable devices |  |  |  |
| 61140 | Methotrexate 20mg/0.4ml solution for injection pre-filled disposable devices |  |  |  |
| 61082 | Methotrexate 22.5mg/0.45ml solution for injection pre-filled disposable devices |  |  |  |
| 65531 | Otezla tablets treatment initiation pack (Celgene Ltd) |  |  |  |
| 21889 | METHOTREXATE 25MG/1ML |  |  |  |
| 62753 | Methotrexate 27.5mg/0.55ml solution for injection pre-filled syringes |  |  |  |
| 61796 | Methotrexate 30mg/0.6ml solution for injection pre-filled disposable devices |  |  |  |
| 61172 | Methotrexate 7.5mg/0.15ml solution for injection pre-filled disposable devices |  |  |  |
| 61211 | Metoject PEN 12.5mg/0.25ml solution for injection pre-filled pen (medac UK) |  |  |  |
| 61419 | Metoject PEN 22.5mg/0.45ml solution for injection pre-filled pen (medac UK) |  |  |  |
| 61178 | Metoject PEN 7.5mg/0.15ml solution for injection pre-filled pen (medac UK) |  |  |  |
| 37395 | MYELOBROMOL (NAMED PATIENT ONLY) 125 MG TAB |  |  |  |
| 63210 | Rapamune 0.5mg tablets (Pfizer Ltd) |  |  |  |
| 64152 | Tacrolimus 1mg modified-release tablets |  |  |  |
| 26332 | THIOTEPA 15 MG INJ |  |  |  |
| 62009 | Trabectedin 250microgram powder for solution for infusion vials |  |  |  |
| 28726 | TREOSULFAN LEO |  |  |  |
| 61908 | Pertuzumab 420mg/14ml solution for infusion vials |  |  |  |
