# Supplementary material for: Inequalities in zoster disease burden: a population‐based cohort study to identify social determinants using linked data from the U.K. Clinical Practice Research Datalink
Source: Br J Dermatol. 2018 Apr 19;178(6):1324–30. doi: 10.1111/bjd.16399 (PMC6033149; doi:10.1111/bjd.16399)
Supplement: Supplementary file 4 — Appendix S2 Conceptual hierarchical framework for the association of social factors with zoster disease burden. [file BJD-178-1324-s004.docx]

Appendix S4 Identification of exposures, co-morbidities and medications in CPRD and HES

**4a)** **Exposure identification in CPRD and HES**

Both CPRD and HES were utilised to gather information for seven exposures: ethnicity, religion, immigration status, living alone, care home residence, marital status and cohabitation. Immigration status was defined using both immigrant and language codes (Appendix 3). Information using code list for these variables was accrued using the Clinical, Referral, Immunisation and Test files. Additionally, information using CPRD’s Consultation and Additional Clinical Details files was obtained using specific code (Consultation type 30 and 31 and Entity type 132 and 98). A pre-existing code list as used by Mathur *et al* was used for ethnicity.^1^ For time-invariant variables: ethnicity, religion and immigration status, individuals with multiple records showing inconsistent information were treated as follows: the most commonly recorded information for that variable was chosen and if the information was equally common, the preference was given to the most recent information.

For time varying exposures, the date of the exposure was defined by using event date (date when Read code was recorded by the GP). System date (date when the event was recorded on the GP database) was used if event dates were missing.^2^

Patients would have had recordings for these variables over multiple GP contact. The observations with discordant information for a particular time varying variable for an individual on same date were excluded. Care home residence, immigration status, living alone and cohabitation individuals were categorised as binary variables.

Additionally, information for living alone, cohabitation and marital status was also sought from family number in GP electronic data that aids in identification of individuals sharing same households.^3^ In order to identify individuals in CPRD sharing same household as the study patient, all individuals had to share family number with the study patient and be actively registered (the later of their current registration date or practice up to standard date was before the start of follow-up date for the study participant and the earliest of their date of death, transfer out date, or practice last collection date were after the start of follow-up date for the study participant) at the study patient’s start of follow-up date. The age of household members was ascertained on the study patient’s start of follow-up date. As the family number may not be updated [personal communication via email CPRD Knowledge Team] the information from family number was only used with information was unavailable from the other CPRD files. Study patients sharing family number with ≥1 individuals were identified as not living alone. Study patient in household size of two or three residing with another individual with age difference of ≤15 years were said to be cohabiting as long as the age difference between the cohabiting couple and the other occupant (if present) was >15 years. Study patients identified as cohabiting were assigned the marital status as `partner uncategorised’.

**4b)** **Co-morbidities and medications**

The specific Read codes identifying these conditions in CPRD and ICD-10 codes in HES were compiled to generate code lists (Appendix 3). Individuals without a relevant code for co-morbidity or medications were assumed not to have that condition. The Office of Population Censuses and Surveys (OPCS) version 4 codes for procedures were also used for to identify: Hematopoietic stem cell transplant, organ transplant and radiotherapy in HES. If an individual had codes for conditions in both CPRD and HES the date of the earliest record was taken. Asthma and Chronic Obstructive Pulmonary Disease codes were combined together.

References:

1. Mathur R, Bhaskaran K, Chaturvedi N, Leon DA, vanStaa T, Grundy E, et al. Completeness and usability of ethnicity data in UK-based primary care and hospital databases. J Public Health. 2014 Dec;36(4):684-92

2. Clinical Practice Research Datalink. Clinical Practice Research Datalink [11/09/2017]. Available from: <https://www.cprd.com/intro.asp>.

3. Carbonari DM, Saine ME, Newcomb CW, Blak B, Roy JA, Haynes K, et al. Use of demographic and pharmacy data to identify patients included within both the Clinical Practice Research Datalink (CPRD) and The Health Improvement Network (THIN). Pharmacoepidemiol Drug Saf. 2015;24(9):999-1003
