# Supplementary material for: Inequalities in zoster disease burden: a population‐based cohort study to identify social determinants using linked data from the U.K. Clinical Practice Research Datalink
Source: Br J Dermatol. 2018 Apr 19;178(6):1324–30. doi: 10.1111/bjd.16399 (PMC6033149; doi:10.1111/bjd.16399)
Supplement: Supplementary file 5 — Appendix S3 Code lists: social factors, comorbidities and medications. [file BJD-178-1324-s005.docx]

Appendix S5 Immunosuppressive medications and conditions: defining periods of immunosuppression

| **Therapy^#^** | **Dose criteria** | **Duration Immuno-suppressed prior to first documented script in electronic health records** | **Duration Immuno-suppressed after every record** |
| --- | --- | --- | --- |
| Biological agents (e.g. Anti-TNF therapy) | Immunosuppressed any doses | 3 months (as the therapy is generally initiated in hospitals) | 12 months |
| Azathiaprine | Immunosuppressed at dose of ≥50mg/daily assuming the worst case scenario of TPMT carrier^1, 2^ | 3 months (as the therapy is generally initiated in hospitals) | 3 months |
| Methotrexate | Immunosuppressed at dose of>25mg per week (>3.57 mg/day) | 3 months (as the therapy is generally initiated in hospitals) | 3 months |
| 6-mercaptopurine | Immunosuppressed at dose of ≥45mg/daily (assuming the worst case scenario of TPMT carrier)^1, 2^ | 3 months (as the therapy is generally initiated in hospitals) | 3 months |
| Other immunosuppressive agents such as tacrolimus, sirolimus | Immunosuppressed any dose | 3 months (as the therapy is generally initiated in hospitals) | 3 months |
| Other dmards  such as ciclosporin, mycophenolate, leflunomide | Immunosuppressed any dose | 3 months (as the therapy is generally initiated in hospitals) | 3 months |
| Injectable or oral steroids | Immunosuppressed : >40 mg/daily for >7 days OR >20mg/ daily for >14 days^3^ | 3 months (as the therapy is generally initiated in hospitals) | 3 months |
| Cancer chemotherapy or radiotherapy | Immunosuppressed any dose | 3 months (as the therapy is generally initiated in hospitals) | 12 months |
| Lymphoma, myeloma, other plasma cell dyscrasias, leukemia, stem cell transplant, bone marrow transplant | Not applicable | - | 24 months |
| Solid organ transplants, HIV & Cellular immune deficiency | Not applicable | - | Immuno-suppressed for life |

^#^ Assumptions for prescriptions: (i) Any overlap between prescriptions take the latest prescription, truncating the previous one to end when the overlapping new one begins (ii) If 2 same products scripts on same day but different strengths: add these together (iii) If 2 same products scripts on same day but same strengths assume error and choose only one TNF tumour necrosis factor TPMT thiopurine methyltransferase DMARD disease modifying anti-rheumatic drugs

References:

1. British Society of Gastroenterology. AZATHIOPRINE / 6 MERCAPTOPURINE [16/02/2017]. Available from: <www.bsg.org.uk/pdf_word_docs/aza_ibd_dr.doc>.

2. Ford LT, Berg JD. Thiopurine S-methyltransferase (TPMT) assessment prior to starting thiopurine drug treatment; a pharmacogenomic test whose time has come. J Clin Pathol. 2010;63(4):288-95

3. Public Health England. Chapter 28a : Shingles . In: Immunisation against infectious disease. The Green Book. 2016 [16/04/2017]. Available from: <https://www.gov.uk/government/publications/shingles-herpes-zoster-the-green-book-chapter-28a>.
