# Supplementary material for: Inequalities in zoster disease burden: a population‐based cohort study to identify social determinants using linked data from the U.K. Clinical Practice Research Datalink
Source: Br J Dermatol. 2018 Apr 19;178(6):1324–30. doi: 10.1111/bjd.16399 (PMC6033149; doi:10.1111/bjd.16399)
Supplement: Supplementary file 6 — Appendix S4 Identification of exposures, comorbidities and medications in Clinical Practice Research Datalink and Hospital Episode Statistics. [file BJD-178-1324-s006.docx]

Appendix S6 Inclusion of explanatory variables in causal modelling based on hierarchical framework

(Based on^1^)

| **Model** | **Explanatory variable** | **Interpretation** |
| --- | --- | --- |
| Adjusted for *a priori* confounders | All variables adjusted for *a priori* confounders: age, sex and calendar period | Effect estimate of each variable adjusted for *a* *priori* confounders |
| Model-1* | Ethnicity and immigration status with *a priori* confounders | Effects of ethnicity and immigration status adjusted for *a priori* confounders and each other |
| Model-2* | Model-1+IMD | Effects of ethnicity and immigration status not mediated via IMD & adjusted for *a priori* confounders and each other  Effects of IMD adjusted for other variables in the model and *a priori* confounders* |
| Model-3* | Model-2 + living alone+ care home residence | Effects of ethnicity and immigration status not mediated via living alone and care home residence, adjusted for other variables in the model and *a priori* confounders*  Effect of IMD not mediated via living alone and care home residence, adjusted for other variables in the model and *a priori* confounders*  Effect of living alone+ and care home residence adjusted for other variables in the model and *a priori* confounders* |
| Model-4* | Model-3 + co-morbidities~ | Effect of ethnicity and immigration status, IMD, living alone and care home residence not mediated via co-morbidities*  Effect of co-morbidities adjusted for other variables in the model and *a priori* confounders* |
| Model-5* | Model-4 + medications^#^ | Effect of ethnicity and immigration status, IMD, living alone, care home residence and co-morbidities not mediated via medications*  Effect of medication adjusted for other variables in the model and *a priori* confounders* |

*all variables in the model adjusted for each other and *a priori* confounders: age, sex and calendar period

IMD index of multiple deprivation

~co-morbidities included systemic lupus erythematosus, rheumatoid arthritis, inflammatory bowel disease, chronic obstructive pulmonary disease, asthma, chronic kidney disease, diabetes, HIV, cellular immune deficiency, acute and chronic leukemia, lymphoma, myeloma and other haematological malignancies including plasma cell dyscrasias, solid organ transplant and bone marrow transplant

^#^ steroids (oral or injectable), radiotherapy and chemotherapy, biological therapy, disease modifying anti-rheumatic drugs (DMARDs): azathioprine, methotrexate, and other DMARDs), other immunosuppressive drugs such as tacrolimus

References:

1. Victora CG, Huttly SR, Fuchs SC, Olinto MT. The role of conceptual frameworks in epidemiological analysis: a hierarchical approach. Int J Epidemiol. 1997 Feb;26(1):224-7
