# Supplementary material for: Inequalities in zoster disease burden: a population‐based cohort study to identify social determinants using linked data from the U.K. Clinical Practice Research Datalink
Source: Br J Dermatol. 2018 Apr 19;178(6):1324–30. doi: 10.1111/bjd.16399 (PMC6033149; doi:10.1111/bjd.16399)
Supplement: Supplementary file 7 — Appendix S5 Immunosuppressive medications and conditions: defining periods of immunosuppression. [file BJD-178-1324-s007.docx]

Appendix S7 Comparison of patients excluded due to prior history of zoster and patients included in the study

| Characteristics | | Excluded individuals from study due to prior history of zoster N=69360 | Included in study N=862470 |
| --- | --- | --- | --- |
| Median age in years at start of the study date (01/09/2003) (interquartile range) | | 70.2 (61.2-79.2) | 68.2 (61.2-76.2) |
| Sex | Male | 27,296 (39.4%) | 389,264 (45.1%) |
|  | Female | 42,064 (60.6) | 473,204 (54.9%) |
|  | Missing | - | 2 (0.0002%) |
| Patient-level IMD^~+^ | 1 (least deprived) | 16542 (23.9%) | 201684 (23.4%) |
|  | 2 | 17933 (25.8%) | 220924 (25.6%) |
|  | 3 | 14705 (21.2%) | 181648 (21.1%) |
|  | 4 | 12200 (17.6%) | 158865 (18.4%) |
|  | 5 (most deprived) | 7980 (11.5%) | 99349 (11.5%) |
| Immigration status | Not immigrant | 68916 (99.4%) | 853123 (98.9%) |
|  | Immigrant | 444 (0.6%) | 9347 (1.1%) |
| Ethnicity | White | 58022 (83.6%) | 684870 (79.4%) |
|  | South Asian | 597 (0.9%) | 12273 (1.4%) |
|  | Black | 213 (0.3%) | 7176 (0.8%) |
|  | Other | 385 (0.6%) | 5850 (0.7%) |
|  | Mixed | 72 (0.1%) | 1422 (0.2%) |
|  | Missing | 10071 (14.5%) | 150879 (17.5%) |

IMD index of multiple deprivation ^~^ for excluded group 42 (0.06%) missing values replaced by practice IMD ^+^ for included group 849 (0.1%) missing values replaced by practice IMD
