# Supplementary material for: Inequalities in zoster disease burden: a population‐based cohort study to identify social determinants using linked data from the U.K. Clinical Practice Research Datalink
Source: Br J Dermatol. 2018 Apr 19;178(6):1324–30. doi: 10.1111/bjd.16399 (PMC6033149; doi:10.1111/bjd.16399)
Supplement: Supplementary file 8 — Appendix S6 Inclusion of explanatory variables in causal modelling based on a hierarchical framework. [file BJD-178-1324-s008.docx]

Appendix S8 Baseline characteristics of patients excluded from analysis due to missing data for ethnicity and included in complete case analysis

|  | | **Patients excluded from complete case analysis^*^** N=150,878 outcome=4555 (3%) Incidence per 1,000 pyr (95%CI) 7.08 (6.88-7.29)  Median age (years) at start of follow-up (IQR) 68.2 (65-77.2)  Median age (years) at current registration date (IQR) 54.5 (40.7-65.2) | | | **Patients included in complete case analysis** N=711,590, outcome=32,459 (4.6%) Incidence per 1,000 pyr (95%CI)= 9.09 (9.0-9.20)  Median age (years)at start of follow-up (IQR) 69.6 (65-77.8)  Median age (years) at current registration date (IQR) 57.8 (43.6-67.7) | | |
| --- | --- | --- | --- | --- | --- | --- | --- |
|  |  | ***Total (column %)*** | ***No outcome (row %)*** | ***With outcome (row %)*** | ***Total (column %)*** | ***No outcome (row %)*** | ***With outcome (row %)*** |
| Age at start of follow-up (years) | 65-69 | 83822 (55.6%) | 81529 (97.3%) | 2293 (2.7%) | 358677 (50.4%) | 344991 (96.2%) | 13686 (3.8%) |
|  | 70-74 | 20483 (13.6%) | 19610 (95.7%) | 873 (4.3%) | 115668 (16.3%) | 108699 (94%) | 6969 (6%) |
|  | 75-79 | 15803 (10.5%) | 15173 (96%) | 630 (4%) | 96499 (13.6%) | 90771 (94.1%) | 5728 (5.9%) |
|  | 80-84 | 13997 (9.3%) | 13568 (96.9%) | 429 (3.1%) | 75828 (10.7%) | 71965 (94.9%) | 3863 (5.1%) |
|  | 85 & above | 16773 (11.1%) | 16443 (98%) | 330 (2%) | 64918 (9.1%) | 62705 (96.6%) | 2213 (3.4%) |
| Sex | Male | 69563 (46.1%) | 67808 (97.5%) | 1755 (2.5%) | 319701 (44.9%) | 306685 (95.9%) | 13016 (4.1%) |
|  | Female | 81315 (53.9%) | 78515 (96.6%) | 2800 (3.4%) | 391889 (55.1%) | 372446 (95%) | 19443 (5%) |
| Ethnicity | White | Missing | - | - | 684869 (96.2%) | 653080 (95.4%) | 31789 (4.6%) |
|  | South Asian | Missing | - | - | 12273 (1.7%) | 11951 (97.4%) | 322 (2.6%) |
|  | Black | Missing | - | - | 7176 (1%) | 7048 (98.2%) | 128 (1.8%) |
|  | Other | Missing | - | - | 5850 (0.8%) | 5672 (97%) | 178 (3%) |
|  | Mixed | Missing | - | - | 1422 (0.2%) | 1380 (97%) | 42 (3%) |
| Immigration status | Not immigrant | 150493 (99.7%) | 145943 (97%) | 4550 (3%) | 702628 (98.7%) | 670393 (95.4%) | 32235 (4.6%) |
|  | Immigrant | 385 (0.3%) | 380 (98.7%) | 5 (1.3%) | 8962 (1.3%) | 8738 (97.5%) | 224 (2.5%) |
| Patient-level IMD^~+^ | 1 (least deprived) | 43002 (28.5%) | 41528 (96.6%) | 1474 (3.4%) | 158682 (22.3%) | 151015 (95.2%) | 7667 (4.8%) |
|  | 2 | 38525 (25.5%) | 37304 (96.8%) | 1221 (3.2%) | 182399 (25.6%) | 173830 (95.3%) | 8569 (4.7%) |
|  | 3 | 30681 (20.3%) | 29743 (96.9%) | 938 (3.1%) | 150966 (21.2%) | 144233 (95.5%) | 6733 (4.5%) |
|  | 4 | 24951 (16.5%) | 24334 (97.5%) | 617 (2.5%) | 133913 (18.8%) | 128045 (95.6%) | 5868 (4.4%) |
|  | 5 (most deprived) | 13719 (9.1%) | 13414 (97.8%) | 305 (2.2%) | 85630 (12%) | 82008 (95.8%) | 3622 (4.2%) |
| Practice-level IMD | 1 (least deprived) | 27600 (18.3%) | 26611 (96.4%) | 989 (3.6%) | 101574 (14.3%) | 96462 (95%) | 5112 (5%) |
|  | 2 | 34219 (22.7%) | 33174 (96.9%) | 1045 (3.1%) | 163530 (23%) | 156155 (95.5%) | 7375 (4.5%) |
|  | 3 | 36234 (24%) | 35084 (96.8%) | 1150 (3.2%) | 159492 (22.4%) | 152151 (95.4%) | 7341 (4.6%) |
|  | 4 | 30206 (20%) | 29429 (97.4%) | 777 (2.6%) | 155544 (21.9%) | 148672 (95.6%) | 6872 (4.4%) |
|  | 5 (most deprived) | 22619 (15%) | 22025 (97.4%) | 594 (2.6%) | 131450 (18.5%) | 125691 (95.6%) | 5759 (4.4%) |
| Lived alone^ | No | 94153 (62.4%) | 91134 (96.8%) | 3019 (3.2%) | 469693 (66%) | 447925 (95.4%) | 21768 (4.6%) |
|  | Yes | 56725 (37.6%) | 55189 (97.3%) | 1536 (2.7%) | 241897 (34%) | 231206 (95.6%) | 10691 (4.4%) |
| Cohabiting^ | No | 86967 (57.6%) | 84630 (97.3%) | 2337 (2.7%) | 384524 (54%) | 368040 (95.7%) | 16484 (4.3%) |
|  | Yes | 63911 (42.4%) | 61693 (96.5%) | 2218 (3.5%) | 327066 (46%) | 311091 (95.1%) | 15975 (4.9%) |
| Care home^ | No | 142765 (94.6%) | 138370 (96.9%) | 4395 (3.1%) | 653967 (91.9%) | 622930 (95.3%) | 31037 (4.7%) |
|  | Yes | 8113 (5.4%) | 7953 (98%) | 160 (2%) | 57623 (8.1%) | 56201 (97.5%) | 1422 (2.5%) |
| Rheumatoid arthritis^ | No | 148501 (98.4%) | 144047 (97%) | 4454 (3%) | 689035 (96.8%) | 657765 (95.5%) | 31270 (4.5%) |
|  | Yes | 2377 (1.6%) | 2276 (95.8%) | 101 (4.2%) | 22555 (3.2%) | 21366 (94.7%) | 1189 (5.3%) |
| Systemic Lupus Erythematosus^^^ | No | 150712 (99.9%) | 146163 (97%) | 4549 (3%) | 710274 (99.8%) | 677900 (95.4%) | 32374 (4.6%) |
|  | Yes | 166 (0.1%) | 160 (96.4%) | 6 (3.6%) | 1316 (0.2%) | 1231 (93.5%) | 85 (6.5%) |
| Inflammatory bowel disease^ | No | 149776 (99.3%) | 145268 (97%) | 4508 (3%) | 700505 (98.4%) | 668596 (95.4%) | 31909 (4.6%) |
|  | Yes | 1102 (0.7%) | 1055 (95.7%) | 47 (4.3%) | 11085 (1.6%) | 10535 (95%) | 550 (5%) |
| Diabetes mellitus^^^ | No | 133054 (88.2%) | 129003 (97%) | 4051 (3%) | 578619 (81.3%) | 551335 (95.3%) | 27284 (4.7%) |
|  | Yes | 17824 (11.8%) | 17320 (97.2%) | 504 (2.8%) | 132971 (18.7%) | 127796 (96.1%) | 5175 (3.9%) |
| Chronic kidney disease^^^ | No | 131782 (87.3%) | 127735 (96.9%) | 4047 (3.1%) | 542165 (76.2%) | 515649 (95.1%) | 26516 (4.9%) |
|  | Yes | 19096 (12.7%) | 18588 (97.3%) | 508 (2.7%) | 169425 (23.8%) | 163482 (96.5%) | 5943 (3.5%) |
| COPD/ asthma^^^ | No | 131132 (86.9%) | 127240 (97%) | 3892 (3%) | 557917 (78.4%) | 532444 (95.4%) | 25473 (4.6%) |
|  | Yes | 19746 (13.1%) | 19083 (96.6%) | 663 (3.4%) | 153673 (21.6%) | 146687 (95.5%) | 6986 (4.5%) |
| HIV infection^^^ | No | 150860 (100%) | 146305 (97%) | 4555 (3%) | 711457 (100%) | 679004 (95.4%) | 32453 (4.6%) |
|  | Yes | 18 (0%) | 18 (100%) | 0 (0%) | 133 (0%) | 127 (95.5%) | 6 (4.5%) |
| Cellular immune deficiency^^^ | No | 150631 (99.8%) | 146081 (97%) | 4550 (3%) | 708715 (99.6%) | 676333 (95.4%) | 32382 (4.6%) |
|  | Yes | 247 (0.2%) | 242 (98%) | 5 (2%) | 2875 (0.4%) | 2798 (97.3%) | 77 (2.7%) |
| Solid organ transplant^^^ | No | 150795 (99.9%) | 146240 (97%) | 4555 (3%) | 710593 (99.9%) | 678199 (95.4%) | 32394 (4.6%) |
|  | Yes | 83 (0.1%) | 83 (100%) | 0 (0%) | 997 (0.1%) | 932 (93.5%) | 65 (6.5%) |
| Bone marrow/ stem cell transplant^^^ | No | 150871 (100%) | 146317 (97%) | 4554 (3%) | 711397 (100%) | 678960 (95.4%) | 32437 (4.6%) |
|  | Yes | 7 (0%) | 6 (85.7%) | 1 (14.3%) | 193 (0%) | 171 (88.6%) | 22 (11.4%) |
| Leukemia and other disorders^$^^ | No | 149762 (99.3%) | 145246 (97%) | 4516 (3%) | 701044 (98.5%) | 669161 (95.5%) | 31883 (4.5%) |
|  | Yes | 1116 (0.7%) | 1077 (96.5%) | 39 (3.5%) | 10546 (1.5%) | 9970 (94.5%) | 576 (5.5%) |
| Cancer CT/ RT^^^ | No | 147571 (97.8%) | 143105 (97%) | 4466 (3%) | 667658 (93.8%) | 636619 (95.4%) | 31039 (4.6%) |
|  | Yes | 3307 (2.2%) | 3218 (97.3%) | 89 (2.7%) | 43932 (6.2%) | 42512 (96.8%) | 1420 (3.2%) |
| Oral corticosteroids^^^ | No | 149154 (98.9%) | 144665 (97%) | 4489 (3%) | 694275 (97.6%) | 662784 (95.5%) | 31491 (4.5%) |
|  | Yes | 1724 (1.1%) | 1658 (96.2%) | 66 (3.8%) | 17315 (2.4%) | 16347 (94.4%) | 968 (5.6%) |
| Other immuno-suppressant drugs^^#^ | No | 150274 (99.6%) | 145745 (97%) | 4529 (3%) | 704364 (99%) | 672362 (95.5%) | 32002 (4.5%) |
|  | Yes | 604 (0.4%) | 578 (95.7%) | 26 (4.3%) | 7226 (1%) | 6769 (93.7%) | 457 (6.3%) |

pyr person-years at risk CI confidence interval ^*^one patients with missing gender information excluded from analysis IQR interquartile range IMD index of multiple deprivation ^~^for excluded group 181 (0.1%) missing values replaced by practice IMD ^+^for included group 668 (0.09%) missing values replaced by practice IMD ^^^ Ever had, ^$^ includes lymphoma, myeloma, other plasma cell dyscrasias CT chemotherapy RT radiotherapy # includes azathioprine, biological therapy, methotrexate, 6-mercaptopurine, other immunosuppressant such as tacrolimus, sirolimus, and other disease-modifying antirheumatic drugs: ciclosporin, mycophenolate, leflunomide
