# Supplementary material for: Inequalities in zoster disease burden: a population‐based cohort study to identify social determinants using linked data from the U.K. Clinical Practice Research Datalink
Source: Br J Dermatol. 2018 Apr 19;178(6):1324–30. doi: 10.1111/bjd.16399 (PMC6033149; doi:10.1111/bjd.16399)
Supplement: Supplementary file 9 — Appendix S7 Comparison of patients excluded due to prior history of zoster and patients included in the study. [file BJD-178-1324-s009.docx]

Appendix S9 Multivariable analysis: Social factors associated with zoster disease incidence amongst patients excluded from analysis due to missing data for ethnicity

(N= 150878, outcome= 4555)^$^

| Exposures | | Minimally adjusted for age, gender and calendar period  RR (95% CI) | P value* | Model additionally adjusted for immigration status & patient-level IMD | P value* | Model additionally adjusted for care home residence & living alone  RR (95% CI) | P value* | Model additionally adjusted for co-morbidities ^  RR (95% CI) | P value* | Model additionally adjusted for immuno-suppressive therapies^+^  RR (95% CI) | P value* |
| --- | --- | --- | --- | --- | --- | --- | --- | --- | --- | --- | --- |
| Age acquired during the study(years) | 65-69 | 1 |  | 1 |  | 1 |  | 1 |  | 1 |  |
|  | 70-74 | 1.10 (1.02-1.19) | <0.0001 | 1.10 (1.02-1.19) | <0.0001 | 1.10 (1.02-1.19) | <0.0001 | 1.09 (1.00-1.18) | <0.0001 | 1.08 (1.00-1.18) | <0.0001 |
|  | 75-79 | 1.33 (1.23-1.45) |  | 1.34 (1.23-1.45) |  | 1.33 (1.23-1.45) |  | 1.29 (1.19-1.41) |  | 1.29 (1.19-1.41) |  |
|  | 80-84 | 1.23 (1.11-1.35) |  | 1.24 (1.12-1.36) |  | 1.23 (1.12-1.36) |  | 1.18 (1.07-1.30) |  | 1.18 (1.07-1.30) |  |
|  | 85 & above | 1.19 (1.08-1.31) |  | 1.20 (1.09-1.33) |  | 1.19 (1.08-1.31) |  | 1.15 (1.04-1.27) |  | 1.15 (1.04-1.27) |  |
| Gender | Male | 0.80 (0.75-0.85) | <0.0001 | 0.80 (0.75-0.85) | <0.0001 | 0.81 (0.76-0.86) | <0.0001 | 0.81 (0.76-0.86) | <0.0001 | 0.81 (0.76-0.86) | <0.0001 |
|  | Female | 1 |  | 1 |  | 1 |  | 1 |  | 1 |  |
| Immigration status | Not immigrant | 1 |  | 1 |  | 1 |  | 1 |  | 1 |  |
|  | Immigrant | 0.52 (0.22-1.26) | 0.11 | 0.54 (0.23-1.31) | 0.13 | 0.54 (0.22-1.30) | 0.13 | 0.53 (0.22-1.29) | 0.12 | 0.54 (0.22-1.29) | 0.12 |
| Patient-level IMD~ | 1 (least deprived) | 1 |  | 1 |  | 1 |  | 1 |  | 1 |  |
|  | 2 | 0.96 (0.89-1.04) | <0.0001 | 0.96 (0.89-1.04) | <0.0001 | 0.97 (0.90-1.04) | <0.0001 | 0.97 (0.90-1.04) | <0.0001 | 0.97 (0.90-1.04) | <0.0001 |
|  | 3 | 0.95 (0.88-1.03) |  | 0.95 (0.88-1.03) |  | 0.96 (0.88-1.04) |  | 0.95 (0.88-1.03) |  | 0.95 (0.88-1.03) |  |
|  | 4 | 0.79 (0.72-0.86) |  | 0.79 (0.72-0.86) |  | 0.80 (0.73-0.88) |  | 0.79 (0.72-0.87) |  | 0.79 (0.72-0.87) |  |
|  | 5 (most deprived) | 0.74 (0.66-0.84) |  | 0.75 (0.66-0.84) |  | 0.76 (0.67-0.86) |  | 0.75 (0.66-0.85) |  | 0.75 (0.66-0.85) |  |
| Practice-level IMD | 1 (least deprived) | 1 |  | Not in model | - | Not in model | - | Not in model | - | Not in model | - |
|  | 2 | 0.91 (0.83-0.99) | <0.0001 |  |  |  |  |  |  |  |  |
|  | 3 | 0.92 (0.85-1.01) |  |  |  |  |  |  |  |  |  |
|  | 4 | 0.81 (0.73-0.89) |  |  |  |  |  |  |  |  |  |
|  | 5 (most deprived) | 0.78 (0.70-0.86) |  |  |  |  |  |  |  |  |  |
| Calendar period | 2003-2005 | 1 |  | 1 |  | 1 |  | 1 |  | 1 |  |
|  | 2006-2007 | 1.01 (0.92-1.10) | 0.63 | 1.00 (0.92-1.09) | 0.53 | 1.00 (0.91-1.08) | 0.49 | 0.98 (0.90-1.07) | 0.19 | 0.99 (0.91-1.07) | 0.22 |
|  | 2008-2009 | 0.95 (0.87-1.04) |  | 0.94 (0.86-1.03) |  | 0.94 (0.86-1.02) |  | 0.92 (0.84-1.00) |  | 0.92 (0.84-1.01) |  |
|  | 2010-2011 | 0.99 (0.91-1.08) |  | 0.98 (0.90-1.07) |  | 0.98 (0.90-1.07) |  | 0.95 (0.87-1.04) |  | 0.96 (0.88-1.05) |  |
|  | 2012-2013 | 0.95 (0.87-1.05) |  | 0.94 (0.86-1.04) |  | 0.94 (0.85-1.03) |  | 0.91 (0.83-1.00) |  | 0.91 (0.83-1.01) |  |
| Care home residence | No | 1 |  | Not in model | - | 1 |  | 1 |  | 1 |  |
|  | Yes | 1.31 (1.11-1.54) | 0.002 |  |  | 1.24 (1.05-1.46) | 0.02 | 1.20 (1.02-1.42) | 0.03 | 1.20 (1.02-1.42) | 0.03 |
| Living alone | No | 1 |  | Not in model | - | 1 |  | 1 |  | 1 |  |
|  | Yes | 0.85 (0.80-0.90) | <0.0001 |  |  | 0.87 (0.82-0.93) | <0.0001 | 0.88 (0.82-0.93) | <0.0001 | 0.88 (0.83-0.94) | <0.0001 |
| Cohabitation | No | 1 |  | Not in model | - | Not in model# | - | Not in model# | - | Not in model# | - |
|  | Yes | 1.18 (1.12-1.25) | <0.0001 |  |  |  |  |  |  |  |  |

^$^one patients with missing gender information excluded from analysis RR rate ratios CI confidence interval IMD index of multiple deprivation ~for excluded group 181 (0.1%) missing values replaced by practice IMD *likelihood ratio test # multicollinearity issue ^included rheumatoid arthritis, systemic lupus erythematosus, inflammatory bowel disease, diabetes mellitus, chronic kidney disease, chronic obstructive pulmonary disease or asthma, HIV infection, other cellular immune deficiency, leukemia, lymphoma, myeloma, other plasma cell dyscrasias, haematopoietic stem cell transplant & solid organ transplant ^+^included immunosuppressive doses of oral/injectable corticosteroids, other immunosuppressants drugs (e.g. azathioprine, biological therapy, methotrexate) and cancer chemo/radiotherapy
