# Supplementary material for: Inequalities in zoster disease burden: a population‐based cohort study to identify social determinants using linked data from the U.K. Clinical Practice Research Datalink
Source: Br J Dermatol. 2018 Apr 19;178(6):1324–30. doi: 10.1111/bjd.16399 (PMC6033149; doi:10.1111/bjd.16399)
Supplement: Supplementary file 10 — Appendix S8 Baseline characteristics of patients excluded from analysis due to missing data for ethnicity and included in complete case analysis. [file BJD-178-1324-s010.docx]

Appendix S10 Multivariable analysis: Social factors associated with zoster disease incidence restricted to patients of White ethnicity

(N= 684869 outcome= 31789)

| Exposures | | Minimally adjusted for age, gender and calendar period  RR (95% CI) | P value* (PT) | Model adjusted for age, gender, calendar period, immigration status and patient-level IMD | P value*  (PT) | Model additionally adjusted for care home residence & living alone  RR (95% CI) | P value*  (PT) | Model additionally adjusted for co-morbidites#  RR (95% CI) | P value*  (PT) | Model 4 additionally adjusted for immuno-suppressive therapies~  RR (95% CI) | P value*  (PT) |
| --- | --- | --- | --- | --- | --- | --- | --- | --- | --- | --- | --- |
| Age acquired during the study(years) | 65-69 | 1 |  | 1 |  | 1 |  | 1 |  | 1 |  |
|  | 70-74 | 1.16 (1.12-1.20) | <0.0001 | 1.16 (1.12-1.20) | <0.0001 | 1.16 (1.12-1.20) | <0.0001 | 1.15 (1.11-1.18) | <0.0001 | 1.15 (1.11-1.18) | <0.0001 |
|  | 75-79 | 1.29 (1.25-1.34) | (<0.0001) | 1.29 (1.25-1.34) | (<0.0001) | 1.29 (1.25-1.34) | (<0.0001) | 1.27 (1.23-1.31) | (<0.0001) | 1.27 (1.23-1.31) | (<0.0001) |
|  | 80-84 | 1.35 (1.30-1.40) |  | 1.35 (1.30-1.40) |  | 1.35 (1.30-1.40) |  | 1.32 (1.27-1.36) |  | 1.32 (1.27-1.37) |  |
|  | 85 & above | 1.38 (1.34-1.44) |  | 1.39 (1.34-1.44) |  | 1.38 (1.33-1.43) |  | 1.35 (1.30-1.40) |  | 1.36 (1.31-1.41) |  |
| Gender | Male | 0.85 (0.83-0.87) | <0.0001 | 0.85 (0.83-0.87) | <0.0001 | 0.85 (0.83-0.87) | <0.0001 | 0.85 (0.84-0.87) | <0.0001 | 0.85 (0.83-0.87) | <0.0001 |
|  | Female | 1 |  | 1 |  | 1 |  | 1 |  | 1 |  |
| Immigration status | Not immigrant | 1 |  | 1 |  | 1 |  | 1 |  | 1 |  |
|  | Immigrant | 0.74 (0.61-0.90) | 0.002 | 0.75 (0.62-0.91) | 0.002 | 0.75 (0.62-0.90) | 0.002 | 0.75 (0.62-0.91) | 0.002 | 0.75 (0.62-0.91) | 0.002 |
| Patient level IMD~ | 1 (least deprived) | 1 |  | 1 |  | 1 |  | 1 |  | 1 |  |
|  | 2 | 0.98 (0.95-1.01) | 0.02 | 0.98 (0.95-1.01) | 0.02 | 0.98 (0.95-1.01) | 0.04 | 0.98 (0.95-1.01) | 0.0006 | 0.98 (0.95-1.01) | 0.0009 |
|  | 3 | 0.96 (0.92-0.99) |  | 0.96 (0.92-0.99) |  | 0.96 (0.93-0.99) |  | 0.95 (0.92-0.98) |  | 0.95 (0.92-0.98) |  |
|  | 4 | 0.95 (0.92-0.99) |  | 0.95 (0.92-0.99) |  | 0.96 (0.92-0.99) |  | 0.94 (0.91-0.98) |  | 0.94 (0.91-0.98) |  |
|  | 5 (most deprived) | 0.95 (0.92-0.99) |  | 0.96 (0.92-0.99) |  | 0.96 (0.92-1.00) |  | 0.93 (0.90-0.97) |  | 0.93 (0.90-0.97) |  |
| Practice-level IMD | 1 (least deprived) | 1 |  | Not in model | - | Not in model | - | Not in model | - | Not in model | - |
|  | 2 | 0.92 (0.88-0.95) | <0.0001 |  |  |  |  |  |  |  |  |
|  | 3 | 0.97 (0.93-1.00) |  |  |  |  |  |  |  |  |  |
|  | 4 | 0.92 (0.89-0.95) |  |  |  |  |  |  |  |  |  |
|  | 5 (most deprived) | 0.90 (0.87-0.94) |  |  |  |  |  |  |  |  |  |
| Calendar period | 2003-2005 | 1 |  | 1 |  | 1 |  | 1 |  | 1 |  |
|  | 2006-2007 | 1.03 (1.00-1.07) | 0.13 | 1.03 (1.00-1.07) | 0.13 | 1.03 (1.00-1.06) | 0.13 | 1.01 (0.98-1.05) | 0.005 | 1.01 (0.98-1.05) | 0.0008 |
|  | 2008-2009 | 1.03 (1.00-1.06) |  | 1.03 (1.00-1.06) |  | 1.03 (0.99-1.06) |  | 1.00 (0.96-1.03) |  | 0.99 (0.96-1.03) |  |
|  | 2010-2011 | 1.02 (0.98-1.05) |  | 1.02 (0.98-1.05) |  | 1.01 (0.98-1.05) |  | 0.98 (0.94-1.01) |  | 0.97 (0.94-1.01) |  |
|  | 2012-2013 | 0.99 (0.96-1.03) |  | 0.99 (0.96-1.03) |  | 0.99 (0.95-1.02) |  | 0.95 (0.91-0.98) |  | 0.94 (0.91-0.98) |  |
| Care home residence | No | 1 |  | Not in model | - | 1 |  | 1 |  | 1 |  |
|  | Yes | 1.11 (1.05-1.18) | 0.0002 |  |  | 1.10 (1.04-1.16) | 0.001 | 1.08 (1.02-1.15) | 0.007 | 1.08 (1.02-1.15) | 0.006 |
| Living alone | No | 1 |  | Not in model | - | 1 |  | 1 |  | 1 |  |
|  | Yes | 0.95 (0.93-0.98) | 0.0001 |  |  | 0.96 (0.94-0.98) | 0.001 | 0.96 (0.94-0.99) | 0.002 | 0.96 (0.94-0.99) | 0.002 |
| Cohabitation | No | 1 |  | Not in model | - | Not in model # |  | Not in model # |  | Not in model # |  |
|  | Yes | 1.06 (1.03-1.08) | <0.0001 |  |  |  |  |  |  |  |  |

RR rate ratios CI confidence interval PT P value for trend IMD index of multiple deprivation ~ 648 patients (0.09%) missing values replaced by practice IMD *likelihood ratio test # multicollinearity issue ^included rheumatoid arthritis, systemic lupus erythematosus, inflammatory bowel disease, diabetes mellitus, chronic kidney disease, chronic obstructive pulmonary disease or asthma, HIV infection, other cellular immune deficiency, leukemia, lymphoma, myeloma, other plasma cell dyscrasias, haematopoietic stem cell transplant & solid organ transplant ~included immunosuppressive doses of oral/injectable corticosteroids, other immunosuppressants drugs (e.g. azathioprine, biological therapy, methotrexate) and cancer chemo/radiotherapy
