# Supplementary material for: Inequalities in zoster disease burden: a population‐based cohort study to identify social determinants using linked data from the U.K. Clinical Practice Research Datalink
Source: Br J Dermatol. 2018 Apr 19;178(6):1324–30. doi: 10.1111/bjd.16399 (PMC6033149; doi:10.1111/bjd.16399)
Supplement: Supplementary file 11 — Appendix S9 Multivariable analysis: social factors associated with zoster disease incidence among patients excluded from analysis due to missing data for ethnicity. [file BJD-178-1324-s011.docx]

Appendix S11 Sensitivity analysis: Multivariable analysis including practice-level IMD

| Exposures | | Model adjusted for age, gender, calendar period, immigration status, ethnicity and practice-level IMD | P value* | Model 2 additionally adjusted for care home residence & living alone  RR (95% CI) | P value* | Model 3 additionally adjusted for co-morbidities#  RR (95% CI) | P value* | Model 4 additionally adjusted for immuno-suppressive therapies~  RR (95% CI) | P value* |
| --- | --- | --- | --- | --- | --- | --- | --- | --- | --- |
| Age acquired during the study(years) | 65-69 | 1 |  | 1 |  | 1 |  | 1 |  |
|  | 70-74 | 1.16 (1.12-1.20) | <0.0001 | 1.16 (1.12-1.20) | <0.0001 | 1.15 (1.11-1.18) | <0.0001 | 1.15 (1.11-1.18) | <0.0001 |
|  | 75-79 | 1.29 (1.25-1.33) |  | 1.29 (1.25-1.33) |  | 1.26 (1.22-1.31) |  | 1.26 (1.22-1.31) |  |
|  | 80-84 | 1.35 (1.30-1.40) |  | 1.35 (1.30-1.39) |  | 1.32 (1.27-1.36) |  | 1.32 (1.27-1.37) |  |
|  | 85 & above | 1.38 (1.33-1.43) |  | 1.37 (1.32-1.42) |  | 1.35 (1.30-1.40) |  | 1.36 (1.31-1.41) |  |
| Gender | Male | 0.85 (0.84-0.87) | <0.0001 | 0.86 (0.84-0.87) | <0.0001 | 0.86 (0.84-0.88) | <0.0001 | 0.86 (0.84-0.88) | <0.0001 |
|  | Female | 1 |  | 1 |  | 1 |  | 1 |  |
| Ethnicity | White | 1 |  | 1 |  | 1 |  | 1 |  |
|  | South Asian | 0.76 (0.68-0.85) | <0.0001 | 0.76 (0.67-0.85) | <0.0001 | 0.75 (0.67-0.84) | <0.0001 | 0.75 (0.67-0.84) | <0.0001 |
|  | Black | 0.49 (0.41-0.59) |  | 0.49 (0.41-0.59) |  | 0.49 (0.41-0.59) |  | 0.49 (0.42-0.59) |  |
|  | Other | 0.85 (0.74-0.99) |  | 0.85 (0.73-0.99) |  | 0.86 (0.74-1.00) |  | 0.86 (0.74-1.00) |  |
|  | Mixed | 0.78 (0.58-1.06) |  | 0.78 (0.58-1.06) |  | 0.78 (0.58-1.06) |  | 0.78 (0.58-1.06) |  |
| Immigration status | Not immigrant | 1 |  | 1 |  | 1 |  | 1 |  |
|  | Immigrant | 0.77 (0.67-0.89) | 0.0002 | 0.77 (0.67-0.89) | 0.0002 | 0.77 (0.67-0.89) | 0.0002 | 0.77 (0.67-0.89) | 0.0002 |
| Patient level IMD~ | 1 (least deprived) | Not in model |  | Not in model | - | Not in model | - | Not in model | - |
|  | 2 |  |  |  |  |  |  |  |  |
|  | 3 |  |  |  |  |  |  |  |  |
|  | 4 |  |  |  |  |  |  |  |  |
|  | 5 (most deprived) |  |  |  |  |  |  |  |  |
| Practice-level IMD | 1 (least deprived) | 1 |  | 1 |  | 1 |  | 1 |  |
|  | 2 | 0.92 (0.88-0.95) | <0.0001 | 0.92 (0.89-0.95) | <0.0001 | 0.92 (0.89-0.95) | <0.0001 | 0.92 (0.89-0.95) | <0.0001 |
|  | 3 | 0.97 (0.93-1.00) |  | 0.97 (0.93-1.00) |  | 0.97 (0.93-1.00) |  | 0.97 (0.93-1.00) |  |
|  | 4 | 0.92 (0.88-0.95) |  | 0.92 (0.89-0.95) |  | 0.91 (0.88-0.95) |  | 0.92 (0.88-0.95) |  |
|  | 5 (most deprived) | 0.90 (0.87-0.94) |  | 0.91 (0.87-0.94) |  | 0.90 (0.86-0.93) |  | 0.90 (0.86-0.93) |  |
| Calendar period | 2003-2005 | 1 |  | 1 |  | 1 |  | 1 |  |
|  | 2006-2007 | 1.03 (0.99-1.06) | 0.18 | 1.03 (0.99-1.06) | 0.17 | 1.01 (0.98-1.04) | 0.007 | 1.01 (0.98-1.04) | 0.0013 |
|  | 2008-2009 | 1.03 (0.99-1.06) |  | 1.02 (0.99-1.06) |  | 0.99 (0.96-1.03) |  | 0.99 (0.96-1.03) |  |
|  | 2010-2011 | 1.02 (0.98-1.05) |  | 1.01 (0.98-1.05) |  | 0.98 (0.94-1.01) |  | 0.97 (0.94-1.01) |  |
|  | 2012-2013 | 0.99 (0.96-1.03) |  | 0.99 (0.95-1.02) |  | 0.95 (0.91-0.98) |  | 0.94 (0.91-0.97) |  |
| Care home residence | No | Not in model | - | 1 |  | 1 |  | 1 |  |
|  | Yes |  |  | 1.10 (1.04-1.16) | 0.0008 | 1.08 (1.02-1.15) | 0.005 | 1.08 (1.03-1.15) | 0.005 |
| Living alone | No | Not in model | - | 1 |  | 1 |  | 1 |  |
|  | Yes |  |  | 0.96 (0.94-0.98) | 0.0007 | 0.96 (0.94-0.98) | 0.001 | 0.96 (0.94-0.99) | 0.001 |
| Cohabitation | No | Not in model | - | Not in model # |  | Not in model # |  | Not in model # |  |
|  | Yes |  |  |  |  |  |  |  |  |

RR rate ratios CI confidence interval IMD index of multiple deprivation ~ 668 patients (0.09%) missing values replaced by practice IMD *likelihood ratio test # multicollinearity issue ^included rheumatoid arthritis, systemic lupus erythematosus, inflammatory bowel disease, diabetes mellitus, chronic kidney disease, chronic obstructive pulmonary disease or asthma, HIV infection, other cellular immune deficiency, leukemia, lymphoma, myeloma, other plasma cell dyscrasias, haematopoietic stem cell transplant & solid organ transplant ~included immunosuppressive doses of oral/injectable corticosteroids, other immunosuppressant drugs (e.g. azathioprine, biological therapy, methotrexate) and cancer chemo/radiotherapy
