# Supplementary material for: Inequalities in zoster disease burden: a population‐based cohort study to identify social determinants using linked data from the U.K. Clinical Practice Research Datalink
Source: Br J Dermatol. 2018 Apr 19;178(6):1324–30. doi: 10.1111/bjd.16399 (PMC6033149; doi:10.1111/bjd.16399)
Supplement: Supplementary file 13 — Appendix S11 Sensitivity analysis: multivariable analysis including practice‐level Index of Multiple Deprivation. [file BJD-178-1324-s013.docx]

Table S1 Baseline characteristics of the study cohort (*N* = 862 470 outcome *n* = 37 014).

| Characteristics | | Total (column %*) | Outcome n (row %*) | Person-years (1000) | Zoster incidence per 1,000 person-years at risk (95% CI) |
| --- | --- | --- | --- | --- | --- |
| Overall | | 862,470 | 37,014 (4.3%) | 4210.5 | 8.79 (8.70–8.88) |
| *Time-constant exposure variables* | |  |  |  |  |
| Age at start of follow-up (years) | 65-69 | 442499 (51.3%) | 15979 (3.6%) | 2051 | 7.79 (7.67-7.91) |
|  | 70-74 | 136151 (15.8%) | 7842 (5.8%) | 856.6 | 9.15 (8.95-9.36) |
|  | 75-79 | 112302 (13%) | 6358 (5.7%) | 632.9 | 10.05 (9.80-10.30) |
|  | 80-84 | 89827 (10.4%) | 4292 (4.8%) | 420.7 | 10.20 (9.90-10.51) |
|  | 85 & above | 81691 (9.5%) | 2543 (3.1%) | 249.3 | 10.20 (9.81-10.60) |
| Sex | Male | 389,264 (45.1%) | 14771 (3.8%) | 1873.7 | 7.88 (7.76-8.01) |
|  | Female | 473,204 (54.9%) | 22243 (4.7%) | 2336.8 | 9.52 (9.39-9.64) |
|  | Missing | 2 (0.0002%) | 0 | - | - |
| Ethnicity | White | 684870 (79.4%) | 31789 (4.6%) | 3454.5 | 9.20 (9.10-9.30) |
|  | South Asian | 12273 (1.4%) | 322 (2.6%) | 52.1 | 6.18 (5.54-6.90) |
|  | Black | 7176 (0.8%) | 128 (1.8%) | 30.5 | 4.19 (3.53-4.99) |
|  | Other | 5850 (0.7%) | 178 (3%) | 24.1 | 7.39 (6.38-8.56) |
|  | Mixed | 1422 (0.2%) | 42 (3%) | 6.2 | 6.75 (4.99-9.14) |
|  | Missing | 150879 (17.5%) | 4555 (3%) | - | - |
| Religion | Christian | 14705 (1.7%) | 660 (4.5%) | 68.7 | 9.60 (8.90-10.36) |
|  | Buddhist | 47 (0%) | 1 (2.1%) | 0.2 | 5.96 (0.84-42.30) |
|  | Hindu | 428 (0.1%) | 16 (3.7%) | 2.1 | 7.69 (4.71-12.55) |
|  | Jewish | 344 (0%) | 8 (2.3%) | 1.7 | 4.79 (2.40-9.59) |
|  | Muslim | 681 (0.1%) | 9 (1.3%) | 2.9 | 3.16 (1.64-6.07) |
|  | Sikh | 136 (0%) | 2 (1.5%) | 0.5 | 4.06 (1.01-16.23) |
|  | Other | 27 (0%) | 0 (0%) | 0.1 | - |
|  | No religion | 1085 (0.1%) | 51 (4.7%) | 4.4 | 11.59 (8.81-15.24) |
|  | Missing | 845017 (98%) | 36267 (4.3%) | - | - |
| Immigration status | Not immigrant | 853123 (98.9%) | 36785 (4.3%) | 4170.6 | 8.82 (8.73-8.91) |
|  | Immigrant | 9347 (1.1%) | 229 (2.4%) | 39.9 | 5.74 (5.04-6.53) |
| Patient-level IMD~ | 1 (least deprived) | 201684 (23.4%) | 9141 (4.5%) | 1011.5 | 9.04 (8.85-9.22) |
|  | 2 | 220924 (25.6%) | 9790 (4.4%) | 1095.6 | 8.94 (8.76-9.11) |
|  | 3 | 181648 (21.1%) | 7671 (4.2%) | 881 | 8.71 (8.51-8.90) |
|  | 4 | 158865 (18.4%) | 6485 (4.1%) | 758.8 | 8.55 (8.34-8.76) |
|  | 5 (most deprived) | 99349 (11.5%) | 3927 (4%) | 463.7 | 8.47 (8.21-8.74) |
| Practice-level IMD | 1 (least deprived) | 129174 (15%) | 6101 (4.7%) | 649.7 | 9.39 (9.16-9.63) |
|  | 2 | 197749 (22.9%) | 8420 (4.3%) | 972 | 8.66 (8.48-8.85) |
|  | 3 | 195727 (22.7%) | 8491 (4.3%) | 935.5 | 9.08 (8.89-9.27) |
|  | 4 | 185751 (21.5%) | 7649 (4.1%) | 896.2 | 8.53 (8.35-8.73) |
|  | 5 (most deprived) | 154069 (17.9%) | 6353 (4.1%) | 757.2 | 8.39 (8.19-8.60) |
| *Time-varying exposure variables* | |  |  |  |  |
| Age acquired during the study (years) | 65-69 | - | 8907 | 1227.4 | 7.26 (7.11-7.41) |
|  | 70-74 | - | 8488 | 1006.6 | 8.43 (8.25-8.61) |
|  | 75-79 | - | 7934 | 827.5 | 9.59 (9.38-9.80) |
|  | 80-84 | - | 6147 | 613.5 | 10.02 (9.77-10.27) |
|  | 85 & above | - | 5538 | 535.5 | 10.34 (10.07-10.62) |
| Living alone | No | - | 24865 | 2767.5 | 8.98 (8.87-9.1) |
|  | Yes | - | 12149 | 1443.1 | 8.42 (8.27-8.57) |
| Marital status | Single | - | 402 | 46.2 | 8.69 (7.88-9.59) |
|  | Married/Civil | - | 7126 | 768.4 | 9.27 (9.06-9.49) |
|  | Widow/er | - | 2730 | 265.1 | 10.30 (9.92-10.69) |
|  | Divorced | - | 332 | 39.5 | 8.41 (7.55-9.37) |
|  | Separated | - | 111 | 11.2 | 9.87 (8.20-11.89) |
|  | Partner uncategorized/ other | - | 10471 | 1169.1 | 8.96 (8.79-9.13) |
|  | Missing | 407609 (47.3%) | 15842 (3.9%) | - | - |
| Cohabiting | No | - | 18855 | 2215.9 | 8.51 (8.39-8.63) |
|  | Yes | - | 18159 | 1994.6 | 9.1 (8.97-9.24) |
| Care home | No | - | 35494 | 4071.7 | 8.72 (8.63-8.81) |
|  | Yes | - | 1520 | 138.8 | 10.95 (10.41-11.51) |
| Calendar period | 2003-2005 | - | 8357 | 958.9 | 8.72 (8.53-8.90) |
|  | 2006-2007 | - | 7611 | 849 | 8.96 (8.77-9.17) |
|  | 2008-2009 | - | 7752 | 872.6 | 8.88 (8.69-9.08) |
|  | 2010-2011 | - | 7528 | 853.2 | 8.82 (8.63-9.02) |
|  | 2012-2013 | - | 5766 | 676.9 | 8.52 (8.30-8.74) |
| Comorbidities and medications | |  |  |  |  |
| Rheumatoid arthritis | No | - | 35724 | 4114.6 | 8.68 (8.59-8.77) |
|  | Yes | - | 1290 | 95.9 | 13.45 (12.74-14.2) |
| Systemic lupus Erythematosus | No | - | 36923 | 4204.5 | 8.78 (8.69-8.87) |
|  | Yes | - | 91 | 6.1 | 15.05 (12.26-18.49) |
| Inflammatory bowel disease | No | - | 36417 | 4158.6 | 8.76 (8.67-8.85) |
|  | Yes | - | 597 | 52 | 11.49 (10.6-12.45) |
| Diabetes mellitus | No | - | 31335 | 3584.9 | 8.74 (8.64-8.84) |
|  | Yes | - | 5679 | 625.7 | 9.08 (8.84-9.32) |
| Chronic kidney disease | No | - | 30563 | 3590.2 | 8.51 (8.42-8.61) |
|  | Yes | - | 6451 | 620.3 | 10.4 (10.15-10.66) |
| COPD/ asthma | No | - | 29365 | 3504.1 | 8.38 (8.28-8.48) |
|  | Yes | - | 7649 | 706.4 | 10.83 (10.59-11.07) |
| Human Immunodeficiency virus infection | No | - | 37008 | 4210 | 8.79 (8.7-8.88) |
|  | Yes | - | 6 | 0.5 | 12.26 (5.51-27.29) |
| Cellular immune deficiency | No | - | 36932 | 4204.2 | 8.78 (8.7-8.87) |
|  | Yes | - | 82 | 6.3 | 12.97 (10.44-16.1) |
| Solid organ transplant | No | - | 36949 | 4206.4 | 8.78 (8.69-8.87) |
|  | Yes | - | 65 | 4.2 | 15.66 (12.28-19.97) |
| Bone marrow/ stem cell transplant | No | - | 36997 | 4210.3 | 8.79 (8.7-8.88) |
|  | Yes | - | 17 | 0.2 | 79.01 (49.12-127.1) |
| Lymphoma, myeloma, other plasma cell dyscrasias and leukaemia | No | - | 36535 | 4191.4 | 8.72 (8.63-8.81) |
|  | Yes | - | 479 | 19.1 | 25.03 (22.88-27.37) |
| Cancer chemotherapeutic agents/ cancer radiotherapy | No | - | 36256 | 4164.3 | 8.71 (8.62-8.8) |
|  | Yes | - | 758 | 46.2 | 16.41 (15.28-17.62) |
| Oral corticosteroids | No | - | 36655 | 4192.6 | 8.74 (8.65-8.83) |
|  | Yes | - | 359 | 17.9 | 20.02 (18.05-22.2) |
| Other immunosuppressant drugs^#^ | No | - | 36673 | 4191.8 | 8.75 (8.66-8.84) |
|  | Yes | - | 341 | 18.7 | 18.2 (16.36-20.23) |

*percentage presented for time-constant variables ~849 (0.1%) missing values for patient-level IMD replaced by practice IMD; ^#^includes azathioprine, biological therapy, methotrexate, 6-mercaptopurine, other immunosuppressants such as tacrolimus, sirolimus, and other disease-modifying antirheumatic drugs: ciclosporin, mycophenolate, leflunomide.
