# Supplementary material for: Inequalities in zoster disease burden: a population‐based cohort study to identify social determinants using linked data from the U.K. Clinical Practice Research Datalink
Source: Br J Dermatol. 2018 Apr 19;178(6):1324–30. doi: 10.1111/bjd.16399 (PMC6033149; doi:10.1111/bjd.16399)
Supplement: Supplementary file 14 — Appendix S12. Sensitivity analysis: multivariable analysis including cohabitation. [file BJD-178-1324-s014.docx]

Table S2 Multivariable analysis: social factors associated with zoster disease incidence (complete case analysis; individuals with missing data for ethnicity and sex excluded) (*N* = 711 590, outcome *n* =32 459)

| Exposures | | Minimally adjusted for age, sex and calendar period  RR (95% CI) | P-value* (PT) | Model 1: additionally adjusted for immigration status & ethnicity RR (95% CI) | P-value*  (PT) | Model 2: additionally adjusted for patient-level IMD RR (95% CI) | P-value*  (PT) | Model 3: additionally adjusted for care home residence & living alone  RR (95% CI) | P-value*  (PT) | Model 4: additionally adjusted for comorbidities  RR (95% CI) | P-value*  (PT) | Model 5: additionally adjusted for immuno-suppressive therapies  RR (95% CI) | P-value*  (PT) |
| --- | --- | --- | --- | --- | --- | --- | --- | --- | --- | --- | --- | --- | --- |
| Age acquired during the study(years) | 65-69 | 1 |  | 1 |  | 1 |  | 1 |  | 1 |  | 1 | <0.0001 |
|  | 70-74 | 1.16 (1.12-1.20) | <0.0001 | 1.16 (1.12-1.20) | <0.0001 | 1.16 (1.12-1.20) | <0.0001 | 1.16 (1.12-1.20) | <0.0001 | 1.15 (1.11-1.18) | <0.0001 | 1.15 (1.11-1.18) |  |
|  | 75-79 | 1.29 (1.25-1.34) | (<0.0001) | 1.29 (1.25-1.33) | (<0.0001) | 1.29 (1.25-1.33) | (<0.0001) | 1.29 (1.25-1.33) | (<0.0001) | 1.26 (1.22-1.31) | (<0.0001) | 1.27 (1.22-1.31) | (<0.0001) |
|  | 80-84 | 1.36 (1.31-1.41) |  | 1.35 (1.31-1.40) |  | 1.35 (1.31-1.40) |  | 1.35 (1.30-1.40) |  | 1.32 (1.27-1.36) |  | 1.32 (1.28-1.37) |  |
|  | 85 & above | 1.40 (1.35-1.45) |  | 1.38 (1.34-1.44) |  | 1.39 (1.34-1.44) |  | 1.38 (1.33-1.43) |  | 1.35 (1.30-1.40) |  | 1.36 (1.31-1.41) |  |
| Sex | Male | 0.85 (0.84-0.87) | <0.0001 | 0.85 (0.84-0.87) | <0.0001 | 0.85 (0.84-0.87) | <0.0001 | 0.86 (0.84-0.87) | <0.0001 | 0.86 (0.84-0.88) | <0.0001 | 0.86 (0.84-0.88) | <0.0001 |
|  | Female | 1 |  | 1 |  | 1 |  | 1 |  | 1 |  | 1 |  |
| Ethnicity | White | 1 |  | 1 |  | 1 |  | 1 |  | 1 |  | 1 |  |
|  | South Asian | 0.70 (0.63-0.79) | <0.0001 | 0.76 (0.67-0.85) | <0.0001 | 0.76 (0.68-0.85) | <0.0001 | 0.76 (0.68-0.85) | <0.0001 | 0.75 (0.67-0.84) | <0.0001 | 0.75 (0.67-0.84) | <0.0001 |
|  | Black | 0.47 (0.40-0.56) |  | 0.49 (0.41-0.58) |  | 0.49 (0.41-0.59) |  | 0.49 (0.41-0.59) |  | 0.49 (0.42-0.59) |  | 0.50 (0.42-0.59) |  |
|  | Other | 0.83 (0.71-0.96) |  | 0.85 (0.74-0.99) |  | 0.86 (0.74-0.99) |  | 0.85 (0.74-0.99) |  | 0.86 (0.75-1.00) |  | 0.87 (0.75-1.00) |  |
|  | Mixed | 0.77 (0.57-1.04) |  | 0.78 (0.58-1.06) |  | 0.78 (0.58-1.06) |  | 0.78 (0.58-1.06) |  | 0.78 (0.58-1.06) |  | 0.79 (0.58-1.06) |  |
| Immigration status | Not immigrant | 1 |  | 1 |  | 1 |  | 1 |  | 1 |  | 1 |  |
|  | Immigrant | 0.65 (0.57-0.74) | <0.0001 | 0.77 (0.67-0.88) | 0.0001 | 0.77 (0.67-0.88) | 0.0001 | 0.77 (0.67-0.88) | 0.0001 | 0.77 (0.67-0.89) | 0.0002 | 0.77 (0.67-0.89) | 0.0002 |
| Patient-level IMD~ | 1 (least deprived) | 1 |  | Not in model | - | 1 |  | 1 |  | 1 |  | 1 |  |
|  | 2 | 0.98 (0.95-1.01) | 0.0006 |  |  | 0.98 (0.95-1.01) | 0.01 | 0.98 (0.95-1.01) | 0.02 | 0.98 (0.95-1.01) | 0.0003 | 0.98 (0.95-1.01) | 0.0005 |
|  | 3 | 0.95 (0.92-0.98) |  |  |  | 0.95 (0.92-0.99) |  | 0.95 (0.92-0.99) |  | 0.95 (0.92-0.98) |  | 0.95 (0.92-0.98) |  |
|  | 4 | 0.94 (0.91-0.98) |  |  |  | 0.95 (0.92-0.99) |  | 0.96 (0.92-0.99) |  | 0.94 (0.91-0.98) |  | 0.94 (0.91-0.98) |  |
|  | 5 (most deprived) | 0.93 (0.90-0.97) |  |  |  | 0.95 (0.92-0.99) |  | 0.96 (0.92-0.99) |  | 0.93 (0.89-0.97) |  | 0.93 (0.89-0.97) |  |
| Practice-level IMD | 1 (least deprived) | 1 |  | Not in model | - | Not in model | - | Not in model | - | Not in model | - | Not in model | - |
|  | 2 | 0.92 (0.88-0.95) | <0.0001 |  |  |  |  |  |  |  |  |  |  |
|  | 3 | 0.96 (0.93-1.00) |  |  |  |  |  |  |  |  |  |  |  |
|  | 4 | 0.91 (0.88-0.94) |  |  |  |  |  |  |  |  |  |  |  |
|  | 5 (most deprived) | 0.90 (0.86-0.93) |  |  |  |  |  |  |  |  |  |  |  |
| Calendar period | 2003-2005 | 1 |  | 1 |  | 1 |  | 1 |  | 1 |  | 1 |  |
|  | 2006-2007 | 1.03 (0.99-1.06) | 0.14 | 1.03 (0.99-1.06) | 0.18 | 1.03 (0.99-1.06) | 0.19 | 1.03 (0.99-1.06) | 0.18 | 1.01 (0.98-1.04) | 0.007 | 1.01 (0.98-1.04) | 0.001 |
|  | 2008-2009 | 1.02 (0.99-1.06) |  | 1.03 (0.99-1.06) |  | 1.03 (0.99-1.06) |  | 1.02 (0.99-1.06) |  | 0.99 (0.96-1.03) |  | 0.99 (0.96-1.03) |  |
|  | 2010-2011 | 1.01 (0.98-1.05) |  | 1.02 (0.99-1.05) |  | 1.02 (0.98-1.05) |  | 1.01 (0.98-1.05) |  | 0.98 (0.94-1.01) |  | 0.97 (0.94-1.01) |  |
|  | 2012-2013 | 0.99 (0.95-1.02) |  | 0.99 (0.96-1.03) |  | 0.99 (0.96-1.03) |  | 0.99 (0.95-1.02) |  | 0.95 (0.91-0.98) |  | 0.94 (0.91-0.97) |  |
| Care home residence | No | 1 |  | Not in model | - | Not in model | - | 1 |  | 1 |  | 1 |  |
|  | Yes | 1.12 (1.06-1.18) | 0.0001 |  |  |  |  | 1.10 (1.04-1.17) | 0.0007 | 1.09 (1.03-1.15) | 0.004 | 1.09 (1.03-1.15) | 0.004 |
| Living alone | No | 1 |  | Not in model | - | Not in model | - | 1 |  | 1 |  | 1 |  |
|  | Yes | 0.95 (0.93-0.97) | <0.0001 |  |  |  |  | 0.96 (0.94-0.98) | 0.0006 | 0.96 (0.94-0.98) | 0.001 | 0.96 (0.94-0.99) | 0.002 |
| Cohabitation | No | 1 |  | Not in model | - | Not in model | - | Not in model | - | Not in model | - | Not in model | - |
|  | Yes | 1.07 (1.05-1.09) | <0.0001 |  |  |  |  |  |  |  |  |  |  |
| Rheumatoid arthritis | No | 1 |  | Not in model | - | Not in model | - | Not in model | - | 1 |  | 1 |  |
|  | Yes | 1.45 (1.37-1.54) | <0.0001 |  |  |  |  |  |  | 1.40 (1.32-1.49) | <0.0001 | 1.34 (1.26-1.42) | <0.0001 |
| Systemic lupus Erythematosus | No | 1 |  | Not in model | - | Not in model | - | Not in model | - | 1 |  | 1 |  |
|  | Yes | 1.67 (1.35-2.07) | <0.0001 |  |  |  |  |  |  | 1.53 (1.24-1.89) | 0.0003 | 1.45 (1.17-1.80) | 0.001 |
| Inflammatory bowel disease | No | 1 |  | Not in model | - | Not in model | - | Not in model | - | 1 |  | 1 |  |
|  | Yes | 1.29 (1.18-1.40) | <0.0001 |  |  |  |  |  |  | 1.25 (1.15-1.36) | <0.0001 | 1.19 (1.10-1.30) | 0.0001 |
| Diabetes mellitus | No | 1 |  | Not in model | - | Not in model | - | Not in model | - | 1 |  | 1 |  |
|  | Yes | 1.02 (0.99-1.05) | 0.21 |  |  |  |  |  |  | 1.01 (0.98-1.04) | 0.48 | 1.01 (0.98-1.04) | 0.52 |
| Chronic kidney disease | No | 1 |  | Not in model | - | Not in model | - | Not in model | - | 1 |  | 1 |  |
|  | Yes | 1.13 (1.10-1.17) | <0.0001 |  |  |  |  |  |  | 1.11 (1.08-1.15) | <0.0001 | 1.11 (1.07-1.14) | <0.0001 |
| COPD/ asthma | No | 1 |  | Not in model | - | Not in model | - | Not in model | - | 1 |  | 1 |  |
|  | Yes | 1.26 (1.23-1.30) | <0.0001 |  |  |  |  |  |  | 1.25 (1.22-1.29) | <0.0001 | 1.24 (1.21-1.27) | <0.0001 |
| HIV | No | 1 |  | Not in model | - | Not in model | - | Not in model | - | 1 |  | 1 |  |
|  | Yes | 1.80 (0.81-4.01) | 0.19 |  |  |  |  |  |  | 1.93 (0.87-4.29) | 0.15 | 1.94 (0.87-4.31) | 0.14 |
| Cellular immune deficiency | No | 1 |  | Not in model | - | Not in model | - | Not in model | - | 1 |  | 1 |  |
|  | Yes | 1.41 (1.13-1.77) | 0.004 |  |  |  |  |  |  | 1.15 (0.91-1.43) | 0.25 | 1.04 (0.83-1.31) | 0.71 |
| Solid organ transplant | No | 1 |  | Not in model | - | Not in model | - | Not in model | - | 1 |  | 1 |  |
|  | Yes | 1.95 (1.53-2.48) | <0.0001 |  |  |  |  |  |  | 1.81 (1.42-2.31) | <0.0001 | 1.43 (1.12-1.84) | 0.008 |
| Bone marrow/ stem cell transplant | No | 1 |  | Not in model | - | Not in model | - | Not in model | - | 1 |  | 1 |  |
|  | Yes | 10.31 (6.41-16.59) | <0.0001 |  |  |  |  |  |  | 4.53 (2.80-7.35) | <0.0001 | 3.84 (2.37-6.24) | <0.0001 |
| Lymphoma, myeloma, other plasma cell dyscrasias & leukaemia | No | 1 |  | Not in model | - | Not in model | - | Not in model | - | 1 |  | 1 |  |
|  | Yes | 2.83 (2.58-3.10) | <0.0001 |  |  |  |  |  |  | 2.68 (2.44-2.94) | <0.0001 | 2.36 (2.14-2.60) | <0.0001 |
| Cancer chemotherapeutic agents/ cancer radiotherapy | No | 1 |  | Not in model | - | Not in model | - | Not in model | - | Not in model | - | 1 |  |
|  | Yes | 1.86 (1.73-2.00) | <0.0001 |  |  |  |  |  |  |  |  | 1.55 (1.43-1.67) | <0.0001 |
| Oral corticosteroids | No | 1 |  | Not in model | - | Not in model | - | Not in model | - | Not in model | - | 1 |  |
|  | Yes | 2.32 (2.09-2.58) | <0.0001 |  |  |  |  |  |  |  |  | 2.00 (1.80-2.23) | <0.0001 |
| Other immuno-suppressant drugs^#^ excluding oral corticosteroids | No | 1 |  | Not in model | - | Not in model | - | Not in model | - | Not in model | - | 1 |  |
|  | Yes | 2.09 (1.87-2.33) | <0.0001 |  |  |  |  |  |  |  |  | 1.63 (1.45-1.83) | <0.0001 |

RR, rate ratios; CI, confidence interval; PT, P-value for trend; IMD, index of multiple deprivation. ~ 668 (0.09%) missing values replaced by practice IMD; COPD, chronic obstructive pulmonary disease; HIV, Human Immunodeficiency virus infection; *likelihood ratio test ^#^azathioprine, biological therapy, methotrexate, 6-mercaptopurine, other immunosuppressants such as tacrolimus, sirolimus, other disease-modifying antirheumatic drugs e.g.: ciclosporin, mycophenolate, leflunomide.
